# Modulation of SOD3 Levels Is Detrimental to Retinal Homeostasis

**DOI:** 10.3390/antiox10101595

**Published:** 2021-10-12

**Authors:** Larissa Ikelle, Muna I. Naash, Muayyad R. Al-Ubaidi

**Affiliations:** Department of Biomedical Engineering, University of Houston, Houston, TX 77004, USA; likelle@central.uh.edu

**Keywords:** retina, extracellular matrix, oxidative stress, SOD3, retinal degeneration, superoxide dismutase 3

## Abstract

Retinal oxidative stress is a common secondary feature of many retinal diseases. Though it may not be the initial insult, it is a major contributor to the pathogenesis of highly prevalent retinal dystrophic diseases like macular degeneration, diabetic retinopathy, and retinitis pigmentosa. We explored the role of superoxide dismutase 3 (SOD3) in retinal homeostasis since SOD3 protects the extracellular matrix (ECM) from oxidative injury. We show that SOD3 is mainly extracellularly localized and is upregulated as a result of environmental and pathogenic stress. Ablation of SOD3 resulted in reduced functional electroretinographic responses and number of photoreceptors, which is exacerbated with age. By contrast, overexpression showed increased electroretinographic responses and increased number of photoreceptors at young ages, but appears deleterious as the animal ages, as determined from the associated functional decline. Our exploration shows that SOD3 is vital to retinal homeostasis but its levels are tightly regulated. This suggests that SOD3 augmentation to combat oxidative stress during retinal degenerative changes may only be effective in the short-term.

## 1. Introduction

Retinal dystrophies and retinopathies are a group of diseases affecting 1 in every 4000 individuals worldwide [1]. A myriad of genetic and environmental factors may be involved in their etiology [2]. However, studies have determined that oxidative stress, which is a tissue’s inability to maintain the level of reactive oxygen species (ROS) below a critical threshold [3,4], is a common modulating factor among these retinal disorders [5,6,7]. Intracellular and extracellular sources of oxidative stress appear to greatly exacerbate the effect of deleterious mutations [8,9,10]. ROSs at sustainable levels are integral to cell proliferation, host defense, gene expression, and intra/extra cellular signal transduction [11,12]. However, beyond this threshold of sustainable ROS production, the cell fails to eliminate these highly reactive molecules resulting in increased cell stress, aberrant signaling, and protein, lipid, and nucleic acid damage [13], and ultimately cell death [12,14,15]. In many cases, its additive effect and damaging self-perpetuating machinery can lead to complete functional decline, as speculated in the rod-cone cell death phenotype exhibited by retinitis pigmentosa (RP) patients [16].

The most common ROSs are the hydroxyl radical (OH), singlet oxygen (O^−^), hydrogen peroxide (H_2_O_2_), and the superoxide anion (O_2_^−^) [4]. Antioxidant enzymes are a class of highly efficient proteins designed to mitigate the injurious effects of these molecules [15]. In the retina and in other tissues, the primary lines of defense against ROSs are a superoxide dismutasing family of proteins (SOD). SODs break down two molecules of superoxide to oxygen and hydrogen peroxide [17]. SODs are metalloproteins using a metallic core to facilitate electron transfer; SOD1 and SOD3 contain copper/zinc in their metallic core [18] while SOD2 contains manganese [19]. This family of SODs is primarily distinguished by their cellular compartment. SOD1 is localized to the intracellular space, SOD2 is found in the mitochondrial matrix where oxidative metabolism occurs, and SOD3 is localized to the extracellular space [20,21].

The retina consumes more oxygen per gram than any other organ of the nervous system [22], which suggests that it may have an increased propensity for intracellular superoxide production. However, extracellular accumulation of ROSs can be equally as destructive. Superoxide and other ROSs fragment critical ECM proteins, attack cell membranes, and disrupt proper nutrient delivery and signal exchange by altering ECM permeability, the function of integrins, and overall ECM health [23,24]. Polyunsaturated fatty acids (PUFAs), major structural constituents of photoreceptors membranes, are also prime targets for extracellular ROSs [15]. NADPH oxidases (NOXs) are a family of proteins that contribute a majority of superoxide to the extracellular space [23]. Roehlecke et al., demonstrated that blue light exposure can cause NOX protein upregulation, contributing to increased levels of superoxide in the retina [25]. Moreover, the flux of reactive molecules, such as hydrogen peroxide and nitrous oxide (NO˙) to the ECM from intracellular activities, can further compound the oxidative state of the ECM by generating superoxides, hydroxyl radicals, or thiol radicals [26]. Pathogenesis, aging, high glycemic diets, and high-fat diets, were all shown to be factors in retinopathies [27,28,29] that alter NOX processes, expression level, and intracellular functions resulting in increased ROSs in the ECM [30,31].

Currently, the scope of evaluations of superoxide dismutase in the retina has been limited to SOD1 and SOD2 since both enzymes are found in cellular compartments with more direct sources of ROSs. Overexpression of mitochondrial SOD has been shown to marginally alleviate oxidative stress in the retinas of streptozotocin-induced diabetic rats [32]. Furthermore, the combination of glutathione peroxide 4 (GPX4) and SOD1 has demonstrated some efficacy in improving cone photoreceptor function in *rd10* mice [33], where oxidative injury has been implicated in late-onset cone death [34,35]. A recent study has shown that the elimination of SOD3 has shown to cause inner retinal dysfunction and increased oxidative stress in the vitreoretinal interface [36]. Thus far, there has been minimal examinations of SOD3 in the outer retina, and in the photoreceptor more specifically.

Fundamentally, the effect of oxidative injury to the ECM has been an understated contributing factor to photoreceptor dysfunction in retinal pathologies. So, the objectives of the current study were to determine the role of SOD3 in maintaining the oxidative homeostasis in the retinal ECM, to delineate its vitality to retinal protection and function, and to potentiate its role as a therapeutic target for retinal dystrophies. We show that SOD3 is upregulated by diseases and light stress, and aging. Differential centrifugation, immunoblotting, and immunofluorescence results indicated that retinal SOD3 is localized extracellularly and cytosolically, to a lesser degree. Knockout studies showed that SOD3 elimination is deleterious since its absence led to initially reduced functionality as determined by declined electroretinographic responses and a reduced number of photoreceptors that was exacerbated with age. By contrast, we observed increased functional responses and number of photoreceptors as a result of transgenic overexpression of SOD3. However, with time, overexpression caused functional and structural decline, suggesting tight regulation of SOD3 levels. Despite its initial positive effects, our study suggests that the use of SOD3 in gene therapy attempts to ameliorate retinal degeneration may not provide sustained improvement. Considering the tight regulation of this enzyme, further exploration of its regulation, and secondary functionalities would be needed for effective use in therapy.

## 2. Materials and Methods

### 2.1. Animal Use

All animal experiments were approved by the University of Houston Institutional Animal Care and Use Committee (IACUC) and adhered to the recommendations in the Guide for the Care and Use of Laboratory Animals of the National Institute of Health and the Association for Research in Vision and Ophthalmology Resolution on the Use of Animals in Research. SOD3 deficient mice (*Sod3^−/−^*) and INS2^Akita^ mice were acquired from Jackson Laboratories (Bar Harbor, ME, USA stock #003548) and were verified for the RPE65^Leu^ variant and the absence of endogenous Sod3 (in case of *Sod3^−/−^*) and rd8 spontaneous mutation. Mutant models *Rho^P23H/+^* [37], a knockin model of rhodopsin that caused retinitis pigmentosa in patients [38], were acquired from Jackson Laboratories (Bar Harbor, ME, USA, stock # 017628). *Prph2^Y/+^* knockin model [39] of pattern dystrophy mutation (Y141C) in peripherin 2 (Prph2) [40] were generated in house. All animals were purchased/generated and maintained in the C57BL6/J background. Mice were housed under cyclic lighting conditions, 12 h light (~30 lux) and 12 h dark. Animals were euthanized by CO_2_ asphyxiation followed by cervical dislocation.

### 2.2. SOD3 Overexpression in Transgenic Mice

A plasmid containing Sod3 human cDNA was targeted to rod photoreceptor cells by a 221 base pair fragment of the mouse opsin promoter [41], which directs expression specifically to the rod photoreceptors of transgenic mice [41]. Transgenic animals were generated by Cyagen (Santa Clara, CA, USA) as described [42]. Three separate lines were generated. The family with the highest transgene expression was the only line evaluated in this study. Transgenic animals were identified by polymerase chain reaction for the transgenic insert. Throughout the manuscript, overexpressing mice are designated as “Sod3^OE^”.

### 2.3. Immunoblotting

Flash frozen retinas were lysed and homogenized in IP buffer (20 mM Tris-HCl (pH 7.5), 150 mM NaCl, 1 mM Na_2_EDTA, 0.05% SDS, and protease inhibitor (Complete Protease Inhibitor Cocktail, Roche). Once tissue has been completely disrupted, samples were place on a nutator at four degrees overnight to ensure complete solubilization. Samples were then centrifuged at 25,000× *g* for 15 min. Standard Bradford Assay (Bio-Rad Protein Assay Dye Reagent Concentrate, Bio-rad, Hercules, CA, USA) was used to determine protein concentration. Between 25 to 30 µg of samples were loaded into each well. Laemmli buffer [43] was added to the protein extract and extracts were then heated at 95 °C for five min, cooled to 65 °C and loaded. Samples were loaded onto 10% acrylamide gels and run at approximately 130 volts for 90 min.

Proteins were transferred onto PVDF membranes (Immuno-Blot^®^ PVDF Membrane, EMD Millipore, Burlington, MA, USA) using a Trans-Blot Turbo transfer System (Bio-rad, Hercules, CA, USA) for 30 min at 25 volts. To ensure successful transfer, blots were treated with Ponceau dye according to methods indicated [44]. Membranes were then washed with PBST (Phosphate Buffered Saline and 0.7% Tween). Non-specific signal was blocked with 5% milk in PBST.

The primary antibodies were used to detect proteins of interest and were incubated at the dilution indicated in the subsequent descriptions. Membranes were incubated in PBST and antibody at the indicated dilution overnight at 4 °C and processed the following day. Membranes were washed 3 times (15 min each) in PBST, probed with secondary for 2 h in goat anti-rabbit, goat anti- mouse, or goat anti-chicken HRP (1:25,000) then washed three more times as previously mentioned, and lastly, imaged by chemiluminescent activation (West Pico Plus ECL substrate, Pierce, Waltham, MA, USA).

Antibodies include anti-SOD3 (sc-376948, SantaCruz Biotechnology, Dallas, TX, USA), anti-SOD3 (ab83108, Abcam, Cambridge, UK), anti-glyceraldehyde 3-phosphate dehydrogenase (GAPDH) (GW22763, Sigma Aldrich, Darmstadt, Germany) and anti-peripherin 2 (PRPH 2C1 (in house, [45])), and anti-interphotoreceptor retinoid-binding protein (IRBP) [46].

### 2.4. Fractionation

Post euthanasia, two retinas were extracted according to Winkler [47]. Freshly harvested retinas were then placed in cold 1× PBS plus protease inhibitor (Complete^™^ Protease Inhibitor Cocktail, Indianapolis, IN, USA). Samples were incubated for 15 min on ice then centrifuged at 1663× *g* (5424R Eppendorf, Enfield, CT, USA) for 1 min to pellet the retina. The supernatant contained the soluble inter-photoreceptor matrix proteins. The remaining retina was incubated in in 0.1× PBS plus protease inhibitor for an additional 30 min. Tissue was disrupted by light homogenization and was ultra-centrifuged (Sorvall™ MX Plus Series Floor Model Micro-Ultracentrifuge, S140-AT Fixed Angle Rotor, Waltham, MA, USA) at 777,000× *g* for half an hour. The supernatant, containing soluble cytosolic components, was collected and stored on ice. Pellet was then treated with 1× PBS, 1% triton X-100, and protease inhibitor and sonicated. The mixture was ultra-centrifuged at 133,000× *g*. This supernatant contains membrane components and organelles. The pellet was sonicated in 2× Laemelli buffer [43] and spun down at 25,000× *g* for 20 min. Fractionated samples were loaded onto a 10% polyacrylamide gel for immunoblot analysis and probed with anti-SOD3, anti-GAPDH, anti-Prph2-CT, and anti-IRBP.

### 2.5. Electroretinography

Electroretinography (ERG) was performed as previously described [48]. Briefly, mice were dark adapted in red cages for 2 h. After which, mice were dilated with 2% cyclopentolate (Ak-Pentolate, Accutome by Keeler, Malrvern, PA, USA) for up to 10 min. During testing mice were kept in a dark room with minimal red lighting to avoid white light exposure. Mice were anesthetized with 85 mg/kg ketamine and 14 mg/kg xylazine and rested on a slide warmer at 37 °C to maintain normal body temperature. Once completely sedated, 2.5% hypermellose ophthalmic demulcent solution (Gonak, Akorn, Inc., Lake Forest, IL, USA) was placed on the cornea to facilitate signal conductivity. Platinum coated electrodes were placed on the cornea of each eye. The mice are carefully positioned in the Ganzfield (BigShot, LKC Technologies, Gaithersburg, MD, USA), and exposed to a full field 120 cdsm^−2^ xenon flash. After five minutes of light adaptation in 30 cds/m^−2^ light animals, a subsequent 90 cdsm^−2^ flash was emitted. All recorded waveforms and data were processed through EMWIN software (LKC Technologies, Gaithersburg, MD, USA).

### 2.6. Tissue Preparation, Immunofluorescence, Histology, and Morphometry

After euthanasia, eyes were cauterized to mark the superior orientation as previously described [49,50]. Eyes were dissected from ocular cavity, and stored in modified Davidson fixative (32% ethanol, 11% acetic acid, 2% formaldehyde) or 4% paraformaldehyde in PBS for 4–6 h. For samples undergoing paraffinization, eyes were washed three times with PBS and transferred to 70% ethanol. Eyes were dehydrated in the STP 120 Spin Tissue Processor (Thermo Scientific, Waltham, MA, USA) and programmed according to the protocol described in [51]. After complete dehydration and paraffin infiltration, eyes were embedded and cut into 10 µm sections along the superior-inferior plane until the optic nerve was reached. Sections containing the optic nerve were processed in Hematoxylin (MHS16, Sigma, Burlington, MA, USA) and Eosin (H&E) (HT110116, Sigma, Burlington, MA, USA). Remaining sections were used for immunofluorescent staining and imaging. 

#### 2.6.1. Immunofluorescence

Eyes were processed, sectioned, and probed according to Chakraborty et al. [50]. After sectioning, slides were left at room temperature for 24 h before further manipulation. Samples were then rehydrated in sequential dilutions of ethanol. Once rehydrated, samples underwent sodium citrate antigen retrieval at 100 °C for 30 min and were then placed in blocking buffer (5% bovine serum albumin, 1% donkey serum, and 0.5% TritonX-100 in PBS) for 1 h to overnight. 

After blocking, sections were labeled with primary antibody diluted in the same blocking buffer as described above. Primary antibodies included anti-SOD3 at 1:250 (ab83108 Abcam, Cambridge, UK), anti-Prph2-CT at 1:500 [45], anti-Syntaxin 3b [49] at 1:250 and peanut agglutinin at 1:100 (FL-1071, Vector Laboratories, Burlingame, CA, USA) dilution. Secondary antibodies (Goat anti-Mouse Alexa Fluor 488 and Goat anti-Rabbit Alexa Fluor 647, Thermo Fisher, Waltham, Massachusetts, USA) along with DAPI were applied for 2 h. Slides were mounted with an anti-fade mounting media, then imaged using either the 40× or 63× objective (EC Plan-NeoFluar, Zeiss White Plains, NY, USA) on an LSM 800 Zeiss confocal/airyscan microscope (Zeiss, White Plains, NY, USA). Images presented are collapsed confocal stacks. 

#### 2.6.2. Histology and Morphometry

Sections across the center of the optic nerve were processed in Hematoxylin and Eosin (H&E) [52] and used for morphometric analysis. Images were taken (40×) superiorly and inferiorly of the optic nerve and at 500 µm increments. A 100 µm × 100 µm box was placed in similar locations in each image and photoreceptor nuclei were counted in that designated area using ZEN blue (Zeiss, Germany) then plotted as a spidergram. Three retinas were used per cohort. 

### 2.7. Terminal Deoxynucleotidyl Transferase dUTP Nick End Labeling (TUNEL) ASSAY

Retinas from 1 day old mice were collected in 4% paraformaldehyde (PFA) and processed as previously indicated [53]. Then, 10µm sections were cut and sections containing the optic nerve were rehydrated and stained. TUNEL staining was performed using a kit per the manufacturer’s recommendations (In Situ Death Detection Kit, Fluorescein, 11684795910, Sigma Aldrich, Darmstadt, Germany). Images of six 150 µm × 150 µm regions left and right of the center of the optic nerve were captured at 40× magnification using LSM 800 Zeiss confocal microscope. Positive cells in each region were counted and averaged. Three retinas were used for each cohort.

### 2.8. RNA Sequencing

Wildtype (WT), *Sod3*^−/−^ and *Sod3*^OE^ libraries were prepared from ∼500 ng of isolated retinal RNA with the TruSeq Stranded Total RNA Library Prep Kit according to the manufacturer’s directions (Illumina, RS-122-2001, San Diego, CA, USA). Paired-end 100-cycle sequencing was performed on HiSeq 2000 or HiSeq 2500 sequencers according to the manufacturer’s directions (Illumina, San Diego, CA, USA).

#### 2.8.1. Mapping and Data Processing

FASTQ sequences of retinal samples were mapped to mouse genome mm10 (GRCm38) downloaded from UCSC genome browser website and aligned using STAR (https://github.com/alexdobin/STAR, accessed on 8 September 2021). Aligned reads were counted per gene using HTseq (https://htseq.readthedocs.io, version 0.6.0, accessed on 8 September 2021) and their default parameters. Transcript structure and abundance were estimated using Cufflinks (http://cole-trapnell-lab.github.io/cufflinks, accessed on 8 September 2021). The expression of each gene was presented in fragments per kilobase of transcript per million mapped reads (FPKM). The matrix data of read count and FPKM were generated for preprocessing.

#### 2.8.2. Data Analysis

RNA-seq read counts were imported into BioJupies Automated Notebook Generator of RNA-seq Data Analysis [54]. Only statistically significant differential expressed genes and gene ontology terms were used in this analysis, where *p* < 0.05. Statistically significant differentially expressed genes were input into g:Profiler to enrich for gene ontological terms [55,56]. Statistical significant terms were analyzed. Overrepresented terms were included, other terms can be accessed in the Supplementary Data.

### 2.9. Statistical Analysis

Statistical testing was performed using GraphPad Prism, v.8.3.0 (GraphPad Software). Student’s *t*-test or one-way ANOVA followed by post hoc testing were the primary statistical test used in this study. All experiments (e.g., IF labeling, ERG) were done on at least three separate occasions, with a minimum sample size of three (animals or retinas).

## 3. Results

### 3.1. Elevated SOD3 Levels after Light Stress and in Retinal Pathologies

**Figure 1:**
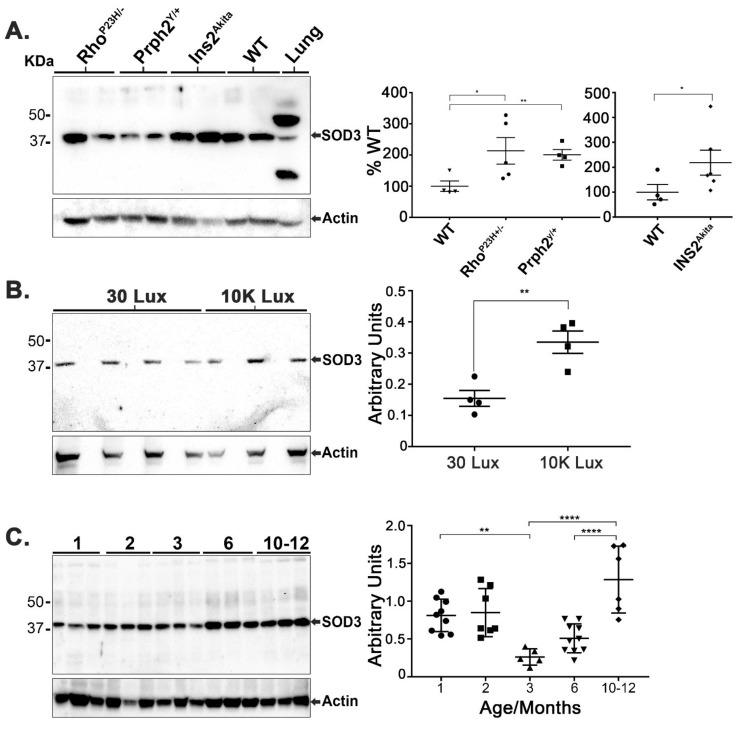
Retinal SOD3 steady state levels are modulated under pathogenic conditions, light stress, and during aging. (**A**) Retinas from 1 month old WT, *Rho^P23H+/−^* and *Prph2^Y/+^,* and 10-month-old male *INS2^Akita^* were collected and analyzed by immunoblotting for SOD3 levels. *Rho^P23H+/−^* and *Prp2^Y/+^* retinas demonstrated a statistically significant increase in SOD3 levels (where *p* = 0.0267 and 0.0028, respectively). INS2^Akita^ retinas exhibited ~2.5 fold upregulation in SOD3 levels over the age-matched WT controls (*p* = 0.0405). (**B**) 2-month-old WT agouti mice were placed under 10,000 lux light for 4 h and retinas were harvested one hour after light exposure. Compared with animals kept under 30 lux standard lighting conditions, there was a statistically significant upregulation of SOD3 (*p* = 0.0482, *n* = 4). (**C**) WT retinas were collected at 1, 2, 3, 6, and 10 to 12 months of age. Retinal SOD3 levels were steady at 1 and 2 months then sharply declined at 3 months. The drop was determined to be statistically significant by one-way ANOVA and Tukey’s post-hoc analysis (*p* = 0.0077) for the decline from 1 to 3 months (*p* < 0.001) and for increases from 3 to 10 months as well as from 6 to 10 months. (*n* = 4 to 9 for all samples). (* is *p* ≤ 0.05, ** is *p* ≤ 0.01, **** is *p* ≤ 0.0001).

**Figure 2:**
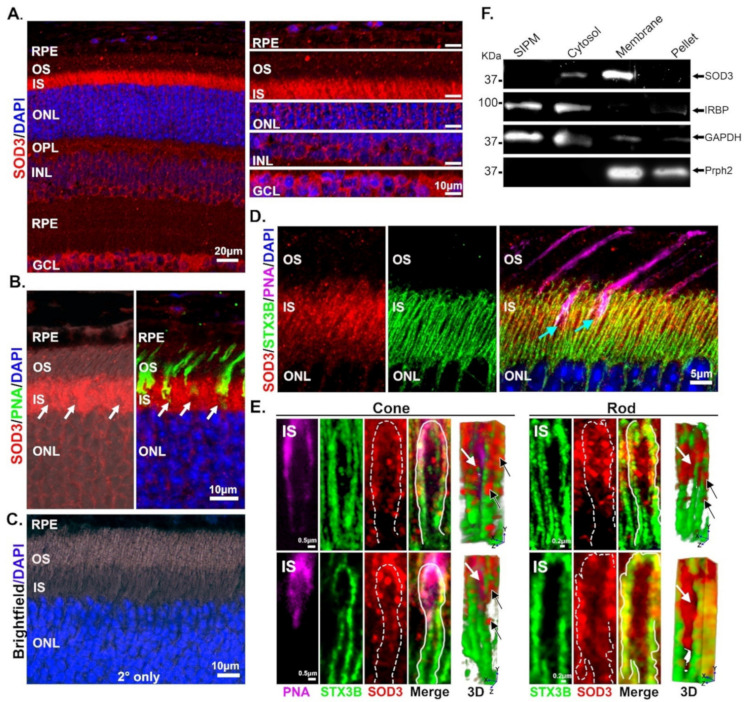
Intracellular and extracellular distribution of retinal SOD3. Immunofluorescence and fractionation experiments were performed. (**A**) Labeling with anti-SOD3 at 20 × (with 1.3 × zoom) indicates ubiquitous expression of the protein throughout the retina. Regional images were extracted from the 20× image to evaluate staining throughout the retinal strata. (**B**) Higher magnification imaging (40× with 2× zoom) demonstrates that the majority of SOD3 is found in the IS, though smaller amount is also present in RPE, OS, and around photoreceptor nuclei. White arrows point to areas of low SOD3 staining. Labeling with PNA show that these low-signal areas are cones (right image). (**C**) A secondary-only control, captured at 63×, demonstrates lack of non-specific signal. (**D**,**E**) Labeling with anti-STX3B and PNA reveal the extracellular and intracellular localization of SOD3 in rod and cone inner segments. Light blue arrows point to co-localization of STX3B and PNA to differentiate cone photoreceptors from rod photoreceptors. White arrows indicate intracellular SOD3 while black arrows indicate areas of colocalization of SOD3 and STX3B and extracellular localization of SOD3. Dashed lines/white lines delimit the IS membrane marked by STX3B labeling. (Scale bar in (**A**) is 20 µm, 10 µm in (**B**), 10 µm in (**C**), and 0.5 and 0.2 µm, respectively, in the bottom panels of (**D**,**E**). (**F**) Cellular fractionation confirms the localization of SOD3 in the cytosol and the membrane/insoluble ECM.

It is clear that pathogenesis creates critical metabolic and functional disruptions that affect the oxidative balance in the retina [8]. A healthy retina can also be affected by the insult of high-intensity light, which can generate ROSs by the photo-oxidative effect, where absorption of light can change the energy state of oxygen and lead to free radical generation (photochemical damage of the retina) [57,58]. As both conditions promote the production of ROSs in the retina, an upregulation of SOD3 may be indicative of a protective responsive effect. Prior studies have demonstrated an upregulation of SOD3 in irradiated rat retina [59]. Here, we investigated the steady-state levels of SOD3 in several pathogenic retinas with different causes: a *Rho^P23H/+^* [60] knockin model of rhodopsin that caused retinitis pigmentosa in patients [38], a *Prph2^Y/+^* knockin model [39] of pattern dystrophy mutation (Y141C) in peripherin 2 (Prph2) [40] and an *INS2^Akita^* Type I diabetic model [61]. Retinas from *Rho^P23H/+^* and *Prph2^Y/+^* were taken at 1 month of age while retinas were isolated from 10 month old *INS2^Akita^* males, and each was compared to its age-matched WT. Retinal SOD3 levels in *Rho^P23H/+^* and *Prph2^Y/+^* increased by ~38% when compared to age-matched WT. Interestingly, SOD3 level in the *INS2^Akita^* retinas increased by 2.5 folds over its age-matched WT controls (Figure 1A). SOD3 levels were also evaluated in 2-month-old WT agouti mice exposed to 10,000 lux light for a period of four hours (Figure 2B). These retinas exhibited a statistically significant increase by ~2 fold over the retinas that remained under low intensity light conditions. To further understand the regulation of retinal SOD3 during aging, we measured retinal SOD3 levels in C57BL/6 WT mice at 1, 2, 3, 6, and 10–12 month (Figure 1C). As shown in Figure 1C, retinal SOD3 levels are steady at 1 and 2 months of age, significantly drop to their lowest levels at 3 months, slightly increase at 6 months, and further increase at 10–12 months.

### 3.2. Retinal SOD3 Is Mainly Extracellular in Localization 

Subsequently, we evaluated SOD3 localization in the retina by immunofluorescence microscopy (Figure 2). We determined that SOD3 is present ubiquitously throughout the retina with varying levels at different retinal layers (Figure 2A). Although the bulk of labeling is in/around the photoreceptor inner segments (IS), labeling was also observed in photoreceptor outer segments (OS), perinuclearly, in the inner nuclear layer (INL) and at the ganglion cell layer (GCL).

At higher magnification, we can see that in the cone, SOD3 labels the extracellular space (Figure 2E left panel) as SOD3 is found around the inner segment membrane. There is also SOD3 signal inside the cone cell, but to a much lesser degree (white arrows in Figure 2E on 3D perspective) compared to the extracellular localization (black arrows). In the case of the rod photoreceptor (cells where PNA staining is absent), SOD3 labeling is also found inside the boundary (Figure 2E, right side) and outside the cell, however, we observed more intracellular staining than we observed in cones. On the 3D renders, (Figure 2E, right-most panels) white arrows point to large intracellular pools of SOD3, while black arrows show labeling on the exterior of the inner segment membrane boundary as well.

To further verify the cellular localization of SOD3, we next performed cellular fractionation to isolate specific subcellular compartments (Figure 2F). Fractions were separated by SDS-PAGE and immunoblotted for SOD3 and interphotoreceptor retinoid-binding protein (IRBP), a marker for soluble interphotoreceptor matrix (IPM) [46]; SOD3 and glyceraldehyde 3-Phosphate dehydrogenase (GAPDH, a marker for the cytosolic fraction [62]), and SOD3 and peripherin 2 (Prph2), a marker for OS membrane fractions [63]). Minor levels of SOD3 were present in the cytosolic fraction, which may represent the *de novo* synthesized SOD3, or the small pools of intracellular expression observed in Figure 2D. However, most retinal SOD3 is present in the membrane fraction (Figure 2F). It is important to mention that the membrane fraction also contains ECM insoluble components. These results are consistent with the known association of SOD3 with cell surface [64].

### 3.3. Modulation of Retinal SOD3 Levels Leads to Functional and Structural Changes

**Figure 3:**
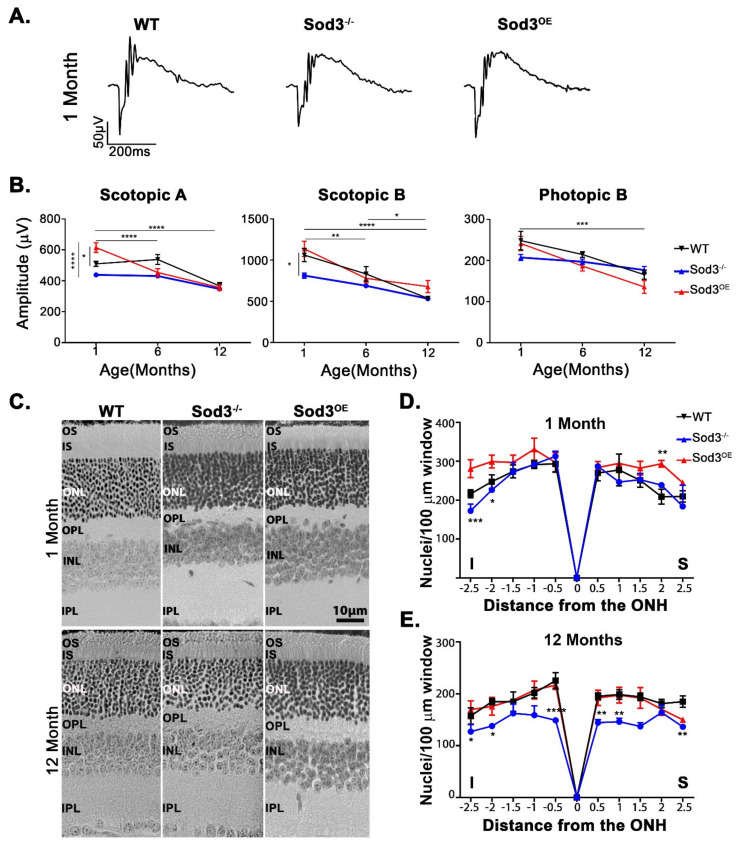
Functional testing and histologic assessments of *Sod3^−/−^* and *Sod3^OE^* retinas. (**A**) Traces of representative responses from the designated genotypes at 1 month. (**B**) At 1 month, the scotopic a-wave response, was markedly elevated in the *Sod3^OE^* retinas over the WT and knockout (*p* < 0.0200 and *p* < 0.0001 respectively). Over time scotopic a-wave responses decline in all cohorts. The most significant decline was observed in the *Sod3^OE^* retinas. Every stage of decline was statistically significant, from 1 month to 6 months *p* < 0.0001, and from 1 to 12 months, *p* < 0.0001. The scotopic b-wave response also showed statistical significant decline in WT and *Sod3*^OE^ cohorts. In the WT response from 6 months to 12 months *p* = 0.0421. In *Sod3^OE^* retinas, the decline of the scotopic b was statistically significant from 1 month to 6 months of age (*p* = 0.0027), and from 1 month to 12 months *p* < 0.0001. Photopic b responses declined in the WT from 1 month to 12 months where *p* = 0.0004 and in *Sod3^OE^* responses from 1 month to 12 months, where *p* < 0.0001. Sample size is ≥8 for each time point. Two-way ANOVA and Tukey’s post hoc analysis was used to determine statistical significance. (**C**) Micrographs of retinal sections (taken 500µm from optic nerve) of from *Sod3*^OE^, *Sod3*^−/−^, and WT. Ablation of SOD3 causes reduced number of photoreceptor nuclei at 12 months. (**D**) Morphometric analysis at 1 month demonstrated an increase in photoreceptor nuclei in *Sod3^OE^* at 2.5 mm inferior (I) of the optic nerve over the WT (not statistically significant) and the knockout (where *p* = 0.0005). At 2 mm inferior of the optic nerve and at 2 mm superior (S) of the optic nerve overexpressed retinas also showed statistically significant increase in nuclei count over the knockout (*p* = 0.0255 and *p* = 0.0080, respectively). (**E**) Morphometric analysis at 12 months indicates a decline in nuclei count from the WT in *Sod3^−/−^* retina at 2.5 mm inferior of the optic nerve (*p* = 0.0356), at 2 mm inferiorly (*p* = 0.0172), and at 0.5 mm inferiorly where there is a reduction from both the over-expresser and WT (*p* = 0.0004 and *p* < 0.0001, respectively). On the superior side (S), there are regions of statistically significant reduction from the WT and over-expresser at 0.5 mm (*p* = 0.0049 and *p* = 0.0155, respectively), at 1 mm (*p* = 0.0036 and *p* = 0.0084 respectively) and at 2.5 mm, where there is only a statistically significant reduction from the WT (*p* = 0.0079). (* is *p* ≤ 0.05, ** is *p* ≤ 0.01, *** is *p* ≤ 0.001, **** is *p* ≤ 0.0001).

To determine the role SOD3 plays in retinal homeostasis we characterized the retina of *Sod3^−/−^* mice functionally and structurally. These mice have been previously developed and used in many studies to assess SOD3′s role in different tissues except the retina (e.g., [65,66]). Functional testing by electroretinography (ERG) showed that scotopic a-wave responses significantly reduced (~15%) in 1 month old *Sod3^−/−^* mice (Figure 3B, left panel). At 12 months of age, the responses for the scotopic a-wave are similar to that observed in WT mice, albeit the decline in *Sod3^−/−^* was less steep than that observed for the WT.

Similar to the reduction in the a-wave, the scotopic b-wave responses of the *Sod3^−/−^* retina was statistically lower (23.4%) than the WT responses at 1 month of age, and continued to decline until 12 months of age (Figure 3B, middle panel), again reflecting an age-dependent decline as seen in WT retinas. Cone function was assessed by both photopic a-wave (see Supplementary Figure S1 for a-wave responses) and b-wave (Figure 3B, right-most panel). Although there was a slight reduction in cone responses at 1 month from the wildtype, both a- and b-wave responses were statistically insignificant from the WT. Photopic b-wave responses remained relatively stable, following the age-related decline exhibited by the WT. Photopic a-wave responses remained steady for 6 months. Afterwards, *Sod3^−/−^* responses steeply declined, matching the decline in WT function (see Figure S1) 

**Figure 4:**
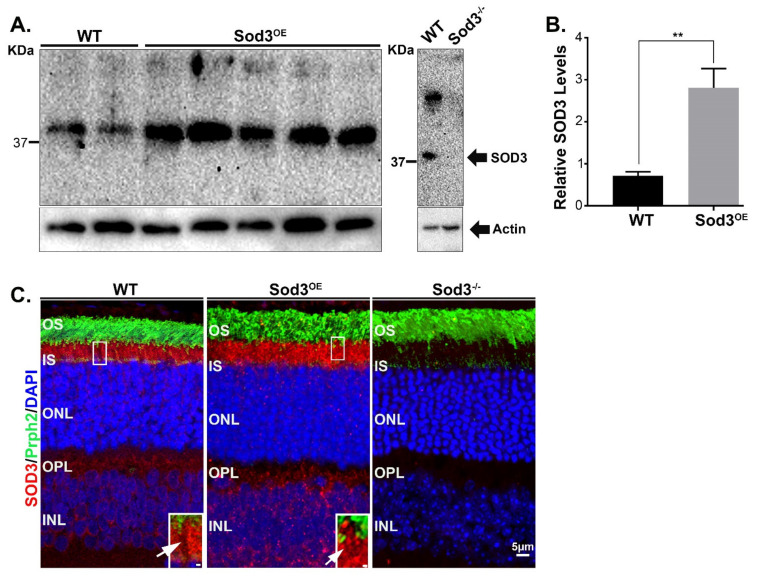
SOD3 levels and localization in *Sod3^OE^* retinas. (**A**) Representative immunoblot of 1 month WT and *Sod3^OE^* retinas demonstrate the overexpression of SOD3. Notice absence of SOD3 in *Sod3^−/−^* retinas serving here as controls. (**B**) Quantification of SOD3 overexpression (plotted in arbitrary units relative to actin) from immunoblots using five independent retinas. Statistical significance was determined by Student’s *t*-test, where *p* = 0.0086. (**C**) Retinal sections captured at 40× from 1 month old *Sod3^OE^*, *Sod3^−/−^* and WT immunolabeled with SOD3 and Prph2 antibodies. Image insets (regions marked by boxes) shows areas of low-fluorescence (white arrows) in the inner segment space. Scale bar is 5 µm and 1 µm in each inset. (** is *p* ≤ 0.01).

We next determined whether the functional variance from WT in *Sod3^−/−^* retinas resulted from structural changes, so histologic and morphometric analyses of photoreceptor nuclei were performed. At 1 month of age, there appears to be a small, yet statistically significant reduction in relation to WT in the number of photoreceptor nuclei in the outer nuclear layer at the inferior side. However, no differences from WT were observed in the central retinal regions (Figure 3C,D). The reduction in the scotopic responses is clearly a reflection of the reduced number of photoreceptors. At one year of age, there was a further decline in the number of photoreceptors throughout the entire retina (Figure 4E). The observed functional reduction and slight histological differences from the WT observed soon after full maturation of the retina (at 1 month of age) suggest that modulation of SOD3 could influence the developmental environment. Proceeding experiments will further explore this observation.

Our structural and functional studies of the *Sod3^−/−^* retina clearly suggest that SOD3 plays a role in retinal health. The important role of SOD3 is further underscored by our observation of a significant increase in its level in several retinal degenerative models (Figure 1). Altogether, this suggests that SOD3 is important in ameliorating cellular stress. Therefore, to further investigate these findings, we generated a transgenic model using a rhodopsin promoter to generate overexpression of SOD3 in the rod photoreceptor (*Sod3^OE^*). First, we assessed retinal levels of SOD3 in these mice by immunoblot and showed the levels are increased by approximately 3 folds from that in WT retinas (Figure 4A,B). Immunofluorescence analysis (imaged at 40×) showed that although SOD3 levels are elevated in transgenic mice, the pattern of localization is maintained (Figure 4C). Upon closer examination of the inner segment area, we still observe areas of low fluorescence in the overexpresser, but to a lesser degree in comparison to WT (white arrows in image inset in Figure 4C). Prph2 labeling of cone outer segments typically extend into the apical portion of the inner segment and was used here to identify cones and found that these extensions of Prph2 labeling colocalized with the areas of low SOD3 signal (Figure 4C, insets).

Functional analyses showed that *Sod3^OE^* retinas exhibited higher scotopic a-wave responses at 1 month of age (Figure 3B, left-most panel). However, these responses equalized to the WT levels by 6 months of age and remained so thereafter (Figure 3B). Scotopic b-wave was different from the scotopic a response as no improvement over WT responses was observed at one month (Figure 3B, middle panel). However, by 6 months, the scotopic b-wave amplitudes from *Sod3^OE^* retinas were reduced in comparison to those from WT retinas, but became equivalent to the WT at 1 year of age (Figure 3B, middle panel), reflecting an age-dependent decline in the function of the retina. Interestingly, overexpression of SOD3 did not seem to influence, negatively or positively, the photopic b-wave (Figure 3B, right-most panel) or a-wave (Figure S1) responses. To determine the source of the increased scotopic a-wave responses, histologic assessments of the *Sod3^OE^* retinas were performed and showed that at 1 month of age there is an appreciable increase in the number of photoreceptor nuclei (Figure 3C,D), albeit only statistically significant from the WT on the superior side. At one year of age, the number of photoreceptor nuclei was similar to that of WT reflecting cell loss from the initial increase over the WT observed at 1 month of age (Figure 3E).

### 3.4. SOD3 Overexpression Reduces Developmental Apoptosis 

**Figure 5:**
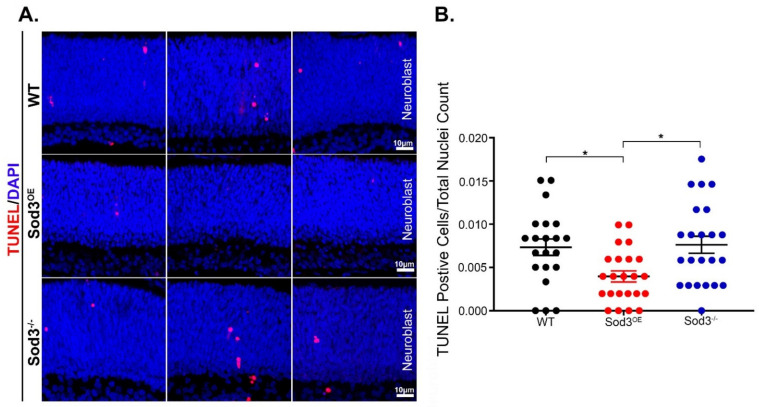
Overexpression of SOD3 reduces retinal developmental apoptosis. TUNEL assay of 1 day old retinas was performed on the indicated genotypes. Representative images from different regions of the retina were captured for each genotype (**A**) and then quantified (**B**). *Sod3^O^*^E^ retinas show a decrease in TUNEL positive cells over the WT (*p* = 0.0249). Positive cells in *Sod3^−/−^* are significantly higher than those in the over-expresser (*p* = 0.0106). The difference in apoptotic nuclei between WT and *Sod3^−/−^* was not significant. Sample size is three per cohort. (* is *p* ≤ 0.05).

The reduction in the number of photoreceptor nuclei and scotopic a-wave functional response in the *Sod3^−/−^* retina and the inverse response in the *Sod3^OE^* at 1 month supports the conjecture that SOD3 or its regulation of ROSs may be important for proper development. Since developmental apoptosis plays a critical role in determining the final photoreceptors count in the mature retina, we investigated whether overexpressing SOD3 modulates retinal developmental apoptosis by performing TUNEL assay on 1-day-old retinas (Figure 5A). Quantitative analysis of TUNEL positive cells in the different genotypes showed that while the elimination of SOD3 did not alter the number of apoptotic cells in the developing retina significantly, its overexpression led to a statistically significant reduction in TUNEL positive cells in 1-day-old retina (Figure 5B).

### 3.5. The Protective Effect of SOD3 Overexpression Is Not Maintained in the Adult Retina 

Overexpression of SOD3 has beneficial effects to the retina at early stages, but that effect seems to wane as the animal ages. Consequently, our next objective is to determine why the effects of SOD3 overexpression appears beneficial at the outset, but deleterious with time. To further elucidate the mechanisms that may contribute to the age-dependent reduction in retinal structure and function of the older *Sod3^O^*^E^, RNA-seq analysis was performed on cDNA libraries prepared from 3-month-old WT, *Sod3^OE^*, and *Sod3^−/−^* retinas. The 3-month time point was chosen to allow the investigation of early events that may have led to the reduction in the number of photoreceptors in the *Sod3^OE^* prior to any changes resulting from the loss of photoreceptors that may confound the results. Similar logic was successfully applied to the analysis performed in [67].

Raw read counts were uploaded into Biojupies automated Jupyter notebook generator, in which differential gene expression (DEG) between cohorts and gene ontological (GO) analysis were modalities chosen to isolate critical changes in gene expression. DEGs were also analyzed using g:Profiler, DAVID (Database for Annotation, Visualization and Integrated Discovery), and KEGG (Kyoto Encyclopedia of Genes and Genomes) to fully assess GO data.

**Figure 6:**
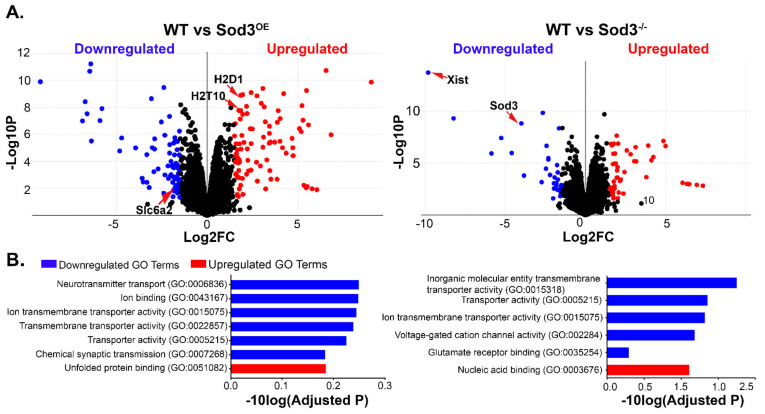
RNA sequencing data uncovers dysregulated processes in mutant animals. (**A**) Volcano plot of downregulated and upregulated genes relative to their log2FC and *p*-value. (**B**) Top identified GO terms were presented as a function of the −10log of the adjusted *p*-value. Blue indicates downregulated terms, while red corresponds to upregulated terms.

Preliminarily, differential gene expression analysis was used to select for statistically significant changes in gene expression between two cohorts. The tested groups were denoted as WT vs OE and WT vs KO. Volcano plots demonstrate the *p*-values and log fold changes of statistically significant genes (Figure 6A). There are clearly fewer differentially regulated genes in the knockout retinas. As expected, SOD3 was found significantly reduced in the *Sod3^−/−^* retinas while it was not captured as an overexpressed gene in the *Sod3^OE^* retinas, since the transgene used was a human SOD3 cDNA, and the reference genome used in this study was mouse.

Gene ontological (GO) analysis (Figure 6B) indicated that upregulated processes in the over-expresser are related to protein folding. There was also a notable upregulation in genes involved in innate immunity, such as histocompatibility antigen (*H2D1*), histocompatibility 2, K region locus 2 (*H2K2*), and histocompatibility 2, T region locus 10 (*H2-T10*), as seen in the gene list of WT vs OE (Figure S2), suggesting increased innate immune activation, which is a common feature of degenerated retinas [67]. Downregulated processes include ion binding, neurotransmission, and transporter activity. Gene ontological analysis shows that synaptic processes are also downregulated in the over-expresser. It appears that processes that require ion-protein interactions are affected in these mutant retinas demonstrated by the downregulation of proteins such as glucagon receptor (*GLP2R*), sulfate transporter (*SLC6A2*), and ATPase H^+^ Transporting V0 Subunit C (*ATP6v0c*) (See DEGs in Figure S2).

Relative to the WT, GO analysis indicates that the knockout retinas have significantly upregulated ribosomal RNA processing (Figure 6B). In the DEGs list, Ribosomal Subunit Protein L3 (*Rpl3*) is upregulated in the knockout (Figure S2). Interestingly, the most downregulated gene in the knockout cohort was X Inactive Specific Transcript (*Xist*) which is involved in X-linked gene inactivation. A recent study has linked SOD3 and gene methylation [68].

**Figure 7:**
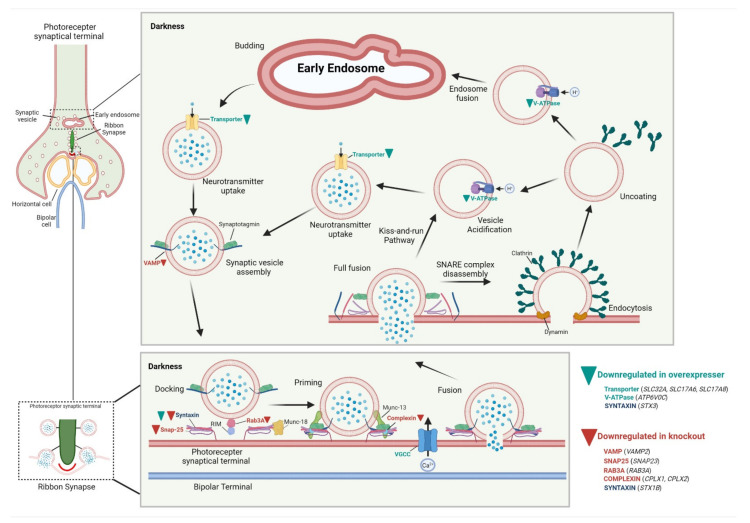
Downregulated genes of SOD3 mutants are associated with synaptic dysregulation. GO pathway analysis enriched for the Synaptic Vesicle Cycle pathway in both the downregulated gene lists of over-expresser and knockout animals. Genes downregulated the over-expresser are denoted in green along with a green arrowhead, while those downregulated in the knockout are in red with a red arrowhead. The gene downregulated in both is designated in dark blue with both red and green arrowheads.

Similar to the over-expresser, elimination of SOD3 in the knockout retinas also affects synaptic transmission as GO analysis showed downregulation of genes associated with synaptic processes. Specifically, glutamate receptor binding was enriched for in this analysis, as seen in Figure 6B. Interestingly enough, as both mutants demonstrate downregulated genes involved in synaptic transmission, synaptic vesicle cycling was a pathway enriched in both mutants, having almost the highest *p*-value amongst both sets of downregulated genes. In Figure 7, the synaptic vesicle cycling pathway, adapted from KEGG, presents the genes downregulated in over-expresser retinas (green arrowheads), knockout retinas (red arrowheads), and in both mutants (both arrowheads). Genes for transmembrane channels, such as vacuolar ATPase (denoted generally as V-ATPase in Figure 7 but as *ATP6v0c* on DEG list) and amino acid transporter genes such as *SLC732A*, *SLC7A6*, *SLC7A8* (see Supplementary Table S1, but denoted as “transporter” in Figure 7) were the components of the cycle downregulated in over-expresser retinas, and genes involved in vesicular trafficking and fusion (i.e., Ras-related protein *Rab-3A* [69], Vesicle Associated Membrane Protein 2 *VAMP2* [70], and Syntaxin 1 [71] (see Figure 7 and a full list of genes in Supplementary Table S1) were downregulated in the knockout. Superoxide, and H_2_O_2_, the product of the dismutase of superoxide, have been implicated in synaptic dysregulation [72], and this sequencing analysis has presented a potential mechanism by which these molecules are involved in regulating the synapse in the retina. Further analyses are required to validate these findings, but the data does potentiate a mechanism impacting the health of the photoreceptor.

## 4. Discussion

The role of SOD3 in retinal function and oxidative homeostasis remains fairly unexplored. The characterization of the protein has primarily focused on the lung, as it constitutes the larger portion of SOD3 enzymatic activity in the body [68]. However, because the retina is highly metabolic and susceptible to internal, environmental, and systemic oxidative damage [22], examination of the role of SOD3 seemed necessary. Our study aimed to assess the functional role of SOD3 in the retina and whether levels are tightly regulated. Our ultimate goal was to determine the applicability SOD3 as a therapeutic target that could alleviate oxidative stress in retinal degenerative diseases.

Our analysis of SOD3 steady-state levels under pathogenic and light-stressed conditions showed statistically significant upregulation of the enzyme. Punzo et al. [69] suggested that during the degenerative changes in RP, the number of photoreceptors decreases, but the oxygen supply from the choroidal blood source remains the same. This large amount of oxygen overwhelms the diminishing photoreceptors, causing ROS levels to increase which further contributes to damage. Therefore, it is reasonable to assume that the observed elevated levels of SOD3 are likely a protective response to counter this oxidative stress. The same may hold true for diabetic retinas where oxidative stress has been proposed to contribute to the observed retinopathy and continued damage even after glycemic levels are controlled [8]. Oxidative stress may damage membrane lipids, activate aberrant metabolic pathways, and disrupt mitochondrial function [13].

The increase in SOD3 levels in light stressed and aged retinas further corroborated the role of oxidative damage in regulating SOD3 levels. Photo-oxidative damage has been implicated in the effects of blue light in the retina [70,71]. Energy from light induces electron transfer in flavoproteins, chromophores, and mitochondria [71], further increasing the potential reduction of oxygen to superoxide. In addition to the changes we observe as function of light stress and disease, SOD3 levels also drastically change as a consequence of development/aging. The free radical theory of aging purported that aging was the result of ROS accumulation and the subsequent decline of antioxidant machinery [72,73,74]. However, we now know that the aging process is a complex nexus of remodeling events, and regulatory and metabolic changes [75,76,77]. Operating on the hypothesis that SOD3 upregulation is a stress or protective response induced by changes to ROS levels, our evaluation of SOD3 levels over time does demonstrate that retinal maturation is a dynamic process marked by significant fluctuations in free radical generation.

SOD3 appears ideally positioned to mitigate extracellular and intracellular oxidative insults, being localized to the cell surface and intracellularly (though, more in rods than in cones). Therefore, we hypothesized that its elimination or overexpression may have significant opposing effects on retinal homeostasis. We observed an appreciable decline in retinal responses in the knockout and over-expresser that was likely exacerbated by aging. However, the most consequential difference was the increased rod response and photoreceptor count in 1 month old *Sod3*^OE^ retinas and the sustained functional reduction in of SOD3 knockout animals. Photopic responses, on the other hand, indicated that SOD3 modulation did not significantly affect the health of cone photoreceptors. These findings complement our localization studies, where we observe SOD3 is distributed both intra and extracellularly in the rod, while in the cone, expression is predominately extracellular. The localization behavior of SOD3 in the rod may indicate that the enzyme has a more vital role in rod health than the cone, however, further studies beyond immunofluorescence are required to substantiate this observation.

To determine how overexpression of SOD3 can lead to increased ERG responses and higher number of photoreceptors, we performed TUNEL assay and showed that a significant reduction in developmental apoptosis contributed to the increased photoreceptor cell count and function. It is accepted that superoxide, H_2_O_2_, and other ROSs are critical for development and maturation [78]. (Also suggested by Figure 1C). During morphogenesis, ROS are highly regulated, and progenitor and differentiated cells have shown markedly differing levels of ROSs [79]. These ROSs may have a significant role in determining cellular fates, proliferation, tissue morphogenesis, and survival [79]. Dysregulation in this controlled environment may lead to excessive apoptosis during development [79]. Elevated levels of SOD3 at critical developmental stages, appear to allow *Sod3*^OE^ retinas to surmount developmental apoptosis, by affectively reducing superoxide levels that contribute to hydrogen peroxide production. Knockout animals (where superoxide levels are theoretically elevated) may have experienced a disruption in this precarious balance of ROSs needed for proper development, and consequently, showed reduced function and number of photoreceptors from the outset.

Overexpressing SOD3 conferred a protective effect early in the life of the animal. However, that effect was not sustained with age as evidenced by the decline in ERG and photoreceptor count after 1 year of overexpression. Though the responses converged to WT levels at 1 year, it does suggest that the positive effect could not be sustained. It can even be argued that constitutive overexpression for long periods of time may be deleterious, since the over-expresser retinas demonstrated a reduction in photoreceptor nuclei at 12 months. Supplementation with antioxidants over long periods of time was shown to have negative effects [80]. Could the same logic be applied to sustained SOD3 overexpression? 

We performed RNA sequencing on retinas from WT, knockout, and over-expresser animals to determine what genes and processes may be affected by modulating SOD3 levels. Computational analysis revealed that many homeostatic pathways could be disrupted, but enrichment analysis indicated that the most substantial effect may involve important electrochemical processes. Ionic exchange, ion-mediated transmembrane transport, and synaptic dysregulation were themes that appeared in both the knockout and *Sod3^OE^* profiles. All of these events require the participation of the cellular membrane and the primary effects of SOD3 activity are extracellular in nature. Cellular redox balance is critical for ionic flux, proper signal transduction, and synaptic function [81]. Synaptic dysregulation has been directly linked to retinal dystrophies and photoreceptor degeneration [82,83,84]. Studies addressing SODs and retinal synapses are limited, but there has been thorough examination of the complex interplay of SODs, superoxide, H_2_O_2_, and synaptic health and plasticity in the brain [85]. Superoxide has been linked to the activation of important extracellular signal-regulated kinases (ERKs) [86] which are crucial for organizing the synapse. Superoxide has also been shown to be essential for induction of long-term potentiation (LTP), which is the molecular basis of synaptic plasticity. Briefly, overexpression of SOD3, and subsequent lower levels of superoxide, has shown to impair LTP in mice [87]. Given this relationship examined in the neural cells, it is not a far leap to intimate that modulation of SOD3 and thus the bioavailability of superoxide and H_2_O_2_ may have effects in the retinal synapse as well. Nevertheless, the RNA seq data provides a preliminary understanding of the potential mechanism affecting photoreceptor decline in both mutant retinas. Further examination may provide insight into the role of superoxide and hydrogen peroxide to the retinal synapse, and how they contribute to the decline in photoreceptor function.

Fundamentally, the changes we observed in the knockout and overexpression models suggest that both superoxide flux and SOD3 expression patterns play a wide array of roles in systemic homeostasis. It is this vital oxidative balance in the retina that is critical for cell structure, function, and metabolism. Aberrations to this balance contribute to the etiologies of many retinal dystrophies. By isolating the properties of SOD3 and illustrating the effects of its ablation and overexpression, we have presented a viable therapeutic target for retinal diseases complicated by the secondary effects of oxidative stress. However, given that the protein appears tightly regulated and has secondary functions, outside of its dismutasing activity, careful analysis would be needed to determine therapeutic dosing and duration of supplementation. Antioxidants are not novel remedies, but the extracellular localization, ubiquitous distribution, and multi-functional role of this particular enzyme in the retina could improve outcomes when carefully delivered in conjunction with other therapies. As previously stated, oxidative stress presents as a consequence of a primary insult, whether genetic or environmental. By addressing both the primary pathology and restoring oxidative homeostasis, we can significantly improve prognoses for this group of debilitating diseases.

## 5. Conclusions

In summation, this study provides insight into the role of SOD3 in the retina by demonstrating that SOD3 affords a protective effect since it is upregulated after environmental and pathogenic insult. Under normal conditions, SOD3 is important for homeostasis and its ablation led to functional and structural abnormalities. On the other hand, overexpression resulted in augmented rod photoreceptor function at an early age, but this advantage was lost as the animals got older. RNA sequencing demonstrated several homeostatic processes that are affected by SOD3 gene perturbations, with synaptic processes being one of critical importance. Ultimately, this enzyme is important to normal retinal functionality and could be a viable antioxidant therapy contingent upon further investigations of its regulation and secondary functions.

## Data Availability

Data are contained within the article and Supplementary Materials.

## Supplementary Materials

The following are available online at https://www.mdpi.com/article/10.3390/antiox10101595/s1, Figure S1: Photopic A-wave responses, Figure S2 List of DEGs in knockout and over-expresser retinas, Table S1: Full gene list and gene ontology data from knockout and over-expresser retinas.
