# Supplementary material for: Modulation of SOD3 Levels Is Detrimental to Retinal Homeostasis
Source: Antioxidants (Basel). 2021 Oct 12;10(10):1595. doi: 10.3390/antiox10101595 (PMC8533566; doi:10.3390/antiox10101595)
Supplement: Supplementary file 1 [file antioxidants-10-01595-s001.zip › Figures S1 and S2.pdf]

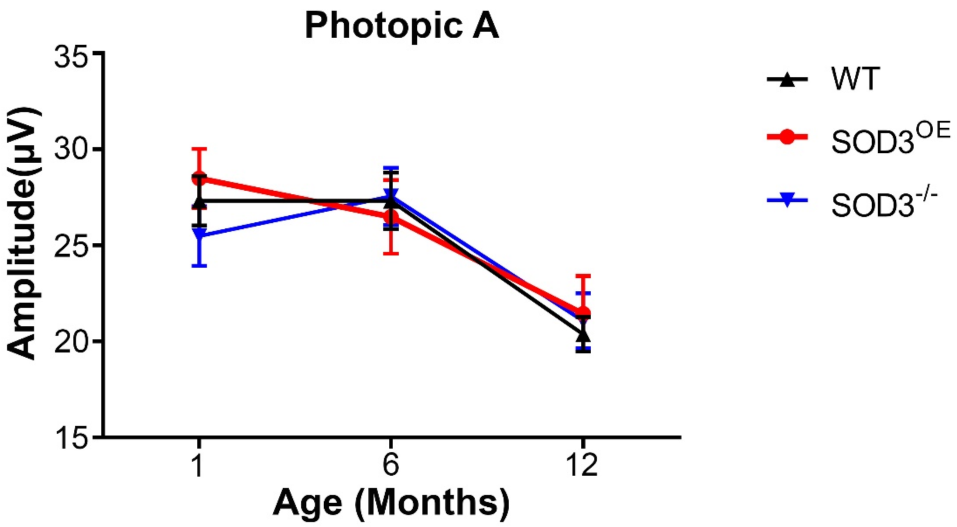

Figure S1. Photopic A-wave responses from WT, *Sod3*$^{-/-}$, and *Sod3*^{OE} retinas at 1 month, 6 months, and 12 months of age show slight variation between all cohorts at 1 month (statistically insignificant) and a steep decline in all groups after 6 months of age. No statistical significance was determined amongst cohorts or between time points. N=8 for all groups and time points.

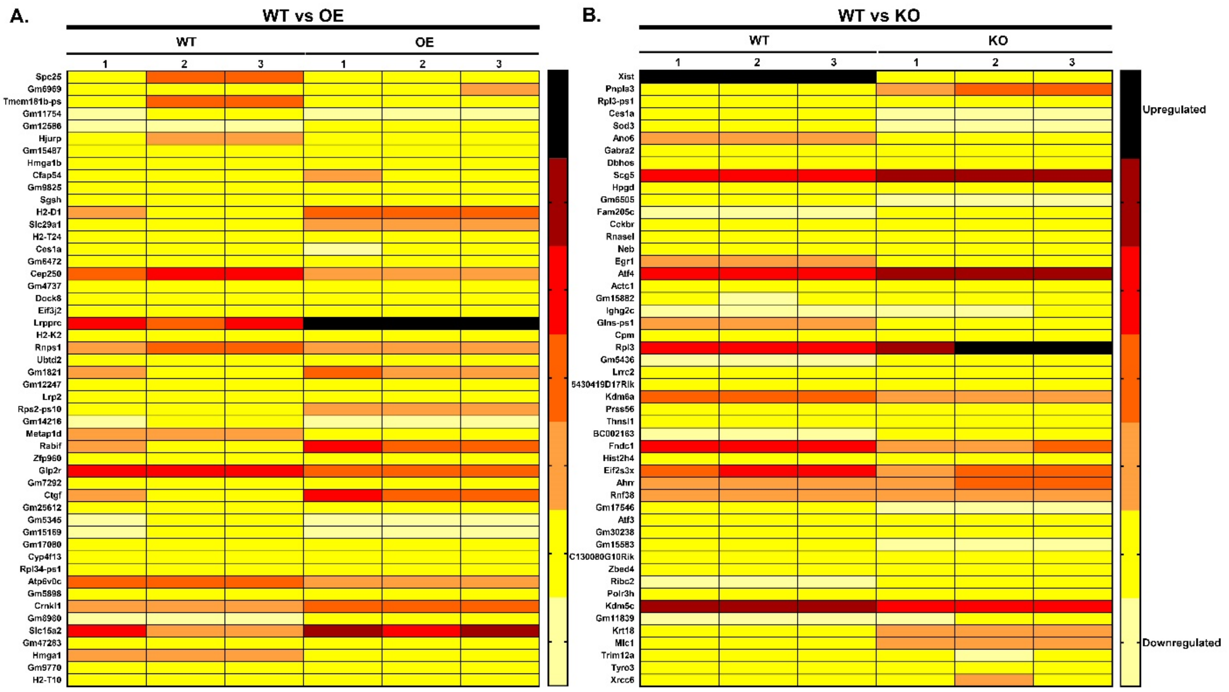

Figure S2. Libraries were prepared on retinas from WT, *Sod3*$^{-/-}$, and *Sod3*^{OE}, denoted above as WT, KO, and OE. (**A**,**B**) Top 50 differential expressed genes (DEGs) for each compared cohort is listed.
