# Supplementary material for: Modulation of SOD3 Levels Is Detrimental to Retinal Homeostasis
Source: Antioxidants (Basel). 2021 Oct 12;10(10):1595. doi: 10.3390/antiox10101595 (PMC8533566; doi:10.3390/antiox10101595)
Supplement: Supplementary file 1 [file antioxidants-10-01595-s001.zip › Table S1.pdf]

Full List of Statistically Significant DEGs in WT vsKO

GENES	logFC	AveExpr	t	P.Value	adj.P.Val	B
ENSMUSG00000086503//Xist	-9.812454343	6.4901309	-50.14806306	2.00E-14	3.48E-10	22.6002
ENSMUSG00000071047//Ces1a	-8.224230257	-0.851696975	-19.89164478	5.03E-10	2.19E-06	8.510913
ENSMUSG00000078648//LOC102634389	-4.597626161	-2.140466244	-9.559658763	1.09E-06	0.000513	3.782282
ENSMUSG00000072941//Sod3	-3.999811275	1.676020927	-17.91999218	1.55E-09	5.39E-06	11.47205
ENSMUSG00000085715//Tsix	-3.835943074	-1.880618932	-5.574348321	0.000161839	0.011203	0.817439
ENSMUSG00000060441//Trim5	-2.464116278	-1.198122951	-6.636960249	3.54E-05	0.005799	2.206894
ENSMUSG00000022129//Dct	-2.320002591	2.753112662	-8.239025999	4.69E-06	0.00148	4.59918
ENSMUSG00000066258//Trim12a	-2.314784251	1.569481997	-8.516225722	3.40E-06	0.001206	4.931166
ENSMUSG00000085336//Gm11732	-2.212829158	-1.79206173	-5.670483782	0.00014014	0.010714	0.954309
ENSMUSG00000059040//Eno1b	-1.971454061	6.383645676	-4.483704551	0.000907947	0.024286	-1.38912
ENSMUSG00000037014//Sstr4	-1.961041837	-1.597829952	-3.809131874	0.002857185	0.041439	-1.41686
ENSMUSG00000044814//Olfr543	-1.861027646	-0.189017375	-3.910186983	0.002398098	0.037701	-1.26252
ENSMUSG00000004651//Tyr	-1.806584771	-0.609064991	-4.335391798	0.001162569	0.026933	-0.59413
ENSMUSG00000025359//Pmel	-1.773234391	0.956718512	-7.241533729	1.59E-05	0.003737	3.428382
ENSMUSG00000056481//Cd248	-1.743941339	-1.193721732	-3.659967962	0.003707329	0.047225	-1.6271
ENSMUSG00000064210//Ano6	-1.647020409	4.95391416	-16.19249047	4.59E-09	1.14E-05	11.46249
ENSMUSG00000049112//Oxtr	-1.634170824	0.477113602	-3.656503761	0.003729919	0.047325	-1.74646
ENSMUSG00000022112//Gpc5	-1.50859115	-0.958815878	-4.243806307	0.001356231	0.029309	-0.743
ENSMUSG00000024806//Mlana	-1.464913508	-1.001753418	-3.914715982	0.002379408	0.03755	-1.2371
ENSMUSG00000057596//Trim30d	-1.450607307	-0.704311867	-3.638974115	0.003846427	0.048114	-1.66822
ENSMUSG00000000560//Gabra2	-1.43806211	4.40922435	-16.29173003	4.30E-09	1.14E-05	11.52869
ENSMUSG00000055994//Nod2	-1.392995502	0.879503666	-3.732701122	0.00326424	0.044423	-1.68067
ENSMUSG00000032911//Cspg4	-1.331328519	0.405936845	-3.955588121	0.002217421	0.036419	-1.24078
ENSMUSG00000005148//Klf5	-1.328969918	-1.220019764	-4.179666043	0.001511718	0.031011	-0.85227
ENSMUSG00000102101//Zbtb11os1	-1.24134537	-0.650888479	-4.147972987	0.001595306	0.031751	-0.87817
ENSMUSG00000102692//Dchs2	-1.215173351	-0.546794458	-3.849780747	0.002662452	0.039823	-1.3415
ENSMUSG00000005994//Tyrp1	-1.214765301	2.62270975	-4.426415209	0.000998576	0.025366	-0.84614
ENSMUSG00000070469//Adamtsl3	-1.179555582	-0.037870146	-4.533592663	0.000836051	0.023131	-0.29263
ENSMUSG00000046312//AI464131	-1.171817041	2.106434901	-7.490464739	1.16E-05	0.002966	3.742563
ENSMUSG00000036480//Prss56	-1.132814428	3.24951797	-10.20526882	5.65E-07	0.000338	6.692267
ENSMUSG00000046637//Ttc34	-1.110060541	1.989863636	-3.634063547	0.003879732	0.048392	-2.07551
ENSMUSG00000030761//Myo7a	-1.101745389	4.489135077	-6.56279954	3.91E-05	0.006027	2.137643
ENSMUSG00000038418//Egr1	-1.026303324	5.523128805	-11.89443851	1.18E-07	0.000148	8.101123
ENSMUSG00000018830//Myh11	-1.020937262	1.823789258	-4.501022763	0.000882281	0.02399	-0.55805
ENSMUSG00000036098//Myrf	-1.017740485	1.11134188	-4.36493416	0.001106457	0.026536	-0.66159
ENSMUSG00000027298//Tyro3	-0.970773469	3.586067789	-8.489081879	3.51E-06	0.001218	4.796743
ENSMUSG00000097615//Gm2061	-0.970206773	-0.149301049	-3.681772818	0.003568336	0.046303	-1.63398
ENSMUSG00000078949//R3hdml	-0.91849192	2.942155051	-3.725479662	0.003305679	0.044611	-2.13528
ENSMUSG00000073609//D2hgdh	-0.915985214	3.491551704	-4.386053676	0.001068084	0.026286	-1.10713
ENSMUSG00000058183//Mmel1	-0.903542495	2.230306441	-4.040441571	0.001916643	0.034798	-1.42137
ENSMUSG00000071984//Fndc1	-0.896900953	6.884424524	-10.05363688	6.57E-07	0.000357	6.221713
ENSMUSG00000068196//Col8a1	-0.878717276	1.740537144	-3.721046336	0.003331387	0.044797	-1.86935
ENSMUSG00000032327//Stra6	-0.876384765	2.187406817	-5.78199727	0.000118781	0.010068	1.40123
ENSMUSG00000035594//Chrna5	-0.875887727	2.740940422	-6.217476372	6.33E-05	0.007692	1.952296
ENSMUSG00000052726//Kcnt2	-0.871815977	0.808849964	-3.930623357	0.00231495	0.037105	-1.33577
ENSMUSG00000021091//Serpina3n	-0.867278089	2.615235973	-3.862439421	0.002604657	0.039434	-1.81798
ENSMUSG00000073680//Tmem88b	-0.865110564	0.377276522	-4.053786219	0.001873347	0.03448	-1.077
ENSMUSG00000097023//Mir9-3hg	-0.864532375	0.613383466	-4.278796967	0.001278538	0.02848	-0.73661
ENSMUSG00000020183//Cpm	-0.859659477	4.015554662	-11.05133478	2.51E-07	0.00019	7.454804
ENSMUSG00000031613//Hpgd	-0.85509325	4.615859675	-13.54911605	3.03E-08	5.27E-05	9.567585
ENSMUSG00000074934//Grem1	-0.826374426	0.791711276	-4.358785775	0.001117897	0.026544	-0.62769
ENSMUSG00000029361//Nos1	-0.816225143	3.489186262	-4.199578054	0.001461549	0.03047	-1.42887
ENSMUSG00000021799//Opn4	-0.798302389	1.973115718	-4.636804371	0.000705625	0.021274	-0.36134
ENSMUSG00000042622//Maff	-0.783999009	0.759153255	-3.696099299	0.003479948	0.045841	-1.72261
ENSMUSG00000049555//Tmie	-0.775362142	1.700986297	-4.341037875	0.00115162	0.026894	-0.80198
ENSMUSG00000018169//Mfng	-0.772188498	2.085382157	-5.423823548	0.000203289	0.012241	0.874412
ENSMUSG00000073643//Wdfy1	-0.76972681	5.446090739	-4.749879064	0.000586952	0.019688	-0.84532
ENSMUSG00000020121//Srgap1	-0.756806203	5.077409972	-4.935323638	0.000435629	0.016934	-0.483
ENSMUSG00000059146//Ntrk3	-0.74702914	3.340061582	-5.8787932	0.000103043	0.009626	1.34155

ENSMUSG00000012819//Cdh23	-0.742716409	1.049303619	-3.762783303	0.003097311	0.043147	-1.65949
ENSMUSG00000026814//Eng	-0.738574139	2.068231615	-3.663961977	0.003681458	0.047068	-2.04071
ENSMUSG00000053441//Adamts19	-0.73323231	1.519695573	-4.110501845	0.001700385	0.032784	-1.15637
ENSMUSG00000038943//Prc1	-0.727915447	3.164656927	-4.733761753	0.000602496	0.019873	-0.44612
ENSMUSG00000046480//Scn4b	-0.725440613	2.605240751	-4.33117469	0.001170819	0.027037	-1.00434
ENSMUSG00000006611//Hfe	-0.720879433	4.279545398	-7.921257648	6.83E-06	0.001947	4.000541
ENSMUSG00000033170//Card10	-0.716433289	2.534130606	-6.508534853	4.22E-05	0.006027	2.399117
ENSMUSG00000045679//Pqlc3	-0.713359912	2.342811909	-5.18699996	0.000292919	0.014137	0.459402
ENSMUSG00000040488//Ltbp4	-0.71193204	3.137058172	-5.032414341	0.000373388	0.015785	0.052412
ENSMUSG00000031805//Jak3	-0.709047234	0.81168402	-3.866039164	0.00258846	0.039338	-1.44449
ENSMUSG00000090799//Klhl33	-0.707897909	2.690746973	-4.943632259	0.000429897	0.016852	7.22E-05
ENSMUSG00000005540//Fcer2a	-0.705800299	0.726082028	-3.621460103	0.003966577	0.048948	-1.8436
ENSMUSG00000051599//Pcdhb2	-0.700581215	2.300774108	-4.28482667	0.001265627	0.02832	-1.01747
ENSMUSG00000042817//Flt3	-0.699367546	2.010471436	-5.121797792	0.000324362	0.014912	0.415402
ENSMUSG00000044254//Pcsk9	-0.694682543	1.396560979	-3.931783799	0.002310319	0.037065	-1.43678
ENSMUSG00000021294//Kif26a	-0.691570571	2.311308117	-3.86517581	0.002592335	0.039338	-1.74377
ENSMUSG00000043857//Mgat5b	-0.675816406	3.782891983	-6.566372675	3.90E-05	0.006027	2.267868
ENSMUSG00000026483//Fam129a	-0.664277578	5.215273348	-3.652661693	0.00375514	0.047451	-2.73394
ENSMUSG00000017754//Pltp	-0.661921687	2.730112	-4.672520113	0.000665628	0.02099	-0.4552
ENSMUSG00000032303//Chrna3	-0.658768913	3.088149338	-3.879608986	0.002528335	0.038852	-1.89716
ENSMUSG00000058153//Sez6l	-0.657811292	5.655501861	-6.193767178	6.55E-05	0.007692	1.432877
ENSMUSG00000022476//Polr3h	-0.657719694	4.260045685	-8.83453219	2.38E-06	0.000931	5.105954
ENSMUSG00000040035//Disp2	-0.656111657	6.646030529	-6.242321534	6.11E-05	0.007586	1.431106
ENSMUSG00000037369//Kdm6a	-0.653180602	6.582839241	-10.29560917	5.17E-07	0.000321	6.48607
ENSMUSG00000031391//L1cam	-0.648971661	5.654680591	-4.919317917	0.0004469	0.017308	-0.58546
ENSMUSG00000052026//Slc6a7	-0.647381482	2.826768001	-5.989522817	8.77E-05	0.008759	1.603222
ENSMUSG00000030110//Ret	-0.642170769	5.477991213	-6.18075658	6.67E-05	0.007725	1.43343
ENSMUSG00000025161//Slc16a3	-0.632680844	4.983107752	-5.55737691	0.000166027	0.011209	0.541142
ENSMUSG00000035407//Kank4	-0.628417938	2.2317161	-4.175538243	0.001522341	0.031061	-1.1893
ENSMUSG00000025020//Slit1	-0.628250667	3.98129889	-5.078518991	0.00034719	0.015272	-0.04907
ENSMUSG00000039057//Myo16	-0.628045478	4.114912925	-6.550435914	3.98E-05	0.006027	2.184982
ENSMUSG00000045689//Pcdhb4	-0.626252169	2.571242028	-5.569248334	0.000163085	0.011209	1.01431
ENSMUSG00000018849//Wwc1	-0.620399555	3.12704679	-4.440659048	0.000975192	0.025325	-0.93256
ENSMUSG00000033633//Clec18a	-0.612494586	2.145756374	-3.946533536	0.002252292	0.036636	-1.56535
ENSMUSG00000070576//Mn1	-0.610827347	2.594803131	-4.803345694	0.000538339	0.018562	-0.21011
ENSMUSG00000045215//Asxl3	-0.610139977	3.084488625	-5.004197235	0.000390445	0.016045	0.017423
ENSMUSG00000042684//Npl	-0.60818086	3.914529717	-4.801216575	0.000540191	0.018585	-0.49445
ENSMUSG00000049537//Tecrl	-0.606289993	1.686326412	-3.698348888	0.003466275	0.045765	-1.89728
ENSMUSG00000032452//Clstn2	-0.601836282	4.795077445	-6.001610724	8.62E-05	0.008708	1.258422
ENSMUSG00000061740//Cyp2d22	-0.600768064	4.571785745	-7.11109823	1.88E-05	0.003806	2.891697
ENSMUSG00000025221//Kcnip2	-0.600087233	2.623636941	-4.854268169	0.000495971	0.018122	-0.13235
ENSMUSG00000036040//Adamtsl2	-0.595167951	2.792490019	-4.674781476	0.000663177	0.02095	-0.46473
ENSMUSG00000054013//Tmem179	-0.59413273	4.106019015	-7.375662743	1.34E-05	0.003237	3.323632
ENSMUSG00000025332//Kdm5c	-0.592828435	7.921750992	-8.82092923	2.41E-06	0.000931	4.815363
ENSMUSG00000086118//Gm14169	-0.592346658	1.823533244	-3.708304167	0.003406431	0.045327	-1.90927
ENSMUSG00000033685//Ucp2	-0.590714473	3.534932191	-3.801089867	0.002897427	0.041744	-2.13951
ENSMUSG00000021259//Cyp46a1	-0.584727459	3.616307911	-4.614354556	0.000732052	0.021664	-0.74525
ENSMUSG00000007594//Hapln4	-0.584133083	2.744296681	-4.300883728	0.001231904	0.027943	-1.08673
ENSMUSG00000023192//Grm2	-0.582034162	2.921655749	-3.766265571	0.003078565	0.043033	-2.05837
ENSMUSG00000042453//Reln	-0.581817362	7.630360628	-4.358579297	0.001118283	0.026544	-1.65754
ENSMUSG00000017167//Cntnap1	-0.581259601	6.216213327	-6.064062638	7.88E-05	0.008438	1.187872
ENSMUSG00000015829//Tnr	-0.57697783	7.013780044	-5.767786642	0.000121298	0.010078	0.692654
ENSMUSG00000043301//Kcnj6	-0.576910003	3.411030324	-4.751886725	0.000585045	0.019688	-0.46914
ENSMUSG00000036422//Pcdh8	-0.575226439	3.639302318	-4.448943082	0.000961856	0.025207	-1.03198
ENSMUSG00000026185//Igfbp5	-0.57369888	6.042479703	-8.147686004	5.22E-06	0.001619	4.071647
ENSMUSG00000066129//Kndc1	-0.570628644	4.137115759	-5.214568277	0.000280611	0.013851	0.141556
ENSMUSG00000029778//Adcyap1r1	-0.569781973	2.784261864	-3.93434571	0.00230013	0.037057	-1.73093
ENSMUSG00000031543//Ank1	-0.569508103	5.951026094	-7.121656786	1.86E-05	0.003806	2.734904
ENSMUSG00000097571//Jpx	-0.566617122	3.575185306	-4.624488675	0.000719996	0.021402	-0.71918
ENSMUSG00000021250//Fos	-0.565457527	4.32055679	-5.694253461	0.000135266	0.010587	0.868474
ENSMUSG00000009734//Pou6f2	-0.565267334	4.168662788	-5.912941424	9.80E-05	0.009463	1.233152

ENSMUSG00000020653//Klf11	-0.556724663	2.625721314	-4.821585173	0.000522741	0.018402	-0.18647
ENSMUSG00000040606//Kazn	-0.556540911	4.391000093	-5.256410533	0.000262964	0.01343	0.16072
ENSMUSG00000031376//Atp2b3	-0.554162633	4.848868472	-5.745898618	0.000125288	0.010078	0.857421
ENSMUSG00000022177//Haus4	-0.554032593	2.776823802	-5.018728043	0.000381561	0.015909	0.104653
ENSMUSG00000037217//Syn1	-0.553898314	6.766045033	-5.632283238	0.000148367	0.010786	0.490063
ENSMUSG00000054003//Tdrd9	-0.553074528	4.465426067	-4.475159288	0.000920899	0.024354	-1.15758
ENSMUSG00000053465//Hs6st3	-0.551019585	2.541302337	-4.427298132	0.00099711	0.025366	-0.82705
ENSMUSG00000030747//Dgat2	-0.549992889	3.106624551	-4.997931449	0.000394343	0.01616	0.002561
ENSMUSG00000097767//Miat	-0.544239601	5.122061611	-3.879459013	0.002528992	0.038852	-2.31456
ENSMUSG00000028980//H6pd	-0.542709905	3.413330724	-5.96132079	9.14E-05	0.00897	1.451837
ENSMUSG00000049313//Sorl1	-0.542258452	6.834140648	-5.121059386	0.000324738	0.014912	-0.33661
ENSMUSG00000055632//Hmcn2	-0.538897193	3.141840176	-5.53894666	0.000170706	0.011364	0.858601
ENSMUSG00000027849//Syt6	-0.536577604	4.153527133	-4.868092886	0.000485086	0.017933	-0.43125
ENSMUSG00000019828//Grm1	-0.534951548	4.421128969	-5.332796656	0.000233711	0.013065	0.278245
ENSMUSG00000028364//Tnc	-0.534409721	4.104890836	-4.900351728	0.000460655	0.017514	-0.36791
ENSMUSG00000029675//Eln	-0.532656596	2.334879911	-4.354940881	0.001125114	0.026598	-0.90555
ENSMUSG00000023017//Asic1	-0.531422349	3.90738456	-5.837711036	0.000109433	0.009955	1.167808
ENSMUSG00000074892//B3galt5	-0.527739665	3.009441756	-4.822900984	0.000521634	0.018402	-0.26476
ENSMUSG00000030089//Slc41a3	-0.527193539	2.273288792	-4.371919618	0.001093607	0.026536	-0.86376
ENSMUSG00000039809//Gabbr2	-0.523594677	6.222443309	-6.856212611	2.63E-05	0.004623	2.345132
ENSMUSG00000033278//Ptprm	-0.522896709	4.409477537	-6.760028058	3.00E-05	0.005154	2.431595
ENSMUSG00000035150//Eif2s3x	-0.522732544	6.848436314	-9.84919966	8.07E-07	0.000413	6.004523
ENSMUSG00000070802//Pnmal2	-0.521488963	7.180847671	-6.567101433	3.89E-05	0.006027	1.887938
ENSMUSG00000028438//Kif24	-0.520674355	2.906159111	-5.075071194	0.000349081	0.015282	0.169501
ENSMUSG00000061654//Spry3	-0.520581632	4.888001226	-5.194308529	0.000289602	0.014131	-0.02683
ENSMUSG00000017756//Slc12a7	-0.519817409	3.405654432	-4.838626355	0.000508598	0.018146	-0.32347
ENSMUSG00000051111//Sv2c	-0.5190574	4.50544293	-3.943820154	0.002262852	0.036711	-2.09352
ENSMUSG00000031227//Magee1	-0.513900738	5.88593677	-5.805385739	0.000114757	0.009972	0.818338
ENSMUSG00000025813//Homer2	-0.513444894	5.025750996	-5.482426011	0.000185949	0.011749	0.415922
ENSMUSG00000055407//Map6	-0.51278684	6.659380243	-5.391392124	0.000213616	0.012632	0.11109
ENSMUSG00000027254//Map1a	-0.512627038	7.66174833	-5.338332804	0.000231729	0.013065	-0.0109
ENSMUSG00000020806//Rhbdf2	-0.509906583	3.279335192	-4.721974817	0.000614138	0.020017	-0.49056
ENSMUSG00000020173//Cobl	-0.5066023	4.794214702	-6.737681537	3.09E-05	0.005259	2.337733
ENSMUSG00000016349//Eef1a2	-0.505782569	7.897944508	-5.896118279	0.000100469	0.009487	0.863708
ENSMUSG00000039059//Hrh3	-0.503857627	3.210060206	-5.070272527	0.000351729	0.015293	0.098719
ENSMUSG00000041263//Rusc1	-0.503538659	4.7213166	-5.527287121	0.000173738	0.011391	0.535427
ENSMUSG00000060961//Slc4a4	-0.503185294	4.178927504	-4.840672689	0.000506927	0.018123	-0.48209
ENSMUSG00000034656//Cacna1a	-0.50213577	6.05829397	-5.115467074	0.0003276	0.014912	-0.29958
ENSMUSG00000032177//Pde4a	-0.500576596	5.407078376	-6.088987049	7.60E-05	0.008416	1.304147
ENSMUSG00000059361//Nrsn2	-0.500129067	4.298046293	-6.208611467	6.41E-05	0.007692	1.653892
ENSMUSG00000025576//Rbfox3	-0.498674147	4.58486174	-4.518331353	0.000857388	0.02355	-1.10591
ENSMUSG00000000632//Sez6	-0.497458929	6.176806835	-5.789805464	0.000117421	0.010068	0.769855
ENSMUSG00000023036//Pcdhgc4	-0.492606962	3.998942674	-4.341585874	0.001150562	0.026894	-1.29436
ENSMUSG00000026114//Cnga3	-0.492213615	3.493428771	-5.128934947	0.000320753	0.014912	0.134428
ENSMUSG00000000627//Sema4f	-0.491962743	2.696822566	-3.67828524	0.003590201	0.046374	-2.16117
ENSMUSG00000058441//Panx2	-0.490933457	5.333661531	-5.069670358	0.000352063	0.015293	-0.29651
ENSMUSG00000024899//Papss2	-0.490921316	2.840190045	-4.283727109	0.001267971	0.02832	-1.13754
ENSMUSG00000061718//Ppp1r1b	-0.4894799	2.443421186	-3.882452881	0.002515919	0.038822	-1.74359
ENSMUSG00000038872//Zfhx3	-0.489455536	6.031086966	-5.9053886	9.91E-05	0.009463	0.959877
ENSMUSG00000037594//BC022687	-0.488289999	4.174680098	-4.435354769	0.000983832	0.025325	-1.16883
ENSMUSG00000063296//Tmem117	-0.487355383	2.310616742	-4.03092056	0.001948168	0.03504	-1.45558
ENSMUSG00000026463//Atp2b4	-0.485820752	5.7048483	-4.228429665	0.001391918	0.029747	-1.7744
ENSMUSG00000020154//Ptprb	-0.484850396	4.447632897	-4.408159535	0.001029409	0.025794	-1.26962
ENSMUSG00000026787//Gad2	-0.484826562	6.17425027	-5.53179943	0.000172558	0.011391	0.36465
ENSMUSG00000049556//Lingo1	-0.484049889	5.053881184	-5.467362108	0.000190251	0.01186	0.387721
ENSMUSG00000057716//Tmem178b	-0.482377933	5.784495738	-7.383074183	1.33E-05	0.003237	3.10424
ENSMUSG00000030376//Slc8a2	-0.482295223	3.861690211	-4.980421218	0.000405457	0.016383	-0.18585
ENSMUSG00000049336//Tenm2	-0.478929903	5.429420666	-4.359212204	0.001117099	0.026544	-1.51311
ENSMUSG00000035547//Capn5	-0.47797631	4.325248185	-4.941083421	0.000431647	0.016854	-0.3434
ENSMUSG00000028664//Ephb2	-0.477537254	5.105158299	-4.761603697	0.000575909	0.019544	-0.77856
ENSMUSG00000027070//Lrp2	-0.477524499	5.020294591	-6.232007844	6.20E-05	0.007592	1.570237

ENSMUSG00000003469//Phyhip	-0.475231442	4.42002755	-4.467559218	0.00093258	0.024626	-1.16201
ENSMUSG00000053024//Cntn2	-0.474218117	3.514388846	-4.107182229	0.001710035	0.032794	-1.59566
ENSMUSG00000013921//Clip3	-0.47289835	8.183919739	-6.937993984	2.36E-05	0.004431	2.388895
ENSMUSG00000045333//Zfp423	-0.47266027	3.97628982	-5.589560052	0.00015818	0.011165	0.770425
ENSMUSG00000026452//Syt2	-0.472198516	4.61241725	-3.827626499	0.002766819	0.040637	-2.32042
ENSMUSG00000030519//Apba2	-0.471910326	5.429379751	-6.251675399	6.03E-05	0.007586	1.545076
ENSMUSG00000035168//Tanc1	-0.471420239	5.700292469	-5.804776867	0.00011486	0.009972	0.835883
ENSMUSG00000029287//Tgfbr3	-0.471133869	3.54758656	-3.796275531	0.002921797	0.041852	-2.15096
ENSMUSG00000030223//Ptpro	-0.470378546	4.87450636	-4.529515797	0.000841696	0.02325	-1.13726
ENSMUSG00000030329//Pianp	-0.470317498	5.041428409	-4.479007554	0.000915042	0.024286	-1.25075
ENSMUSG00000030209//Grin2b	-0.468264785	5.451991788	-6.069410771	7.82E-05	0.008438	1.269058
ENSMUSG00000026437//Cdk18	-0.467867921	3.616922343	-5.208113854	0.000283442	0.013912	0.236685
ENSMUSG00000041895//Wipi1	-0.467381212	5.799516448	-6.38455367	5.01E-05	0.006744	1.701095
ENSMUSG00000030683//Sez6l2	-0.466654541	6.205862637	-6.175693235	6.72E-05	0.007729	1.356899
ENSMUSG00000102918//Pcdhgc3	-0.464481243	3.972781738	-5.257129788	0.000262671	0.01343	0.242839
ENSMUSG00000043460//Elfn2	-0.462805747	5.884888592	-5.263481232	0.0002601	0.013429	-0.04206
ENSMUSG00000007817//Zmiz1	-0.462430261	7.425275998	-5.178049172	0.000297038	0.014296	-0.26457
ENSMUSG00000056679//Gpr173	-0.461690999	3.002310379	-3.961298509	0.002195718	0.036368	-1.73392
ENSMUSG00000058297//Spock2	-0.461394823	8.461372666	-5.528759263	0.000173352	0.011391	0.276788
ENSMUSG00000062591//Tubb4a	-0.461297311	7.060012866	-4.848433018	0.000500643	0.018122	-0.79917
ENSMUSG00000015709//Arnt2	-0.460495002	4.458745064	-5.334471952	0.000233109	0.013065	0.274069
ENSMUSG00000041592//Sdk2	-0.459610779	5.533807801	-7.157224197	1.77E-05	0.00376	2.824251
ENSMUSG00000042632//Pla2g6	-0.459319465	4.768281736	-7.126263534	1.85E-05	0.003806	2.881851
ENSMUSG00000030302//Atp2b2	-0.457156375	7.152383731	-5.747266752	0.000125035	0.010078	0.655671
ENSMUSG00000056602//Fry	-0.456743282	6.580297932	-4.730115789	0.000606072	0.019873	-0.9786
ENSMUSG00000039202//Abhd2	-0.456189957	5.618500791	-6.006012761	8.57E-05	0.008703	1.153706
ENSMUSG00000022054//Nefm	-0.45472501	7.003982872	-4.432083665	0.0009892	0.02535	-1.50812
ENSMUSG00000047875//Gpr157	-0.454223232	2.56683182	-3.729074844	0.003284981	0.044439	-2.04109
ENSMUSG00000033595//Lgi3	-0.453755427	3.927690949	-3.806261257	0.002871483	0.041577	-2.21887
ENSMUSG00000038020//Rapgefl1	-0.452737295	4.305310666	-5.662128139	0.000141897	0.010714	0.82124
ENSMUSG00000048078//Tenm4	-0.451447485	6.717903185	-4.738984411	0.000597412	0.019842	-0.97021
ENSMUSG00000032356//Rasgrf1	-0.450871121	6.877345106	-5.238483774	0.000270376	0.013607	-0.146
ENSMUSG00000032875//Arhgef17	-0.450655179	6.819526402	-6.075520996	7.75E-05	0.008416	1.172902
ENSMUSG00000034040//Wbscr17	-0.447744153	5.167505462	-4.182346755	0.00150486	0.03098	-1.78572
ENSMUSG00000032482//Cspg5	-0.447226303	6.454944263	-5.282389861	0.000252602	0.013422	-0.05451
ENSMUSG00000020277//Pfkl	-0.446294667	7.911344322	-4.273507566	0.001289978	0.028588	-1.81383
ENSMUSG00000027577//Chrna4	-0.446256565	4.605016316	-5.080501663	0.000346108	0.015265	-0.16522
ENSMUSG00000019146//Cacng2	-0.446011329	4.801997352	-5.266227468	0.000258996	0.013429	0.103973
ENSMUSG00000026853//Crat	-0.445941141	4.488967104	-5.808428399	0.000114244	0.009972	1.014791
ENSMUSG00000066392//Nrxn3	-0.445650188	7.542242026	-4.647995586	0.000692827	0.021236	-1.15567
ENSMUSG00000086040//Wipf3	-0.445496838	3.525801392	-3.75865909	0.003119665	0.043329	-2.2127
ENSMUSG00000033039//Micall1	-0.44526443	4.353177256	-5.825600563	0.000111396	0.009972	1.065586
ENSMUSG00000029673//Auts2	-0.444941018	5.926606506	-5.754790513	0.000123651	0.010078	0.735984
ENSMUSG00000053141//Ptprt	-0.444938854	5.583700111	-5.686274229	0.000136882	0.01064	0.664119
ENSMUSG00000025475//Adgra1	-0.443841896	5.27878782	-5.228045144	0.000274794	0.013681	-0.02972
ENSMUSG00000005986//Ankrd13d	-0.443512481	3.429879444	-3.967185901	0.002173573	0.036261	-1.8218
ENSMUSG00000022421//Nptxr	-0.443128311	5.75044639	-5.75326511	0.00012393	0.010078	0.750652
ENSMUSG00000028833//Ncdn	-0.442581929	6.803519187	-5.240587968	0.000269495	0.013607	-0.13951
ENSMUSG00000029408//Abcb9	-0.442195761	3.929546206	-5.066583989	0.00035378	0.015329	-0.05816
ENSMUSG00000040125//Gpr26	-0.440900759	5.492153734	-5.074897804	0.000349176	0.015282	-0.30811
ENSMUSG00000030806//Stx1b	-0.440598243	7.444982203	-5.58951515	0.000158191	0.011165	0.397736
ENSMUSG00000068566//Myadm	-0.4395991	3.470494843	-3.869555881	0.002572737	0.03933	-2.00333
ENSMUSG00000025189//Cnnm1	-0.439419744	5.541716654	-5.49280114	0.000183046	0.011736	0.363477
ENSMUSG00000045009//Prrt3	-0.438692302	2.704703488	-4.04569803	0.001899465	0.034663	-1.51845
ENSMUSG00000056222//Spock1	-0.438391681	5.895693399	-7.118654436	1.87E-05	0.003806	2.735577
ENSMUSG00000028373//Astn2	-0.438388083	3.862927921	-3.99095021	0.002086515	0.036037	-1.87774
ENSMUSG00000028532//Cachd1	-0.436975746	3.87802827	-4.026812013	0.001961939	0.035143	-1.8179
ENSMUSG00000036862//Dchs1	-0.435783686	3.27391986	-4.39290698	0.001055934	0.026098	-1.04677
ENSMUSG00000006800//Sulf2	-0.434235076	5.655282079	-5.228304413	0.000274683	0.013681	-0.0757
ENSMUSG00000052949//Rnf157	-0.434010958	7.931316052	-7.419594838	1.27E-05	0.003193	3.05372
ENSMUSG00000027894//Slc6a17	-0.433858456	6.921241758	-5.81354031	0.000113388	0.009972	0.767253

ENSMUSG00000027520//Zdbf2	-0.433222775	4.415464992	-4.188393966	0.00148951	0.030787	-1.64543
ENSMUSG00000002341//Ncan	-0.431443187	2.907081842	-3.658707616	0.003715531	0.047277	-2.24528
ENSMUSG00000038257//Glra3	-0.429257586	4.044328247	-4.486549098	0.000903678	0.024286	-1.05432
ENSMUSG00000035713//Usp35	-0.427626584	2.863919714	-3.897328603	0.002451995	0.038141	-1.81421
ENSMUSG00000053166//Cdh22	-0.426324866	3.471704767	-4.422325737	0.001005398	0.025431	-1.04045
ENSMUSG00000037138//Aff3	-0.426213798	4.900655466	-4.760180179	0.000577238	0.019551	-0.7491
ENSMUSG00000046204//Pnma2	-0.425880663	3.373010165	-4.327551439	0.001177956	0.027037	-1.18128
ENSMUSG00000002489//Tiam1	-0.42467288	4.96068646	-4.366794137	0.00110302	0.026536	-1.43209
ENSMUSG00000015501//Hivep2	-0.424440919	5.784719898	-4.299200397	0.001235395	0.027986	-1.65933
ENSMUSG00000040136//Abcc8	-0.423662632	3.537492073	-3.973981343	0.0021483	0.036261	-1.83459
ENSMUSG00000050628//Ubald2	-0.422141929	3.730651565	-4.009499198	0.002021085	0.035579	-1.81579
ENSMUSG00000008153//Clstn3	-0.421145983	6.315182223	-6.69363802	3.28E-05	0.005474	2.108302
ENSMUSG00000024736//Tmem132a	-0.420877117	4.449338657	-5.854550035	0.000106764	0.009821	1.092692
ENSMUSG00000029101//Rgs12	-0.419943866	3.23915331	-3.98183041	0.002119488	0.036122	-1.75226
ENSMUSG00000031654//Cbln1	-0.419626022	3.023091359	-3.61437568	0.00401627	0.049386	-2.3514
ENSMUSG00000027784//Ppm1l	-0.418884734	6.720436407	-6.081251727	7.69E-05	0.008416	1.185679
ENSMUSG00000029712//Actl6b	-0.418863805	4.066734326	-5.498654982	0.00018143	0.011719	0.609725
ENSMUSG00000000628//Hk2	-0.418679511	8.62988931	-5.624008569	0.000150216	0.01083	0.425037
ENSMUSG00000031837//Necab2	-0.418326123	5.183882084	-4.034452285	0.001936412	0.034974	-2.04863
ENSMUSG00000035226//Rims4	-0.416692598	4.533738498	-4.668453384	0.000670059	0.021015	-0.84034
ENSMUSG00000035735//Dagla	-0.416225152	4.616065672	-4.318417159	0.00119615	0.027346	-1.45672
ENSMUSG00000030731//Syt3	-0.415373254	3.689757483	-4.078896611	0.001794614	0.033601	-1.68477
ENSMUSG00000051375//Pcdh1	-0.414882405	5.548887211	-4.391747016	0.00105798	0.026111	-1.47155
ENSMUSG00000068740//Celsr2	-0.413326239	6.189575537	-5.324103012	0.000236859	0.013065	0.030487
ENSMUSG00000004508//Gab2	-0.412754511	4.206458158	-5.003928308	0.000390612	0.016045	-0.21646
ENSMUSG00000041000//Trim62	-0.411447771	3.360483588	-4.364837009	0.001106636	0.026536	-1.11434
ENSMUSG00000048814//Lonrf2	-0.410590497	6.76429723	-5.15416383	0.00030833	0.014558	-0.27924
ENSMUSG00000063430//Wscd2	-0.410412632	4.356180347	-4.639499421	0.00070252	0.021274	-0.85576
ENSMUSG00000036766//Dner	-0.410020882	6.027955682	-4.636449567	0.000706034	0.021274	-1.10036
ENSMUSG00000023991//Foxp4	-0.409597159	5.309576221	-5.208282859	0.000283368	0.013912	-0.06605
ENSMUSG00000022099//Dmtn	-0.409259439	5.761666818	-7.176476319	1.73E-05	0.00376	2.827057
ENSMUSG00000029608//Rph3a	-0.408799778	7.563894521	-5.390319209	0.000213967	0.012632	0.075906
ENSMUSG00000028351//Brinp1	-0.408535064	4.495985356	-5.246554748	0.000267012	0.013565	0.125476
ENSMUSG00000028782//Adgrb2	-0.407304453	5.331720959	-6.081650265	7.68E-05	0.008416	1.302461
ENSMUSG00000025582//Nptx1	-0.406417362	6.445398725	-4.636020448	0.00070653	0.021274	-1.13132
ENSMUSG00000035566//Pcdh17	-0.406344928	5.160863976	-4.661306759	0.000677922	0.021071	-0.9567
ENSMUSG00000035936//Aldh5a1	-0.405597259	5.062271883	-5.261098534	0.000261061	0.013429	0.054848
ENSMUSG00000025153//Fasn	-0.405072386	7.181578934	-4.680082931	0.000657468	0.020808	-1.08861
ENSMUSG00000031790//Mmp15	-0.40464602	3.53823905	-4.833960144	0.00051243	0.018208	-0.35987
ENSMUSG00000060519//Tor3a	-0.404159689	3.357819723	-4.247160657	0.001348574	0.029253	-1.31671
ENSMUSG00000003316//Glg1	-0.40401917	7.602530704	-5.11333336	0.000328699	0.014912	-0.37656
ENSMUSG00000024617//Camk2a	-0.402530053	5.722274713	-6.19339412	6.55E-05	0.007692	1.425413
ENSMUSG00000040724//Kcna2	-0.401731843	5.46600213	-4.339897604	0.001153822	0.02691	-1.55144
ENSMUSG00000003410//Elavl3	-0.401635811	6.58338488	-6.442169954	4.62E-05	0.006333	1.729464
ENSMUSG00000010066//Cacna2d2	-0.400580639	6.564652593	-5.624282827	0.000150154	0.01083	0.48674
ENSMUSG00000058571//Gpc6	-0.40044595	5.079306478	-4.962391323	0.000417246	0.016538	-0.43825
ENSMUSG00000031492//Chrnb3	-0.400109526	6.354723316	-4.704906369	0.000631419	0.020285	-1.00843
ENSMUSG00000029516//Cit	-0.399845969	4.783394035	-5.35631477	0.000225415	0.012926	0.252336
ENSMUSG00000028661//Epha8	-0.399439877	5.136808625	-6.599138968	3.73E-05	0.006027	2.09053
ENSMUSG00000026959//Grin1	-0.399055455	5.832658572	-4.603600784	0.000745078	0.021721	-1.13827
ENSMUSG00000015484//Fam163a	-0.398891976	4.538100815	-5.576930175	0.000161212	0.011203	0.645454
ENSMUSG00000042978//Sbk1	-0.398426856	6.335858638	-5.664597081	0.000141375	0.010714	0.563288
ENSMUSG00000029869//Ephb6	-0.397765761	3.956537872	-4.395459299	0.001051446	0.026024	-1.19241
ENSMUSG00000029581//Fscn1	-0.397329856	5.788550774	-4.335668563	0.00116203	0.026933	-1.59617
ENSMUSG00000059857//Ntng1	-0.397184752	5.736230124	-4.302593064	0.001228369	0.027899	-1.64817
ENSMUSG00000020340//Cyfip2	-0.396807326	8.502855268	-6.038417003	8.17E-05	0.008454	1.0695
ENSMUSG00000032387//Rbpms2	-0.396363058	4.503100555	-4.088612914	0.00176508	0.033371	-1.83734
ENSMUSG00000030310//Slc6a1	-0.396352567	8.241185568	-4.886993334	0.000470611	0.017707	-0.76878
ENSMUSG00000034402//Kcnh5	-0.396305762	4.657824539	-4.510272675	0.000868885	0.0237	-1.13288
ENSMUSG00000062991//Nrg1	-0.395244433	2.822779227	-3.691392286	0.003508736	0.046011	-2.16764
ENSMUSG00000073805//Fam196a	-0.392133951	4.949243705	-4.657376222	0.000682288	0.021116	-0.93113

ENSMUSG00000011752//Pgam1	-0.392051203	7.45029138	-4.528045684	0.000843741	0.02327	-1.35837
ENSMUSG00000022199//Slc22a17	-0.391370156	6.546466533	-6.03197207	8.25E-05	0.008483	1.119065
ENSMUSG00000036913//Trim67	-0.391245545	5.528674518	-5.190383636	0.000291378	0.014137	-0.12287
ENSMUSG00000042724//Map3k9	-0.390909388	5.275427772	-4.541931098	0.000824629	0.022925	-1.17703
ENSMUSG00000013367//Iglon5	-0.390774271	5.70482383	-6.045552674	8.09E-05	0.008438	1.204531
ENSMUSG00000033214//Slitrk5	-0.39028354	3.397060752	-4.228839585	0.001390955	0.029747	-1.35727
ENSMUSG00000074657//Kif5a	-0.389812833	7.967966236	-4.73795606	0.00059841	0.019842	-1.01434
ENSMUSG00000026458//Ppfia4	-0.389467045	4.973518961	-5.32992765	0.000234745	0.013065	0.179533
ENSMUSG00000048349//Pou4f1	-0.389346542	4.919581217	-4.856209682	0.000494427	0.018122	-0.59074
ENSMUSG00000017314//Mpp2	-0.388554056	6.33934749	-4.849312545	0.000499935	0.018122	-0.76331
ENSMUSG00000039252//Lgi2	-0.388308091	3.253112429	-3.926125793	0.002332989	0.037257	-1.85336
ENSMUSG00000041577//Prelp	-0.38791071	5.45155076	-3.950738673	0.002236027	0.036488	-2.23407
ENSMUSG00000047013//Fbxo41	-0.386788353	4.1153378	-3.621060069	0.003969366	0.048948	-2.58972
ENSMUSG00000026975//Dph7	-0.386685795	4.332279643	-5.121401211	0.000324564	0.014912	-0.04771
ENSMUSG00000034731//Dgkh	-0.386369027	4.383200642	-3.984071904	0.002111334	0.036122	-1.99845
ENSMUSG00000044628//Rnf208	-0.386205414	4.179558099	-3.604181141	0.004088904	0.049857	-2.63348
ENSMUSG00000020828//Pld2	-0.385618494	3.164528939	-3.686996683	0.003535842	0.046157	-2.25565
ENSMUSG00000060534//Dcc	-0.384950973	4.266503545	-4.080330356	0.001790224	0.033601	-1.80535
ENSMUSG00000033209//Ttc28	-0.384760934	6.44136027	-4.714988143	0.000621151	0.020098	-0.99658
ENSMUSG00000037259//Dzank1	-0.384715902	6.832750545	-4.819779544	0.000524264	0.018402	-0.83853
ENSMUSG00000031778//Cx3cl1	-0.38407437	6.026792556	-4.096655186	0.001741017	0.033104	-2.03825
ENSMUSG00000030350//Prmt8	-0.383469373	4.28874537	-4.523076816	0.000850693	0.023424	-1.04109
ENSMUSG00000021130//Galnt16	-0.382872872	3.805583458	-4.215152247	0.001423522	0.030106	-1.47228
ENSMUSG00000033768//Nrxn2	-0.382631175	7.039595351	-5.639177927	0.000146845	0.010766	0.490205
ENSMUSG00000062044//Lmtk3	-0.381958516	5.8468776	-4.663086643	0.000675955	0.021071	-1.03812
ENSMUSG00000047976//Kcna1	-0.380511909	5.509779228	-5.645156081	0.000145539	0.010758	0.608191
ENSMUSG00000029174//Tbc1d1	-0.379765979	3.684932064	-3.7312324	0.003272625	0.044423	-2.29808
ENSMUSG00000021136//Smoc1	-0.379687873	3.707935106	-3.9665191	0.002176069	0.036261	-1.88643
ENSMUSG00000020042//Btbd11	-0.379396821	4.22605904	-4.686669041	0.000650449	0.020661	-0.75039
ENSMUSG00000038665//Dgki	-0.378662972	4.623377997	-4.643550302	0.000697881	0.021236	-0.89908
ENSMUSG00000070476//Fam217b	-0.377868323	5.019784977	-5.453498972	0.000194303	0.011987	0.37073
ENSMUSG00000028982//Slc25a33	-0.377503083	5.342755524	-4.055131097	0.00186904	0.034438	-2.03462
ENSMUSG00000061576//Dpp6	-0.377302201	6.741491494	-4.56798035	0.00078999	0.022502	-1.26301
ENSMUSG00000027864//Ptgfrn	-0.376292338	4.180287621	-4.663614045	0.000675373	0.021071	-0.78024
ENSMUSG00000026475//Rgs16	-0.3759739	4.432568983	-4.003019175	0.002043697	0.035796	-1.97472
ENSMUSG00000051228//Nyx	-0.375968734	4.394659846	-4.810558623	0.000532113	0.018528	-0.57452
ENSMUSG00000064125//Prr36	-0.375950261	3.379589414	-3.68186192	0.003567779	0.046303	-2.31515
ENSMUSG00000058975//Kcnc1	-0.375228082	7.084439018	-5.058594085	0.000358265	0.015408	-0.44938
ENSMUSG00000008658//Rbfox1	-0.373051106	6.182127068	-5.1293638	0.000320537	0.014912	-0.28658
ENSMUSG00000030084//Plxna1	-0.372884254	5.153038662	-4.437756848	0.000979909	0.025325	-1.33883
ENSMUSG00000048483//Zdhhc22	-0.37155369	3.599859464	-4.373209156	0.001091252	0.026536	-1.1533
ENSMUSG00000029822//Osbpl3	-0.371236363	4.022657604	-4.015033404	0.002001979	0.035386	-1.86974
ENSMUSG00000039262//Prrc2b	-0.370895213	8.699416642	-5.720690974	0.000130057	0.010336	0.575971
ENSMUSG00000027200//Sema6d	-0.369595363	3.853028329	-3.632050039	0.003893474	0.048487	-2.51295
ENSMUSG00000047496//Rnf152	-0.368361803	6.223005559	-5.420525895	0.000204315	0.012241	0.183456
ENSMUSG00000024743//Syt7	-0.368218164	6.78538879	-5.090153541	0.000340891	0.015219	-0.3855
ENSMUSG00000049422//Chchd10	-0.368000826	5.381621429	-4.566345954	0.000792118	0.022525	-1.14939
ENSMUSG00000020866//Cacna1g	-0.367876249	6.340438989	-4.736804586	0.000599529	0.019842	-0.95334
ENSMUSG00000028542//Slc6a9	-0.367551302	6.446236472	-4.432976952	0.000987731	0.02535	-1.48061
ENSMUSG00000040209//Zfp704	-0.367371721	5.970422893	-5.038396553	0.000369874	0.015688	-0.41929
ENSMUSG00000024112//Cacna1h	-0.367217841	6.752552815	-6.025773885	8.32E-05	0.008508	1.100131
ENSMUSG00000025810//Nrp1	-0.366493088	3.795546374	-3.787528741	0.002966618	0.042078	-2.22287
ENSMUSG00000031586//Rbpms	-0.365989685	5.090058353	-4.063633256	0.001842052	0.034012	-1.98303
ENSMUSG00000030787//Lyve1	-0.365331124	3.558389774	-4.14631947	0.001599797	0.031793	-1.53728
ENSMUSG00000038453//Srcin1	-0.364759675	5.73324021	-3.773369542	0.003040686	0.042744	-2.58407
ENSMUSG00000022463//Srebf2	-0.364308896	6.960550438	-5.134662674	0.000317887	0.014912	-0.31923
ENSMUSG00000009418//Nav1	-0.363685104	7.255401345	-5.565922059	0.000163904	0.011209	0.36673
ENSMUSG00000035891//Cerk	-0.363448796	7.43880535	-6.931447838	2.38E-05	0.004431	2.398439
ENSMUSG00000048385//Scrt1	-0.363013971	6.28232596	-4.762512428	0.000575062	0.019544	-0.90579
ENSMUSG00000032549//Rab6b	-0.362871426	8.212675179	-5.386556529	0.000215203	0.012632	0.053621
ENSMUSG00000025927//Tfap2b	-0.362204098	7.690243093	-4.487337674	0.000902498	0.024286	-1.43606

ENSMUSG00000051177//Plcb1	-0.362004254	4.743263863	-3.740086327	0.003222417	0.044115	-2.50062
ENSMUSG00000068735//Trp53i11	-0.361688067	3.33525354	-3.763073228	0.003095746	0.043147	-2.16054
ENSMUSG00000057777//Mab21l2	-0.361182376	4.939088543	-5.550284897	0.000167811	0.011214	0.536602
ENSMUSG00000040373//Cacng5	-0.361169989	4.845616921	-3.965797616	0.002178774	0.036261	-2.11645
ENSMUSG00000053965//Pde5a	-0.360889486	3.72966312	-4.101928322	0.001725426	0.03298	-1.65332
ENSMUSG00000020181//Nav3	-0.360481072	4.235254269	-3.830632067	0.002752415	0.04047	-2.24078
ENSMUSG00000069072//Slc7a14	-0.360404897	5.922398651	-4.73106485	0.000605139	0.019873	-0.92982
ENSMUSG00000047085//Lrrc4b	-0.360336812	5.048943929	-5.165154802	0.000303079	0.014467	-0.09937
ENSMUSG00000063626//Unc5d	-0.360067299	5.067161262	-5.296191678	0.000247274	0.013254	0.110954
ENSMUSG00000040907//Atp1a3	-0.359782172	10.92115251	-5.018293175	0.000381824	0.015909	-0.5909
ENSMUSG00000041272//Tox	-0.359335428	4.913748442	-4.806674424	0.000535456	0.018562	-0.67293
ENSMUSG00000039474//Wfs1	-0.358564326	5.567083975	-4.623996657	0.000720576	0.021402	-1.07404
ENSMUSG00000063160//Numbl	-0.358537557	4.68296804	-4.030759734	0.001948706	0.03504	-1.9729
ENSMUSG00000006731//B4galnt1	-0.35827031	4.02597108	-4.849954688	0.00049942	0.018122	-0.43592
ENSMUSG00000031226//Pbdc1	-0.358140168	4.122591807	-4.561107254	0.000798979	0.022612	-0.94282
ENSMUSG00000026587//Astn1	-0.35804277	6.046438504	-4.560042026	0.000800381	0.022612	-1.23271
ENSMUSG00000055322//Tns1	-0.357777202	5.366600606	-4.535173125	0.000833874	0.023108	-1.20089
ENSMUSG00000037605//Adgrl3	-0.357503342	5.870637901	-4.663701325	0.000675277	0.021071	-1.03938
ENSMUSG00000052512//Nav2	-0.357178224	5.723002061	-3.848892406	0.002666558	0.039824	-2.44795
ENSMUSG00000061911//Myt1l	-0.357110224	6.173102872	-4.361340216	0.001113129	0.026544	-1.5864
ENSMUSG00000032558//Nphp3	-0.356948447	4.354219292	-3.788160929	0.002963355	0.042066	-2.34049
ENSMUSG00000042826//Fgf11	-0.35668518	6.119275635	-5.027155768	0.000376506	0.015864	-0.45041
ENSMUSG00000025969//Nrp2	-0.356380367	4.792188478	-4.223132054	0.001404439	0.029909	-1.65421
ENSMUSG00000041540//Sox5	-0.356269565	3.936801082	-4.176876235	0.001518889	0.031061	-1.56747
ENSMUSG00000050822//Slc29a4	-0.355166708	4.337346802	-3.852578064	0.002649567	0.039823	-2.22239
ENSMUSG00000079481//Nhsl2	-0.354797499	4.379220356	-4.606882769	0.000741077	0.021664	-0.9157
ENSMUSG00000034730//Adgrb1	-0.354604757	6.525843472	-5.780001445	0.000119131	0.010068	0.732587
ENSMUSG00000030172//Erc1	-0.353887036	4.864927584	-4.051012469	0.001882261	0.034562	-1.96899
ENSMUSG00000025089//Gfra1	-0.353648165	5.104836371	-4.156154565	0.001573276	0.031493	-1.82236
ENSMUSG00000025207//Sema4g	-0.353161061	5.591550206	-5.162462078	0.000304356	0.014468	-0.17595
ENSMUSG00000063646//Jakmip1	-0.352234946	3.770217945	-3.798678588	0.002909606	0.041789	-2.1974
ENSMUSG00000029651//Mtus2	-0.352033828	6.344486074	-4.240095163	0.001364756	0.029395	-1.81035
ENSMUSG00000029108//Pcdh7	-0.351030184	6.397001708	-4.552013416	0.000811037	0.022656	-1.27232
ENSMUSG00000071234//Syndig1l	-0.350683465	3.493499306	-3.860785515	0.002612133	0.0395	-2.02417
ENSMUSG00000034891//Sncb	-0.350212021	8.457214857	-5.122921228	0.000323791	0.014912	-0.38049
ENSMUSG00000020889//Nr1d1	-0.349041317	7.497441584	-5.260684463	0.000261229	0.013429	-0.1319
ENSMUSG00000042846//Lrrtm3	-0.348875547	3.205244467	-3.651756604	0.003761107	0.047492	-2.32781
ENSMUSG00000032735//Ablim3	-0.348782183	4.319642163	-4.050659399	0.001883399	0.034562	-1.8682
ENSMUSG00000022946//Dopey2	-0.348250214	4.869249836	-4.843102568	0.00050495	0.018123	-0.60446
ENSMUSG00000072825//Cep170b	-0.348009546	6.808017231	-4.511646212	0.000866914	0.023683	-1.36277
ENSMUSG00000039372//March4	-0.346004742	4.474989879	-3.714444453	0.003370053	0.045146	-2.49605
ENSMUSG00000049532//Sall2	-0.34566726	6.111477869	-5.120763028	0.000324889	0.014912	-0.2952
ENSMUSG00000034926//Dhcr24	-0.345659952	6.045963625	-4.593266674	0.000757825	0.021954	-1.17571
ENSMUSG00000000617//Grm6	-0.345437494	6.805894184	-5.29313383	0.000248444	0.013254	-0.05415
ENSMUSG00000000093//Tbx2	-0.345256667	5.811367216	-5.428938122	0.00020171	0.012241	0.232027
ENSMUSG00000055013//Agap1	-0.34516366	8.140138835	-4.159754586	0.001563684	0.031373	-2.01888
ENSMUSG00000020262//Adarb1	-0.345077817	7.955762031	-5.75280637	0.000124014	0.010078	0.640081
ENSMUSG00000037771//Slc32a1	-0.344927973	5.009793654	-3.660916275	0.003701169	0.047216	-2.68756
ENSMUSG00000003279//Dlgap1	-0.344351407	6.425304472	-4.371760796	0.001093898	0.026536	-1.58557
ENSMUSG00000023032//Slc4a8	-0.343745492	7.271826441	-4.72882409	0.000607345	0.019873	-1.00895
ENSMUSG00000017631//Abr	-0.342498042	6.620304817	-5.423612462	0.000203355	0.012241	0.164756
ENSMUSG00000039741//Bahcc1	-0.342095741	5.409295311	-3.921986215	0.00234972	0.037387	-2.27959
ENSMUSG00000069227//Gprin1	-0.341986788	5.210253495	-4.911205802	0.000452729	0.017402	-0.54252
ENSMUSG00000027895//Kcnc4	-0.341970844	4.214946704	-3.645345065	0.003803656	0.047838	-2.56721
ENSMUSG00000053930//Shisa6	-0.341696623	3.723846854	-3.937086886	0.00228928	0.037057	-1.94191
ENSMUSG00000053414//Hunk	-0.341562307	5.606659217	-4.273592488	0.001289794	0.028588	-1.68419
ENSMUSG00000038738//Shank1	-0.340461614	6.85229967	-5.113839526	0.000328438	0.014912	-0.34926
ENSMUSG00000048142//Nat8l	-0.340126023	5.443411745	-4.800006714	0.000541247	0.018585	-0.76035
ENSMUSG00000006576//Slc4a3	-0.339914737	6.516005726	-5.273258174	0.000256194	0.013429	-0.07284
ENSMUSG00000036155//Mgat5	-0.339607436	6.765607474	-5.085553131	0.000343367	0.015219	-0.39225
ENSMUSG00000031709//Tbc1d9	-0.339277759	5.911402704	-4.021910715	0.001978498	0.035213	-2.15975

ENSMUSG00000042401//Crtac1	-0.339211401	3.864247977	-4.19917628	0.001462544	0.03047	-1.51297
ENSMUSG00000048899//Rimkla	-0.339187203	5.565462898	-5.933042184	9.52E-05	0.009264	1.048352
ENSMUSG00000047787//Flrt1	-0.338457005	4.282499978	-3.816611896	0.002820275	0.041093	-2.27533
ENSMUSG00000034612//Chst11	-0.338211807	3.700362371	-3.945241612	0.002257313	0.036655	-1.92224
ENSMUSG00000033361//Prrg3	-0.337625491	4.439577645	-4.517918906	0.000857973	0.02355	-1.0793
ENSMUSG00000026923//Notch1	-0.336966364	4.705767643	-4.995879229	0.000395629	0.016174	-0.32242
ENSMUSG00000029875//Ccdc184	-0.336542495	3.219664853	-3.61756067	0.00399385	0.049145	-2.39202
ENSMUSG00000028214//Gem	-0.336507679	4.789326787	-4.644730805	0.000696535	0.021236	-0.92623
ENSMUSG00000039385//Cdh6	-0.336081205	3.162147605	-3.67636143	0.003602321	0.046432	-2.27397
ENSMUSG00000023011//Faim2	-0.336020984	7.247153894	-5.680503249	0.000138063	0.010662	0.54783
ENSMUSG00000028467//Gba2	-0.335985536	5.859503981	-5.574683636	0.000161757	0.011203	0.459432
ENSMUSG00000024186//Rgs11	-0.335789752	5.268563454	-3.831914987	0.00274629	0.04047	-2.42084
ENSMUSG00000050272//Dscam	-0.335013846	5.681132183	-4.848576073	0.000500527	0.018122	-0.70686
ENSMUSG00000062960//Kdr	-0.334695882	7.347717253	-4.216280068	0.001420809	0.030106	-1.89708
ENSMUSG00000023169//Slc38a1	-0.334454264	8.100896947	-4.181478089	0.001507079	0.030989	-1.97979
ENSMUSG00000034771//Tle2	-0.334083276	4.50698888	-4.263415713	0.001312102	0.028749	-1.53195
ENSMUSG00000034818//Celf5	-0.333996167	4.757485406	-4.302659905	0.001228231	0.027899	-1.50949
ENSMUSG00000026764//Kif5c	-0.333674815	8.301693885	-6.051922543	8.02E-05	0.008438	1.093536
ENSMUSG00000036882//Arhgap33	-0.333134325	4.88186408	-3.927063311	0.002329217	0.037257	-2.19136
ENSMUSG00000034390//Cmip	-0.332842602	6.355246688	-4.659589792	0.000679825	0.021093	-1.0856
ENSMUSG00000009394//Syn2	-0.332726126	4.928007646	-3.977523434	0.002135248	0.03616	-2.10961
ENSMUSG00000043259//Fam13c	-0.332277115	4.502025982	-4.688112514	0.000648921	0.02065	-0.80107
ENSMUSG00000020747//Tmem94	-0.332261111	5.493967206	-4.843779111	0.000504401	0.018123	-0.69304
ENSMUSG00000020524//Gria1	-0.332086819	5.773164616	-4.608577421	0.00073902	0.021664	-1.12356
ENSMUSG00000041528//Rnf123	-0.331462929	7.578269493	-5.473342878	0.00018853	0.011826	0.208696
ENSMUSG00000034336//Ina	-0.331046193	6.33399139	-4.074351824	0.001808604	0.033753	-2.10139
ENSMUSG00000049176//Frmpd4	-0.330513228	4.092204511	-3.68389759	0.003555082	0.046269	-2.47246
ENSMUSG00000074785//Plxnc1	-0.330318551	5.070103207	-3.850945937	0.002657077	0.039823	-2.35728
ENSMUSG00000057147//Dph6	-0.329360931	5.136794146	-4.258526128	0.001322963	0.028876	-1.64784
ENSMUSG00000032501//Trib1	-0.328982713	5.254528515	-4.29363066	0.001247019	0.028139	-1.60364
ENSMUSG00000042751//Nmnat2	-0.328876209	5.46535492	-4.236521039	0.001373019	0.029489	-1.73176
ENSMUSG00000029674//Limk1	-0.32857802	4.948170452	-4.018518752	0.001990043	0.035283	-2.04035
ENSMUSG00000023034//Nr4a1	-0.328480402	4.852480185	-3.832076248	0.002745522	0.04047	-2.35536
ENSMUSG00000033597//Caskin1	-0.32838199	6.238375097	-5.187255299	0.000292803	0.014137	-0.19565
ENSMUSG00000018470//Kcnab3	-0.328203294	4.749550205	-3.833807216	0.002737283	0.04047	-2.33445
ENSMUSG00000018411//Mapt	-0.328079883	7.505591914	-4.371546313	0.00109429	0.026536	-1.63105
ENSMUSG00000025375//Aatk	-0.327921463	6.178310453	-4.89492923	0.000464669	0.017628	-0.67539
ENSMUSG00000028931//Kcnab2	-0.327392599	7.95820377	-4.197638914	0.001466358	0.030513	-1.94805
ENSMUSG00000056755//Grm7	-0.327342996	4.496703815	-3.966882152	0.002174709	0.036261	-2.05099
ENSMUSG00000045994//B3gat1	-0.327069903	6.422683915	-5.484000845	0.000185505	0.011749	0.272004
ENSMUSG00000036192//Rorb	-0.326962435	8.47739911	-5.583081735	0.000159727	0.011191	0.362793
ENSMUSG00000042942//Greb1l	-0.326515988	4.322638342	-3.703058649	0.003437829	0.045489	-2.48601
ENSMUSG00000013076//Amotl1	-0.326434615	5.467403412	-4.109349387	0.001703729	0.032784	-1.95542
ENSMUSG00000010476//Ebf3	-0.326387491	5.638737929	-3.606892291	0.004069457	0.049768	-2.87217
ENSMUSG00000033080//Vsx1	-0.326007527	4.551339054	-4.153012874	0.001581698	0.031547	-1.73343
ENSMUSG00000034912//Mdga2	-0.325595074	5.748389645	-3.679648061	0.00358164	0.046374	-2.75375
ENSMUSG00000019302//Atp6v0a1	-0.325544856	8.244549734	-4.859283515	0.000491993	0.018111	-0.8154
ENSMUSG00000096351//Samd11	-0.325378509	8.852582977	-4.12818716	0.00164992	0.032341	-2.08713
ENSMUSG00000034168//Irf2bpl	-0.325252263	5.63612737	-4.098199202	0.001736437	0.033082	-1.99567
ENSMUSG00000045038//Prkce	-0.325168375	6.864144453	-4.209132036	0.001438098	0.030214	-1.89151
ENSMUSG00000041014//Nrg3	-0.324194995	3.576272823	-3.865767908	0.002589677	0.039338	-2.0344
ENSMUSG00000005045//Chd5	-0.323561003	6.477088785	-4.175246036	0.001523096	0.031061	-1.93236
ENSMUSG00000020331//Hcn2	-0.322497605	4.69751314	-3.851453542	0.002654739	0.039823	-2.29357
ENSMUSG00000032536//Trak1	-0.322323629	6.915591623	-5.968940174	9.04E-05	0.00897	1.006868
ENSMUSG00000002032//Tmem25	-0.321919086	4.130685483	-3.934067264	0.002301235	0.037057	-2.03546
ENSMUSG00000041534//Rbp3	-0.321505947	12.08157922	-4.192098624	0.001480187	0.030742	-2.0204
ENSMUSG00000040797//Iqsec3	-0.321467449	8.358551164	-5.375039942	0.000219033	0.012735	0.032279
ENSMUSG00000000305//Cdh4	-0.321455771	5.480339959	-3.654496247	0.003743075	0.047368	-2.76714
ENSMUSG00000015599//Ttbk1	-0.320592508	4.833122548	-3.958339344	0.002206937	0.036381	-2.12755
ENSMUSG00000055254//Ntrk2	-0.320591498	6.550175944	-4.25246512	0.001336557	0.029065	-1.80094
ENSMUSG00000034570//Inpp5j	-0.319454895	4.17212243	-3.878891291	0.002531478	0.038856	-2.1419

ENSMUSG00000035596//Mboat7	-0.319057985	5.612822651	-4.341147184	0.001151409	0.026894	-1.56714
ENSMUSG00000039683//Sdk1	-0.319025941	4.675730077	-4.741797941	0.000594692	0.019842	-0.74192
ENSMUSG00000042804//Gpr153	-0.317998011	5.901315019	-5.192649305	0.000290351	0.014131	-0.15906
ENSMUSG00000024393//Prrc2a	-0.317940698	8.817867902	-5.338411809	0.000231701	0.013065	-0.03439
ENSMUSG00000028444//Cntfr	-0.317715749	5.485770117	-4.288831497	0.001257127	0.028293	-1.64291
ENSMUSG00000040093//Bmf	-0.317363593	3.725251435	-3.798557568	0.002910219	0.041789	-2.18756
ENSMUSG00000024952//Rps6ka4	-0.316549571	4.535890872	-4.435465554	0.000983651	0.025325	-1.23921
ENSMUSG00000052911//Lamb2	-0.316344455	4.390979465	-3.656261057	0.003731507	0.047325	-2.58359
ENSMUSG00000036565//Ttyh3	-0.316320455	6.269098485	-4.557509355	0.000803727	0.022626	-1.25433
ENSMUSG00000022840//Adcy5	-0.315806391	5.550122422	-4.803224937	0.000538444	0.018562	-0.76799
ENSMUSG00000050989//Selenon	-0.31565474	4.612589003	-4.169215063	0.001538765	0.031197	-1.71649
ENSMUSG00000034958//Atcay	-0.315481448	5.81404168	-4.757180822	0.000580049	0.01957	-0.8751
ENSMUSG00000029033//Acap3	-0.315476032	5.176502635	-4.311433107	0.00121026	0.027632	-1.56135
ENSMUSG00000024962//Vegfb	-0.315316255	4.937963433	-4.900959849	0.000460207	0.017514	-0.51888
ENSMUSG00000041351//Rap1gap	-0.314850872	6.723924102	-4.69205184	0.00064477	0.020597	-1.0502
ENSMUSG00000038486//Sv2a	-0.31367518	7.979454737	-4.046708638	0.001896181	0.034663	-2.21494
ENSMUSG00000036002//Fam214b	-0.313648928	4.078648143	-4.180772194	0.001508884	0.030989	-1.59072
ENSMUSG00000003352//Cacnb3	-0.313633049	5.004196898	-4.640959276	0.000700845	0.021274	-0.96763
ENSMUSG00000040697//Dnajc16	-0.313459455	5.658938026	-4.245060054	0.001353364	0.029284	-1.7402
ENSMUSG00000016346//Kcnq2	-0.313332411	6.991232033	-4.58332111	0.000770309	0.022159	-1.24693
ENSMUSG00000049690//Nckap5	-0.312897286	4.381808304	-3.788727497	0.002960433	0.042058	-2.34504
ENSMUSG00000039126//Prune2	-0.312460346	7.636253074	-4.989072256	0.000399925	0.016273	-0.58337
ENSMUSG00000047658//Gal3st3	-0.312086191	4.65206681	-4.154127067	0.001578706	0.031547	-1.75011
ENSMUSG00000004207//Psap	-0.311828421	8.511898489	-4.875596524	0.000479284	0.017794	-0.79286
ENSMUSG00000037989//Wnk2	-0.311257415	6.560948029	-4.853016453	0.000496969	0.018122	-0.77006
ENSMUSG00000038034//Igsf8	-0.311163531	5.928067676	-3.97053532	0.002161078	0.036261	-2.2525
ENSMUSG00000038366//Lasp1	-0.311083311	5.808560354	-4.515207868	0.000861825	0.023581	-1.28748
ENSMUSG00000025579//Gaa	-0.311064531	6.662190095	-4.071829003	0.00181642	0.033827	-2.12452
ENSMUSG00000020926//Adam11	-0.311059384	5.611539226	-3.870502026	0.002568524	0.03933	-2.39667
ENSMUSG00000070000//Fcho1	-0.310778421	4.892960886	-3.645004149	0.003805932	0.047838	-2.6969
ENSMUSG00000054863//Fam19a5	-0.309855286	4.773960973	-4.892371085	0.000466576	0.017662	-0.50628
ENSMUSG00000058793//Cds2	-0.308391447	8.88730542	-5.27688895	0.000254759	0.013429	-0.13524
ENSMUSG00000042589//Cux2	-0.307607591	5.479656272	-4.083970325	0.001779128	0.033564	-2.00174
ENSMUSG00000038248//Sobp	-0.307507356	5.587147704	-4.304776725	0.00122387	0.02787	-1.62742
ENSMUSG00000021194//Chga	-0.30729107	6.832618164	-4.407692831	0.00103021	0.025794	-1.54357
ENSMUSG00000022456//Sept3	-0.307227265	7.290561071	-4.909864532	0.000453701	0.017402	-0.70435
ENSMUSG00000026883//Dab2ip	-0.307097781	6.405824676	-5.329557416	0.000234879	0.013065	0.024823
ENSMUSG00000070337//Gpr179	-0.30690033	7.617922975	-3.846349612	0.002678345	0.039911	-2.56166
ENSMUSG00000020396//Nefh	-0.306135004	6.586536701	-4.268726397	0.001300411	0.028652	-1.77433
ENSMUSG00000036427//Gpi1	-0.305684806	9.188917447	-4.354116612	0.001126667	0.026598	-1.69597
ENSMUSG00000047146//Tet1	-0.303947148	5.506708732	-4.976890441	0.000407737	0.016401	-0.47214
ENSMUSG00000050896//Rtn4rl2	-0.303706067	4.428012879	-4.191799517	0.001480938	0.030742	-1.6419
ENSMUSG00000086370//Ftx	-0.303355257	5.560769695	-4.434083017	0.000985915	0.025341	-1.39966
ENSMUSG00000052516//Robo2	-0.302552392	6.890887502	-4.079251328	0.001793527	0.033601	-2.12148
ENSMUSG00000021239//Vsx2	-0.302443898	7.485100156	-4.346240303	0.001141626	0.026814	-1.67443
ENSMUSG00000026179//Pnkd	-0.302076261	6.111741372	-5.137402668	0.000316525	0.014904	-0.26786
ENSMUSG00000040265//Dnm3	-0.302009062	8.433775903	-4.972739295	0.000410436	0.01647	-0.62896
ENSMUSG00000025964//Adam23	-0.301292537	6.429066733	-5.225856488	0.00027573	0.013688	-0.14501
ENSMUSG00000054843//Atrnl1	-0.301291238	6.473348504	-4.854322708	0.000495928	0.018122	-0.76306
ENSMUSG00000042292//Mkl1	-0.301281878	4.568804817	-4.166883511	0.001544868	0.031248	-1.71244
ENSMUSG00000041329//Atp1b2	-0.300295846	10.54211029	-4.696820596	0.000639782	0.02051	-1.12642
ENSMUSG00000009035//Tmem184b	-0.299714272	5.402238314	-4.6062364	0.000741863	0.021664	-1.08385
ENSMUSG00000020723//Cacng4	-0.299443632	5.748463218	-4.51682365	0.000859527	0.023556	-1.27829
ENSMUSG00000029648//Flt1	-0.299283335	6.915664613	-4.044550244	0.001903203	0.034663	-2.18388
ENSMUSG00000031748//Gnao1	-0.298293264	8.491818447	-4.072347587	0.00181481	0.033827	-2.18
ENSMUSG00000035637//Grhpr	-0.298194286	3.737568207	-3.603416961	0.004094403	0.049888	-2.53808
ENSMUSG00000017721//Pigt	-0.298107541	6.813614762	-4.716608985	0.000619516	0.020082	-1.01231
ENSMUSG00000034075//Zdhhc5	-0.297890098	7.93915676	-5.657054607	0.000142975	0.010714	0.490393
ENSMUSG00000030397//Mark4	-0.297824764	6.025146692	-4.646455097	0.000694574	0.021236	-1.08305
ENSMUSG00000051790//Nlgn2	-0.297792015	7.130423021	-4.865564976	0.000487058	0.017967	-0.77294
ENSMUSG00000039542//Ncam1	-0.29736243	8.66538817	-4.262170731	0.001314859	0.028773	-1.84864

ENSMUSG00000026833//Olfm1	-0.297155949	6.956242893	-4.568119912	0.000789809	0.022502	-1.27165
ENSMUSG00000021087//Rtn1	-0.296998122	7.414925177	-4.7209036	0.000615208	0.020017	-1.02734
ENSMUSG00000062661//Ncs1	-0.296833319	5.378309593	-4.220838931	0.001409896	0.029982	-1.74777
ENSMUSG00000036985//Zdhhc9	-0.295807388	4.896830597	-4.109256859	0.001703998	0.032784	-1.8717
ENSMUSG00000003378//Grik5	-0.295760599	6.08077218	-5.254900793	0.00026358	0.01343	-0.07324
ENSMUSG00000030096//Slc6a6	-0.295554295	10.86248379	-5.439109959	0.000198607	0.01215	0.097428
ENSMUSG00000041353//Tmem29	-0.295272428	4.195994996	-4.004490809	0.002038539	0.035756	-1.92456
ENSMUSG00000024935//Slc1a1	-0.294849317	4.613577084	-3.850621113	0.002658574	0.039823	-2.27968
ENSMUSG00000053963//Stum	-0.294782975	3.86589276	-3.720657851	0.003333649	0.044797	-2.35768
ENSMUSG00000017400//Stac2	-0.29472439	6.371072775	-3.865424477	0.002591218	0.039338	-2.47505
ENSMUSG00000050148//Ubqln2	-0.293962127	7.031466653	-4.608855823	0.000738682	0.021664	-1.20472
ENSMUSG00000037692//Ahdc1	-0.293417512	5.865427634	-4.069848996	0.001822578	0.033869	-2.07047
ENSMUSG00000040560//Wdr7	-0.293025521	6.877136678	-4.081777694	0.001785803	0.033601	-2.11646
ENSMUSG00000070304//Scn2b	-0.292742048	5.976483796	-4.482424254	0.000909875	0.024286	-1.36019
ENSMUSG00000024130//Abca3	-0.292696084	5.614717477	-4.841440265	0.000506302	0.018123	-0.71135
ENSMUSG00000040249//Lrp1	-0.292479441	7.649369795	-3.754045877	0.003144867	0.043609	-2.72787
ENSMUSG00000030103//Bhlhe40	-0.292419883	5.491707303	-4.032823009	0.001941826	0.035021	-2.09366
ENSMUSG00000003949//Hlf	-0.292372041	8.124056295	-4.379128715	0.00108051	0.026405	-1.63452
ENSMUSG00000037709//Fam13a	-0.292289925	4.645283844	-3.959448178	0.002202726	0.036372	-2.09221
ENSMUSG00000025867//Cplx2	-0.290536804	8.060289973	-4.174352328	0.001525407	0.031071	-1.99141
ENSMUSG00000041112//Elmo1	-0.29036222	6.153937049	-4.291580125	0.001251327	0.028199	-1.70646
ENSMUSG00000028469//Npr2	-0.289670777	5.304774441	-4.269482685	0.001298755	0.028652	-1.65285
ENSMUSG00000033615//Cplx1	-0.289355434	6.690935265	-4.905358973	0.000456981	0.017483	-0.6886
ENSMUSG00000020900//Myh10	-0.288662903	7.463136812	-4.365563031	0.001105293	0.026536	-1.64006
ENSMUSG00000020135//Apc2	-0.287915581	6.440811119	-3.6951784	0.003485561	0.04588	-2.7842
ENSMUSG00000014602//Kif1a	-0.287853	9.445721856	-4.670032499	0.000668335	0.021012	-1.15511
ENSMUSG00000040811//Eml2	-0.287448277	5.138622986	-4.831703228	0.000514294	0.018236	-0.66545
ENSMUSG00000039419//Cntnap2	-0.286884855	6.615147556	-4.963021382	0.000416828	0.016538	-0.5886
ENSMUSG00000034187//Nsf	-0.286800714	8.966225882	-4.175536174	0.001522346	0.031061	-2.0054
ENSMUSG00000037306//Man1c1	-0.286188159	4.992592086	-4.329155697	0.00117479	0.027037	-1.50247
ENSMUSG00000027210//Meis2	-0.285928355	7.358403476	-5.648379026	0.00014484	0.010755	0.49353
ENSMUSG00000069601//Ank3	-0.285609166	8.873411807	-3.798006469	0.002913011	0.041795	-2.67474
ENSMUSG00000029512//Ulk1	-0.285240539	6.388438164	-3.918933573	0.002362138	0.037432	-2.38074
ENSMUSG00000029095//Ablim2	-0.285101443	5.11781573	-3.966870747	0.002174752	0.036261	-2.15856
ENSMUSG00000024524//Gnal	-0.284781485	5.319616315	-4.626081035	0.000718121	0.021402	-1.03908
ENSMUSG00000028830//AU040320	-0.284469908	6.178368393	-4.825330118	0.000519598	0.018387	-0.79229
ENSMUSG00000028434//Epb41l4b	-0.284336219	4.554188763	-3.782377805	0.002993345	0.042318	-2.39006
ENSMUSG00000011751//Sptbn4	-0.284124566	5.826186463	-3.794744599	0.002929591	0.04186	-2.55588
ENSMUSG00000030207//Fam234b	-0.283734658	5.98776995	-3.992213965	0.002081989	0.035995	-2.21967
ENSMUSG00000068747//Sort1	-0.283168864	7.573227671	-4.956408765	0.000421237	0.016596	-0.63593
ENSMUSG00000037541//Shank2	-0.283083967	6.589288932	-4.066215078	0.001833937	0.033899	-2.1308
ENSMUSG00000031066//Usp11	-0.281948951	6.062173927	-3.952420278	0.002229557	0.036443	-2.2968
ENSMUSG00000044700//Tmem201	-0.281677067	6.852772434	-4.124784012	0.001659508	0.032402	-2.03949
ENSMUSG00000025738//Fbxl16	-0.28142738	6.835396888	-4.406797726	0.001031749	0.025794	-1.54524
ENSMUSG00000041879//Ipo9	-0.281242798	7.913519216	-5.899110655	0.000100031	0.009487	0.867934
ENSMUSG00000063904//Dpp3	-0.281203972	4.501484808	-3.790768378	0.002949935	0.042034	-2.36489
ENSMUSG00000028476//Reck	-0.280639032	4.652988767	-3.835809979	0.002727782	0.040405	-2.31329
ENSMUSG00000022240//Ctnnd2	-0.280615425	6.061263356	-3.834893837	0.002732124	0.040435	-2.50619
ENSMUSG00000032625//Thsd7a	-0.280171777	6.506720995	-3.904614935	0.002421301	0.037799	-2.41339
ENSMUSG00000021268//Meg3	-0.280064669	10.40449523	-3.790474158	0.002951446	0.042034	-2.71209
ENSMUSG00000029467//Atp2a2	-0.279784084	8.126079147	-4.004252782	0.002039372	0.035756	-2.29361
ENSMUSG00000070683//Lactbl1	-0.279651274	7.228412463	-4.111613429	0.001697166	0.032784	-2.07713
ENSMUSG00000063873//Slc24a3	-0.279373732	7.104747352	-3.997648698	0.002062637	0.035955	-2.27436
ENSMUSG00000038967//Pdk2	-0.278798186	6.013423213	-4.427851973	0.000996191	0.025366	-1.45782
ENSMUSG00000031840//Rab3a	-0.278628603	7.452029066	-4.109974243	0.001701915	0.032784	-2.08783
ENSMUSG00000061751//Kalrn	-0.278186709	4.875660529	-3.606070753	0.004075339	0.049789	-2.76382
ENSMUSG00000018909//Arrb1	-0.277874108	6.922217385	-4.190438063	0.001484358	0.030777	-1.9267
ENSMUSG00000057897//Camk2b	-0.276778796	7.154455243	-5.090348681	0.000340787	0.015219	-0.39943
ENSMUSG00000002332//Dhrs1	-0.276185549	4.491522633	-3.983152746	0.002114674	0.036122	-2.0212
ENSMUSG00000037996//Slc24a2	-0.275969409	7.15886113	-4.44304544	0.00097133	0.025325	-1.49492
ENSMUSG00000035504//Reep6	-0.275699954	10.4417832	-4.706306818	0.000629982	0.020285	-1.10877

ENSMUSG00000024277//Mapre2	-0.274952101	7.830176195	-4.164524845	0.001551067	0.031307	-2.00303
ENSMUSG00000022263//Trio	-0.274741689	6.766392624	-4.108819688	0.001705268	0.032784	-2.06394
ENSMUSG00000042050//Wdr60	-0.274266149	6.240871209	-4.16034062	0.001562128	0.031373	-1.94309
ENSMUSG00000042734//Ttc9	-0.274155273	4.686414892	-3.905621312	0.002417093	0.037789	-2.1952
ENSMUSG00000028760//Eif4g3	-0.27410952	8.23408454	-5.116986758	0.00032682	0.014912	-0.38614
ENSMUSG00000038807//Rap1gap2	-0.274072076	6.053186477	-3.91864362	0.002363321	0.037432	-2.35614
ENSMUSG00000052928//Ctif	-0.273727709	6.353289754	-4.785543168	0.000554036	0.01895	-0.87171
ENSMUSG00000042992//Borcs5	-0.273600425	4.106135772	-3.642787591	0.003820766	0.047932	-2.5489
ENSMUSG00000020894//Vamp2	-0.273489129	9.626311713	-4.600450178	0.00074894	0.021797	-1.27689
ENSMUSG00000039220//Ppp1r10	-0.273472804	6.121352967	-4.739766234	0.000596655	0.019842	-0.93234
ENSMUSG00000022619//Mapk8ip2	-0.273306807	6.927553918	-4.750337903	0.000586515	0.019688	-0.95972
ENSMUSG00000053580//Tanc2	-0.272777309	6.272044825	-4.076278442	0.00180266	0.033712	-2.0936
ENSMUSG00000027624//Epb41l1	-0.272464047	7.191080339	-4.631844602	0.000711376	0.021347	-1.17127
ENSMUSG00000020668//Kif3c	-0.272231768	6.206187335	-3.838612891	0.002714544	0.040278	-2.5113
ENSMUSG00000031137//Fgf13	-0.271303137	5.143807587	-4.016442055	0.001997146	0.035372	-2.07462
ENSMUSG00000034341//Wbp2	-0.271093314	8.00970655	-4.612917494	0.000733779	0.021664	-1.22845
ENSMUSG00000041729//Coro2b	-0.27105095	7.483524203	-4.723228116	0.000612889	0.020017	-1.02565
ENSMUSG00000062785//Kcnc3	-0.270601943	6.694253899	-4.094356885	0.001747859	0.033118	-2.08625
ENSMUSG00000015476//Prrt1	-0.270364958	6.015981198	-4.812821992	0.000530175	0.018498	-0.80018
ENSMUSG00000066357//Wdr6	-0.269697878	6.964510302	-4.495738393	0.000890031	0.02405	-1.39654
ENSMUSG00000038805//Six3	-0.269538167	6.645476805	-4.160650685	0.001561306	0.031373	-1.96698
ENSMUSG00000037905//Bri3bp	-0.269443601	5.849464668	-3.977986099	0.002133549	0.03616	-2.23162
ENSMUSG00000013033//Adgrl1	-0.268985547	7.020566533	-4.096517802	0.001741426	0.033104	-2.09606
ENSMUSG00000009569//Mkl2	-0.268604558	5.372502015	-3.653658759	0.003748578	0.047403	-2.75432
ENSMUSG00000044667//Plppr4	-0.268573972	5.711563446	-3.959029409	0.002204315	0.036372	-2.25056
ENSMUSG00000017740//Slc12a5	-0.268436083	9.225310022	-4.156868425	0.001571369	0.031491	-2.04216
ENSMUSG00000035513//Ntng2	-0.268396125	7.013990016	-3.905279762	0.00241852	0.037789	-2.43525
ENSMUSG00000045532//C1ql1	-0.268345129	5.819415192	-4.206062689	0.001445589	0.03027	-1.82598
ENSMUSG00000020810//Cygb	-0.268302391	6.519574912	-4.346034475	0.001142019	0.026814	-1.6359
ENSMUSG00000030741//Spns1	-0.268085082	4.856859866	-3.750580075	0.003163939	0.043795	-2.50162
ENSMUSG00000038077//Kcna6	-0.267848549	4.703021524	-3.752771389	0.003151866	0.043671	-2.47062
ENSMUSG00000024897//Apba1	-0.267803099	7.348491597	-3.993158412	0.002078612	0.035974	-2.29116
ENSMUSG00000026116//Tmem131	-0.266656567	6.892160862	-4.369444145	0.001098143	0.026536	-1.61248
ENSMUSG00000024403//Atp6v1g2	-0.265735911	6.50188993	-4.011132934	0.002015425	0.035568	-2.22371
ENSMUSG00000020814//Mxra7	-0.265201718	6.990085031	-5.360187415	0.000224079	0.012926	0.047103
ENSMUSG00000024462//Gabbr1	-0.265011092	7.67524467	-4.481026309	0.000911985	0.024286	-1.44651
ENSMUSG00000036564//Ndrg4	-0.264695507	8.193674758	-3.746093212	0.003188807	0.043988	-2.75592
ENSMUSG00000057880//Abat	-0.264287835	7.006704264	-3.696944496	0.003474804	0.045808	-2.80784
ENSMUSG00000024947//Men1	-0.264010904	5.673774945	-4.395787681	0.00105087	0.026024	-1.47924
ENSMUSG00000024193//Phf1	-0.263123551	5.877589114	-4.032460818	0.001943032	0.035021	-2.13779
ENSMUSG00000046574//Prr12	-0.26276342	6.778849569	-4.170242791	0.001536083	0.031191	-1.95622
ENSMUSG00000030257//Srgap3	-0.26265796	8.012815597	-3.732229331	0.003266931	0.044423	-2.77678
ENSMUSG00000024855//Pacs1	-0.26237272	5.839078183	-3.911197571	0.002393914	0.037701	-2.34934
ENSMUSG00000047181//Samd14	-0.261974014	6.568350273	-4.579379784	0.000775316	0.022266	-1.23514
ENSMUSG00000022212//Cpne6	-0.260760002	5.058811361	-3.678896555	0.003586358	0.046374	-2.66313
ENSMUSG00000048895//Cdk5r1	-0.260267881	5.847539394	-3.816354454	0.002821537	0.041093	-2.51939
ENSMUSG00000045007//Tubg2	-0.25949679	4.317514349	-3.617750996	0.003992515	0.049145	-2.63765
ENSMUSG00000019558//Slc6a8	-0.25828098	6.005586499	-3.956726725	0.002213076	0.036419	-2.28431
ENSMUSG00000018476//Kdm6b	-0.257948357	6.349232091	-4.339043018	0.001155476	0.026912	-1.6378
ENSMUSG00000028430//Nol6	-0.257804264	5.28132196	-3.965815309	0.002178707	0.036261	-2.18437
ENSMUSG00000005982//Naa60	-0.257513634	5.233420747	-3.63249858	0.003890408	0.048487	-2.77283
ENSMUSG00000017692//Rhbdl3	-0.256642112	4.89961645	-3.655581082	0.00373596	0.047347	-2.67906
ENSMUSG00000034135//Sik3	-0.256556901	6.835238218	-3.801434533	0.00289569	0.041744	-2.61355
ENSMUSG00000033152//Podxl2	-0.256533671	6.170658218	-3.788909122	0.002959498	0.042058	-2.59748
ENSMUSG00000022517//Mgrn1	-0.255417036	6.528445595	-4.682986737	0.000654364	0.020747	-1.05604
ENSMUSG00000022812//Gsk3b	-0.255007363	8.151417336	-4.440293376	0.000975785	0.025325	-1.52893
ENSMUSG00000061887//Ssbp3	-0.253692631	6.894242677	-4.183596721	0.001501674	0.030951	-1.93762
ENSMUSG00000039652//Cpeb3	-0.253358163	6.361117711	-3.802927195	0.002888181	0.041714	-2.58611
ENSMUSG00000051391//Ywhag	-0.251617688	7.833104503	-3.963672368	0.00218676	0.036324	-2.35863
ENSMUSG00000022436//Sh3bp1	-0.251350967	6.098261468	-4.461085609	0.000942653	0.024779	-1.40742
ENSMUSG00000021719//Rgs7bp	-0.250978896	6.350445041	-3.706887613	0.003414881	0.045327	-2.75753

ENSMUSG00000033763//Mtss1l	-0.250852802	7.053892161	-3.850948719	0.002657064	0.039823	-2.53376
ENSMUSG00000032012//Nectin1	-0.250540413	6.488429553	-3.997077268	0.002064663	0.035955	-2.24786
ENSMUSG00000030352//Tspan9	-0.249074702	5.477730834	-3.819326623	0.002807001	0.040985	-2.47156
ENSMUSG00000017344//Vtn	-0.248154459	9.074553299	-4.069854873	0.00182256	0.033869	-2.19358
ENSMUSG00000030306//Tmtc1	-0.24788913	5.13582051	-3.64207156	0.003825571	0.047946	-2.74119
ENSMUSG00000039728//Slc6a5	-0.247866618	4.707050658	-3.891758528	0.002475731	0.038371	-2.22361
ENSMUSG00000032187//Smarca4	-0.24721955	7.936263457	-3.946895033	0.002250889	0.036636	-2.39117
ENSMUSG00000001424//Snd1	-0.247050259	5.309130279	-3.759502048	0.003115082	0.0433	-2.55599
ENSMUSG00000024308//Tapbp	-0.246938871	5.285979714	-3.779119084	0.003010382	0.04249	-2.51766
ENSMUSG00000040383//Aqr	-0.246598895	6.895471401	-3.886369687	0.002498922	0.038629	-2.4643
ENSMUSG00000022415//Syngr1	-0.246062671	7.861425398	-4.397143872	0.001048495	0.026024	-1.59696
ENSMUSG00000037049//Smpd1	-0.245873144	6.301485479	-4.124777533	0.001659526	0.032402	-2.01007
ENSMUSG00000015944//Gatsl2	-0.24585364	6.91908412	-3.62594337	0.003935458	0.048757	-2.93202
ENSMUSG00000038740//Mvb12b	-0.245722455	5.756695079	-4.018816356	0.001989028	0.035283	-2.14944
ENSMUSG00000035781//R3hdm4	-0.245493774	5.369575417	-3.636475805	0.003863334	0.048222	-2.78478
ENSMUSG00000028803//Nipal3	-0.245037856	6.826712586	-4.427792939	0.000996289	0.025366	-1.50847
ENSMUSG00000018474//Chd3	-0.244667982	7.959367108	-4.098330023	0.001736049	0.033082	-2.12312
ENSMUSG00000032890//Rims3	-0.244528896	6.644642083	-3.750233833	0.003165851	0.043795	-2.6966
ENSMUSG00000025422//Agap2	-0.2427764	6.157064448	-4.223040581	0.001404657	0.029909	-1.82667
ENSMUSG00000034902//Pip5k1c	-0.24231755	7.47849673	-4.336997131	0.001159445	0.026933	-1.69032
ENSMUSG00000042155//Klhl23	-0.242293215	6.4358801	-3.924232372	0.002340626	0.03731	-2.3742
ENSMUSG00000050556//Kcnb1	-0.242261259	9.546786927	-4.218586986	0.001415275	0.030025	-1.93851
ENSMUSG00000027457//Snph	-0.241570256	5.892489539	-3.722516123	0.003322841	0.044755	-2.69182
ENSMUSG00000036026//Tmem63b	-0.24086849	6.70451033	-4.439035043	0.000977828	0.025325	-1.48368
ENSMUSG00000020431//Adcy1	-0.240656935	9.082109283	-4.349745695	0.001134943	0.026757	-1.70198
ENSMUSG00000079179//Rab10os	-0.240647369	5.227973639	-3.743372905	0.003203983	0.044077	-2.5732
ENSMUSG00000028137//Celf3	-0.240644196	6.609319318	-4.593019847	0.000758132	0.021954	-1.21381
ENSMUSG00000020456//Ogdh	-0.239325461	7.685374109	-4.137780736	0.0016232	0.032047	-2.04611
ENSMUSG00000020658//Efr3b	-0.239183239	6.494494869	-3.994864755	0.002072526	0.035974	-2.25214
ENSMUSG00000054728//Phactr1	-0.237794532	7.00386737	-3.908001423	0.002407171	0.037731	-2.43
ENSMUSG00000038256//Bcl9	-0.237209499	6.025145743	-3.668758759	0.003650633	0.046812	-2.80079
ENSMUSG00000079055//Slc8a3	-0.236873646	6.233260467	-3.825258763	0.002778221	0.040666	-2.53715
ENSMUSG00000026576//Atp1b1	-0.236695074	8.337734454	-3.903583154	0.002425623	0.037832	-2.47706
ENSMUSG00000044647//Csrnp3	-0.236318139	6.412995537	-3.673030203	0.003623408	0.0466	-2.82228
ENSMUSG00000028854//Slc9a1	-0.236204469	5.814981385	-3.923326853	0.002344288	0.037335	-2.32528
ENSMUSG00000057378//Ryr3	-0.234849319	5.26054632	-3.648264114	0.003784223	0.047696	-2.74847
ENSMUSG00000006494//Pdk1	-0.233766901	6.06807756	-3.952856096	0.002227883	0.036443	-2.29652
ENSMUSG00000038143//Stox2	-0.232475144	7.287491568	-3.72525388	0.003306983	0.044611	-2.76746
ENSMUSG00000017639//Rab11fip4	-0.231287385	6.745371706	-3.984399228	0.002110146	0.036122	-2.28336
ENSMUSG00000027977//Ndst3	-0.230913899	5.273345173	-3.728800973	0.003286553	0.044439	-2.60591
ENSMUSG00000000538//Tom1l2	-0.230656341	7.323950829	-3.713030718	0.003378393	0.045188	-2.7907
ENSMUSG00000032936//Camkv	-0.229849756	6.3798667	-3.690346453	0.003515166	0.04606	-2.78912
ENSMUSG00000040276//Pacsin1	-0.229774505	8.531182928	-3.630483438	0.0039042	0.048523	-2.97008
ENSMUSG00000039740//Alg2	-0.229736701	6.501480414	-3.990361081	0.002088629	0.036038	-2.26055
ENSMUSG00000043670//Diras1	-0.229530729	6.169408483	-3.612231643	0.004031434	0.049535	-2.91449
ENSMUSG00000030530//Furin	-0.22912383	5.707586906	-3.688977816	0.003523598	0.046082	-2.7325
ENSMUSG00000004099//Dnmt1	-0.229070181	6.483896338	-4.164399458	0.001551397	0.031307	-1.95184
ENSMUSG00000022048//Dpysl2	-0.228954876	6.755714811	-4.380127399	0.001078709	0.026405	-1.58813
ENSMUSG00000037926//Ssh2	-0.228913439	5.920729216	-3.697851893	0.003469291	0.04577	-2.73881
ENSMUSG00000031386//Hcfc1	-0.22875662	7.351277028	-3.688785036	0.003524788	0.046082	-2.83522
ENSMUSG00000032280//Tle3	-0.228496859	5.592320849	-3.671814997	0.003631132	0.046652	-2.7499
ENSMUSG00000026825//Dnm1	-0.228305068	9.176957095	-3.678201334	0.003590729	0.046374	-2.89434
ENSMUSG00000025404//R3hdm2	-0.226895134	8.385349458	-3.967567537	0.002172145	0.036261	-2.36399
ENSMUSG00000022957//Itsn1	-0.226257771	7.797962863	-4.328087975	0.001176896	0.027037	-1.71549
ENSMUSG00000029020//Mfn2	-0.226225194	7.003048402	-4.069014028	0.001825181	0.033881	-2.14401
ENSMUSG00000041720//Pi4ka	-0.226041665	7.572856771	-3.93265437	0.002306852	0.037065	-2.40629
ENSMUSG00000031993//Snx19	-0.225719128	6.164768794	-3.713670672	0.003374615	0.045173	-2.73184
ENSMUSG00000028078//Dclk2	-0.225382923	5.503859721	-3.688947807	0.003523783	0.046082	-2.70826
ENSMUSG00000075415//Fnbp1	-0.220892496	6.255923799	-3.701908695	0.003444753	0.045515	-2.75976
ENSMUSG00000029580//Actb	-0.220514198	8.503977249	-4.106995121	0.001710581	0.032794	-2.11894
ENSMUSG00000037470//Uggt1	-0.219566333	6.684029452	-3.62453975	0.003945174	0.048823	-2.92431

ENSMUSG00000060279//Ap2a1	-0.219147097	6.213958573	-3.684900935	0.003548841	0.046257	-2.78724
ENSMUSG00000025608//Podxl	-0.218962984	7.663908787	-3.932220038	0.002308581	0.037065	-2.40983
ENSMUSG00000034593//Myo5a	-0.217561188	8.322753968	-3.731887658	0.003268881	0.044423	-2.78402
ENSMUSG00000033565//Rbfox2	-0.216933899	6.762004877	-4.066241919	0.001833853	0.033899	-2.139
ENSMUSG00000029705//Cux1	-0.21639727	7.012706407	-3.812084867	0.002842554	0.041276	-2.60165
ENSMUSG00000024268//Celf4	-0.215980831	7.960571025	-3.980817924	0.002123182	0.036122	-2.33142
ENSMUSG00000026469//Xpr1	-0.21441814	7.611849329	-3.708370764	0.003406035	0.045327	-2.80871
ENSMUSG00000078440//Dohh	-0.214006432	6.33109383	-4.02379447	0.001972117	0.035213	-2.19069
ENSMUSG00000004263//Atn1	-0.213754088	7.715948871	-3.961268325	0.002195832	0.036368	-2.35967
ENSMUSG00000045039//Megf8	-0.212126931	7.097784073	-3.848603824	0.002667893	0.039824	-2.53959
ENSMUSG00000051853//Arf3	-0.210735732	7.042618535	-3.843039889	0.002693769	0.040072	-2.54745
ENSMUSG00000033128//Gga1	-0.208465544	5.506218538	-3.716966227	0.003355229	0.044982	-2.65831
ENSMUSG00000029120//Ppp2r2c	-0.208261403	6.033928897	-3.746781035	0.003184982	0.043988	-2.66155
ENSMUSG00000002496//Tsc2	-0.205373662	6.551742556	-3.677817257	0.003593145	0.046374	-2.82182
ENSMUSG00000005442//Cic	-0.205257442	7.820558306	-3.77732091	0.003019826	0.042554	-2.69105
ENSMUSG00000022708//Zbtb20	-0.204226578	8.17100897	-3.789408301	0.002956927	0.042058	-2.6778
ENSMUSG00000026594//Ralgps2	-0.202429886	7.04383448	-3.960605162	0.002198341	0.036368	-2.33789
ENSMUSG00000018750//Zbtb4	-0.201829427	8.422006932	-4.021299492	0.001980574	0.035213	-2.26921
ENSMUSG00000022390//Zc3h7b	-0.200675725	7.536318552	-3.664671906	0.003676879	0.047044	-2.88483
ENSMUSG00000000787//Ddx3x	-0.200018934	9.092747129	-3.638243835	0.003851361	0.048114	-2.96495
ENSMUSG00000026623//Lpgat1	-0.193293519	8.501532061	-3.604171667	0.004088972	0.049857	-3.01695
ENSMUSG00000031144//Syp	-0.191089277	8.362394193	-3.621301571	0.003967682	0.048948	-2.98357
ENSMUSG00000032405//Pias1	0.180546406	6.970053396	3.7030294	0.003438005	0.045489	-2.79546
ENSMUSG00000024976//Shoc2	0.186900979	6.912700138	3.644594617	0.003808669	0.047838	-2.89821
ENSMUSG00000020608//Smc6	0.187022485	6.573943181	3.741142804	0.003216479	0.044115	-2.7093
ENSMUSG00000040359//Ufl1	0.18938432	6.333487623	3.656483914	0.003730049	0.047325	-2.84691
ENSMUSG00000049606//Zfp644	0.191435533	6.967385705	3.675191404	0.003609713	0.046493	-2.84537
ENSMUSG00000020687//Cdc27	0.191494384	6.418828547	3.623552312	0.003952023	0.048873	-2.9116
ENSMUSG00000021796//Bmpr1a	0.191802039	7.787293162	3.811869912	0.002843617	0.041276	-2.62828
ENSMUSG00000037416//Dmxl1	0.192481036	7.585261819	3.731366749	0.003271857	0.044423	-2.7666
ENSMUSG00000029447//Cct6a	0.19704294	7.139102668	3.67313246	0.003622759	0.0466	-2.85565
ENSMUSG00000020076//Ddx50	0.197513629	6.552742374	3.683080818	0.003560171	0.046301	-2.81242
ENSMUSG00000037608//Bclaf1	0.199243324	8.513544269	3.841544851	0.002700766	0.040122	-2.59103
ENSMUSG00000057315//Arhgap24	0.199796099	6.737828523	3.719223876	0.003342015	0.04484	-2.75664
ENSMUSG00000020074//Ccar1	0.200660519	7.637973523	3.683910257	0.003555003	0.046269	-2.85344
ENSMUSG00000041408//Wapl	0.200718818	7.824150355	3.785185231	0.002978747	0.042146	-2.67705
ENSMUSG00000007739//Cct4	0.200788319	7.121299351	3.920126993	0.002357275	0.037432	-2.41285
ENSMUSG00000029169//Dhx15	0.200834361	7.408761562	3.721281042	0.00333002	0.044797	-2.77887
ENSMUSG00000024811//Tnks2	0.200967907	8.180777622	4.272278973	0.001292651	0.028611	-1.82213
ENSMUSG00000071172//Srsf3	0.201521453	7.751359086	3.818435386	0.002811352	0.041014	-2.61555
ENSMUSG00000058638//Zfp110	0.201734034	6.009933195	3.629606174	0.00391022	0.048548	-2.86984
ENSMUSG00000003226//Ranbp2	0.202197555	8.865290612	3.841340857	0.002701723	0.040122	-2.59706
ENSMUSG00000016559//H3f3b	0.203514582	9.374536368	4.228711848	0.001391255	0.029747	-1.91808
ENSMUSG00000020954//Strn3	0.203602319	7.064415511	3.742031202	0.003211495	0.04411	-2.72914
ENSMUSG00000025907//Rb1cc1	0.206386571	7.506933155	3.702701514	0.003439978	0.045489	-2.81552
ENSMUSG00000025220//Mgea5	0.206487942	7.718227666	3.82616823	0.002773835	0.040666	-2.60075
ENSMUSG00000033808//Tmem87a	0.206656602	6.226948129	3.755228461	0.003138386	0.043554	-2.66203
ENSMUSG00000039068//Zzz3	0.206839748	7.31711668	3.96938955	0.002165343	0.036261	-2.33229
ENSMUSG00000024993//Fam45a	0.206895367	6.71121222	3.7458912	0.003189932	0.043988	-2.7076
ENSMUSG00000029238//Clock	0.207271924	7.803487194	3.644316536	0.003810528	0.047838	-2.92944
ENSMUSG00000049969//Plekhf2	0.208823941	6.758375464	4.024098203	0.00197109	0.035213	-2.21348
ENSMUSG00000025235//Bbs4	0.209007226	7.27368728	3.987616946	0.002098505	0.036065	-2.29836
ENSMUSG00000037857//Nufip2	0.209306257	7.420737151	4.079227538	0.0017936	0.033601	-2.14112
ENSMUSG00000027615//Hps3	0.210163778	6.071023296	3.606875037	0.00406958	0.049768	-2.91596
ENSMUSG00000002028//Kmt2a	0.213445071	9.659477071	4.119914945	0.001673328	0.032631	-2.11405
ENSMUSG00000068823//Csde1	0.214388858	8.847804982	3.786420107	0.00297235	0.042124	-2.6951
ENSMUSG00000034252//Senp6	0.214418555	7.311705364	4.00522414	0.002035974	0.035756	-2.26844
ENSMUSG00000035898//Uba6	0.214683742	5.746751703	3.847954972	0.002670897	0.039834	-2.45224
ENSMUSG00000027259//Adal	0.214916914	6.541613836	3.682303503	0.003565021	0.046303	-2.81323
ENSMUSG00000053411//Cbx7	0.21579625	6.328847368	4.080885883	0.001788526	0.033601	-2.08948
ENSMUSG00000059486//Kbtbd2	0.216115498	5.895517852	3.868570911	0.002577131	0.03933	-2.43085

ENSMUSG00000032292//Nr2e3	0.216348208	8.794780238	4.325410741	0.001182194	0.027098	-1.7401
ENSMUSG00000035133//Arhgap5	0.216355409	8.200530049	3.974187661	0.002147537	0.036261	-2.3486
ENSMUSG00000060475//Wtap	0.216956569	6.447789799	3.86666316	0.002585663	0.039338	-2.47757
ENSMUSG00000074733//Zfp950	0.217199395	6.163720775	3.801266834	0.002896535	0.041744	-2.57481
ENSMUSG00000015748//Prpf3	0.217260802	5.992210682	3.778372477	0.003014299	0.042511	-2.6013
ENSMUSG00000054074//Skida1	0.217675319	7.226726616	3.81551946	0.002825635	0.041118	-2.60352
ENSMUSG00000045576//St7l	0.217678148	5.148446801	3.742339288	0.003209768	0.04411	-2.56333
ENSMUSG00000020922//Lsm12	0.218613989	5.702692709	3.727278309	0.003295307	0.044523	-2.66326
ENSMUSG00000019971//Cep290	0.219484071	8.46479501	3.602198011	0.00410319	0.04996	-3.01987
ENSMUSG00000041057//Wdr43	0.220483626	5.816849935	3.77993696	0.003006097	0.042464	-2.5814
ENSMUSG00000026655//Fam107b	0.221458957	6.530221624	4.205050502	0.001448068	0.03027	-1.88304
ENSMUSG00000030059//Tmf1	0.222125874	6.239224489	3.667567278	0.003658265	0.046875	-2.82023
ENSMUSG00000027423//Snx5	0.222254281	5.978774431	3.85261586	0.002649393	0.039823	-2.46733
ENSMUSG00000040565//Btaf1	0.222871052	7.175802267	3.738758777	0.003229894	0.044154	-2.73916
ENSMUSG00000038344//Txlng	0.223289002	5.286395145	3.762209202	0.003100413	0.043147	-2.54797
ENSMUSG00000020463//Ppp4r3b	0.223348038	7.264646329	4.049865722	0.00188596	0.034566	-2.18762
ENSMUSG00000052748//Swt1	0.224038714	5.546272878	3.830480289	0.00275314	0.04047	-2.46013
ENSMUSG00000026977//March7	0.224364631	7.230183449	3.844233235	0.002688197	0.040024	-2.5523
ENSMUSG00000056216//Cebpg	0.224524385	5.533047479	3.980507945	0.002124314	0.036122	-2.19145
ENSMUSG00000022987//Zfp641	0.225092762	5.179789046	3.638161386	0.003851919	0.048114	-2.75475
ENSMUSG00000051469//Zfp24	0.22550005	6.732933063	3.909770144	0.002399825	0.037701	-2.41559
ENSMUSG00000020063//Sirt1	0.225679498	6.409022557	3.628672383	0.003916638	0.048574	-2.90179
ENSMUSG00000033411//Ctdspl2	0.225702491	6.725774377	3.850091173	0.002661019	0.039823	-2.52176
ENSMUSG00000039361//Picalm	0.22609704	7.545630368	4.007300005	0.00202873	0.035677	-2.27269
ENSMUSG00000000275//Trim25	0.226213602	5.256567827	3.607462163	0.004065381	0.049768	-2.82119
ENSMUSG00000028522//Mier1	0.226959024	6.860785021	4.245125981	0.001353214	0.029284	-1.82824
ENSMUSG00000049811//Fam161a	0.227208537	8.683646401	3.964997815	0.002181776	0.036276	-2.37367
ENSMUSG00000027804//Ppid	0.227239124	6.446658103	3.938377438	0.00228419	0.037022	-2.34968
ENSMUSG00000026209//Dnpep	0.227514532	5.062924079	3.853709071	0.002644376	0.039823	-2.35123
ENSMUSG00000030224//Strap	0.227739887	6.59889883	3.768499844	0.0030666	0.04298	-2.66153
ENSMUSG00000030878//Cdr2	0.227909037	7.921308488	4.56102377	0.000799089	0.022612	-1.31536
ENSMUSG00000030660//Pik3c2a	0.228042359	6.415418433	3.740181885	0.003221879	0.044115	-2.70189
ENSMUSG00000021012//Zc3h14	0.228312389	6.666635521	3.691598544	0.003507469	0.046011	-2.8029
ENSMUSG00000032030//Cul5	0.228710509	7.741087537	4.16096433	0.001560474	0.031373	-2.00689
ENSMUSG00000030016//Zfp638	0.229046744	8.076128242	4.187425338	0.001491958	0.030787	-1.96877
ENSMUSG00000027088//Phospho2	0.230553101	5.257776445	3.648078662	0.003785455	0.047696	-2.7484
ENSMUSG00000063884//Ptcd3	0.230708389	5.956510972	3.915205645	0.002377396	0.03755	-2.35362
ENSMUSG00000034120//Srsf2	0.230917075	7.160925917	4.109872876	0.001702209	0.032784	-2.07773
ENSMUSG00000041278//Ttc1	0.231807828	5.442746866	3.892395903	0.002473003	0.038365	-2.33668
ENSMUSG00000037325//Bbs7	0.232752122	8.216231335	3.799358528	0.002906166	0.041789	-2.66093
ENSMUSG00000066152//Slc31a2	0.232876495	7.206013343	4.046363681	0.001897302	0.034663	-2.1917
ENSMUSG00000053907//Mat2a	0.233520557	8.738295615	4.264211512	0.001310343	0.028746	-1.84621
ENSMUSG00000027472//Pdrg1	0.234120974	5.005580241	3.767775914	0.003070471	0.042981	-2.49552
ENSMUSG00000003929//Zfp81	0.234122315	6.338578237	4.28716867	0.001260649	0.02832	-1.72758
ENSMUSG00000066441//Rdh11	0.234742555	5.832503989	3.644667069	0.003808184	0.047838	-2.82564
ENSMUSG00000070697//Utp3	0.235813211	6.169367083	4.137148506	0.001624947	0.032047	-1.97863
ENSMUSG00000032633//Flcn	0.236009292	6.795690462	4.23986056	0.001365297	0.029395	-1.83469
ENSMUSG00000027091//Zc3h15	0.236242459	6.67503283	3.926346714	0.002332099	0.037257	-2.38336
ENSMUSG00000029920//Smarcad1	0.237424515	5.755937344	4.152744227	0.00158242	0.031547	-1.91287
ENSMUSG00000022160//Mettl3	0.237497308	5.32216246	3.691779475	0.003506359	0.046011	-2.67909
ENSMUSG00000022538//Lsg1	0.237507939	5.200867033	3.693581018	0.003495319	0.045974	-2.65841
ENSMUSG00000027287//Snap23	0.238612765	5.195614092	3.669047867	0.003648784	0.046812	-2.70164
ENSMUSG00000000276//Dgke	0.238666063	8.751674036	4.135998156	0.00162813	0.032073	-2.0718
ENSMUSG00000028669//Pithd1	0.239784704	5.464183554	3.740431387	0.003220476	0.044115	-2.61094
ENSMUSG00000021785//Ngly1	0.240139433	5.167938167	3.768357168	0.003067362	0.04298	-2.51968
ENSMUSG00000019857//Asf1a	0.240314351	4.893839257	3.707372236	0.003411988	0.045327	-2.58526
ENSMUSG00000034349//Smc4	0.240471362	6.239689641	3.836727911	0.002723439	0.040375	-2.51711
ENSMUSG00000027680//Fxr1	0.241885416	6.525173049	3.960881563	0.002197295	0.036368	-2.31429
ENSMUSG00000046404//Yod1	0.242008672	5.671486256	4.478759431	0.000915419	0.024286	-1.33549
ENSMUSG00000035597//Prpf39	0.243324887	7.616517097	4.102570574	0.001723537	0.03298	-2.1062
ENSMUSG00000033883//D3Ertd254e	0.244694293	5.889014464	4.440787699	0.000974983	0.025325	-1.42383

ENSMUSG00000031283//Chrdl1	0.245032945	5.466264774	4.09481473	0.001746494	0.033118	-1.98091
ENSMUSG00000038957//Edc3	0.24512807	4.649000598	3.762506997	0.003098803	0.043147	-2.44332
ENSMUSG00000027708//Dcun1d1	0.24566527	4.962056289	3.935149515	0.002296943	0.037057	-2.19034
ENSMUSG00000032905//Atg12	0.245814702	6.374731835	4.223496317	0.001403575	0.029909	-1.84139
ENSMUSG00000056458//Mok	0.246193516	5.230987907	3.661850425	0.003695112	0.047173	-2.71977
ENSMUSG00000026034//Clk1	0.246406826	8.740085022	4.237097096	0.001371684	0.029489	-1.89377
ENSMUSG00000055116//Arntl	0.246946764	7.075474938	4.413895878	0.001019614	0.025675	-1.54237
ENSMUSG00000038535//Zfp280d	0.247221706	6.273294447	3.868324516	0.002578231	0.03933	-2.46312
ENSMUSG00000036825//Ssx2ip	0.247802464	7.000504635	4.607223563	0.000740663	0.021664	-1.20633
ENSMUSG00000002617//Zfp40	0.24795623	5.348967596	3.966856196	0.002174807	0.036261	-2.19179
ENSMUSG00000026577//Blzf1	0.248360998	5.739566494	3.869324136	0.00257377	0.03933	-2.41332
ENSMUSG00000025173//Wdr45b	0.249470562	5.204648646	3.906711857	0.002412542	0.037764	-2.27825
ENSMUSG00000029863//Casp2	0.250574529	4.714430448	3.79890387	0.002908466	0.041789	-2.39032
ENSMUSG00000028397//Kdm4c	0.250593812	8.24172019	5.168789718	0.000301363	0.014425	-0.3011
ENSMUSG00000058799//Nap1l1	0.250883544	8.822335757	3.720132662	0.003336711	0.044803	-2.81362
ENSMUSG00000028389//Zfp37	0.251121642	6.17531781	4.884467722	0.000472518	0.017732	-0.6927
ENSMUSG00000053347//Zfp943	0.251201797	5.729839498	3.666772921	0.003663362	0.046906	-2.77482
ENSMUSG00000020525//Ppm1d	0.251725031	6.130073926	3.980402544	0.002124699	0.036122	-2.25274
ENSMUSG00000024991//Eif3a	0.252020903	8.000564563	5.015634953	0.000383434	0.015938	-0.54881
ENSMUSG00000027206//Cops2	0.252310237	7.197807664	3.916388081	0.002372545	0.037544	-2.42234
ENSMUSG00000032253//Phip	0.252606165	7.875847786	4.395638696	0.001051131	0.026024	-1.59995
ENSMUSG00000070709//Zfp974	0.252758631	5.143143984	3.997427428	0.002063421	0.035955	-2.10819
ENSMUSG00000021282//Eif5	0.253532722	7.562727958	4.08786167	0.001767345	0.033378	-2.13053
ENSMUSG00000029246//Ppat	0.254012756	4.504750708	3.739713075	0.003224517	0.044115	-2.45666
ENSMUSG00000067942//Zfp160	0.254022545	4.971139445	3.606781954	0.004070246	0.049768	-2.77855
ENSMUSG00000042042//Csgalnact2	0.254091679	6.109422876	4.370380445	0.001096425	0.026536	-1.56555
ENSMUSG00000034218//Atm	0.254910276	5.894470414	4.127797222	0.001651016	0.032341	-1.971
ENSMUSG00000050565//Tor1aip2	0.255139681	6.031544815	4.428033753	0.000995889	0.025366	-1.45904
ENSMUSG00000041992//Rapgef5	0.255679228	7.174469187	5.054674156	0.000360488	0.015465	-0.45912
ENSMUSG00000039753//Fbxl5	0.256273571	8.444996742	4.628765887	0.00071497	0.021381	-1.21015
ENSMUSG00000095432//Zfp748	0.257714251	5.296440976	3.703540845	0.003434931	0.045489	-2.65447
ENSMUSG00000021608//Lpcat1	0.257980332	7.470618441	4.946959682	0.000427624	0.01681	-0.64841
ENSMUSG00000036676//Tmtc3	0.258552402	6.529344816	4.729153632	0.00060702	0.019873	-0.97763
ENSMUSG00000045817//Zfp36l2	0.258675737	7.433653267	5.097001032	0.00033724	0.015141	-0.39814
ENSMUSG00000048720//Tbc1d12	0.259174492	5.136858349	4.213176979	0.001428287	0.030117	-1.72717
ENSMUSG00000038332//Sesn1	0.25946463	6.415801798	4.976834385	0.000407774	0.016401	-0.5547
ENSMUSG00000040025//Ythdf2	0.259716642	5.402329221	3.979697179	0.002127279	0.036127	-2.17615
ENSMUSG00000022427//Tomm22	0.260954506	5.195093102	4.021439014	0.0019801	0.035213	-2.07329
ENSMUSG00000057337//Chst3	0.261103186	6.461865906	4.232362567	0.001382698	0.02966	-1.83116
ENSMUSG00000000827//Tpd52l2	0.261827962	5.282355359	4.283673555	0.001268085	0.02832	-1.62497
ENSMUSG00000028910//Mecr	0.26289591	4.612354918	4.056867414	0.001863495	0.034372	-1.91398
ENSMUSG00000049928//Glp2r	0.262913893	7.829780962	4.843811943	0.000504375	0.018123	-0.83195
ENSMUSG00000021392//Nol8	0.263155763	5.378693285	4.23986766	0.001365281	0.029395	-1.7145
ENSMUSG00000020124//Usp15	0.263373493	6.359178433	4.01060819	0.002017241	0.035568	-2.21595
ENSMUSG00000037730//Mynn	0.263498196	5.928371285	4.609069827	0.000738423	0.021664	-1.13812
ENSMUSG00000059820//AU019823	0.263868347	5.076086776	4.138876245	0.001620177	0.032047	-1.84828
ENSMUSG00000049488//Tmem67	0.264199159	5.051789852	3.869070841	0.0025749	0.03933	-2.3221
ENSMUSG00000050846//Zfp623	0.264491139	4.524903453	3.72460285	0.003310747	0.044627	-2.48759
ENSMUSG00000028909//Ptpru	0.264526206	5.89213828	3.825428118	0.002777404	0.040666	-2.50755
ENSMUSG00000034083//Ccdc174	0.264976101	5.422468387	4.189624356	0.001486407	0.030782	-1.80836
ENSMUSG00000004364//Cul3	0.265517368	8.569711475	4.109533076	0.001703195	0.032784	-2.11558
ENSMUSG00000018848//Rars	0.265688325	5.796626521	4.397557337	0.001047772	0.026024	-1.48949
ENSMUSG00000022724//Riox2	0.267490678	4.539430805	3.986849838	0.002101274	0.036077	-2.02388
ENSMUSG00000053799//Exoc6	0.2681778	4.789478917	3.994801205	0.002072753	0.035974	-2.05551
ENSMUSG00000015757//Ppil4	0.268311542	6.599276328	4.220152907	0.001411532	0.029982	-1.86007
ENSMUSG00000031770//Herpud1	0.268593391	5.74445686	3.897355456	0.002451881	0.038141	-2.36389
ENSMUSG00000079108//Srp54c	0.269042412	6.288352269	4.040718053	0.001915735	0.034798	-2.15769
ENSMUSG00000022241//Tars	0.269214276	5.940594907	4.367594196	0.001101545	0.026536	-1.55568
ENSMUSG00000037134//Prmt9	0.26941987	4.890621287	4.360216093	0.001115224	0.026544	-1.43202
ENSMUSG00000028676//Srsf10	0.270242868	7.65977295	5.069925444	0.000351922	0.015293	-0.44991
ENSMUSG00000020677//Ddx52	0.270855123	5.88649199	3.919049511	0.002361666	0.037432	-2.34002

ENSMUSG00000050332//Amer1	0.271155736	4.705627736	4.110002146	0.001701834	0.032784	-1.83734
ENSMUSG00000067367//Lyar	0.271510145	7.187548321	4.644256672	0.000697075	0.021236	-1.14993
ENSMUSG00000071266//Zfp946	0.271528607	6.157145259	3.983343412	0.002113981	0.036122	-2.24972
ENSMUSG00000006599//Gtf2h1	0.27196472	6.558980012	4.3278387	0.001177388	0.027037	-1.66969
ENSMUSG00000027274//Mkks	0.272215197	5.176570358	3.936102208	0.002293171	0.037057	-2.2219
ENSMUSG00000032667//Pon2	0.272615162	5.772689114	3.849606356	0.002663258	0.039823	-2.45213
ENSMUSG00000020064//Herc4	0.274014549	6.277433448	4.025318093	0.001966971	0.035197	-2.18419
ENSMUSG00000021945//Zmym2	0.274802198	7.751346403	4.875932508	0.000479026	0.017794	-0.77586
ENSMUSG00000022858//Tra2b	0.274876383	7.210689221	5.16161994	0.000304757	0.014468	-0.28413
ENSMUSG00000033799//Fam208b	0.275150743	7.065217821	4.960061543	0.000418796	0.016538	-0.61238
ENSMUSG00000067928//Zfp760	0.275245294	5.270371015	4.577807922	0.000777322	0.022287	-1.11482
ENSMUSG00000058291//Zfp68	0.275514417	6.208858473	4.804589085	0.00053726	0.018562	-0.82949
ENSMUSG00000068854//Hist2h2be	0.275709081	4.428579095	3.946086684	0.002254027	0.036636	-2.07466
ENSMUSG00000050711//Scg2	0.275889174	8.791836113	5.214813176	0.000280504	0.013851	-0.23505
ENSMUSG00000037029//Zfp146	0.276424102	6.182620281	3.970519198	0.002161137	0.036261	-2.2745
ENSMUSG00000027132//Katnbl1	0.278496682	5.184339813	4.305063126	0.001223281	0.02787	-1.57358
ENSMUSG00000029422//Rsrc2	0.278877656	8.424358084	4.942559031	0.000430633	0.016852	-0.67916
ENSMUSG00000073678//Pgap1	0.278933585	5.872046216	3.744824928	0.003195873	0.044011	-2.64984
ENSMUSG00000038774//Ascc3	0.280532191	5.731082257	3.736047931	0.003245218	0.044294	-2.65067
ENSMUSG00000031827//Cotl1	0.28053881	5.799946258	4.209678264	0.001436769	0.030214	-1.81758
ENSMUSG00000001627//Ifrd1	0.280713825	5.476994571	3.851999689	0.002652226	0.039823	-2.41314
ENSMUSG00000025898//Cwf19l2	0.280752053	5.691932884	4.089488713	0.001762443	0.033358	-2.01736
ENSMUSG00000032599//Ip6k2	0.281485183	8.311930933	3.679350057	0.00358351	0.046374	-2.87818
ENSMUSG00000047141//Zfp654	0.281506206	7.527032481	5.080415474	0.000346155	0.015265	-0.42854
ENSMUSG00000040729//Cep126	0.281846052	5.758645916	4.630331979	0.000713139	0.021363	-1.08484
ENSMUSG00000026646//Suv39h2	0.282922542	5.183568441	4.554037316	0.000808337	0.022631	-1.14314
ENSMUSG00000026020//Nop58	0.283178482	6.611275206	4.462913638	0.000939797	0.024777	-1.43783
ENSMUSG00000040321//Zfp770	0.28438467	5.786353273	4.404398808	0.001035883	0.02586	-1.47653
ENSMUSG00000044018//Mrpl50	0.284532928	5.295180627	3.792711195	0.002939977	0.041974	-2.49467
ENSMUSG00000028869//Gnl2	0.284931996	6.458599058	4.835722505	0.000510979	0.018193	-0.79347
ENSMUSG00000006288//Ttc5	0.284956127	4.961584043	3.973280113	0.002150894	0.036261	-2.12262
ENSMUSG00000026377//Nifk	0.285233395	5.100127493	4.037946169	0.001924854	0.034838	-2.02997
ENSMUSG00000025616//Usp16	0.286394207	6.086173814	4.259376125	0.001321068	0.028872	-1.7572
ENSMUSG00000000058//Cav2	0.287169421	4.955479575	3.955535972	0.00221762	0.036419	-2.1531
ENSMUSG00000035517//Tdrd7	0.287359989	8.635671506	4.556722386	0.000804769	0.022626	-1.3369
ENSMUSG00000027829//Ccnl1	0.287366491	7.523747473	4.814422845	0.000528808	0.018492	-0.87259
ENSMUSG00000025439//Clns1a	0.287537256	5.059595424	3.986190205	0.002103658	0.036082	-2.11525
ENSMUSG00000039763//Dnajc28	0.287584775	5.709976278	4.173519184	0.001527565	0.031079	-1.87132
ENSMUSG00000041438//Utp4	0.288290408	4.685934025	3.954413216	0.002221914	0.036421	-2.10853
ENSMUSG00000058402//Zfp420	0.289965171	5.314691218	5.023112167	0.000378922	0.015886	-0.37096
ENSMUSG00000028187//Rpf1	0.29020065	5.646330578	4.690670507	0.000646222	0.020602	-0.96983
ENSMUSG00000020290//Xpo1	0.290476521	7.951532276	4.418443555	0.001011919	0.025555	-1.56227
ENSMUSG00000040782//Cop1	0.290592337	5.992379887	5.230260031	0.00027385	0.013681	-0.10605
ENSMUSG00000024528//Srfbp1	0.291031914	4.642638827	4.0667754	0.00183218	0.033899	-1.90204
ENSMUSG00000032010//Usp2	0.291394896	7.172000117	4.584158689	0.000769249	0.022159	-1.25222
ENSMUSG00000030322//Mbd4	0.292047113	4.308954739	3.98825952	0.002096188	0.036061	-1.97616
ENSMUSG00000016984//Etaa1	0.292149977	4.436283406	3.702673133	0.003440149	0.045489	-2.50956
ENSMUSG00000042396//Rbm7	0.292354943	6.341623259	4.841267203	0.000506443	0.018123	-0.777
ENSMUSG00000057396//Zfp759	0.293266662	4.470091408	3.8047634	0.002878972	0.041621	-2.33387
ENSMUSG00000074909//Ranbp6	0.29370626	6.591263436	4.112647276	0.001694178	0.032784	-2.04886
ENSMUSG00000049164//Zfp518a	0.294055713	5.951540414	3.832158917	0.002745128	0.04047	-2.50127
ENSMUSG00000095567//Noc2l	0.294782016	5.087228981	4.124714063	0.001659706	0.032402	-1.87492
ENSMUSG00000028576//Ift74	0.294925426	5.614527286	3.824782222	0.002780521	0.040666	-2.47863
ENSMUSG00000021635//Rad17	0.295247198	5.280858261	3.89480254	0.00246273	0.038239	-2.31052
ENSMUSG00000074656//Eif2s2	0.297076895	6.371879244	3.749545814	0.003169653	0.043813	-2.6824
ENSMUSG00000027286//Lrrc57	0.29785354	5.041299037	4.380216497	0.001078549	0.026405	-1.42142
ENSMUSG00000028455//Stoml2	0.297907924	4.689484478	4.267656443	0.001302757	0.028652	-1.5583
ENSMUSG00000034640//Tiparp	0.298971699	4.664546511	4.029412313	0.001953212	0.035059	-1.97193
ENSMUSG00000068798//Rap1a	0.299256403	5.396390355	3.677495854	0.003595169	0.046374	-2.71476
ENSMUSG00000022019//Tdrd3	0.299754076	5.171605393	3.671551342	0.00363281	0.046652	-2.69362
ENSMUSG00000027109//Sp3	0.300199379	7.211390688	5.022257035	0.000379436	0.015886	-0.51418

ENSMUSG00000023075//Akirin1	0.301743679	5.714156545	4.963598243	0.000416446	0.016538	-0.51806
ENSMUSG00000025408//Ddit3	0.302151088	5.097755607	4.440687393	0.000975146	0.025325	-1.32554
ENSMUSG00000027905//Ddx20	0.303195708	5.146670462	4.737288953	0.000599058	0.019842	-0.82576
ENSMUSG00000021929//Kpna3	0.303213462	7.124354545	4.960665486	0.000418393	0.016538	-0.61352
ENSMUSG00000033773//Rpap2	0.303966204	4.885048649	4.010260633	0.002018445	0.035568	-2.04443
ENSMUSG00000071267//Zfp942	0.304708181	5.476297498	4.380100803	0.001078757	0.026405	-1.48289
ENSMUSG00000046311//Zfp62	0.306325609	6.794850463	4.644970059	0.000696263	0.021236	-1.13351
ENSMUSG00000026723//Trdmt1	0.306368585	4.241530429	4.075701057	0.001804439	0.033712	-1.80847
ENSMUSG00000025036//Sfxn2	0.306566287	4.528706641	3.866651783	0.002585714	0.039338	-2.23502
ENSMUSG00000053931//Cnn3	0.308804793	7.40807231	3.873380442	0.002555751	0.039193	-2.50654
ENSMUSG00000011148//Adssl1	0.308823881	3.853322436	3.735264653	0.00324966	0.044319	-2.32883
ENSMUSG00000026273//Mterf4	0.309432586	4.268181335	3.954488751	0.002221625	0.036421	-2.02771
ENSMUSG00000028218//Fam92a	0.310754895	5.573865619	4.066327256	0.001833585	0.033899	-2.04446
ENSMUSG00000036977//Anapc10	0.312479131	4.573603527	4.576669668	0.000778778	0.022292	-1.00399
ENSMUSG00000021428//Riok1	0.312860789	4.837652826	4.361502665	0.001112826	0.026544	-1.4209
ENSMUSG00000058093//Zfp729b	0.313152569	5.155588359	4.775707947	0.000562915	0.019215	-0.76215
ENSMUSG00000092417//Gpank1	0.31360219	3.647624723	3.761986915	0.003101615	0.043147	-2.23479
ENSMUSG00000026516//Nvl	0.314438035	5.833882404	3.885368872	0.002503254	0.038661	-2.39483
ENSMUSG00000039531//Zufsp	0.314872872	4.474583821	4.268352173	0.001301231	0.028652	-1.51719
ENSMUSG00000033960//Jcad	0.315074239	5.497842369	4.257806708	0.001324569	0.028876	-1.69859
ENSMUSG00000021477//Ctsl	0.316051612	7.027512289	4.015136101	0.002001627	0.035386	-2.24041
ENSMUSG00000024360//Etf1	0.317451317	6.960095431	5.115500445	0.000327583	0.014912	-0.35075
ENSMUSG00000038975//Rabggtb	0.317635548	5.835451976	4.114556528	0.001688675	0.032784	-1.98857
ENSMUSG00000028282//Casp8ap2	0.317919338	6.11527359	5.317559283	0.000239258	0.013114	0.025638
ENSMUSG00000004500//Zfp324	0.318595405	4.289090696	4.108674007	0.001705692	0.032784	-1.76006
ENSMUSG00000001774//Chordc1	0.319473842	6.020732448	5.10946427	0.000330701	0.014935	-0.30637
ENSMUSG00000020541//Tom1l1	0.319728589	4.335744211	3.711905886	0.003385044	0.045227	-2.47285
ENSMUSG00000041623//D11Wsu47e	0.321164088	4.275128095	4.268255397	0.001301443	0.028652	-1.47828
ENSMUSG00000011427//Zfp790	0.321606768	5.224775638	5.272459778	0.00025651	0.013429	0.049929
ENSMUSG00000030134//Rasgef1a	0.322719821	5.235433837	4.436182517	0.000982478	0.025325	-1.35345
ENSMUSG00000074264//Amy1	0.323081947	7.037909107	3.924767433	0.002338465	0.03731	-2.40145
ENSMUSG00000022462//Slc38a2	0.32507625	7.065259372	5.328824577	0.000235144	0.013065	-0.00639
ENSMUSG00000021149//Gtpbp4	0.325761689	5.797290718	4.757707749	0.000579554	0.01957	-0.87247
ENSMUSG00000021846//Peli2	0.325800473	5.060044778	3.971496754	0.002157505	0.036261	-2.14137
ENSMUSG00000029276//Glmn	0.326030144	6.764859733	5.59142616	0.000157737	0.011165	0.425596
ENSMUSG00000037031//Tspan15	0.328141154	4.517781417	3.649422051	0.003776543	0.047652	-2.62075
ENSMUSG00000032459//Mrps22	0.328305498	4.352897314	4.144511384	0.001604723	0.031793	-1.70989
ENSMUSG00000022951//Rcan1	0.328525317	5.35706079	5.298525768	0.000246384	0.013254	0.074398
ENSMUSG00000049300//Prmt6	0.329480284	4.311244385	3.607369879	0.004066041	0.049768	-2.65498
ENSMUSG00000029048//Rer1	0.329890946	5.828929564	5.553150432	0.000167088	0.011209	0.428244
ENSMUSG00000022474//Pmm1	0.329946743	6.331632762	5.08856432	0.000341744	0.015219	-0.36428
ENSMUSG00000035293//G2e3	0.33046215	5.082975799	3.639279144	0.003844368	0.048114	-2.73802
ENSMUSG00000043639//Rbm20	0.333444461	5.561958394	4.904211897	0.00045782	0.017483	-0.59989
ENSMUSG00000019970//Sgk1	0.33372246	8.747019826	4.729898029	0.000606287	0.019873	-1.04274
ENSMUSG00000056260//Lrif1	0.335822249	6.330539103	5.421627818	0.000203971	0.012241	0.177824
ENSMUSG00000030521//Mphosph10	0.335831038	5.111108406	5.063253862	0.000355642	0.015371	-0.27604
ENSMUSG00000034998//Foxn2	0.336948611	5.213832182	4.214113323	0.001426026	0.030106	-1.73674
ENSMUSG00000037260//Hgsnat	0.337274345	5.236067591	4.886691004	0.000470839	0.017707	-0.58717
ENSMUSG00000095325//Zfp870	0.337709639	4.254924332	4.33664224	0.001160135	0.026933	-1.35552
ENSMUSG00000041957//Pkp2	0.338623042	4.426332509	4.491556137	0.000896215	0.02418	-1.12199
ENSMUSG00000034401//Spata6	0.340193731	3.516569612	3.808218621	0.002861726	0.04147	-2.12261
ENSMUSG00000031577//Tti2	0.340459349	4.655574231	4.478697259	0.000915513	0.024286	-1.18672
ENSMUSG00000035840//Lysmd3	0.341878364	5.440438794	4.879193106	0.000476529	0.017794	-0.62693
ENSMUSG00000061244//Exoc5	0.341904573	6.303753748	5.521660638	0.000175222	0.011401	0.339466
ENSMUSG00000033849//B3galt2	0.343557845	7.157072757	5.374668695	0.000219157	0.012735	0.06436
ENSMUSG00000028140//Mrpl9	0.344013827	4.49144169	3.998957764	0.002058003	0.035955	-1.99323
ENSMUSG00000028345//Tex10	0.345033863	5.006402498	4.138358069	0.001621606	0.032047	-1.83826
ENSMUSG00000020674//Pxdn	0.346527344	5.683177762	4.208188377	0.001440396	0.030226	-1.8075
ENSMUSG00000021990//Spata13	0.347603223	3.458976376	3.71165764	0.003386514	0.045227	-2.28063
ENSMUSG00000040297//Suco	0.349127833	7.208422747	6.926802812	2.40E-05	0.004431	2.399413
ENSMUSG00000035161//Ints6	0.349511527	5.617730112	6.058125513	7.95E-05	0.008438	1.232891

ENSMUSG00000044375//BC027072	0.350668981	9.004415649	6.445416671	4.60E-05	0.006333	1.669923
ENSMUSG00000032413//Rasa2	0.35186386	6.090595069	4.106542627	0.001711901	0.032794	-2.02616
ENSMUSG00000034595//Ppp1r18	0.353056028	4.334641807	4.414978486	0.001017777	0.025666	-1.23583
ENSMUSG00000057895//Zfp105	0.353833589	3.902202321	3.819424603	0.002806523	0.040985	-2.18994
ENSMUSG00000033192//Lpcat2	0.354058761	5.237040523	5.059765002	0.000357604	0.015408	-0.29972
ENSMUSG00000070407//Hs3st3b1	0.354466504	6.981782038	6.505222996	4.24E-05	0.006027	1.805071
ENSMUSG00000032374//Plod2	0.356484877	4.933845194	5.261460367	0.000260915	0.013429	0.074973
ENSMUSG00000067219//Nipal1	0.358660129	4.300103905	4.145081461	0.001603168	0.031793	-1.69837
ENSMUSG00000068154//Insm1	0.361006469	5.176974116	5.6566535	0.000143061	0.010714	0.669217
ENSMUSG00000027722//Spata5	0.361646007	4.388646393	4.918810776	0.000447262	0.017308	-0.39258
ENSMUSG00000079065//BC005561	0.364568265	5.732903394	5.642471882	0.000146124	0.010758	0.579116
ENSMUSG00000019845//Tube1	0.364855964	5.22345113	5.996107157	8.69E-05	0.008727	1.187079
ENSMUSG00000071350//Setdb2	0.366071143	4.237966417	4.364506023	0.001107249	0.026536	-1.3039
ENSMUSG00000039661//Dusp26	0.368773372	5.293792036	5.684823745	0.000137178	0.01064	0.697374
ENSMUSG00000027284//Cdan1	0.36895165	5.997435912	4.2044695	0.001449493	0.03027	-1.84591
ENSMUSG00000023892//Zfp51	0.369542094	4.170450871	4.050543346	0.001883774	0.034562	-1.83809
ENSMUSG00000046010//Zfp830	0.370244577	4.489366129	3.795015993	0.002928208	0.04186	-2.35497
ENSMUSG00000018593//Sparc	0.370800146	7.647581646	3.730128228	0.003278942	0.044439	-2.77072
ENSMUSG00000033066//Gas7	0.37258185	7.732811837	7.207046509	1.66E-05	0.003755	2.771129
ENSMUSG00000012017//Scarf2	0.373331832	2.922822183	3.611859689	0.004034071	0.049535	-2.33211
ENSMUSG00000056870//Gulp1	0.376114444	5.475749797	4.145122764	0.001603056	0.031793	-1.89348
ENSMUSG00000008301//Phax	0.376402667	6.287006995	5.392422175	0.00021328	0.012632	0.133861
ENSMUSG00000023284//Zfp605	0.376734353	5.537290869	5.873964298	0.000103773	0.009642	0.960874
ENSMUSG00000025532//Crcp	0.379378658	4.580029212	4.368132996	0.001100553	0.026536	-1.36386
ENSMUSG00000018102//Hist1h2bc	0.382235474	6.054385238	4.621048043	0.000724066	0.021469	-1.12888
ENSMUSG00000050229//Pigm	0.383817452	5.856668271	6.370088362	5.11E-05	0.006829	1.674389
ENSMUSG00000054079//Utp18	0.384545271	4.782398023	4.870550711	0.000483178	0.0179	-0.54415
ENSMUSG00000074749//Kiz	0.384897437	5.316157565	5.099430031	0.000335955	0.015122	-0.2452
ENSMUSG00000056752//Dnah9	0.3856576	5.001224303	4.916030136	0.000449253	0.017346	-0.50354
ENSMUSG00000098176//Ccdc166	0.38631805	4.026079925	4.624187447	0.000720351	0.021402	-0.81581
ENSMUSG00000049232//Tigd2	0.38809466	5.369015721	5.823514187	0.000111738	0.009972	0.903471
ENSMUSG00000075054//Yae1d1	0.389068834	5.056435277	5.46023271	0.000192324	0.011934	0.375973
ENSMUSG00000025591//Tma16	0.389792035	4.291633106	4.791966157	0.000548318	0.018791	-0.58581
ENSMUSG00000063281//Zfp35	0.392175241	4.959097384	4.656732732	0.000683005	0.021116	-0.93378
ENSMUSG00000042354//Gnl3	0.393417011	4.686170515	5.292520724	0.000248679	0.013254	0.166089
ENSMUSG00000034379//Wdr5b	0.394876853	4.49687137	5.308296989	0.000242698	0.013219	0.225053
ENSMUSG00000039713//Plekhg5	0.395107481	4.05451501	4.635190946	0.00070749	0.021274	-0.80304
ENSMUSG00000058331//Zfp85	0.395979816	3.291262664	4.407063855	0.001031291	0.025794	-1.02639
ENSMUSG00000027715//Ccna2	0.396375296	5.095914337	4.09833828	0.001736025	0.033082	-1.92272
ENSMUSG00000035299//Mid1	0.39682505	4.794986892	5.276347148	0.000254973	0.013429	0.121499
ENSMUSG00000085795//Zfp703	0.397823111	3.967284822	3.880112882	0.002526131	0.038852	-2.09631
ENSMUSG00000036009//Mettl25	0.398816216	3.741437674	4.935288583	0.000435653	0.016934	-0.23521
ENSMUSG00000022193//Psmb5	0.398940761	2.875593909	3.654665197	0.003741966	0.047368	-2.24507
ENSMUSG00000031596//Slc7a2	0.399055275	6.34285788	4.477765094	0.000916929	0.024286	-1.39678
ENSMUSG00000028121//Bcar3	0.40061715	4.537039902	4.983444672	0.000403514	0.016349	-0.31297
ENSMUSG00000039756//Dnttip2	0.400804912	6.435463	6.137394236	7.09E-05	0.008056	1.284679
ENSMUSG00000024293//Esco1	0.401455789	6.502482924	6.2810715	5.79E-05	0.007449	1.495848
ENSMUSG00000025916//Ppp1r42	0.403864758	4.321371598	4.204805662	0.001448668	0.03027	-1.59808
ENSMUSG00000015994//Fnta	0.408208525	5.738930501	6.547759014	4.00E-05	0.006027	1.945265
ENSMUSG00000026771//Spopl	0.408865286	4.77922272	6.245213528	6.09E-05	0.007586	1.626297
ENSMUSG00000022453//Naga	0.409675352	4.281545769	5.262666116	0.000260428	0.013429	0.191477
ENSMUSG00000074794//Arrdc3	0.410137758	7.008281118	5.466698594	0.000190443	0.01186	0.217555
ENSMUSG00000029171//Pgm1	0.413146913	3.415745544	3.969007012	0.00216677	0.036261	-1.81536
ENSMUSG00000025747//Tyms	0.415106272	3.393664902	4.48209475	0.000910372	0.024286	-0.92097
ENSMUSG00000005124//Wisp1	0.422399469	5.93619319	6.478048447	4.40E-05	0.006163	1.825415
ENSMUSG00000036430//Tbcc	0.423289171	4.486858989	5.238665528	0.0002703	0.013607	0.114354
ENSMUSG00000027848//Olfml3	0.425008115	4.161476457	3.915523378	0.002376091	0.03755	-2.07471
ENSMUSG00000024074//Crim1	0.425798742	6.883362211	4.548587273	0.000815629	0.022711	-1.30235
ENSMUSG00000053279//Aldh1a1	0.426955064	6.64483886	4.911319275	0.000452647	0.017402	-0.67643
ENSMUSG00000053600//Zfp472	0.427961791	4.401841652	6.358349894	5.19E-05	0.006889	1.856099
ENSMUSG00000030533//Unc45a	0.429283929	6.080795563	4.989771629	0.000399482	0.016273	-0.50933

ENSMUSG00000053399//Adamts18	0.433314791	4.537882492	3.630575363	0.00390357	0.048523	-2.65845
ENSMUSG00000057835//Zfp119a	0.435504699	3.606044805	4.635001635	0.000707709	0.021274	-0.7081
ENSMUSG00000018217//Pmp22	0.436785749	5.071375367	3.968995115	0.002166814	0.036261	-2.14757
ENSMUSG00000096910//Zfp955b	0.438324638	4.814330031	5.528949101	0.000173302	0.011391	0.522692
ENSMUSG00000091402//Rd3l	0.443478187	6.253298067	8.021719962	6.06E-06	0.001807	3.898923
ENSMUSG00000018196//Glrx2	0.443894712	5.780637593	7.623623076	9.83E-06	0.002669	3.4232
ENSMUSG00000006678//Pola1	0.445995925	4.037948518	4.589819139	0.000762128	0.022033	-0.87667
ENSMUSG00000023249//Parp3	0.448621412	3.47298537	3.97151417	0.00215744	0.036261	-1.82409
ENSMUSG00000046152//Fut10	0.451230774	3.866545094	5.833527669	0.000110107	0.009964	1.169329
ENSMUSG00000033705//Stard9	0.453260633	5.114300619	4.478899886	0.000915205	0.024286	-1.26215
ENSMUSG00000039831//Arhgap29	0.453270627	4.899814167	5.55985707	0.000165408	0.011209	0.557924
ENSMUSG00000023186//Vwa5a	0.457334879	4.507636149	3.76485005	0.003086171	0.043105	-2.41233
ENSMUSG00000097195//Snhg5	0.458452786	3.620215348	5.596912738	0.000156443	0.011165	0.852867
ENSMUSG00000050930//Map10	0.461251117	5.375396624	6.511703315	4.20E-05	0.006027	1.933568
ENSMUSG00000067786//Nnat	0.4612828	4.80179563	4.550639887	0.000812875	0.02267	-1.08891
ENSMUSG00000062937//Mtap	0.46313265	3.118883871	3.795695906	0.002924746	0.04186	-2.05236
ENSMUSG00000062743//Zfp677	0.463829163	3.594023877	4.346447245	0.00114123	0.026814	-1.19811
ENSMUSG00000002797//Ggct	0.466544525	3.396984686	3.934529013	0.002299403	0.037057	-1.87176
ENSMUSG00000078773//Rad54b	0.467421339	3.036481033	4.319051176	0.001194878	0.027346	-1.12062
ENSMUSG00000057132//Rpgrip1	0.469203187	9.688976894	6.159257799	6.88E-05	0.00786	1.234821
ENSMUSG00000021069//Pygl	0.475000802	5.09538663	5.450276215	0.000195259	0.011988	0.354311
ENSMUSG00000026478//Lamc1	0.477924977	5.099476568	6.770153583	2.96E-05	0.005135	2.339461
ENSMUSG00000019232//Etnppl	0.480195037	4.548792502	3.910667283	0.002396108	0.037701	-2.16068
ENSMUSG00000023150//Ivns1abp	0.484311914	8.467559164	4.611610931	0.000735352	0.021664	-1.23991
ENSMUSG00000039985//Fam60a	0.492319526	2.309687668	3.891265381	0.002477844	0.038371	-1.6979
ENSMUSG00000026659//Dusp12	0.492498923	2.219191306	3.737330459	0.003237959	0.044229	-1.94634
ENSMUSG00000078866//Zfp970	0.495215718	4.656560776	5.479544416	0.000186764	0.011757	0.470769
ENSMUSG00000069833//Ahnak	0.499131285	6.689502592	4.983200121	0.000403671	0.016349	-0.55858
ENSMUSG00000050323//Ndufaf6	0.505455338	2.758048956	4.597973952	0.00075199	0.021849	-0.58587
ENSMUSG00000005667//Mthfd2	0.505487917	2.688540088	3.879665868	0.002528086	0.038852	-1.80469
ENSMUSG00000001672//Marveld3	0.508416162	3.831892579	5.488545981	0.000184231	0.011749	0.640188
ENSMUSG00000026726//Cubn	0.513306498	3.06204832	4.354250645	0.001126414	0.026598	-1.06589
ENSMUSG00000063171//Rps4l	0.514299315	4.208673781	6.509438214	4.21E-05	0.006027	2.109413
ENSMUSG00000092274//Neat1	0.521391317	5.022606874	5.336710284	0.000232308	0.013065	0.183067
ENSMUSG00000068114//Ccdc134	0.523826448	3.364195219	5.452675024	0.000194547	0.011987	0.6788
ENSMUSG00000079465//Col4a3	0.525772126	6.740663631	4.280129339	0.001275674	0.028453	-1.7618
ENSMUSG00000060572//Mfap2	0.526686844	3.330284749	4.4222387	0.001005544	0.025431	-1.00914
ENSMUSG00000055150//Zfp78	0.529167895	3.848482262	5.313676268	0.000240694	0.013151	0.358719
ENSMUSG00000067158//Col4a4	0.535064286	6.112275004	7.159086343	1.77E-05	0.00376	2.773522
ENSMUSG00000025940//Tmem70	0.537497593	4.022384366	6.198716528	6.50E-05	0.007692	1.689404
ENSMUSG00000026728//Vim	0.539219123	7.850436828	5.964550738	9.10E-05	0.00897	0.970015
ENSMUSG00000045275//Lca5l	0.540916533	3.504321798	3.886421391	0.002498698	0.038629	-1.98136
ENSMUSG00000027133//Nop10	0.545649917	3.304304593	5.436851137	0.000199291	0.01215	0.666093
ENSMUSG00000055435//Maf	0.550276701	5.603622119	3.949165845	0.002242096	0.036544	-2.25569
ENSMUSG00000036721//Zscan12	0.551908474	4.072776554	6.511858441	4.20E-05	0.006027	2.137076
ENSMUSG00000030532//Hddc3	0.556396425	3.852811005	4.536060821	0.000832653	0.023108	-0.92942
ENSMUSG00000090812//Samd15	0.557927512	2.367844549	3.621464509	0.003966547	0.048948	-2.18432
ENSMUSG00000047881//Rell1	0.558074771	2.47582618	4.553651061	0.000808851	0.022631	-0.60032
ENSMUSG00000022122//Ednrb	0.559775496	3.575949608	6.193234622	6.55E-05	0.007692	1.765697
ENSMUSG00000029217//Tec	0.559986644	2.502996717	4.07935262	0.001793216	0.033601	-1.41462
ENSMUSG00000044362//Ccdc89	0.560293904	2.880762167	4.387044587	0.001066318	0.02628	-0.96962
ENSMUSG00000048485//Zbtb8b	0.56492726	3.662044493	5.750763268	0.00012439	0.010078	1.082687
ENSMUSG00000036598//Ccdc113	0.565296944	3.949470341	5.778647334	0.000119369	0.010068	1.069128
ENSMUSG00000026511//Srp9	0.565690411	4.623725745	5.327293394	0.000235698	0.013065	0.232922
ENSMUSG00000051499//Zfp786	0.566418132	2.762256926	3.907741525	0.002408253	0.037731	-1.77244
ENSMUSG00000022471//Xrcc6	0.570953276	5.060965211	8.455735601	3.64E-06	0.001218	4.549415
ENSMUSG00000045975//C2cd2	0.570956733	4.836448918	8.010949664	6.14E-06	0.001807	4.030821
ENSMUSG00000021583//Erap1	0.572537316	4.514608177	5.931411503	9.54E-05	0.009264	1.198765
ENSMUSG00000031609//Sap30	0.577533547	3.83193785	7.059589152	2.01E-05	0.003978	2.945923
ENSMUSG00000031578//Mak16	0.578261504	4.209000845	6.096802451	7.52E-05	0.008416	1.50332
ENSMUSG00000078851//Hist3h2a	0.585936857	4.941461379	7.172501742	1.74E-05	0.00376	2.919546

ENSMUSG00000038539//Atf5	0.587305843	4.167463204	4.691958756	0.000644868	0.020597	-0.7297
ENSMUSG00000018672//Copz2	0.58923149	1.775655652	4.168373862	0.001540964	0.031205	-1.10751
ENSMUSG00000029452//Tmem116	0.597018058	2.813819621	3.610365314	0.004044683	0.04963	-2.30905
ENSMUSG00000027695//Pld1	0.598061873	1.975219396	3.692051915	0.003504687	0.046011	-1.9708
ENSMUSG00000085156//Snhg15	0.601792168	1.731169695	4.152432563	0.001583259	0.031547	-1.12582
ENSMUSG00000028447//Dctn3	0.601926426	3.937326404	7.266417878	1.54E-05	0.00367	3.206587
ENSMUSG00000031502//Col4a1	0.606792161	8.074792074	4.507907843	0.000872289	0.023756	-1.4104
ENSMUSG00000022404//Slc25a17	0.608145654	4.299903608	7.870414289	7.27E-06	0.002004	3.933196
ENSMUSG00000002910//Arrdc2	0.610327567	3.55106365	4.717183402	0.000618938	0.020082	-0.55778
ENSMUSG00000024421//Lama3	0.611579326	4.070526227	6.078575971	7.72E-05	0.008416	1.501482
ENSMUSG00000032715//Trib3	0.612069048	2.163135076	3.895950857	0.002457844	0.038198	-1.65695
ENSMUSG00000027082//Tfpi	0.612081567	1.250138087	3.658501152	0.003716877	0.047277	-1.87654
ENSMUSG00000035805//Mlc1	0.613501522	5.639878117	8.668593643	2.86E-06	0.001035	4.740942
ENSMUSG00000022912//Pros1	0.614852913	4.039498703	6.919254845	2.42E-05	0.004431	2.717383
ENSMUSG00000063383//Zfp947	0.619752267	1.999422338	4.438952863	0.000977962	0.025325	-0.6951
ENSMUSG00000015133//Lrrk1	0.626176957	3.35869659	7.177500173	1.73E-05	0.00376	3.185244
ENSMUSG00000035696//Rnf38	0.627288038	5.899389726	9.556359597	1.09E-06	0.000513	5.735006
ENSMUSG00000070047//Fat1	0.629023691	6.703991196	5.366616445	0.00022188	0.012851	0.069092
ENSMUSG00000026946//Nmi	0.631143231	1.625304392	4.030330044	0.001950141	0.03504	-1.313
ENSMUSG00000028763//Hspg2	0.631499773	5.797809166	4.73846742	0.000597913	0.019842	-0.90509
ENSMUSG00000008393//Carhsp1	0.638244589	4.730184727	4.568319211	0.00078955	0.022502	-1.04631
ENSMUSG00000000325//Arvcf	0.641650236	6.347812296	4.359968272	0.001115687	0.026544	-1.60127
ENSMUSG00000044447//Dock5	0.649162038	5.486070166	5.801815141	0.000115362	0.009972	0.855395
ENSMUSG00000021575//Ahrr	0.652390029	6.124691152	9.645567622	9.95E-07	0.000494	5.817165
ENSMUSG00000057060//Slc35f3	0.660182869	2.559183785	4.814265907	0.000528942	0.018492	-0.18482
ENSMUSG00000041954//Tnfrsf18	0.66921591	2.927505795	3.709038353	0.00340206	0.045327	-2.16093
ENSMUSG00000027111//Itga6	0.669393905	5.361318168	5.114990231	0.000327845	0.014912	-0.22548
ENSMUSG00000026879//Gsn	0.670485841	4.531663586	5.050214188	0.000363034	0.015536	-0.20173
ENSMUSG00000030606//Hapln3	0.670900445	1.391894322	4.34148905	0.001150749	0.026894	-0.74599
ENSMUSG00000024659//Anxa1	0.673182309	5.671786991	7.516894677	1.12E-05	0.002966	3.293213
ENSMUSG00000028295//Smim8	0.675320729	3.049647137	4.888998867	0.000469102	0.017707	-0.16413
ENSMUSG00000039457//Ppl	0.677797806	4.533775177	5.553308581	0.000167048	0.011209	0.609003
ENSMUSG00000015312//Gadd45b	0.677983911	2.151081844	4.297951405	0.001237991	0.028008	-0.96354
ENSMUSG00000032495//Lrrc2	0.684912485	4.995619303	10.6876964	3.54E-07	0.000236	7.002548
ENSMUSG00000051627//Hist1h1e	0.692284365	1.908686439	3.812047306	0.00284274	0.041276	-1.74717
ENSMUSG00000023019//Gpd1	0.698082039	2.407672731	4.038958981	0.001921517	0.034818	-1.46321
ENSMUSG00000040562//Gstm2	0.703357458	2.645524162	3.979179771	0.002129173	0.036127	-1.62085
ENSMUSG00000027820//Mme	0.705031747	4.356725273	4.820722066	0.000523468	0.018402	-0.55017
ENSMUSG00000022665//Ccdc80	0.706155292	2.388943983	5.044354816	0.000366409	0.015605	0.223057
ENSMUSG00000035539//Ccdc180	0.714371487	3.458470317	4.480539693	0.000912721	0.024286	-0.93795
ENSMUSG00000023236//Scg5	0.718373993	7.599931929	13.57404338	2.97E-08	5.27E-05	9.459529
ENSMUSG00000028173//Wls	0.720509453	4.485833875	5.648350086	0.000144846	0.010755	0.766846
ENSMUSG00000044881//Coa4	0.727933493	1.950560546	4.499504339	0.0008845	0.024005	-0.58452
ENSMUSG00000042406//Atf4	0.731267981	7.681765973	11.40428038	1.82E-07	0.000176	7.555355
ENSMUSG00000023043//Krt18	0.731587862	5.281857897	8.692040266	2.79E-06	0.00103	4.805938
ENSMUSG00000032060//Cryab	0.740047649	10.216387	3.909027175	0.002402908	0.037715	-2.4972
ENSMUSG00000027907//S100a11	0.742704426	2.833754565	4.03683672	0.001928516	0.034868	-1.56331
ENSMUSG00000019853//Hebp2	0.749439418	2.54398829	4.372383782	0.001092759	0.026536	-0.9208
ENSMUSG00000047786//Lix1	0.753443086	4.255700883	6.275531756	5.83E-05	0.007452	1.760308
ENSMUSG00000036964//Trim17	0.765557562	3.938808141	8.331642915	4.21E-06	0.001379	4.558281
ENSMUSG00000058135//Gstm1	0.768784881	5.753955047	6.26149012	5.95E-05	0.007545	1.52373
ENSMUSG00000011179//Odc1	0.773996332	6.752329287	6.931023025	2.39E-05	0.004431	2.42191
ENSMUSG00000031521//Aga	0.776164217	2.310992261	6.535053952	4.07E-05	0.006027	2.466841
ENSMUSG00000024041//Cryaa	0.783296106	12.17846969	4.134906423	0.001631158	0.032097	-2.12225
ENSMUSG00000059434//Gckr	0.784020082	5.255841888	6.884325285	2.54E-05	0.004545	2.479559
ENSMUSG00000031665//Sall1	0.784667389	2.942570231	4.409652301	0.001026851	0.025794	-0.94468
ENSMUSG00000044518//Foxe3	0.787569524	2.252670657	5.194778648	0.00028939	0.014131	0.48824
ENSMUSG00000005087//Cd44	0.788888106	5.510313282	5.770855242	0.00012075	0.010078	0.804522
ENSMUSG00000002603//Tgfb1	0.791390986	1.591854498	4.70472595	0.000631604	0.020285	-0.18322
ENSMUSG00000031562//Dctd	0.79207651	0.943725276	4.130700701	0.001642875	0.032291	-1.0228
ENSMUSG00000041324//Inhba	0.799917426	2.196981988	4.876676045	0.000478455	0.017794	-0.01113

ENSMUSG00000000303//Cdh1	0.800696802	3.315548891	6.927418688	2.40E-05	0.004431	2.854858
ENSMUSG00000095241//Gm5478	0.802872342	1.647701496	3.605741107	0.004077702	0.049789	-2.05021
ENSMUSG00000053398//Phgdh	0.812709308	3.759383158	6.441081913	4.63E-05	0.006333	2.092505
ENSMUSG00000021943//Gdf10	0.820797588	1.141001388	4.028180888	0.001957339	0.035097	-1.22635
ENSMUSG00000035778//Ggta1	0.824179087	2.241481068	5.388390727	0.0002146	0.012632	0.79347
ENSMUSG00000072572//Slc39a2	0.826155395	1.774553455	5.592753495	0.000157423	0.011165	1.177051
ENSMUSG00000044726//Erich5	0.826559913	5.465272425	6.425256998	4.73E-05	0.006423	1.796736
ENSMUSG00000057143//Trim12c	0.826779031	2.460655575	7.077221062	1.97E-05	0.003932	3.180866
ENSMUSG00000037681//Esyt3	0.827460396	2.363382571	5.852356445	0.000107108	0.009821	1.478284
ENSMUSG00000029082//Bst1	0.831209695	3.509415209	5.172082035	0.000299817	0.01439	0.200934
ENSMUSG00000048572//Tmem252	0.834849044	0.663000641	3.687523912	0.003532579	0.046149	-1.72182
ENSMUSG00000041731//Pgm5	0.855005623	2.465137926	4.964395031	0.000415918	0.016538	0.079108
ENSMUSG00000005397//Nid1	0.859309502	6.579023206	6.291372713	5.70E-05	0.007397	1.507293
ENSMUSG00000060036//Rpl3	0.859641489	7.95452096	10.88126893	2.94E-07	0.000209	7.042121
ENSMUSG00000036256//Igfbp7	0.880266162	6.30716255	8.458729135	3.63E-06	0.001218	4.437325
ENSMUSG00000042254//Cilp	0.881752971	0.920665284	3.776327969	0.003025054	0.042593	-1.61374
ENSMUSG00000022382//Wnt7b	0.882522152	4.852126267	4.254424241	0.001332147	0.029005	-1.60966
ENSMUSG00000030717//Nupr1	0.883785499	5.624928867	6.643523749	3.51E-05	0.005799	2.095001
ENSMUSG00000006546//Cryba2	0.894015663	8.865564357	3.774096486	0.003036837	0.042725	-2.71746
ENSMUSG00000056427//Slit3	0.894971498	2.813303002	5.376277649	0.000218618	0.012735	0.668528
ENSMUSG00000034459//Ifit1	0.903137072	1.907343685	5.850508105	0.000107399	0.009821	1.542331
ENSMUSG00000031548//Sfrp1	0.903530003	6.282320123	4.669654821	0.000668747	0.021012	-1.06345
ENSMUSG00000072494//Ppp1r3e	0.908113917	1.581104038	5.890629225	0.000101277	0.009512	1.638026
ENSMUSG00000031004//Mki67	0.911122588	0.713686535	4.047951866	0.001892149	0.034643	-1.12684
ENSMUSG00000090053//Palm2	0.925822168	3.136466072	3.830922493	0.002751027	0.04047	-1.99422
ENSMUSG00000058385//Hist1h2bg	0.929741848	0.720078339	5.037851061	0.000370193	0.015688	0.445222
ENSMUSG00000024076//Vit	0.933408251	5.777466267	6.187924684	6.60E-05	0.007698	1.411602
ENSMUSG00000047935//Sox1ot	0.9356163	3.405629702	3.955961428	0.002215995	0.036419	-1.83601
ENSMUSG00000044350//Lacc1	0.938903053	2.58421206	5.026200305	0.000377076	0.015864	0.155595
ENSMUSG00000037661//Gpr160	0.941029098	1.901251308	3.960205507	0.002199855	0.036368	-1.48921
ENSMUSG00000053198//Prx	0.946945596	2.193976241	5.109028415	0.000330928	0.014935	0.362903
ENSMUSG00000025229//Pitx3	0.953293159	1.943543591	4.560803042	0.000799379	0.022612	-0.48136
ENSMUSG00000099930//LOC105245043	0.953602817	1.72968493	3.853537606	0.002645162	0.039823	-1.63753
ENSMUSG00000099583//Hist1h3d	0.967157258	0.074299382	3.993716918	0.002076618	0.035974	-1.14678
ENSMUSG00000034333//Zbed4	0.980159983	3.914207571	9.367359716	1.33E-06	0.000551	5.751752
ENSMUSG00000036586//Grifin	0.990223222	5.183275738	3.898193538	0.002448331	0.038141	-2.29028
ENSMUSG00000064288//Hist1h4k	0.99681269	1.041055179	4.214250916	0.001425694	0.030106	-0.89922
ENSMUSG00000049908//Gja8	1.005817413	4.462073245	5.635851508	0.000147577	0.010774	0.751512
ENSMUSG00000067399//Trim43c	1.008040591	2.152452719	5.51930394	0.000175847	0.011401	1.010042
ENSMUSG00000048550//Thnsl1	1.015812089	3.760172969	10.14225547	6.01E-07	0.000348	6.586971
ENSMUSG00000027995//Tlr2	1.017340111	-0.009982067	3.880759292	0.002523305	0.038852	-1.32254
ENSMUSG00000028718//Stil	1.02260123	0.59705887	5.263392843	0.000260135	0.013429	0.787749
ENSMUSG00000028871//Rspo1	1.031354213	2.754837592	6.336567337	5.35E-05	0.006995	2.121479
ENSMUSG00000041482//Piezo2	1.036159206	1.049397919	3.920645354	0.002355167	0.037432	-1.39175
ENSMUSG00000027875//Hmgcs2	1.044937398	3.739598999	7.217569641	1.64E-05	0.003753	3.174851
ENSMUSG00000054252//Fgfr3	1.049402385	3.805600764	6.557819414	3.94E-05	0.006027	2.251515
ENSMUSG00000026628//Atf3	1.052373571	2.68139883	9.512443993	1.14E-06	0.000523	6.013668
ENSMUSG00000069270//Hist1h2ac	1.055006195	1.346481844	3.996922448	0.002065212	0.035955	-1.31639
ENSMUSG00000096014//Sox1	1.05732827	2.498159631	4.357498126	0.001120308	0.026556	-0.93625
ENSMUSG00000052912//Smarca5-ps	1.058336597	3.001091826	7.219536941	1.64E-05	0.003753	3.296462
ENSMUSG00000051367//Six1	1.063923049	0.566644665	5.085694956	0.000343291	0.015219	0.525255
ENSMUSG00000006403//Adamts4	1.065209995	1.14457343	5.716469135	0.000130874	0.010336	1.422488
ENSMUSG00000069132//Nxph2	1.078561039	0.627957304	5.71669535	0.00013083	0.010336	1.437834
ENSMUSG00000022766//Serpind1	1.08600648	1.479996216	4.765822382	0.00057199	0.019487	-0.06674
ENSMUSG00000030093//Wnt7a	1.08955172	2.514204631	5.325446409	0.00023637	0.013065	0.645913
ENSMUSG00000091405//Hist2h4	1.090238221	2.964711778	9.954519985	7.26E-07	0.000382	6.455727
ENSMUSG00000078503//Zfp990	1.096322034	0.833252766	5.557997942	0.000165872	0.011209	1.209306
ENSMUSG00000038541//Srd5a2	1.109764381	2.224033461	6.500172113	4.27E-05	0.006027	2.429566
ENSMUSG00000031103//Elf4	1.122368097	0.394393817	3.684942711	0.003548581	0.046257	-1.68761
ENSMUSG00000078502//Gm13212	1.124557517	-0.81686592	3.707325722	0.003412265	0.045327	-1.55727
ENSMUSG00000042200//Cdrt4	1.139654514	-0.735404899	3.731658052	0.003270192	0.044423	-1.52126

ENSMUSG00000005251//Ripk4	1.141902514	2.299506632	8.300167698	4.36E-06	0.001404	4.701711
ENSMUSG00000022686//B3gnt5	1.170430256	4.876309936	4.266574324	0.001305135	0.028668	-1.59253
ENSMUSG00000041653//Pnpla3	1.178227521	5.909190736	21.58840322	2.07E-10	1.20E-06	14.53822
ENSMUSG00000048582//Gja3	1.211043992	4.407815869	5.007562377	0.000388368	0.016028	-0.24917
ENSMUSG00000071478//Hist1h2ad	1.219391847	-0.425322306	3.85175885	0.002653334	0.039823	-1.34309
ENSMUSG00000056880//Gadl1	1.24056877	-0.576557833	4.451033427	0.000958521	0.025158	-0.42055
ENSMUSG00000042717//Ppp1r3a	1.271566861	0.741978065	4.662263095	0.000676864	0.021071	-0.13717
ENSMUSG00000031877//Ces2g	1.273262977	-0.011744806	5.522757082	0.000174932	0.011401	1.142039
ENSMUSG00000031963//Bmper	1.28322521	0.901945784	6.58251251	3.81E-05	0.006027	2.607321
ENSMUSG00000089789//Rdh1	1.311081132	0.701595151	5.587293272	0.00015872	0.011165	1.254653
ENSMUSG00000030898//Cckbr	1.312940246	3.353955978	11.80317586	1.28E-07	0.000148	8.172691
ENSMUSG00000050700//Emilin3	1.315600223	0.663700066	5.320715765	0.000238098	0.013092	0.869864
ENSMUSG00000017446//C1qtnf1	1.32305383	0.688664276	4.423919443	0.001002734	0.025431	-0.51082
ENSMUSG00000000958//Slc7a7	1.343434719	0.076682399	3.9320529	0.002309247	0.037065	-1.24663
ENSMUSG00000047143//Dmrta2	1.356500875	1.597022805	5.666878161	0.000140895	0.010714	1.309226
ENSMUSG00000038086//Hspb2	1.394945001	0.334256026	4.25159873	0.001338512	0.029071	-0.75342
ENSMUSG00000042842//Serpinb6b	1.630176065	1.37380806	3.662802031	0.003688952	0.047129	-1.89408
ENSMUSG00000068614//Actc1	1.650109036	2.705134845	11.27880828	2.04E-07	0.000185	7.700729
ENSMUSG00000009628//Tex15	1.716390108	2.511559518	7.408699854	1.29E-05	0.003193	3.60458
ENSMUSG00000097677//Gm26611	1.763441035	-1.012646505	4.709278297	0.000626944	0.020247	-0.0793
ENSMUSG00000026950//Neb	1.776521948	2.032476232	11.69592595	1.40E-07	0.000148	7.960347
ENSMUSG00000060044//Tmem26	1.821529124	0.440846734	7.501698418	1.14E-05	0.002966	3.647123
ENSMUSG00000043424//Eif3j2	1.888308842	1.420456291	7.507287581	1.14E-05	0.002966	3.77564
ENSMUSG00000085008//Dbhos	1.94644362	2.027491603	13.87536047	2.36E-08	5.13E-05	9.536255
ENSMUSG00000055976//Cldn23	1.96976363	1.391371666	6.35132807	5.24E-05	0.006904	2.298615
ENSMUSG00000042641//Rgsl1	1.983845408	-0.442276547	5.297177922	0.000246897	0.013254	0.793672
ENSMUSG00000026601//Axdnd1	1.994600742	-0.673736136	6.900635544	2.48E-05	0.004494	2.677174
ENSMUSG00000066800//Rnasel	2.034008366	1.167167795	11.70400183	1.39E-07	0.000148	7.666781
ENSMUSG00000067455//Hist1h4j	2.150597968	-1.766893766	4.804960103	0.000536939	0.018562	-0.07207
ENSMUSG00000097891//Gm3650	2.431242741	-0.63585173	6.112604742	7.35E-05	0.008292	1.801259
ENSMUSG00000022431//Ribc2	2.815504301	-0.537716671	9.287366382	1.45E-06	0.000586	4.884851
ENSMUSG00000090272//Mndal	4.112835016	-2.241062607	8.108525526	5.46E-06	0.001666	2.844769
ENSMUSG00000050141//Fam205c	4.855082406	-0.802574699	12.41370046	7.57E-08	0.00011	6.496238
ENSMUSG00000068457//Uty	6.325894467	2.659023054	4.400441446	0.001042743	0.025994	-0.89816
ENSMUSG00000069049//Eif2s3y	6.462730032	3.810877031	4.379932529	0.00107906	0.026405	-1.18802
ENSMUSG00000069045//Ddx3y	6.937688103	3.278623091	4.285906169	0.00126333	0.02832	-1.23183
ENSMUSG00000056673//Kdm5d	7.315245958	2.48858648	4.170024841	0.001536651	0.031191	-1.25493

Gene Ontology Terms- Downregulated Terms/Processes in the KO

source	term_name	term_id	adjusted_p_value
GO:MF	protein binding	GO:0005515	3.12E-26
GO:MF	inorganic cation transmembrane transporter activity	GO:0022890	3.34E-18
GO:MF	cation transmembrane transporter activity	GO:0008324	4.97E-18
GO:MF	metal ion transmembrane transporter activity	GO:0046873	6.26E-18
GO:MF	transmembrane transporter activity	GO:0022857	4.61E-15
GO:MF	inorganic molecular entity transmembrane transporter activity	GO:0015318	1.48E-14
GO:MF	transporter activity	GO:0005215	2.88E-14
GO:MF	ion transmembrane transporter activity	GO:0015075	6.41E-14
GO:MF	binding	GO:0005488	1.13E-13
GO:MF	cation channel activity	GO:0005261	8.27E-13
GO:MF	gated channel activity	GO:0022836	8.27E-13
GO:MF	voltage-gated cation channel activity	GO:0022843	1.53E-12
GO:MF	voltage-gated ion channel activity	GO:0005244	2.83E-11
GO:MF	voltage-gated channel activity	GO:0022832	3.30E-11
GO:MF	monovalent inorganic cation transmembrane transporter activity	GO:0015077	9.14E-11
GO:MF	ion channel activity	GO:0005216	1.43E-10
GO:MF	channel activity	GO:0015267	1.18E-09
GO:MF	passive transmembrane transporter activity	GO:0022803	1.18E-09
GO:MF	glutamate receptor binding	GO:0035254	1.48E-08
GO:MF	cytoskeletal protein binding	GO:0008092	2.25E-08
GO:MF	potassium ion transmembrane transporter activity	GO:0015079	4.95E-08
GO:MF	calcium ion transmembrane transporter activity	GO:0015085	6.64E-08
GO:MF	sodium ion transmembrane transporter activity	GO:0015081	1.06E-07
GO:MF	calcium ion binding	GO:0005509	4.87E-07
GO:MF	active ion transmembrane transporter activity	GO:0022853	7.47E-07
GO:MF	calmodulin binding	GO:0005516	2.00E-06
GO:MF	divalent inorganic cation transmembrane transporter activity	GO:0072509	4.57E-06
GO:MF	ion binding	GO:0043167	6.03E-06
GO:MF	active transmembrane transporter activity	GO:0022804	1.38E-05
GO:MF	voltage-gated calcium channel activity	GO:0005245	2.05E-05
GO:MF	voltage-gated potassium channel activity	GO:0005249	7.27E-05
GO:MF	channel regulator activity	GO:0016247	0.000135881
GO:MF	carbohydrate derivative binding	GO:0097367	0.000155296
GO:MF	ion channel binding	GO:0044325	0.00017055
GO:MF	calcium channel activity	GO:0005262	0.000211577
GO:MF	anion binding	GO:0043168	0.000232152
GO:MF	small molecule binding	GO:0036094	0.000241656
GO:MF	potassium channel activity	GO:0005267	0.000346508
GO:MF	kinase binding	GO:0019900	0.000402792
GO:MF	transmembrane receptor protein kinase activity	GO:0019199	0.000457699
GO:MF	PDZ domain binding	GO:0030165	0.000514356
GO:MF	protein kinase binding	GO:0019901	0.000864029
GO:MF	transmembrane receptor protein tyrosine kinase activity	GO:0004714	0.001072345
GO:MF	phosphotransferase activity, alcohol group as acceptor	GO:0016773	0.001148457
GO:MF	glutamate receptor activity	GO:0008066	0.00116782
GO:MF	ionotropic glutamate receptor binding	GO:0035255	0.001293243
GO:MF	cadherin binding	GO:0045296	0.001490939
GO:MF	microtubule binding	GO:0008017	0.001807384

GO:MF	protein-containing complex binding	GO:0044877	0.001895597
GO:MF	neurotrophin receptor activity	GO:0005030	0.003541038
GO:MF	cell adhesion molecule binding	GO:0050839	0.004004859
GO:MF	ribonucleotide binding	GO:0032553	0.004185666
GO:MF	purine ribonucleotide binding	GO:0032555	0.004793608
GO:MF	protein kinase activity	GO:0004672	0.005341411
GO:MF	kinase activity	GO:0016301	0.00559167
GO:MF	postsynaptic neurotransmitter receptor activity	GO:0098960	0.005903393
GO:MF	purine nucleotide binding	GO:0017076	0.006462009
GO:MF	nucleoside phosphate binding	GO:1901265	0.00665284
GO:MF	nucleotide binding	GO:0000166	0.00665284
GO:MF	purine ribonucleoside triphosphate binding	GO:0035639	0.006926168
GO:MF	structural constituent of postsynapse	GO:0099186	0.007420592
GO:MF	identical protein binding	GO:0042802	0.00912278
GO:MF	ATP binding	GO:0005524	0.010303432
GO:MF	vascular endothelial growth factor-activated receptor activity	GO:0005021	0.010336887
GO:MF	structural constituent of synapse	GO:0098918	0.010395744
GO:MF	amino acid:cation symporter activity	GO:0005416	0.010812172
GO:MF	symporter activity	GO:0015293	0.011216009
GO:MF	transmitter-gated ion channel activity	GO:0022824	0.012526524
GO:MF	transmitter-gated channel activity	GO:0022835	0.012526524
GO:MF	enzyme binding	GO:0019899	0.01292724
GO:MF	kinesin binding	GO:0019894	0.013411671
GO:MF	syntaxin-1 binding	GO:0017075	0.013625517
GO:MF	ligand-gated cation channel activity	GO:0099094	0.016465351
GO:MF	ATPase-coupled ion transmembrane transporter activity	GO:0042625	0.01702862
GO:MF	adenyl ribonucleotide binding	GO:0032559	0.017208712
GO:MF	molecular adaptor activity	GO:0060090	0.018060325
GO:MF	ion transmembrane transporter activity, phosphorylative mechanism	GO:0015662	0.018495629
GO:MF	cation binding	GO:0043169	0.019772732
GO:MF	adenyl nucleotide binding	GO:0030554	0.022945361
GO:MF	ligand-gated ion channel activity	GO:0015276	0.023513752
GO:MF	calcium-dependent protein binding	GO:0048306	0.0253972
GO:MF	protein-macromolecule adaptor activity	GO:0030674	0.025828694
GO:MF	ATPase-coupled cation transmembrane transporter activity	GO:0019829	0.026532855
GO:MF	neurotransmitter receptor activity involved in regulation of postsynapt	GO:0099529	0.026532855
GO:MF	secondary active transmembrane transporter activity	GO:0015291	0.026797636
GO:MF	solute:cation symporter activity	GO:0015294	0.028414727
GO:MF	ligand-gated channel activity	GO:0022834	0.030573608
GO:MF	nucleoside-triphosphatase regulator activity	GO:0060589	0.037925866
GO:MF	amino acid:sodium symporter activity	GO:0005283	0.038060159
GO:MF	neurotransmitter binding	GO:0042165	0.038696194
GO:MF	G protein-coupled glutamate receptor activity	GO:0098988	0.045678619
GO:MF	protein tyrosine kinase activity	GO:0004713	0.046508247
GO:BP	nervous system development	GO:0007399	2.75E-59
GO:BP	trans-synaptic signaling	GO:0099537	2.02E-46
GO:BP	synaptic signaling	GO:0099536	2.76E-46
GO:BP	chemical synaptic transmission	GO:0007268	3.97E-46
GO:BP	anterograde trans-synaptic signaling	GO:0098916	3.97E-46
GO:BP	neurogenesis	GO:0022008	6.41E-46

GO:BP	synapse organization	GO:0050808	2.88E-44
GO:BP	generation of neurons	GO:0048699	3.18E-44
GO:BP	modulation of chemical synaptic transmission	GO:0050804	1.10E-42
GO:BP	regulation of trans-synaptic signaling	GO:0099177	1.33E-42
GO:BP	cell-cell signaling	GO:0007267	8.62E-40
GO:BP	neuron development	GO:0048666	8.95E-40
GO:BP	neuron differentiation	GO:0030182	1.48E-39
GO:BP	system development	GO:0048731	1.62E-39
GO:BP	cell junction organization	GO:0034330	4.20E-38
GO:BP	neuron projection development	GO:0031175	8.52E-38
GO:BP	multicellular organism development	GO:0007275	8.62E-38
GO:BP	localization	GO:0051179	9.25E-37
GO:BP	plasma membrane bounded cell projection organization	GO:0120036	1.45E-31
GO:BP	cell projection organization	GO:0030030	2.08E-31
GO:BP	anatomical structure development	GO:0048856	2.70E-31
GO:BP	regulation of localization	GO:0032879	1.69E-30
GO:BP	neuron projection morphogenesis	GO:0048812	4.20E-29
GO:BP	cell projection morphogenesis	GO:0048858	9.92E-29
GO:BP	plasma membrane bounded cell projection morphogenesis	GO:0120039	2.84E-28
GO:BP	developmental process	GO:0032502	1.48E-27
GO:BP	cell part morphogenesis	GO:0032990	3.87E-27
GO:BP	cell morphogenesis involved in neuron differentiation	GO:0048667	6.77E-27
GO:BP	cell development	GO:0048468	7.02E-26
GO:BP	behavior	GO:0007610	1.72E-25
GO:BP	regulation of biological quality	GO:0065008	2.09E-25
GO:BP	cellular component morphogenesis	GO:0032989	2.03E-24
GO:BP	regulation of cell communication	GO:0010646	3.24E-24
GO:BP	regulation of signaling	GO:0023051	5.69E-24
GO:BP	neurotransmitter transport	GO:0006836	8.10E-24
GO:BP	cellular component organization	GO:0016043	1.35E-23
GO:BP	cation transport	GO:0006812	2.49E-23
GO:BP	central nervous system development	GO:0007417	3.20E-23
GO:BP	regulation of transport	GO:0051049	5.73E-23
GO:BP	cell morphogenesis involved in differentiation	GO:0000904	9.83E-23
GO:BP	cell morphogenesis	GO:0000902	1.61E-22
GO:BP	cell differentiation	GO:0030154	2.57E-22
GO:BP	export from cell	GO:0140352	3.89E-22
GO:BP	regulation of neurotransmitter levels	GO:0001505	5.40E-22
GO:BP	regulation of membrane potential	GO:0042391	9.06E-22
GO:BP	multicellular organismal process	GO:0032501	1.22E-21
GO:BP	cellular developmental process	GO:0048869	1.80E-21
GO:BP	cellular component organization or biogenesis	GO:0071840	1.81E-21
GO:BP	transport	GO:0006810	3.41E-21
GO:BP	anatomical structure morphogenesis	GO:0009653	3.52E-21
GO:BP	axon development	GO:0061564	1.88E-20
GO:BP	axonogenesis	GO:0007409	4.48E-20
GO:BP	cation transmembrane transport	GO:0098655	5.88E-20
GO:BP	brain development	GO:0007420	7.90E-20
GO:BP	establishment of localization	GO:0051234	8.74E-20
GO:BP	regulation of synapse structure or activity	GO:0050803	6.02E-19

GO:BP	head development	GO:0060322	1.14E-18
GO:BP	neurotransmitter secretion	GO:0007269	1.41E-18
GO:BP	signal release from synapse	GO:0099643	1.41E-18
GO:BP	synaptic vesicle cycle	GO:0099504	3.48E-18
GO:BP	regulation of synapse organization	GO:0050807	3.70E-18
GO:BP	movement of cell or subcellular component	GO:0006928	5.52E-18
GO:BP	locomotion	GO:0040011	1.75E-17
GO:BP	regulation of cellular component organization	GO:0051128	4.17E-17
GO:BP	signal release	GO:0023061	5.26E-17
GO:BP	secretion by cell	GO:0032940	6.01E-17
GO:BP	synapse assembly	GO:0007416	9.79E-17
GO:BP	metal ion transport	GO:0030001	1.19E-16
GO:BP	ion transmembrane transport	GO:0034220	1.36E-16
GO:BP	regulation of synaptic plasticity	GO:0048167	1.97E-16
GO:BP	cognition	GO:0050890	3.00E-16
GO:BP	vesicle-mediated transport in synapse	GO:0099003	3.11E-16
GO:BP	ion transport	GO:0006811	4.20E-16
GO:BP	transmembrane transport	GO:0055085	5.21E-16
GO:BP	cell migration	GO:0016477	6.68E-16
GO:BP	signaling	GO:0023052	8.35E-16
GO:BP	learning or memory	GO:0007611	2.96E-15
GO:BP	cell communication	GO:0007154	7.61E-15
GO:BP	inorganic cation transmembrane transport	GO:0098662	9.48E-15
GO:BP	cellular localization	GO:0051641	1.45E-14
GO:BP	secretion	GO:0046903	1.59E-14
GO:BP	regulation of postsynaptic membrane potential	GO:0060078	2.22E-14
GO:BP	inorganic ion transmembrane transport	GO:0098660	2.49E-14
GO:BP	cell motility	GO:0048870	3.13E-14
GO:BP	localization of cell	GO:0051674	3.13E-14
GO:BP	dendrite development	GO:0016358	5.20E-14
GO:BP	synaptic vesicle exocytosis	GO:0016079	7.47E-14
GO:BP	regulation of neurotransmitter secretion	GO:0046928	8.29E-14
GO:BP	regulation of neuron projection development	GO:0010975	8.53E-14
GO:BP	cell junction assembly	GO:0034329	1.46E-13
GO:BP	regulation of neurotransmitter transport	GO:0051588	2.88E-13
GO:BP	regulated exocytosis	GO:0045055	3.67E-13
GO:BP	regulation of secretion by cell	GO:1903530	5.32E-13
GO:BP	regulation of ion transport	GO:0043269	8.31E-13
GO:BP	animal organ development	GO:0048513	1.05E-12
GO:BP	learning	GO:0007612	1.07E-12
GO:BP	forebrain development	GO:0030900	1.69E-12
GO:BP	neuron migration	GO:0001764	2.18E-12
GO:BP	exocytosis	GO:0006887	2.52E-12
GO:BP	regulation of vesicle-mediated transport	GO:0060627	4.08E-12
GO:BP	regulation of secretion	GO:0051046	6.32E-12
GO:BP	dendrite morphogenesis	GO:0048813	6.35E-12
GO:BP	regulation of plasma membrane bounded cell projection organization	GO:0120035	7.36E-12
GO:BP	memory	GO:0007613	1.30E-11
GO:BP	glutamate receptor signaling pathway	GO:0007215	1.77E-11
GO:BP	sodium ion transmembrane transport	GO:0035725	1.87E-11

GO:BP	positive regulation of synaptic transmission	GO:0050806	1.88E-11
GO:BP	regulation of cell projection organization	GO:0031344	2.38E-11
GO:BP	chemical synaptic transmission, postsynaptic	GO:0099565	4.86E-11
GO:BP	regulation of transmembrane transport	GO:0034762	6.97E-11
GO:BP	monovalent inorganic cation transport	GO:0015672	8.37E-11
GO:BP	locomotory behavior	GO:0007626	1.08E-10
GO:BP	establishment of localization in cell	GO:0051649	1.52E-10
GO:BP	regulation of ion transmembrane transport	GO:0034765	1.79E-10
GO:BP	regulation of cation transmembrane transport	GO:1904062	2.90E-10
GO:BP	postsynapse organization	GO:0099173	3.34E-10
GO:BP	regulation of regulated secretory pathway	GO:1903305	5.94E-10
GO:BP	regulation of transmembrane transporter activity	GO:0022898	6.21E-10
GO:BP	excitatory postsynaptic potential	GO:0060079	6.27E-10
GO:BP	regulation of exocytosis	GO:0017157	8.42E-10
GO:BP	regulation of ion transmembrane transporter activity	GO:0032412	8.47E-10
GO:BP	regulation of nervous system process	GO:0031644	1.13E-09
GO:BP	protein localization to synapse	GO:0035418	1.19E-09
GO:BP	regulation of nervous system development	GO:0051960	1.22E-09
GO:BP	regulation of synaptic vesicle exocytosis	GO:2000300	2.04E-09
GO:BP	regulation of signal transduction	GO:0009966	2.05E-09
GO:BP	regulation of transporter activity	GO:0032409	2.60E-09
GO:BP	axon guidance	GO:0007411	2.93E-09
GO:BP	positive regulation of cellular process	GO:0048522	3.06E-09
GO:BP	neuron projection guidance	GO:0097485	3.73E-09
GO:BP	telencephalon development	GO:0021537	5.03E-09
GO:BP	cell adhesion	GO:0007155	7.18E-09
GO:BP	cell-cell adhesion via plasma-membrane adhesion molecules	GO:0098742	8.73E-09
GO:BP	regulation of cation channel activity	GO:2001257	8.86E-09
GO:BP	protein localization to cell junction	GO:1902414	9.45E-09
GO:BP	biological adhesion	GO:0022610	1.18E-08
GO:BP	regulation of synapse assembly	GO:0051963	1.34E-08
GO:BP	vesicle-mediated transport	GO:0016192	1.55E-08
GO:BP	positive regulation of cell communication	GO:0010647	2.28E-08
GO:BP	cellular component assembly	GO:0022607	2.35E-08
GO:BP	positive regulation of signaling	GO:0023056	2.83E-08
GO:BP	adult behavior	GO:0030534	3.51E-08
GO:BP	neuron projection organization	GO:0106027	3.53E-08
GO:BP	macromolecule localization	GO:0033036	3.74E-08
GO:BP	positive regulation of biological process	GO:0048518	3.78E-08
GO:BP	sodium ion transport	GO:0006814	3.95E-08
GO:BP	neuromuscular process	GO:0050905	3.99E-08
GO:BP	regulation of system process	GO:0044057	5.09E-08
GO:BP	synaptic transmission, glutamatergic	GO:0035249	5.27E-08
GO:BP	regulation of cellular process	GO:0050794	5.63E-08
GO:BP	neuromuscular process controlling balance	GO:0050885	5.70E-08
GO:BP	protein localization to cell periphery	GO:1990778	7.06E-08
GO:BP	positive regulation of cellular component organization	GO:0051130	7.67E-08
GO:BP	protein localization to membrane	GO:0072657	9.61E-08
GO:BP	positive regulation of nervous system development	GO:0051962	9.87E-08
GO:BP	biological regulation	GO:0065007	1.17E-07

GO:BP	regulation of neurotransmitter receptor activity	GO:0099601	1.20E-07
GO:BP	regulation of multicellular organismal process	GO:0051239	1.56E-07
GO:BP	protein localization	GO:0008104	1.65E-07
GO:BP	regulation of molecular function	GO:0065009	1.79E-07
GO:BP	neuron projection extension	GO:1990138	1.80E-07
GO:BP	cell surface receptor signaling pathway	GO:0007166	2.06E-07
GO:BP	regulation of cellular component movement	GO:0051270	3.37E-07
GO:BP	regulation of biological process	GO:0050789	3.57E-07
GO:BP	regulation of axonogenesis	GO:0050770	3.88E-07
GO:BP	regulation of cell migration	GO:0030334	3.91E-07
GO:BP	cell surface receptor signaling pathway involved in cell-cell signaling	GO:1905114	4.02E-07
GO:BP	developmental growth involved in morphogenesis	GO:0060560	5.20E-07
GO:BP	calcium ion transport	GO:0006816	5.46E-07
GO:BP	regulation of synaptic transmission, glutamatergic	GO:0051966	6.93E-07
GO:BP	transmission of nerve impulse	GO:0019226	7.09E-07
GO:BP	modulation of excitatory postsynaptic potential	GO:0098815	7.52E-07
GO:BP	negative regulation of signaling	GO:0023057	8.61E-07
GO:BP	positive regulation of synapse assembly	GO:0051965	8.68E-07
GO:BP	regulation of cell junction assembly	GO:1901888	9.49E-07
GO:BP	taxis	GO:0042330	1.40E-06
GO:BP	regulation of metal ion transport	GO:0010959	1.70E-06
GO:BP	negative regulation of cell communication	GO:0010648	1.71E-06
GO:BP	dendritic spine development	GO:0060996	1.77E-06
GO:BP	cellular component biogenesis	GO:0044085	1.84E-06
GO:BP	regulation of locomotion	GO:0040012	2.01E-06
GO:BP	potassium ion transmembrane transport	GO:0071805	2.06E-06
GO:BP	regulation of cell development	GO:0060284	2.20E-06
GO:BP	potassium ion transport	GO:0006813	2.36E-06
GO:BP	developmental maturation	GO:0021700	2.97E-06
GO:BP	negative regulation of cellular process	GO:0048523	3.42E-06
GO:BP	cellular process	GO:0009987	3.80E-06
GO:BP	regulation of cell motility	GO:2000145	3.85E-06
GO:BP	dendritic spine organization	GO:0097061	4.09E-06
GO:BP	divalent metal ion transport	GO:0070838	4.87E-06
GO:BP	multicellular organismal signaling	GO:0035637	5.52E-06
GO:BP	homophilic cell adhesion via plasma membrane adhesion molecules	GO:0007156	5.59E-06
GO:BP	long-term synaptic potentiation	GO:0060291	5.62E-06
GO:BP	protein localization to postsynapse	GO:0062237	5.75E-06
GO:BP	regulation of neuronal synaptic plasticity	GO:0048168	5.75E-06
GO:BP	intracellular signal transduction	GO:0035556	6.92E-06
GO:BP	divalent inorganic cation transport	GO:0072511	7.13E-06
GO:BP	axon extension	GO:0048675	7.80E-06
GO:BP	chemotaxis	GO:0006935	8.40E-06
GO:BP	regulation of cellular component biogenesis	GO:0044087	9.30E-06
GO:BP	regulation of neurogenesis	GO:0050767	9.66E-06
GO:BP	export across plasma membrane	GO:0140115	1.02E-05
GO:BP	positive regulation of cell junction assembly	GO:1901890	1.46E-05
GO:BP	negative regulation of synaptic transmission	GO:0050805	1.99E-05
GO:BP	regulation of AMPA receptor activity	GO:2000311	2.10E-05
GO:BP	dendritic spine morphogenesis	GO:0060997	2.15E-05

GO:BP	nervous system process	GO:0050877	2.19E-05
GO:BP	regulation of neuron migration	GO:2001222	2.24E-05
GO:BP	calcium ion transmembrane transport	GO:0070588	2.27E-05
GO:BP	receptor localization to synapse	GO:0097120	2.65E-05
GO:BP	positive regulation of transport	GO:0051050	2.71E-05
GO:BP	response to alkaloid	GO:0043279	3.33E-05
GO:BP	action potential	GO:0001508	3.39E-05
GO:BP	developmental growth	GO:0048589	3.49E-05
GO:BP	regulation of developmental process	GO:0050793	3.76E-05
GO:BP	regulation of anatomical structure morphogenesis	GO:0022603	3.80E-05
GO:BP	regulation of amine transport	GO:0051952	4.88E-05
GO:BP	developmental cell growth	GO:0048588	4.96E-05
GO:BP	import into cell	GO:0098657	5.17E-05
GO:BP	system process	GO:0003008	5.89E-05
GO:BP	neuron apoptotic process	GO:0051402	5.97E-05
GO:BP	central nervous system neuron differentiation	GO:0021953	6.26E-05
GO:BP	gamma-aminobutyric acid transport	GO:0015812	6.74E-05
GO:BP	negative regulation of biological process	GO:0048519	7.66E-05
GO:BP	cellular protein localization	GO:0034613	8.30E-05
GO:BP	cellular macromolecule localization	GO:0070727	0.0001098
GO:BP	protein localization to postsynaptic membrane	GO:1903539	0.000119196
GO:BP	positive regulation of cell projection organization	GO:0031346	0.000120078
GO:BP	regulation of protein localization to membrane	GO:1905475	0.000132915
GO:BP	postsynapse assembly	GO:0099068	0.000138112
GO:BP	receptor-mediated endocytosis	GO:0006898	0.00014577
GO:BP	amine transport	GO:0015837	0.000153067
GO:BP	neuron death	GO:0070997	0.000161047
GO:BP	response to cocaine	GO:0042220	0.000183432
GO:BP	signal transduction	GO:0007165	0.000186962
GO:BP	ion homeostasis	GO:0050801	0.000195286
GO:BP	nitrogen compound transport	GO:0071705	0.00020416
GO:BP	regulation of postsynapse organization	GO:0099175	0.000215762
GO:BP	cellular chemical homeostasis	GO:0055082	0.000229429
GO:BP	cellular homeostasis	GO:0019725	0.000235469
GO:BP	response to organonitrogen compound	GO:0010243	0.000236751
GO:BP	negative regulation of locomotion	GO:0040013	0.000250898
GO:BP	regulation of axon extension	GO:0030516	0.000285929
GO:BP	regulation of signaling receptor activity	GO:0010469	0.000308813
GO:BP	regulation of postsynaptic neurotransmitter receptor activity	GO:0098962	0.000332878
GO:BP	regulation of sodium ion transmembrane transport	GO:1902305	0.000338647
GO:BP	postsynaptic specialization assembly	GO:0098698	0.000342568
GO:BP	neuron maturation	GO:0042551	0.000346671
GO:BP	regulation of postsynaptic membrane neurotransmitter receptor levels	GO:0099072	0.000394755
GO:BP	chemical homeostasis	GO:0048878	0.000434325
GO:BP	regulation of response to stimulus	GO:0048583	0.000437687
GO:BP	hindbrain development	GO:0030902	0.000446691
GO:BP	positive regulation of synaptic transmission, glutamatergic	GO:0051968	0.000481692
GO:BP	ionotropic glutamate receptor signaling pathway	GO:0035235	0.000489801
GO:BP	amino acid transport	GO:0006865	0.00052096
GO:BP	protein localization to plasma membrane	GO:0072659	0.00052668

GO:BP	regulation of protein localization	GO:0032880	0.000543366
GO:BP	negative regulation of cellular component movement	GO:0051271	0.000574101
GO:BP	calcium-ion regulated exocytosis	GO:0017156	0.000602797
GO:BP	growth	GO:0040007	0.000624881
GO:BP	neuron cell-cell adhesion	GO:0007158	0.000635736
GO:BP	positive regulation of cell development	GO:0010720	0.000643116
GO:BP	regulation of G protein-coupled receptor signaling pathway	GO:0008277	0.000708213
GO:BP	cell-cell adhesion	GO:0098609	0.000729779
GO:BP	inorganic ion homeostasis	GO:0098771	0.000756893
GO:BP	regulation of cell size	GO:0008361	0.000786649
GO:BP	postsynaptic density assembly	GO:0097107	0.000795915
GO:BP	forebrain cell migration	GO:0021885	0.000869159
GO:BP	response to nitrogen compound	GO:1901698	0.000888937
GO:BP	positive regulation of cation transmembrane transport	GO:1904064	0.000948726
GO:BP	regulation of dendritic spine development	GO:0060998	0.000974478
GO:BP	cation homeostasis	GO:0055080	0.00102821
GO:BP	positive regulation of molecular function	GO:0044093	0.001088422
GO:BP	retina development in camera-type eye	GO:0060041	0.001111494
GO:BP	negative regulation of cell migration	GO:0030336	0.001138896
GO:BP	catecholamine transport	GO:0051937	0.001142055
GO:BP	response to stimulus	GO:0050896	0.001181931
GO:BP	anion transport	GO:0006820	0.001259508
GO:BP	regulation of extent of cell growth	GO:0061387	0.00135342
GO:BP	positive regulation of axonogenesis	GO:0050772	0.001510746
GO:BP	gamma-aminobutyric acid secretion	GO:0014051	0.001643254
GO:BP	protein localization to postsynaptic specialization membrane	GO:0099633	0.00176264
GO:BP	neurotransmitter receptor localization to postsynaptic specialization m	GO:0099645	0.00176264
GO:BP	cellular response to stimulus	GO:0051716	0.001957809
GO:BP	gliogenesis	GO:0042063	0.001961428
GO:BP	monoamine transport	GO:0015844	0.001990085
GO:BP	positive regulation of developmental process	GO:0051094	0.001995795
GO:BP	cerebellar cortex development	GO:0021695	0.002126948
GO:BP	positive regulation of signal transduction	GO:0009967	0.002296174
GO:BP	positive regulation of ion transport	GO:0043270	0.002370704
GO:BP	regulation of neuron apoptotic process	GO:0043523	0.002415996
GO:BP	sensory system development	GO:0048880	0.002457437
GO:BP	phosphorylation	GO:0016310	0.002462678
GO:BP	regulation of catecholamine secretion	GO:0050433	0.002547724
GO:BP	response to oxygen-containing compound	GO:1901700	0.00269236
GO:BP	negative regulation of cell motility	GO:2000146	0.002974311
GO:BP	regulation of cellular component size	GO:0032535	0.003242614
GO:BP	cellular ion homeostasis	GO:0006873	0.003280335
GO:BP	social behavior	GO:0035176	0.003555547
GO:BP	presynapse assembly	GO:0099054	0.003555547
GO:BP	metal ion homeostasis	GO:0055065	0.003559864
GO:BP	telencephalon cell migration	GO:0022029	0.00361597
GO:BP	catecholamine secretion	GO:0050432	0.00361597
GO:BP	central nervous system neuron development	GO:0021954	0.003729183
GO:BP	synaptic vesicle recycling	GO:0036465	0.003853761
GO:BP	metencephalon development	GO:0022037	0.004246104

GO:BP	acidic amino acid transport	GO:0015800	0.004285564
GO:BP	regulation of dendritic spine morphogenesis	GO:0061001	0.004310434
GO:BP	eye development	GO:0001654	0.004388662
GO:BP	cellular cation homeostasis	GO:0030003	0.004449655
GO:BP	neural retina development	GO:0003407	0.004483734
GO:BP	positive regulation of neurogenesis	GO:0050769	0.00487473
GO:BP	regulation of sodium ion transport	GO:0002028	0.004905013
GO:BP	visual system development	GO:0150063	0.005172614
GO:BP	neurotransmitter receptor transport	GO:0099637	0.005202404
GO:BP	camera-type eye morphogenesis	GO:0048593	0.005260857
GO:BP	associative learning	GO:0008306	0.005579813
GO:BP	negative regulation of neuron apoptotic process	GO:0043524	0.006059271
GO:BP	camera-type eye development	GO:0043010	0.006124695
GO:BP	biological process involved in intraspecies interaction between organi	GO:0051703	0.006252184
GO:BP	cell growth	GO:0016049	0.006285938
GO:BP	receptor internalization	GO:0031623	0.006569069
GO:BP	positive regulation of excitatory postsynaptic potential	GO:2000463	0.006723028
GO:BP	animal organ morphogenesis	GO:0009887	0.006808156
GO:BP	postsynaptic specialization organization	GO:0099084	0.007020745
GO:BP	regulation of neuron death	GO:1901214	0.007180501
GO:BP	cerebellum development	GO:0021549	0.007337522
GO:BP	cellular response to chemical stimulus	GO:0070887	0.00832804
GO:BP	organic substance transport	GO:0071702	0.008334597
GO:BP	synapse maturation	GO:0060074	0.008667542
GO:BP	excitatory synapse assembly	GO:1904861	0.008667542
GO:BP	maintenance of synapse structure	GO:0099558	0.008667542
GO:BP	positive regulation of sodium ion transmembrane transport	GO:1902307	0.008732763
GO:BP	positive regulation of protein localization to membrane	GO:1905477	0.008988134
GO:BP	sensory organ development	GO:0007423	0.009174429
GO:BP	cellular response to endogenous stimulus	GO:0071495	0.00988411
GO:BP	synaptic vesicle localization	GO:0097479	0.01059444
GO:BP	regulation of anatomical structure size	GO:0090066	0.010633303
GO:BP	regulation of presynapse assembly	GO:1905606	0.010782417
GO:BP	positive regulation of ion transmembrane transporter activity	GO:0032414	0.011282893
GO:BP	import across plasma membrane	GO:0098739	0.011612759
GO:BP	anatomical structure maturation	GO:0071695	0.011953951
GO:BP	inorganic cation import across plasma membrane	GO:0098659	0.012058461
GO:BP	inorganic ion import across plasma membrane	GO:0099587	0.012058461
GO:BP	regulation of endocytosis	GO:0030100	0.012485253
GO:BP	regulation of intracellular signal transduction	GO:1902531	0.013307883
GO:BP	response to growth factor	GO:0070848	0.013381723
GO:BP	presynapse organization	GO:0099172	0.014781151
GO:BP	enzyme linked receptor protein signaling pathway	GO:0007167	0.016200862
GO:BP	response to organic substance	GO:0010033	0.016219933
GO:BP	negative regulation of signal transduction	GO:0009968	0.016505242
GO:BP	phosphorus metabolic process	GO:0006793	0.01818528
GO:BP	regulation of sodium ion transmembrane transporter activity	GO:2000649	0.018427752
GO:BP	G protein-coupled glutamate receptor signaling pathway	GO:0007216	0.020379199
GO:BP	regulation of presynapse organization	GO:0099174	0.020727589
GO:BP	anatomical structure formation involved in morphogenesis	GO:0048646	0.021611263

GO:BP	postsynaptic neurotransmitter receptor diffusion trapping	GO:0098970	0.022246874
GO:BP	receptor diffusion trapping	GO:0098953	0.022246874
GO:BP	neurotransmitter receptor diffusion trapping	GO:0099628	0.022246874
GO:BP	regulation of modification of postsynaptic structure	GO:0099159	0.022246874
GO:BP	response to endogenous stimulus	GO:0009719	0.022707488
GO:BP	receptor metabolic process	GO:0043112	0.022726548
GO:BP	positive regulation of transporter activity	GO:0032411	0.022927096
GO:BP	glial cell migration	GO:0008347	0.023750131
GO:BP	pallium development	GO:0021543	0.024328822
GO:BP	postsynaptic density organization	GO:0097106	0.02543716
GO:BP	neuromuscular junction development	GO:0007528	0.0262341
GO:BP	positive regulation of neuron projection development	GO:0010976	0.029164826
GO:BP	negative regulation of neuron death	GO:1901215	0.030884742
GO:BP	synaptic vesicle endocytosis	GO:0048488	0.03207853
GO:BP	presynaptic endocytosis	GO:0140238	0.03207853
GO:BP	regulation of cellular localization	GO:0060341	0.033724686
GO:BP	positive regulation of multicellular organismal process	GO:0051240	0.034810975
GO:BP	divalent inorganic cation homeostasis	GO:0072507	0.035553867
GO:BP	regulation of catalytic activity	GO:0050790	0.035590585
GO:BP	regulation of synaptic vesicle fusion to presynaptic active zone memb	GO:0031630	0.039653709
GO:BP	neuron cellular homeostasis	GO:0070050	0.039747711
GO:BP	protein phosphorylation	GO:0006468	0.040371198
GO:BP	carboxylic acid transport	GO:0046942	0.040886115
GO:BP	cellular response to organic substance	GO:0071310	0.041548754
GO:BP	regulation of dendrite development	GO:0050773	0.044303667
GO:BP	cellular metal ion homeostasis	GO:0006875	0.045671273
GO:BP	negative regulation of neuron projection development	GO:0010977	0.046434397
GO:BP	phosphate-containing compound metabolic process	GO:0006796	0.046619241
GO:BP	calcium ion homeostasis	GO:0055074	0.046685494
GO:BP	axon extension involved in axon guidance	GO:0048846	0.049606074
GO:BP	cerebellar Purkinje cell layer development	GO:0021680	0.049606074
GO:BP	neuron projection extension involved in neuron projection guidance	GO:1902284	0.049606074
GO:CC	synapse	GO:0045202	3.89E-76
GO:CC	cell junction	GO:0030054	2.46E-73
GO:CC	neuron projection	GO:0043005	8.68E-59
GO:CC	somatodendritic compartment	GO:0036477	3.27E-56
GO:CC	postsynapse	GO:0098794	4.39E-55
GO:CC	dendrite	GO:0030425	5.95E-51
GO:CC	dendritic tree	GO:0097447	8.09E-51
GO:CC	synaptic membrane	GO:0097060	3.28E-49
GO:CC	plasma membrane bounded cell projection	GO:0120025	4.78E-46
GO:CC	cell projection	GO:0042995	5.78E-45
GO:CC	axon	GO:0030424	2.76E-41
GO:CC	glutamatergic synapse	GO:0098978	1.06E-40
GO:CC	neuronal cell body	GO:0043025	7.14E-38
GO:CC	cell body	GO:0044297	1.28E-36
GO:CC	presynapse	GO:0098793	2.94E-36
GO:CC	postsynaptic membrane	GO:0045211	6.24E-36
GO:CC	intrinsic component of synaptic membrane	GO:0099240	1.06E-35
GO:CC	plasma membrane region	GO:0098590	3.04E-35

GO:CC	neuron to neuron synapse	GO:0098984	7.46E-34
GO:CC	integral component of synaptic membrane	GO:0099699	1.29E-32
GO:CC	asymmetric synapse	GO:0032279	1.09E-31
GO:CC	postsynaptic specialization	GO:0099572	1.36E-30
GO:CC	postsynaptic density	GO:0014069	1.42E-29
GO:CC	intrinsic component of postsynaptic membrane	GO:0098936	3.13E-29
GO:CC	integral component of postsynaptic membrane	GO:0099055	5.25E-27
GO:CC	intrinsic component of plasma membrane	GO:0031226	6.32E-26
GO:CC	membrane	GO:0016020	8.82E-24
GO:CC	integral component of plasma membrane	GO:0005887	1.56E-23
GO:CC	plasma membrane	GO:0005886	3.44E-21
GO:CC	cell periphery	GO:0071944	3.98E-21
GO:CC	distal axon	GO:0150034	9.83E-21
GO:CC	presynaptic membrane	GO:0042734	2.40E-20
GO:CC	transmembrane transporter complex	GO:1902495	1.09E-16
GO:CC	neuron spine	GO:0044309	3.06E-16
GO:CC	transporter complex	GO:1990351	4.79E-16
GO:CC	postsynaptic specialization membrane	GO:0099634	8.03E-16
GO:CC	dendritic spine	GO:0043197	5.88E-15
GO:CC	postsynaptic density membrane	GO:0098839	6.84E-14
GO:CC	synaptic vesicle	GO:0008021	9.47E-14
GO:CC	ion channel complex	GO:0034702	2.26E-13
GO:CC	intrinsic component of postsynaptic specialization membrane	GO:0098948	2.46E-13
GO:CC	plasma membrane protein complex	GO:0098797	2.62E-13
GO:CC	Schaffer collateral - CA1 synapse	GO:0098685	7.09E-13
GO:CC	intrinsic component of membrane	GO:0031224	1.10E-12
GO:CC	exocytic vesicle	GO:0070382	1.52E-12
GO:CC	transport vesicle	GO:0030133	2.11E-12
GO:CC	endomembrane system	GO:0012505	2.44E-12
GO:CC	site of polarized growth	GO:0030427	3.33E-12
GO:CC	cytoplasmic vesicle	GO:0031410	3.71E-12
GO:CC	intracellular vesicle	GO:0097708	4.52E-12
GO:CC	intrinsic component of postsynaptic density membrane	GO:0099146	5.17E-12
GO:CC	GABA-ergic synapse	GO:0098982	7.12E-12
GO:CC	integral component of postsynaptic specialization membrane	GO:0099060	7.12E-12
GO:CC	growth cone	GO:0030426	4.00E-11
GO:CC	cation channel complex	GO:0034703	4.58E-11
GO:CC	neuron projection terminus	GO:0044306	6.81E-11
GO:CC	axon terminus	GO:0043679	1.46E-10
GO:CC	vesicle	GO:0031982	1.59E-10
GO:CC	integral component of postsynaptic density membrane	GO:0099061	1.72E-10
GO:CC	exocytic vesicle membrane	GO:0099501	1.82E-10
GO:CC	synaptic vesicle membrane	GO:0030672	1.82E-10
GO:CC	integral component of membrane	GO:0016021	3.57E-10
GO:CC	transport vesicle membrane	GO:0030658	4.08E-10
GO:CC	perikaryon	GO:0043204	5.10E-10
GO:CC	main axon	GO:0044304	1.56E-09
GO:CC	presynaptic active zone	GO:0048786	1.89E-09
GO:CC	intrinsic component of presynaptic membrane	GO:0098889	2.01E-09
GO:CC	bounding membrane of organelle	GO:0098588	3.31E-09

GO:CC	receptor complex	GO:0043235	6.22E-08
GO:CC	integral component of presynaptic membrane	GO:0099056	8.81E-08
GO:CC	vesicle membrane	GO:0012506	1.02E-07
GO:CC	membrane protein complex	GO:0098796	1.43E-07
GO:CC	cytoplasmic vesicle membrane	GO:0030659	2.34E-07
GO:CC	cytoplasm	GO:0005737	3.53E-07
GO:CC	cell leading edge	GO:0031252	6.16E-07
GO:CC	integral component of synaptic vesicle membrane	GO:0030285	8.62E-07
GO:CC	intrinsic component of synaptic vesicle membrane	GO:0098563	1.24E-06
GO:CC	secretory vesicle	GO:0099503	1.26E-06
GO:CC	neuron projection membrane	GO:0032589	4.16E-06
GO:CC	calyx of Held	GO:0044305	4.82E-06
GO:CC	Golgi apparatus	GO:0005794	5.31E-06
GO:CC	potassium channel complex	GO:0034705	1.31E-05
GO:CC	organelle membrane	GO:0031090	1.34E-05
GO:CC	perinuclear region of cytoplasm	GO:0048471	1.44E-05
GO:CC	dendritic shaft	GO:0043198	1.48E-05
GO:CC	leading edge membrane	GO:0031256	1.52E-05
GO:CC	cell projection membrane	GO:0031253	1.64E-05
GO:CC	cellular anatomical entity	GO:0110165	1.92E-05
GO:CC	presynaptic active zone membrane	GO:0048787	2.92E-05
GO:CC	voltage-gated potassium channel complex	GO:0008076	7.81E-05
GO:CC	dendrite membrane	GO:0032590	0.000192231
GO:CC	myelin sheath	GO:0043209	0.000278672
GO:CC	ionotropic glutamate receptor complex	GO:0008328	0.000363234
GO:CC	microtubule	GO:0005874	0.000375018
GO:CC	neurotransmitter receptor complex	GO:0098878	0.000541134
GO:CC	excitatory synapse	GO:0060076	0.000553581
GO:CC	endosome	GO:0005768	0.000642269
GO:CC	apical dendrite	GO:0097440	0.00071038
GO:CC	plasma membrane signaling receptor complex	GO:0098802	0.000884579
GO:CC	cytoskeleton	GO:0005856	0.00093126
GO:CC	anchoring junction	GO:0070161	0.001196491
GO:CC	synaptic cleft	GO:0043083	0.001294097
GO:CC	Golgi membrane	GO:0000139	0.001405339
GO:CC	axonal growth cone	GO:0044295	0.003109263
GO:CC	juxtaparanode region of axon	GO:0044224	0.003247936
GO:CC	cell surface	GO:0009986	0.005318345
GO:CC	neurofilament	GO:0005883	0.005787819
GO:CC	cell body membrane	GO:0044298	0.005920884
GO:CC	Golgi apparatus subcompartment	GO:0098791	0.006073492
GO:CC	sarcolemma	GO:0042383	0.007957756
GO:CC	early endosome	GO:0005769	0.01222004
GO:CC	extrinsic component of membrane	GO:0019898	0.013334358
GO:CC	cell-cell junction	GO:0005911	0.013922819
GO:CC	neuromuscular junction	GO:0031594	0.015513057
GO:CC	intrinsic component of organelle membrane	GO:0031300	0.015828938
GO:CC	extracellular matrix	GO:0031012	0.021469325
GO:CC	intrinsic component of presynaptic active zone membrane	GO:0098945	0.023454882
GO:CC	neuronal cell body membrane	GO:0032809	0.027045217

GO:CC	clathrin-coated vesicle	GO:0030136	0.02758949
GO:CC	perisynaptic space	GO:0099544	0.027602681
GO:CC	pigment granule	GO:0048770	0.02967998
GO:CC	melanosome	GO:0042470	0.02967998
GO:CC	anchored component of membrane	GO:0031225	0.031693917
GO:CC	terminal bouton	GO:0043195	0.033433348
GO:CC	cation-transporting ATPase complex	GO:0090533	0.033588846
GO:CC	endoplasmic reticulum	GO:0005783	0.036369755
GO:CC	voltage-gated calcium channel complex	GO:0005891	0.0401892
GO:CC	dendritic spine head	GO:0044327	0.04749414
GO:CC	dopaminergic synapse	GO:0098691	0.04749414
GO:CC	organelle subcompartment	GO:0031984	0.047650142
KEGG	Axon guidance	KEGG:04360	1.47E-08
KEGG	Synaptic vesicle cycle	KEGG:04721	5.09E-08
KEGG	Glutamatergic synapse	KEGG:04724	1.75E-07
KEGG	Adrenergic signaling in cardiomyocytes	KEGG:04261	6.41E-07
KEGG	cAMP signaling pathway	KEGG:04024	2.13E-06
KEGG	Circadian entrainment	KEGG:04713	2.43E-05
KEGG	GABAergic synapse	KEGG:04727	3.74E-05
KEGG	Aldosterone synthesis and secretion	KEGG:04925	3.75E-05
KEGG	Endocrine and other factor-regulated calcium reabsorption	KEGG:04961	9.53E-05
KEGG	Calcium signaling pathway	KEGG:04020	9.83E-05
KEGG	Dopaminergic synapse	KEGG:04728	0.000269069
KEGG	Salivary secretion	KEGG:04970	0.000792934
KEGG	Oxytocin signaling pathway	KEGG:04921	0.001602256
KEGG	Cardiac muscle contraction	KEGG:04260	0.003180593
KEGG	Insulin secretion	KEGG:04911	0.015220855
KEGG	Phospholipase D signaling pathway	KEGG:04072	0.02116981
KEGG	Gastric acid secretion	KEGG:04971	0.025778058
KEGG	Morphine addiction	KEGG:05032	0.025794171
KEGG	Dilated cardiomyopathy	KEGG:05414	0.034713841
KEGG	Mineral absorption	KEGG:04978	0.03817237
KEGG	Nicotine addiction	KEGG:05033	0.044580484
KEGG	Cholinergic synapse	KEGG:04725	0.049256965
REAC	Neuronal System	REAC:R-MMU-1	7.24E-24
REAC	Transmission across Chemical Synapses	REAC:R-MMU-1	5.57E-14
REAC	Cardiac conduction	REAC:R-MMU-5	5.07E-09
REAC	Neurotransmitter release cycle	REAC:R-MMU-1	7.19E-06
REAC	Protein-protein interactions at synapses	REAC:R-MMU-6	1.34E-05
REAC	Ion homeostasis	REAC:R-MMU-5	1.63E-05
REAC	Muscle contraction	REAC:R-MMU-3	1.73E-05
REAC	Neurotransmitter receptors and postsynaptic signal transmission	REAC:R-MMU-1	1.89E-05
REAC	Axon guidance	REAC:R-MMU-4	3.18E-05
REAC	Nervous system development	REAC:R-MMU-9	3.18E-05
REAC	GABA synthesis, release, reuptake and degradation	REAC:R-MMU-8	6.19E-05
REAC	LGI-ADAM interactions	REAC:R-MMU-5	7.17E-05
REAC	Phase 0 - rapid depolarisation	REAC:R-MMU-5	9.61E-05
REAC	Neurexins and neuroligins	REAC:R-MMU-6	0.001693319
REAC	Reduction of cytosolic Ca++ levels	REAC:R-MMU-4	0.001776021
REAC	Dopamine Neurotransmitter Release Cycle	REAC:R-MMU-2	0.00521974

REAC	Developmental Biology	REAC:R-MMU-1:	0.006315229
REAC	Transport of inorganic cations/anions and amino acids/oligopeptides	REAC:R-MMU-4:	0.006445104
REAC	Presynaptic depolarization and calcium channel opening	REAC:R-MMU-1	0.007137291
REAC	Serotonin Neurotransmitter Release Cycle	REAC:R-MMU-1:	0.011181504
REAC	Trafficking of AMPA receptors	REAC:R-MMU-3!	0.013265649
REAC	Glutamate binding, activation of AMPA receptors and synaptic plastic	REAC:R-MMU-3!	0.013265649
REAC	Transport of small molecules	REAC:R-MMU-3:	0.018347951
REAC	Voltage gated Potassium channels	REAC:R-MMU-1:	0.02280103
REAC	Semaphorin interactions	REAC:R-MMU-3	0.033054012
REAC	Potassium Channels	REAC:R-MMU-1:	0.038487496
WP	Calcium Regulation in the Cardiac Cell	WP:WP553	0.000381386
WP	Splicing factor NOVA regulated synaptic proteins	WP:WP1983	0.000723538
WP	GPCRs, Class C Metabotropic glutamate, pheromone	WP:WP327	0.001600689
WP	Myometrial Relaxation and Contraction Pathways	WP:WP385	0.007265772
WP	Hypothetical Network for Drug Addiction	WP:WP1246	0.020756268
TF	Factor: CTCF; motif: NAGGGGGCGCNNKNNNN; match class: 1	TF:M08995_1	8.53E-52
TF	Factor: E2F-1; motif: GNGGGCGGGRMN; match class: 1	TF:M10209_1	7.18E-45
TF	Factor: MOVO-B; motif: GNGGGGG	TF:M01104	1.71E-44
TF	Factor: Sp1; motif: GGNGGGGGNGGGGGMGGGGCNGGG	TF:M10375	6.84E-44
TF	Factor: Kaiso; motif: GCMGGGRGCRGS; match class: 1	TF:M03876_1	5.90E-42
TF	Factor: GKLF; motif: NNNRGGNGNGGSN; match class: 1	TF:M07289_1	1.02E-41
TF	Factor: WT1; motif: CGCCCCCNCN	TF:M02036	1.66E-41
TF	Factor: Kaiso; motif: GCMGGGRGCRGS	TF:M03876	3.47E-41
TF	Factor: Sp1; motif: GGNGGGGGNGGGGGMGGGGCNGGG; match	TF:M10375_1	4.91E-40
TF	Factor: ZF5; motif: NRNGNGCGCGCWN; match class: 1	TF:M00333_1	1.91E-39
TF	Factor: ZF5; motif: GSGCGCGR; match class: 1	TF:M00716_1	3.21E-39
TF	Factor: GKLF; motif: NNRRGRRNGNSNNN; match class: 1	TF:M07040_1	5.30E-39
TF	Factor: BCL6B; motif: NNNNCCGCCCCWNNNN; match class: 1	TF:M02844_1	6.21E-38
TF	Factor: E2F-1; motif: GNGGGCGGGRMN	TF:M10209	9.03E-38
TF	Factor: CTCF; motif: NAGGGGGCGCNNKNNNN	TF:M08995	9.47E-38
TF	Factor: E2F-3; motif: GGCGGGN	TF:M02089	1.09E-37
TF	Factor: FOXN4; motif: NNWANNCGWMCGCGTCNNNNMT; match c	TF:M04662_1	1.25E-37
TF	Factor: ZF5; motif: GSGCGCGR	TF:M00716	1.18E-36
TF	Factor: E2F; motif: GGCGSG	TF:M00803	1.31E-36
TF	Factor: BEN; motif: CAGCGRNV; match class: 1	TF:M01240_1	1.29E-35
TF	Factor: E2F; motif: GGCGSG; match class: 1	TF:M00803_1	6.24E-35
TF	Factor: Egr-1; motif: GCGGGGGCGG	TF:M07354	7.00E-35
TF	Factor: Sp5; motif: RNGGRGGNGGRGNNGGGGGAGGRG	TF:M10376	2.67E-34
TF	Factor: ZF5; motif: NRNGNGCGCGCWN	TF:M00333	7.10E-34
TF	Factor: SP1:SP3; motif: CCSCCCCCYCC	TF:M01219	7.18E-34
TF	Factor: Kaiso; motif: SARNYCTCGCGAGAN; match class: 1	TF:M10276_1	7.77E-33
TF	Factor: WT1; motif: SMCNCCNSC	TF:M01118	1.46E-32
TF	Factor: ZF5; motif: GYCGCGCARNGCNN; match class: 1	TF:M02933_1	1.88E-32
TF	Factor: EGR; motif: CGCCCCCGCNN	TF:M08878	2.11E-32
TF	Factor: ZF5; motif: GYCGCGCARNGCNN	TF:M02933	3.90E-32
TF	Factor: FOXN4; motif: NNWANNCGWMCGCGTCNNNNMT	TF:M04662	1.55E-31
TF	Factor: ZBP89; motif: CCCCKCCCCCNN	TF:M07397	6.95E-31
TF	Factor: BEN; motif: CAGCGRNV	TF:M01240	7.67E-31
TF	Factor: Osx; motif: CCNCCCCCNNN	TF:M07329	3.36E-30
TF	Factor: E2F-3; motif: GGCGGGN; match class: 1	TF:M02089_1	4.48E-30

TF	Factor: AP-2alpha; motif: NGCCYSNNGSN	TF:M01857	5.75E-30
TF	Factor: GKLF; motif: NNNRGGNGNGGSN	TF:M07289	5.89E-30
TF	Factor: GKLF; motif: NNRRGRRNGNSNNN	TF:M07040	6.34E-30
TF	Factor: AP2; motif: GCCYGSGGSN	TF:M08867	1.01E-29
TF	Factor: MOVO-B; motif: GNGGGGG; match class: 1	TF:M01104_1	2.20E-29
TF	Factor: Kaiso; motif: SARNYCTCGCGAGAN	TF:M10276	2.74E-29
TF	Factor: WT1; motif: GNGGGGGCGGGG	TF:M03893	4.06E-29
TF	Factor: ZAC; motif: KGGGCCR	TF:M05547	1.55E-28
TF	Factor: MAZ; motif: GGGGAGGG	TF:M00649	3.94E-28
TF	Factor: Egr-1; motif: GCGGGGGCGG	TF:M01873	5.56E-28
TF	Factor: Sp1; motif: CCCCGCCCCN	TF:M00933	5.97E-28
TF	Factor: KROX; motif: CCCGCCCCCRCCCC	TF:M00982	1.05E-27
TF	Factor: Sp1; motif: GGGGCGGGGC	TF:M00931	7.64E-27
TF	Factor: CKROX; motif: SCCCTCCCC	TF:M01175	8.47E-27
TF	Factor: BTEB3; motif: BNRNGGGAGGNGT	TF:M01865	9.58E-27
TF	Factor: Sp1; motif: NNGGGGCGGGGNN	TF:M00932	1.59E-26
TF	Factor: WT1; motif: CGCCCCCNCN; match class: 1	TF:M02036_1	1.83E-26
TF	Factor: SP1; motif: GGGGYGGGGNS	TF:M01303	2.71E-26
TF	Factor: Pax-4; motif: NNNNNYCACCCB; match class: 1	TF:M00378_1	1.01E-25
TF	Factor: AP-2; motif: MKCCCSCNGGCG	TF:M00189	1.97E-25
TF	Factor: WT1; motif: NNGGGNGGGSGN	TF:M07436	2.64E-25
TF	Factor: Egr-1; motif: NNGCGKGGGCGGGG	TF:M10213	1.69E-24
TF	Factor: Sp1; motif: NGGGGGCGGGGYN	TF:M00196	4.23E-24
TF	Factor: Egr1; motif: NCCGCCCCCGCANN	TF:M02744	1.63E-23
TF	Factor: BCL6B; motif: NNNNCCGCCCCWNNNN	TF:M02844	2.43E-23
TF	Factor: RNF96; motif: BCCCGCRGCC	TF:M01199	4.20E-23
TF	Factor: MAZ; motif: GGGGAGGG; match class: 1	TF:M00649_1	5.53E-23
TF	Factor: Sp1; motif: NGGGGCGGGGN	TF:M07395	8.23E-23
TF	Factor: AP-2; motif: GSCCSCRGGCNRNRNN	TF:M00800	1.59E-22
TF	Factor: AP-2alpha; motif: NGCCYSNNGSN; match class: 1	TF:M01857_1	6.41E-22
TF	Factor: E2F-4; motif: GCGGGAAANA	TF:M02090	9.90E-22
TF	Factor: LKLF; motif: GGGGTGGKSN	TF:M07261	9.99E-22
TF	Factor: ZNF151; motif: GGSGGNGGGGGAGGGGMGG	TF:M10412	1.19E-21
TF	Factor: Egr-1; motif: GCGGGGGCGG; match class: 1	TF:M07354_1	1.34E-21
TF	Factor: Sp1; motif: NGGGGCGGGGN; match class: 1	TF:M07395_1	2.75E-21
TF	Factor: Zic1; motif: NNCCCCCGGGGGGG	TF:M02835	3.01E-21
TF	Factor: AP-2gamma; motif: GCCYNNGGS	TF:M00470	4.31E-21
TF	Factor: Sp1; motif: CCCCGCCCCN; match class: 1	TF:M00933_1	1.22E-20
TF	Factor: EGR; motif: CGCCCCCGCNN; match class: 1	TF:M08878_1	1.40E-20
TF	Factor: BTEB2; motif: RGGGNGKGGN	TF:M07277	2.16E-20
TF	Factor: Sp1; motif: NNGGGGCGGGGNN; match class: 1	TF:M00932_1	2.20E-20
TF	Factor: HSF4; motif: CTGCMRN; match class: 1	TF:M07322_1	2.39E-20
TF	Factor: SP2; motif: GNNGGGGGCGGGGSN	TF:M03807	3.32E-20
TF	Factor: Zic1; motif: NNCCCCCGGGGGGG; match class: 1	TF:M02835_1	3.50E-20
TF	Factor: WT1; motif: NGCGGGGGGGTSMMCYN	TF:M05599	3.83E-20
TF	Factor: AP-2beta; motif: GCNNNGGSCNGVGGGN	TF:M01858	4.45E-20
TF	Factor: Klf15; motif: RGGGMGGRGNNGGGGGNGG	TF:M10277	4.82E-20
TF	Factor: E2F-4; motif: NGGGGGCGGGRMNN	TF:M10211	6.71E-20
TF	Factor: AP-2; motif: SNNNCCNCAGGCN	TF:M00915	1.13E-19
TF	Factor: Tfap2a; motif: TGCCCYNRGGGCA	TF:M04157	1.15E-19

TF	Factor: KLF3; motif: NNNNNNGGGCGGGGCNNGN	TF:M10278	2.14E-19
TF	Factor: CACD; motif: CCACRCCC; match class: 1	TF:M01113_1	2.75E-19
TF	Factor: PUR1; motif: GGGNCAGNN	TF:M01721	3.93E-19
TF	Factor: IRF6; motif: NNNACTCYCGGKNNN	TF:M02874	5.46E-19
TF	Factor: BTEB3; motif: BNRNGGGAGGNGT; match class: 1	TF:M01865_1	7.75E-19
TF	Factor: Hes1; motif: NNCACGYGNN	TF:M07991	9.10E-19
TF	Factor: Egr-2; motif: GCGTGGGCGG	TF:M05494	1.62E-18
TF	Factor: ZBP89; motif: CCCCKCCCCCNN; match class: 1	TF:M07397_1	2.25E-18
TF	Factor: VDR; motif: GGGKNARNRRGGWSA	TF:M00444	2.71E-18
TF	Factor: E2F-4; motif: NTTTCSCGCC	TF:M07380	2.87E-18
TF	Factor: PLAG1; motif: CCCCCKWNNNGGSCCC	TF:M01973	4.65E-18
TF	Factor: E2F-7; motif: GRGGCGGGAANNN	TF:M10212	4.71E-18
TF	Factor: ZAC; motif: KGGGCCR; match class: 1	TF:M05547_1	6.33E-18
TF	Factor: Osx; motif: CCNCCCCCNNN; match class: 1	TF:M07329_1	7.50E-18
TF	Factor: CKROX; motif: SCCCTCCCC; match class: 1	TF:M01175_1	1.06E-17
TF	Factor: Sp1; motif: GGGGCGGGGT	TF:M00008	1.21E-17
TF	Factor: KLF; motif: GGGNGGGG	TF:M07461	1.51E-17
TF	Factor: Sp1; motif: GGGGCGGGGC; match class: 1	TF:M00931_1	1.70E-17
TF	Factor: Pax-5; motif: BCNNNRNGCANBGNTGNRTAGCSGCHNB; m	TF:M00143_1	1.75E-17
TF	Factor: KROX; motif: CCCGCCCCCRCCCC; match class: 1	TF:M00982_1	1.76E-17
TF	Factor: WT1; motif: GNGGGGGCGGGG; match class: 1	TF:M03893_1	2.01E-17
TF	Factor: Sp5; motif: RNGGRGGNGGRGNNGGGGGAGGRG; match c	TF:M10376_1	2.13E-17
TF	Factor: Egr-1; motif: GCGGGGGCGG; match class: 1	TF:M01873_1	3.84E-17
TF	Factor: BTEB2; motif: GNAGGGGGNGGGSSNN	TF:M03814	4.77E-17
TF	Factor: Tfap2a; motif: TGCCCYNRGGGCA; match class: 1	TF:M04157_1	7.82E-17
TF	Factor: Spz1; motif: DNNGGRGGGWWNNNN; match class: 1	TF:M00446_1	1.14E-16
TF	Factor: MAZ; motif: NKGGGAGGGGRGGR	TF:M02023	1.23E-16
TF	Factor: WT1; motif: SMCNCCNSC; match class: 1	TF:M01118_1	2.03E-16
TF	Factor: WT1; motif: NNGGGNGGGSGN; match class: 1	TF:M07436_1	2.08E-16
TF	Factor: LKLF; motif: CNCCACCCS	TF:M08819	2.53E-16
TF	Factor: MAZ; motif: NKGGGAGGGGRGGR; match class: 1	TF:M02023_1	3.89E-16
TF	Factor: Tfap2a; motif: NGCCYSAGGCN	TF:M04156	4.35E-16
TF	Factor: LRF; motif: GGGGKYNNB	TF:M01100	5.38E-16
TF	Factor: KLF; motif: GGGNGGGG; match class: 1	TF:M07461_1	6.88E-16
TF	Factor: AP-2gamma; motif: GCCYNCRGSN	TF:M03811	1.03E-15
TF	Factor: Sp2; motif: NYSGCCCCGCCCCCY	TF:M03567	1.28E-15
TF	Factor: E2F-1; motif: NNNSSCGCSAANN	TF:M07250	1.85E-15
TF	Factor: HIC1; motif: NSNNNNTGCCCSSNN	TF:M01072	2.08E-15
TF	Factor: AP-2alpha; motif: NSCCNCRGGSN	TF:M07348	3.08E-15
TF	Factor: Sp1; motif: NGGGGGCGGGGYN; match class: 1	TF:M00196_1	4.43E-15
TF	Factor: Tfap2a; motif: NGCCYSAGGCN; match class: 1	TF:M04156_1	5.26E-15
TF	Factor: ZIC3; motif: NNNCACAGCAKGNNN; match class: 1	TF:M02941_1	5.88E-15
TF	Factor: Egr-2; motif: GCGTGGGCGG; match class: 1	TF:M05494_1	7.38E-15
TF	Factor: Zic3; motif: NGGGKGGTC; match class: 1	TF:M00450_1	8.45E-15
TF	Factor: VDR; motif: GGGKNARNRRGGWSA; match class: 1	TF:M00444_1	1.71E-14
TF	Factor: E2F-1; motif: TTGGCGCGRAANNGNM	TF:M00938	1.92E-14
TF	Factor: Zfp740; motif: NCCCCCCCAC	TF:M03944	2.09E-14
TF	Factor: AP-2; motif: MKCCCSCNGGCG; match class: 1	TF:M00189_1	2.67E-14
TF	Factor: FPM315; motif: SRGGGAGGAGGN	TF:M01587	3.37E-14
TF	Factor: EGR-1; motif: NGCGKGGGCGKNN	TF:M08996	3.50E-14

TF	Factor: Pax-5; motif: BCNNNRNGCANBGNTGNRTAGCSGCHNB	TF:M00143	4.66E-14
TF	Factor: E2F-2; motif: NNNANGGCGCGCNNN	TF:M02742	4.93E-14
TF	Factor: AP-2alpha; motif: GCCNNNRGS	TF:M00469	7.03E-14
TF	Factor: FKLF; motif: BGGGNGGVMD	TF:M01837	1.01E-13
TF	Factor: CPBP; motif: SNCCCNN; match class: 1	TF:M01822_1	1.18E-13
TF	Factor: SP1; motif: GGGGYGGGGNS; match class: 1	TF:M01303_1	1.28E-13
TF	Factor: IRF6; motif: NNNACTCYCGGKNNN; match class: 1	TF:M02874_1	1.78E-13
TF	Factor: Hes1; motif: NNCACGYGNN; match class: 1	TF:M07991_1	2.45E-13
TF	Factor: AP2; motif: GCCYGSGGSN; match class: 1	TF:M08867_1	3.37E-13
TF	Factor: CACD; motif: CCACRCCC	TF:M01113	4.16E-13
TF	Factor: RNF96; motif: BCCCGCRGCC; match class: 1	TF:M01199_1	4.60E-13
TF	Factor: AP-2; motif: GSCCSCRGGCNRNRNN; match class: 1	TF:M00800_1	1.53E-12
TF	Factor: GKLF; motif: CCTCCYN; match class: 1	TF:M01835_1	1.83E-12
TF	Factor: AP-2alphaA; motif: ANMGCCTNAGGCKNT	TF:M01047	2.07E-12
TF	Factor: AP-2alphaA; motif: ANMGCCTNAGGCKNT; match class: 1	TF:M01047_1	2.07E-12
TF	Factor: GKLF; motif: NNNRNWGGGTGKGGC	TF:M10279	2.48E-12
TF	Factor: Zic3; motif: NMCCCCCGGGGGGN	TF:M02837	3.13E-12
TF	Factor: AP-2beta; motif: GCNNNGGSCNGVGGGN; match class: 1	TF:M01858_1	7.26E-12
TF	Factor: Sp3; motif: AGGGCGG	TF:M05665	8.51E-12
TF	Factor: LKLF; motif: NGGGCGG	TF:M05391	8.51E-12
TF	Factor: CPBP; motif: NGGGCGG	TF:M05301	8.51E-12
TF	Factor: SP6; motif: WGGGCGG	TF:M05455	8.51E-12
TF	Factor: AP-2gamma; motif: GCCYNNGGS; match class: 1	TF:M00470_1	1.31E-11
TF	Factor: Sp1; motif: GGGGCGGGGT; match class: 1	TF:M00008_1	1.38E-11
TF	Factor: Zic2; motif: NMCCCCCGGGGGGN	TF:M02836	1.48E-11
TF	Factor: BEN; motif: CWGCGAYA	TF:M01241	1.60E-11
TF	Factor: NGFI-C; motif: WTGCGTGGGYGG	TF:M00244	2.06E-11
TF	Factor: E2F-1; motif: NNNSSCGCSAANN; match class: 1	TF:M07250_1	2.39E-11
TF	Factor: BTEB2; motif: RGGGNGKGGN; match class: 1	TF:M07277_1	2.79E-11
TF	Factor: KLF3; motif: NNNNNNGGGCGGGGCNNGN; match class: 1	TF:M10278_1	3.00E-11
TF	Factor: SP2; motif: GNNGGGGGCGGGGSN; match class: 1	TF:M03807_1	3.06E-11
TF	Factor: LKLF; motif: GGGGTGGKSN; match class: 1	TF:M07261_1	8.70E-11
TF	Factor: E2F-4; motif: GCGGGAAANA; match class: 1	TF:M02090_1	8.76E-11
TF	Factor: HIC1; motif: NNNGGKTGCCCSNNNNNN	TF:M01073	9.91E-11
TF	Factor: E2F-4; motif: GNNGGCGGGAAN	TF:M09682	1.10E-10
TF	Factor: Sp3; motif: ASMCTTGGGSRGGG	TF:M00665	1.11E-10
TF	Factor: ZBED6; motif: NRRGCTCGCCNN	TF:M01598	1.34E-10
TF	Factor: BTEB2; motif: GNAGGGGGNGGGSSNN; match class: 1	TF:M03814_1	1.35E-10
TF	Factor: MZF-1; motif: TGGGGAR	TF:M01733	1.70E-10
TF	Factor: CNOT3; motif: GGCCGCGSSS	TF:M01253	1.94E-10
TF	Factor: Zic3; motif: NGGGKGGTC	TF:M00450	2.00E-10
TF	Factor: E2F-1; motif: NKTSSCGC	TF:M00428	2.05E-10
TF	Factor: Zfp740; motif: NCCCCCCCAC; match class: 1	TF:M03944_1	2.29E-10
TF	Factor: HDAC1; motif: KGCARGGTC	TF:M07041	2.43E-10
TF	Factor: Egr; motif: GTGGGSGCRRS	TF:M00807	3.53E-10
TF	Factor: Sp4; motif: NNNNMCGCCCCCTNNNN	TF:M02810	3.87E-10
TF	Factor: SP2; motif: GGGCGGGAC	TF:M01783	7.98E-10
TF	Factor: SP1:SP3; motif: CCSCCCCCYCC; match class: 1	TF:M01219_1	9.14E-10
TF	Factor: MZF-1; motif: KNGNKAGGGGNAA; match class: 1	TF:M00084_1	9.38E-10
TF	Factor: Plagl1; motif: NNNGGGGSSCCCCNNN; match class: 1	TF:M02786_1	1.01E-09

TF	Factor: RREB-1; motif: CCCCAAACMMCCCC	TF:M00257	1.14E-09
TF	Factor: IRF4; motif: NNNAYTCTCGGWNNN	TF:M02872	1.18E-09
TF	Factor: Egr1; motif: NNMCGCCCMCTCAMWN	TF:M04506	1.18E-09
TF	Factor: EGR-1; motif: TGCGTGGGCGK	TF:M01972	1.63E-09
TF	Factor: PLAG1; motif: CCCCCKWNNNGGSCCC; match class: 1	TF:M01973_1	1.83E-09
TF	Factor: MAZ; motif: CCCTCCCYCYN	TF:M07297	2.57E-09
TF	Factor: E2F-4; motif: NTTTCSCGCC; match class: 1	TF:M07380_1	2.66E-09
TF	Factor: Plagl1; motif: NNNGGGGSSCCCCNNN	TF:M02786	2.89E-09
TF	Factor: Pax-4; motif: NNNNNYCACCCB	TF:M00378	2.93E-09
TF	Factor: MYF6; motif: NNNRACAGNCNCNCC; match class: 1	TF:M02885_1	3.54E-09
TF	Factor: PLAG1; motif: GRGGCNNHNNNRRGGG	TF:M01778	3.87E-09
TF	Factor: ZFP740; motif: NNNTWCCCCCCGGNRNN	TF:M02938	3.90E-09
TF	Factor: GKLF; motif: NNNRNWGGGTGKGGC; match class: 1	TF:M10279_1	5.21E-09
TF	Factor: TCFAP2B; motif: NTWGCCKSMGGCRNN	TF:M02924	6.37E-09
TF	Factor: SREBP-2; motif: NNGYCACNNSMN	TF:M01177	7.13E-09
TF	Factor: Egr-1; motif: NNGCGKGGGCGGGG; match class: 1	TF:M10213_1	8.32E-09
TF	Factor: IRF4; motif: NNNAYTCTCGGWNNN; match class: 1	TF:M02872_1	9.52E-09
TF	Factor: E2F-7; motif: GRGGCGGGAANNN; match class: 1	TF:M10212_1	9.68E-09
TF	Factor: Zic2; motif: NMCCCCCGGGGGGGN; match class: 1	TF:M02836_1	1.06E-08
TF	Factor: Sp4; motif: SCCCCKCCCCCSN	TF:M07617	1.23E-08
TF	Factor: Spz1; motif: DNNGGRGGGWWNNNN	TF:M00446	1.60E-08
TF	Factor: MYF6; motif: NNNRACAGNCNCNCC	TF:M02885	1.90E-08
TF	Factor: AP-2alpha; motif: NSCCNCRGGSN; match class: 1	TF:M07348_1	2.27E-08
TF	Factor: LF-A1; motif: GGGSTCWR; match class: 1	TF:M00646_1	2.68E-08
TF	Factor: MZF-1; motif: KNGNKAGGGGNAA	TF:M00084	3.57E-08
TF	Factor: GKLF; motif: WGGGYGKGGCCN	TF:M09692	4.04E-08
TF	Factor: ctcf; motif: CCNCNAGRKGGCRSTN	TF:M07249	4.09E-08
TF	Factor: TIEG1; motif: GGSGGKGNNN	TF:M08905	5.01E-08
TF	Factor: Tcfl5; motif: NNCNCGNGNN	TF:M08005	5.25E-08
TF	Factor: Tcfl5; motif: NNCNCGNGNN; match class: 1	TF:M08005_1	5.25E-08
TF	Factor: Pax-5; motif: RRMSWGANWYCTNRAGCGKRACSRYNSM	TF:M00144	5.48E-08
TF	Factor: GKLF; motif: CCTCCYN	TF:M01835	5.98E-08
TF	Factor: TFII-I; motif: RGAGGKAGG	TF:M00706	6.18E-08
TF	Factor: GKLF; motif: GCCMCRCCCNNN	TF:M01588	7.17E-08
TF	Factor: AP-2; motif: SNNNCCNCAGGCN; match class: 1	TF:M00915_1	7.35E-08
TF	Factor: ctcf; motif: YGGCCASCAGRGGGCGCYNN	TF:M10203	7.92E-08
TF	Factor: HSF4; motif: CTGCMRN	TF:M07322	8.98E-08
TF	Factor: SMAD5; motif: GSGGCAGM	TF:M03846	1.04E-07
TF	Factor: NRSF; motif: TTCAGCACCACGGACAGMGCC	TF:M00256	1.18E-07
TF	Factor: ZNF151; motif: GGSGGNGGGGGAGGGGMGG; match class	TF:M10412_1	1.19E-07
TF	Factor: AHR; motif: CACGCN; match class: 1	TF:M01855_1	1.65E-07
TF	Factor: AP-2alpha; motif: GCCNNNRGS; match class: 1	TF:M00469_1	1.69E-07
TF	Factor: REST; motif: YCAGCACCATGGACAGCNCCC; match class:	TF:M09705_1	1.95E-07
TF	Factor: HDAC1; motif: KGCARGGTC; match class: 1	TF:M07041_1	1.96E-07
TF	Factor: Zfp740; motif: NNNCCCCCCCCMNNNN	TF:M02834	2.08E-07
TF	Factor: ZN451; motif: CKGGGGGG	TF:M06457	3.00E-07
TF	Factor: SREBP-2; motif: NNGYCACNNSMN; match class: 1	TF:M01177_1	3.23E-07
TF	Factor: Egr-3; motif: NTGCGTGGGCGK	TF:M00245	3.37E-07
TF	Factor: Zic3; motif: NMCCCCCGGGGGGGN; match class: 1	TF:M02837_1	3.57E-07
TF	Factor: E2F-2; motif: NNNANGGCGCGCNNN; match class: 1	TF:M02742_1	3.62E-07

TF	Factor: Zfp281; motif: NNCCCCCCCCCCMYC	TF:M02831	3.99E-07
TF	Factor: DRRS; motif: GNNGGGWGGG	TF:M10363	4.17E-07
TF	Factor: GLI; motif: NGACCMCCCAN	TF:M07290	4.28E-07
TF	Factor: CP2/LBP-1c/LSF; motif: GCTGGNTNGNNCYNG; match clas	TF:M00947_1	4.67E-07
TF	Factor: c-Myc:Max; motif: GCCAYGYGSN	TF:M00322	4.92E-07
TF	Factor: REST; motif: KTCAGCACCAYGGACAGCKCCN	TF:M10354	5.43E-07
TF	Factor: E2F-3; motif: GGGCGGGARN	TF:M10210	6.51E-07
TF	Factor: LRF; motif: GGGGKYNNB; match class: 1	TF:M01100_1	7.21E-07
TF	Factor: Zfp740; motif: NNNCCCCCCCCMNNNN; match class: 1	TF:M02834_1	8.27E-07
TF	Factor: MZF-1; motif: TGGGGAR; match class: 1	TF:M01733_1	9.68E-07
TF	Factor: Zfp536; motif: NCGGAKG	TF:M05541	1.05E-06
TF	Factor: REST; motif: YCAGCACCATGGACAGCNCCC	TF:M09705	1.18E-06
TF	Factor: E2F; motif: NNTTTCGCGCN	TF:M08875	1.19E-06
TF	Factor: Egr-1; motif: WTGCGTGGGCGK	TF:M00243	1.58E-06
TF	Factor: PUR1; motif: GGGNCAGNN; match class: 1	TF:M01721_1	1.64E-06
TF	Factor: ELK-1; motif: ACCGGAWRTN	TF:M01981	1.65E-06
TF	Factor: RREB-1; motif: CCCCAAACMMCCCC; match class: 1	TF:M00257_1	1.84E-06
TF	Factor: NRSF; motif: GYRCTGTCCRYGGTGCTGA	TF:M01028	1.91E-06
TF	Factor: Tfap2a; motif: TGCCCYNRGGCA	TF:M04155	2.09E-06
TF	Factor: REST; motif: KTCAGCACCAYGGACAGCKCCN; match class	TF:M10354_1	2.45E-06
TF	Factor: HES-1; motif: GSCACGMGMC	TF:M02011	3.32E-06
TF	Factor: E2F-1; motif: TTGGCGCGRAANNGNM; match class: 1	TF:M00938_1	3.46E-06
TF	Factor: Tbx5; motif: SNAGGTGTGRNGGSS	TF:M07475	3.47E-06
TF	Factor: TIEG1; motif: GGSGGKGNNN; match class: 1	TF:M08905_1	3.77E-06
TF	Factor: FKLF; motif: BGGGNGGVMD; match class: 1	TF:M01837_1	5.34E-06
TF	Factor: E2F-1; motif: NKTSSCGC; match class: 1	TF:M00428_1	5.99E-06
TF	Factor: Sp2; motif: NYSGCCCCGCCCCCY; match class: 1	TF:M03567_1	6.59E-06
TF	Factor: WT1; motif: NGCGGGGGGGTSMMCYN; match class: 1	TF:M05599_1	1.53E-05
TF	Factor: LF-A1; motif: GGGSTCWR	TF:M00646	1.57E-05
TF	Factor: AhR:Arnt; motif: GRGKATYGCGTGMCWNSCC	TF:M00237	1.60E-05
TF	Factor: Klf7; motif: NNNRCCMCGCCCNNNN	TF:M02773	1.75E-05
TF	Factor: AP-2gamma; motif: GCCYNCRGSN; match class: 1	TF:M03811_1	2.21E-05
TF	Factor: LRF; motif: NGKGGGTSNCN	TF:M07387	2.26E-05
TF	Factor: MAZR; motif: NSGGGGGGGGMCN	TF:M00491	2.35E-05
TF	Factor: ctcf; motif: YGGCCASCAGRGGGCGCYNN; match class: 1	TF:M10203_1	2.40E-05
TF	Factor: ZBED6; motif: NRRGCTCGCCNN; match class: 1	TF:M01598_1	2.46E-05
TF	Factor: SREBP-1; motif: CACSCCA	TF:M00749	3.34E-05
TF	Factor: CP2; motif: GCHCDAMCCAG; match class: 1	TF:M00072_1	3.51E-05
TF	Factor: E2F-3; motif: NNNANGGCGCGCSNW	TF:M02743	3.60E-05
TF	Factor: E2F1; motif: NNNNNGCGSSAAAN	TF:M08874	3.91E-05
TF	Factor: ZNF515; motif: WNSCCCCCWGCNG	TF:M08835	4.47E-05
TF	Factor: Sp4; motif: NNNNMCGCCCCCTNNNN; match class: 1	TF:M02810_1	4.91E-05
TF	Factor: E2F-1; motif: TTTSGCGCGMNR	TF:M00516	5.18E-05
TF	Factor: CP2; motif: NNNNCCAGNCNN; match class: 1	TF:M07602_1	7.02E-05
TF	Factor: ER-beta; motif: RGGTCASCNTGMCCY	TF:M10227	7.87E-05
TF	Factor: p54NRB; motif: GRNNNMGGATGRMNNCGGA	TF:M07611	8.78E-05
TF	Factor: EGR-1; motif: NGCGKGGGCGKNN; match class: 1	TF:M08996_1	9.27E-05
TF	Factor: HIC1; motif: NSNNNNTGCCCSSNN; match class: 1	TF:M01072_1	9.71E-05
TF	Factor: E2F-4; motif: NGGGGGCGGGRMNN; match class: 1	TF:M10211_1	9.74E-05
TF	Factor: CACCC-binding; motif: CANCCNNWGGGTGDGG	TF:M00721	0.000105951

TF	Factor: TCFAP2B; motif: NTWGCCKSMGGCRNN; match class: 1	TF:M02924_1	0.000106584
TF	Factor: AP-2alpha; motif: NNNSCCTGRGGC	TF:M10175	0.000115873
TF	Factor: myogenin; motif: RGCAGSTG	TF:M00712	0.000129042
TF	Factor: CAC-binding; motif: GRGGSTGGG	TF:M00720	0.000135369
TF	Factor: ZIC3; motif: NNNCACAGCAKGNNN	TF:M02941	0.000138309
TF	Factor: SMAD4; motif: GKSRKKCAGMCANCY	TF:M00733	0.000139714
TF	Factor: GKLF; motif: WGGGYGKGGCCN; match class: 1	TF:M09692_1	0.000164808
TF	Factor: Sp4; motif: SCCCCKCCCCCSN; match class: 1	TF:M07617_1	0.000196071
TF	Factor: MRF4; motif: CASCTGC; match class: 1	TF:M03831_1	0.000212788
TF	Factor: HTF4; motif: CASCTGB; match class: 1	TF:M02018_1	0.000212788
TF	Factor: MyoD; motif: SCAGCTGYYNCNNNC	TF:M09698	0.00021365
TF	Factor: Pax-5; motif: RRNGRNGCAN; match class: 1	TF:M03577_1	0.000219049
TF	Factor: E12; motif: RRCAGGTGNCV	TF:M00693	0.000224939
TF	Factor: HES-1; motif: NNCKYGTGNNN	TF:M07042	0.000247512
TF	Factor: EGR-1; motif: TGCGTGGGCGK; match class: 1	TF:M01972_1	0.000257339
TF	Factor: Egr-2; motif: NTGCGTRGGCGK; match class: 1	TF:M00246_1	0.000258878
TF	Factor: Egr-2; motif: NTGCGTRGGCGK	TF:M00246	0.000260075
TF	Factor: Kid3; motif: CCACN; match class: 1	TF:M01160_1	0.000282707
TF	Factor: Zfp281; motif: TGGGGGAGGGG	TF:M01597	0.000292378
TF	Factor: Tbx5; motif: SNAGGTGTGRNGGSS; match class: 1	TF:M07475_1	0.000354663
TF	Factor: Ikaros; motif: TGGGAGN; match class: 1	TF:M07260_1	0.000359025
TF	Factor: NGFI-C; motif: WTGCGTGGGYGG; match class: 1	TF:M00244_1	0.000366823
TF	Factor: SMAD5; motif: GSGGCAGM; match class: 1	TF:M03846_1	0.000367608
TF	Factor: LKLF; motif: CNCCACCCS; match class: 1	TF:M08819_1	0.000383726
TF	Factor: Zic1; motif: KGGGTGGTC	TF:M00448	0.000392769
TF	Factor: CPBP; motif: SNCCCNN	TF:M01822	0.000400606
TF	Factor: GLI; motif: NSTGGGTGGTCY	TF:M10254	0.000404907
TF	Factor: Zfp57; motif: CTGCGGCARN	TF:M10416	0.000439424
TF	Factor: E2F-1; motif: TTTSGCGS	TF:M00431	0.000526956
TF	Factor: Pax-5; motif: RRMSWGANWYCTNRAGCGKRACSRYNSM; r	TF:M00144_1	0.000553818
TF	Factor: SP6; motif: WGGGCGG; match class: 1	TF:M05455_1	0.000569454
TF	Factor: LKLF; motif: NGGGCGG; match class: 1	TF:M05391_1	0.000569454
TF	Factor: Sp3; motif: AGGGCGG; match class: 1	TF:M05665_1	0.000569454
TF	Factor: CPBP; motif: NGGGCGG; match class: 1	TF:M05301_1	0.000569454
TF	Factor: BEN; motif: CWGCGAYA; match class: 1	TF:M01241_1	0.000679507
TF	Factor: Zfp281; motif: NNCCCCCCCCCCMYC; match class: 1	TF:M02831_1	0.000723027
TF	Factor: AHR; motif: CACGCN	TF:M01855	0.000787861
TF	Factor: CP2; motif: NNNNCCAGNCNN	TF:M07602	0.000796628
TF	Factor: GKLF; motif: GCCMCRCCCNNN; match class: 1	TF:M01588_1	0.000800596
TF	Factor: ZNF515; motif: WNSCCCCCWGCNG; match class: 1	TF:M08835_1	0.000843686
TF	Factor: AhR,; motif: NRCGTGNGN	TF:M00976	0.000858219
TF	Factor: Zic1; motif: KGGGTGGTC; match class: 1	TF:M00448_1	0.000873711
TF	Factor: SREBP-1; motif: CACSCCA; match class: 1	TF:M00749_1	0.000981772
TF	Factor: Tfap2a; motif: TGCCCYNRGGCA; match class: 1	TF:M04155_1	0.001119916
TF	Factor: Egr-3; motif: NTGCGTGGGCGK; match class: 1	TF:M00245_1	0.001212742
TF	Factor: PPARalpha:RXRalpha; motif: NNRGGTCATWGGGGTSANG	TF:M00518_1	0.001317894
TF	Factor: CP2/LBP-1c/LSF; motif: GCTGGNTNGNNCYNG	TF:M00947	0.001404776
TF	Factor: FPM315; motif: SRGGGAGGAGGN; match class: 1	TF:M01587_1	0.001551323
TF	Factor: Ikaros; motif: TGGGAGN	TF:M07260	0.001557507
TF	Factor: MAX; motif: NNGNCACGCGACNN	TF:M02881	0.001597746

TF	Factor: MASH-1; motif: CNSCASCTGCYNCN	TF:M10177	0.001701337
TF	Factor: ZBP89; motif: YCCYCCCCCM	TF:M01816	0.001706144
TF	Factor: AP-4; motif: NCAGCTGYNGNCN	TF:M01860	0.001706633
TF	Factor: DRRS; motif: GNNGGGWGGG; match class: 1	TF:M10363_1	0.001783892
TF	Factor: PPARalpha:RXRalpha; motif: NNRGGTCATWGGGGTSANG	TF:M00518	0.001819763
TF	Factor: BTEB2; motif: NGGGSWGGGNGGGGC	TF:M10280	0.001950343
TF	Factor: E2F-1; motif: NTTSGCGG	TF:M00430	0.002058907
TF	Factor: NF-1B; motif: CTGGCASGV	TF:M07051	0.002080299
TF	Factor: TFII-I; motif: RGAGGKAGG; match class: 1	TF:M00706_1	0.002207462
TF	Factor: OLIG2; motif: NCCAGCTGNTNNCNNNCNGN	TF:M09703	0.002234983
TF	Factor: ctcf; motif: YGGCCASYAGGGGGCGCYNN	TF:M09681	0.002538886
TF	Factor: c-Myc:Max; motif: GCCAYGYGSN; match class: 1	TF:M00322_1	0.002694127
TF	Factor: SREBP-2; motif: NTCACCYNNNN	TF:M03852	0.002844518
TF	Factor: E2F-2; motif: NNNWYGGCGCCAANNNN	TF:M02846	0.002846646
TF	Factor: Pet-1; motif: GCNGGAAGYG	TF:M10233	0.002912263
TF	Factor: NFKAPPAB; motif: NNGGGANTTCCCN	TF:M08891	0.003318303
TF	Factor: AP-2gamma; motif: NNNNWGCCYNCRGSCN	TF:M07349	0.00409395
TF	Factor: Egr-2; motif: CCCMCNCN; match class: 1	TF:M02092_1	0.004278711
TF	Factor: GLI; motif: MCVNNGACCACCCAV	TF:M03871	0.004441162
TF	Factor: p54NRB; motif: GRNNNMGGATGRMNNCGGA; match class:	TF:M07611_1	0.005662166
TF	Factor: MZF-1; motif: GKGGGGARNR	TF:M07361	0.006279945
TF	Factor: Egr1; motif: NCCGCCCCCGCANN; match class: 1	TF:M02744_1	0.006415188
TF	Factor: MAZ; motif: CCCTCCCYCYN; match class: 1	TF:M07297_1	0.006602535
TF	Factor: TWIST; motif: CACCTGG	TF:M03582	0.006643902
TF	Factor: E2F-1; motif: TTTSGCGS; match class: 1	TF:M00431_1	0.006766774
TF	Factor: E2A; motif: NRMCASCTGCNNN	TF:M02088	0.007546774
TF	Factor: LRF; motif: NGNAGNGGGTYN	TF:M04617	0.007664387
TF	Factor: Sp3; motif: ASMCTTGGGSRGGG; match class: 1	TF:M00665_1	0.008997014
TF	Factor: Klf17; motif: NGGGCGG	TF:M05407	0.00910552
TF	Factor: Klf7; motif: NNNRCCMCGCCCNNNN; match class: 1	TF:M02773_1	0.009346055
TF	Factor: GATA-1; motif: SNNGATNNNN	TF:M00075	0.010142028
TF	Factor: SMAD4; motif: GKSRKKCAGMCANCY; match class: 1	TF:M00733_1	0.010256513
TF	Factor: REST; motif: NNNNGGNGCTGTCCATGGTGCT	TF:M01256	0.011033053
TF	Factor: NR1B2; motif: NTGACCY	TF:M02111	0.011861295
TF	Factor: MAZR; motif: NSGGGGGGGGMCN; match class: 1	TF:M00491_1	0.012366539
TF	Factor: HES-1; motif: GSCACGMGMC; match class: 1	TF:M02011_1	0.012406278
TF	Factor: CTCF; motif: NNNGCCASCAGRKGGCRSNN	TF:M01200	0.012855439
TF	Factor: E47; motif: RCAGGTGY	TF:M07477	0.01387327
TF	Factor: GLI; motif: NTGGGTGGTN	TF:M07292	0.01503197
TF	Factor: E47; motif: RCAGGTGY; match class: 1	TF:M07477_1	0.016499989
TF	Factor: GLI1; motif: GACCACCCAV	TF:M01042	0.016561894
TF	Factor: HTF4; motif: CASCTGB	TF:M02018	0.018218364
TF	Factor: MRF4; motif: CASCTGC	TF:M03831	0.018218364
TF	Factor: KLF15; motif: GAGNNGGGGNGTDG	TF:M01714	0.01908677
TF	Factor: Egr; motif: GTGGGSGCRRS; match class: 1	TF:M00807_1	0.02086887
TF	Factor: LRF; motif: NRGGGKCKY	TF:M10413	0.021115241
TF	Factor: Prdm16; motif: YCCCAGGGRN	TF:M10348	0.023519786
TF	Factor: AhR:Arnt; motif: KNNKNNTYGCGTGCMS	TF:M00235	0.023705653
TF	Factor: E2F; motif: TTTSGCGSG	TF:M00939	0.024713838
TF	Factor: Klf15; motif: RGGGMGGRGNNGGGGGNGG; match class: 1	TF:M10277_1	0.025487122

TF	Factor: E2A; motif: CACCTGNY	TF:M00973	0.026454709
TF	Factor: DEC1; motif: NCNCACRTGNSC	TF:M08870	0.027539782
TF	Factor: MTF-1; motif: TGCGCAC	TF:M01243	0.028058513
TF	Factor: LRF; motif: NGNAGNGGGTYN; match class: 1	TF:M04617_1	0.028233154
TF	Factor: GLI; motif: NSTGGGTGGTCY; match class: 1	TF:M10254_1	0.028371515
TF	Factor: SP100; motif: NNCGTCGNNTAAWNN	TF:M02913	0.029384996
TF	Factor: Egr-2; motif: NGNGTGGGNGGGGG	TF:M10214	0.0300362
TF	Factor: ctcf; motif: CCNCNAGRKGGCRSTN; match class: 1	TF:M07249_1	0.030278566
TF	Factor: REST; motif: NNNNGGNGCTGTCCATGGTGCT; match class	TF:M01256_1	0.031155223
TF	Factor: HIC1; motif: NNNGGKTGCCCSNNNNNN; match class: 1	TF:M01073_1	0.032750535
TF	Factor: PTF1; motif: SCAGCTGNYNNNNYYCNN	TF:M10351	0.034296838
TF	Factor: AP-4; motif: WGARYCAGCTGYGGNCNK	TF:M00005	0.034804026
TF	Factor: NRSE; motif: TTYAGCWCCDCGGASAGYRCC	TF:M00325	0.034854151
TF	Factor: ERR1; motif: CAAGGTCAMNN	TF:M02093	0.038016108
TF	Factor: LRF; motif: NGKGGGTSNCN; match class: 1	TF:M07387_1	0.041875851
TF	Factor: GLI; motif: NGACCMCCCAN; match class: 1	TF:M07290_1	0.046801893
TF	Factor: EBF; motif: KTCCCYWGRGA	TF:M00977	0.049531245
MIRNA	mmu-miR-7b-5p	MIRNA:mmu-miF	3.38E-06
MIRNA	mmu-miR-149-5p	MIRNA:mmu-miF	4.01E-06
MIRNA	mmu-miR-9-5p	MIRNA:mmu-miF	1.45E-05
MIRNA	mmu-miR-181a-5p	MIRNA:mmu-miF	0.001218006
MIRNA	mmu-miR-34b-5p	MIRNA:mmu-miF	0.002697905
MIRNA	mmu-miR-541-5p	MIRNA:mmu-miF	0.004178482
MIRNA	mmu-miR-136-5p	MIRNA:mmu-miF	0.023183188
MIRNA	mmu-miR-24-3p	MIRNA:mmu-miF	0.02730371
MIRNA	mmu-miR-758-3p	MIRNA:mmu-miF	0.036302971
CORUM	Profilin 2 complex	CORUM:2835	0.007484381
CORUM	GluR delta-2 complex, postsynaptic	CORUM:1913	0.049969428
HP	Non-motor seizure	HP:0033259	3.18E-10
HP	Abnormality of esophagus physiology	HP:0025270	4.13E-10
HP	Involuntary movements	HP:0004305	6.69E-10
HP	Autistic behavior	HP:0000729	1.44E-09
HP	Focal non-motor seizure	HP:0032679	3.06E-08
HP	Status epilepticus	HP:0002133	3.13E-08
HP	Myoclonus	HP:0001336	3.91E-08
HP	Gastroesophageal reflux	HP:0002020	8.60E-08
HP	Functional abnormality of the gastrointestinal tract	HP:0012719	1.62E-07
HP	Abnormal aggressive, impulsive or violent behavior	HP:0006919	1.71E-07
HP	Generalized non-motor (absence) seizure	HP:0002121	2.22E-07
HP	Dialeptic seizure	HP:0011146	1.44E-06
HP	Self-injurious behavior	HP:0100716	1.50E-06
HP	Dysphagia	HP:0002015	2.71E-06
HP	Generalized myoclonic seizure	HP:0002123	3.01E-06
HP	Generalized-onset motor seizure	HP:0032677	3.15E-06
HP	Seizure	HP:0001250	3.24E-06
HP	Abnormal central motor function	HP:0011442	3.83E-06
HP	Stereotypy	HP:0000733	4.48E-06
HP	Abnormality of higher mental function	HP:0011446	5.89E-06
HP	Eyelid myoclonus	HP:0025097	7.15E-06
HP	Intellectual disability	HP:0001249	8.46E-06

HP	Generalized-onset seizure	HP:0002197	8.51E-06
HP	Behavioral abnormality	HP:0000708	1.72E-05
HP	Interictal epileptiform activity	HP:0011182	1.94E-05
HP	Interictal EEG abnormality	HP:0025373	1.94E-05
HP	Generalized tonic seizure	HP:0010818	2.46E-05
HP	Myoclonic seizure	HP:0032794	2.94E-05
HP	Bilateral tonic-clonic seizure	HP:0002069	4.36E-05
HP	Motor seizure	HP:0020219	4.57E-05
HP	EEG with generalized epileptiform discharges	HP:0011198	4.87E-05
HP	Focal emotional seizure	HP:0025613	5.08E-05
HP	Focal emotional seizure with laughing	HP:0010821	5.08E-05
HP	Spasticity	HP:0001257	7.73E-05
HP	EEG abnormality	HP:0002353	0.000106758
HP	Hypsarrhythmia	HP:0002521	0.000113725
HP	Tonic seizure	HP:0032792	0.00011452
HP	Bilateral generalized polymicrogyria	HP:0032410	0.000121613
HP	Focal-onset seizure	HP:0007359	0.00016207
HP	Abnormality of central nervous system electrophysiology	HP:0030178	0.000193909
HP	Epileptic encephalopathy	HP:0200134	0.000206469
HP	Atonic seizure	HP:0010819	0.000209243
HP	Abnormal emotion/affect behavior	HP:0100851	0.000212001
HP	Neurodevelopmental abnormality	HP:0012759	0.000266458
HP	Mental deterioration	HP:0001268	0.000316335
HP	Hypertonia	HP:0001276	0.00043945
HP	Epileptic spasm	HP:0011097	0.00048316
HP	Abnormal morphology of the hippocampus	HP:0025100	0.000524855
HP	Dyskinesia	HP:0100660	0.000565418
HP	Abnormality of movement	HP:0100022	0.000599591
HP	Tremor	HP:0001337	0.000642416
HP	Abnormal morphology of the limbic system	HP:0007343	0.000657317
HP	Typical absence seizure	HP:0011147	0.000792244
HP	Upper motor neuron dysfunction	HP:0002493	0.001038194
HP	Neurological speech impairment	HP:0002167	0.001134877
HP	Oculogyric crisis	HP:0010553	0.001539306
HP	Abnormal subarachnoid space morphology	HP:0012703	0.001613404
HP	Cerebral atrophy	HP:0002059	0.001634029
HP	Oral-pharyngeal dysphagia	HP:0200136	0.002092686
HP	Abnormality of eye movement	HP:0000496	0.002105416
HP	Atrophy/Degeneration affecting the cerebrum	HP:0007369	0.003304353
HP	Autism	HP:0000717	0.00405116
HP	Sleep disturbance	HP:0002360	0.004673599
HP	Focal impaired awareness seizure	HP:0002384	0.00508281
HP	Abnormal nervous system electrophysiology	HP:0001311	0.00513071
HP	Unsteady gait	HP:0002317	0.006543775
HP	Abnormality of coordination	HP:0011443	0.00654441
HP	Hyperactivity	HP:0000752	0.006575175
HP	Chorea	HP:0002072	0.006759143
HP	Focal motor seizure	HP:0011153	0.006992
HP	Global developmental delay	HP:0001263	0.008790393
HP	EEG with multifocal slow activity	HP:0010844	0.011143753

HP	Ataxia	HP:0001251	0.01171129
HP	Aggressive behavior	HP:0000718	0.012030041
HP	Abnormal muscle tone	HP:0003808	0.012628867
HP	Abnormality of the cerebral white matter	HP:0002500	0.013081102
HP	Brain atrophy	HP:0012444	0.013155094
HP	Widened subarachnoid space	HP:0012704	0.016100195
HP	Delayed speech and language development	HP:0000750	0.016543796
HP	Atrophy/Degeneration affecting the central nervous system	HP:0007367	0.01716935
HP	EEG with abnormally slow frequencies	HP:0011203	0.017742165
HP	Abnormality of the gastrointestinal tract	HP:0011024	0.017804197
HP	Abnormal corpus callosum morphology	HP:0001273	0.026576626
HP	Abnormal nervous system physiology	HP:0012638	0.028888935
HP	Generalized hypotonia	HP:0001290	0.030745379
HP	Absent speech	HP:0001344	0.037617052
HP	Infantile spasms	HP:0012469	0.039227758
HP	Developmental regression	HP:0002376	0.040024517
HP	Precocious puberty	HP:0000826	0.040918186
HP	Impairment in personality functioning	HP:0031466	0.042824608
HP	Speech apraxia	HP:0011098	0.045921699

Gene Ontology Terms- Upregulated Terms/Processes in the KO

source	term_name
GO:MF	nucleic acid binding
GO:MF	binding
GO:MF	DNA binding
GO:MF	heterocyclic compound binding
GO:MF	organic cyclic compound binding
GO:MF	transcription regulator activity
GO:MF	protein binding
GO:MF	RNA polymerase II transcription regulatory region sequence-specific DNA binding
GO:MF	transcription regulatory region sequence-specific DNA binding
GO:MF	regulatory region nucleic acid binding
GO:MF	DNA-binding transcription factor activity, RNA polymerase II-specific
GO:MF	ion binding
GO:MF	sequence-specific double-stranded DNA binding
GO:MF	DNA-binding transcription factor activity
GO:MF	double-stranded DNA binding
GO:MF	cis-regulatory region sequence-specific DNA binding
GO:MF	RNA polymerase II cis-regulatory region sequence-specific DNA binding
GO:MF	sequence-specific DNA binding
GO:MF	cation binding
GO:MF	metal ion binding
GO:MF	RNA binding
GO:BP	primary metabolic process
GO:BP	cellular metabolic process
GO:BP	nitrogen compound metabolic process
GO:BP	regulation of primary metabolic process
GO:BP	nucleic acid metabolic process
GO:BP	regulation of metabolic process
GO:BP	RNA metabolic process
GO:BP	cellular nitrogen compound metabolic process
GO:BP	regulation of macromolecule metabolic process
GO:BP	heterocycle metabolic process
GO:BP	macromolecule metabolic process
GO:BP	regulation of cellular metabolic process
GO:BP	cellular aromatic compound metabolic process
GO:BP	nucleobase-containing compound metabolic process
GO:BP	regulation of nitrogen compound metabolic process
GO:BP	organic cyclic compound metabolic process
GO:BP	cellular macromolecule metabolic process
GO:BP	organic substance metabolic process
GO:BP	macromolecule biosynthetic process
GO:BP	regulation of macromolecule biosynthetic process
GO:BP	gene expression
GO:BP	regulation of nucleobase-containing compound metabolic process
GO:BP	regulation of biosynthetic process
GO:BP	cellular macromolecule biosynthetic process
GO:BP	biosynthetic process
GO:BP	organic substance biosynthetic process
GO:BP	regulation of cellular macromolecule biosynthetic process

GO:BP	metabolic process
GO:BP	regulation of RNA metabolic process
GO:BP	regulation of cellular biosynthetic process
GO:BP	cellular biosynthetic process
GO:BP	regulation of gene expression
GO:BP	cellular nitrogen compound biosynthetic process
GO:BP	RNA biosynthetic process
GO:BP	nucleobase-containing compound biosynthetic process
GO:BP	nucleic acid-templated transcription
GO:BP	regulation of nucleic acid-templated transcription
GO:BP	regulation of RNA biosynthetic process
GO:BP	heterocycle biosynthetic process
GO:BP	transcription, DNA-templated
GO:BP	aromatic compound biosynthetic process
GO:BP	regulation of transcription, DNA-templated
GO:BP	organic cyclic compound biosynthetic process
GO:BP	transcription by RNA polymerase II
GO:BP	regulation of transcription by RNA polymerase II
GO:BP	ribonucleoprotein complex biogenesis
GO:BP	positive regulation of cellular metabolic process
GO:BP	chromosome organization
GO:BP	cellular component biogenesis
GO:BP	positive regulation of nitrogen compound metabolic process
GO:BP	lens development in camera-type eye
GO:BP	cellular component organization or biogenesis
GO:BP	positive regulation of metabolic process
GO:BP	positive regulation of macromolecule metabolic process
GO:BP	negative regulation of macromolecule biosynthetic process
GO:BP	eye development
GO:BP	visual system development
GO:BP	ribosome biogenesis
GO:BP	sensory system development
GO:BP	cellular protein metabolic process
GO:BP	RNA processing
GO:BP	negative regulation of biosynthetic process
GO:BP	chromatin organization
GO:BP	chromatin assembly or disassembly
GO:BP	negative regulation of cellular biosynthetic process
GO:BP	cellular process
GO:BP	negative regulation of cellular macromolecule biosynthetic process
GO:BP	negative regulation of macromolecule metabolic process
GO:BP	negative regulation of metabolic process
GO:BP	negative regulation of nucleobase-containing compound metabolic process
GO:BP	protein metabolic process
GO:BP	lens fiber cell differentiation
GO:BP	camera-type eye development
GO:BP	animal organ development
GO:BP	positive regulation of macromolecule biosynthetic process
GO:BP	negative regulation of cellular process
GO:BP	positive regulation of nucleobase-containing compound metabolic process

GO:BP	rRNA processing
GO:BP	negative regulation of RNA metabolic process
GO:BP	negative regulation of cellular metabolic process
GO:BP	rRNA metabolic process
GO:BP	negative regulation of nucleic acid-templated transcription
GO:BP	negative regulation of nitrogen compound metabolic process
GO:BP	negative regulation of RNA biosynthetic process
GO:BP	chromatin assembly
GO:BP	positive regulation of cellular process
GO:BP	negative regulation of biological process
GO:BP	positive regulation of biosynthetic process
GO:BP	negative regulation of gene expression
GO:BP	positive regulation of RNA metabolic process
GO:BP	macromolecule catabolic process
GO:BP	cellular protein modification process
GO:BP	protein modification process
GO:BP	macromolecule modification
GO:BP	positive regulation of cellular biosynthetic process
GO:BP	cellular component organization
GO:BP	organelle organization
GO:BP	eye morphogenesis
GO:BP	DNA packaging
GO:BP	fat cell differentiation
GO:BP	organonitrogen compound metabolic process
GO:CC	nucleus
GO:CC	intracellular anatomical structure
GO:CC	intracellular organelle
GO:CC	intracellular membrane-bounded organelle
GO:CC	organelle
GO:CC	membrane-bounded organelle
GO:CC	nuclear lumen
GO:CC	intracellular organelle lumen
GO:CC	organelle lumen
GO:CC	membrane-enclosed lumen
GO:CC	nucleoplasm
GO:CC	nucleolus
GO:CC	intracellular non-membrane-bounded organelle
GO:CC	non-membrane-bounded organelle
GO:CC	cytoplasm
GO:CC	nuclear body
GO:CC	DNA packaging complex
GO:CC	cellular anatomical entity
GO:CC	protein-containing complex
GO:CC	chromatin
GO:CC	nucleosome
GO:CC	protein-DNA complex
GO:CC	cytosol
GO:CC	nuclear speck
GO:CC	extracellular matrix
GO:CC	collagen-containing extracellular matrix

GO:CC	preribosome
KEGG	Ribosome biogenesis in eukaryotes
KEGG	Herpes simplex virus 1 infection
REAC	RNA Polymerase II Transcription
REAC	Generic Transcription Pathway
REAC	Gene expression (Transcription)
REAC	Activated PKN1 stimulates transcription of AR (androgen receptor) regulated genes KL
REAC	Senescence-Associated Secretory Phenotype (SASP)
REAC	RUNX1 regulates genes involved in megakaryocyte differentiation and platelet function
REAC	DNA Damage/Telomere Stress Induced Senescence
REAC	Condensation of Prophase Chromosomes
REAC	RNA Polymerase I Promoter Opening
REAC	Cellular Senescence
REAC	RHO GTPases activate PKNs
REAC	Chromatin organization
REAC	Chromatin modifying enzymes
REAC	Laminin interactions
REAC	PRC2 methylates histones and DNA
REAC	Mitotic Prophase
TF	Factor: FOXN4; motif: NNWANNCGWMCGCGTCNNNNMT
TF	Factor: ZF5; motif: GSGCGCGR
TF	Factor: FOXN4; motif: NNWANNCGWMCGCGTCNNNNMT; match class: 1
TF	Factor: ZF5; motif: GSGCGCGR; match class: 1
TF	Factor: E2F-4; motif: NTTTCSCGCC
TF	Factor: ZF5; motif: NRNGNGCGCGCWN
TF	Factor: Kaiso; motif: SARNYCTCGCGAGAN
TF	Factor: E2F; motif: GGCGSG
TF	Factor: E2F-1; motif: GNGGGCGGGRMN
TF	Factor: ZF5; motif: NRNGNGCGCGCWN; match class: 1
TF	Factor: IRF6; motif: NNNACTCYCGGKNNN
TF	Factor: ZF5; motif: GYCGCGCARNGCNN
TF	Factor: Pax-5; motif: RRMSWGANWYCTNRAGCGKRACSRYNSM
TF	Factor: BEN; motif: CAGCGRNV
TF	Factor: Elk-1; motif: RACCGGAAGTR
TF	Factor: E2F-1; motif: GNGGGCGGGRMN; match class: 1
TF	Factor: ELK-1; motif: ACCGGAWRTN
TF	Factor: TEL1; motif: CNCGGAANNN
TF	Factor: BEN; motif: CAGCGRNV; match class: 1
TF	Factor: Kaiso; motif: SARNYCTCGCGAGAN; match class: 1
TF	Factor: ELK-1; motif: ACCGGAWRTN; match class: 1
TF	Factor: E2F; motif: GGCGSG; match class: 1
TF	Factor: Elk-1; motif: AACCGGAAGTR
TF	Factor: E2F-1; motif: NKTSSCGC
TF	Factor: Pax-3; motif: NNNNNNCGTCACGSTYNNNNN; match class: 1
TF	Factor: E2F; motif: NNTTTCGCGCN
TF	Factor: Elk-1; motif: NNNNCCGGAARTNN
TF	Factor: c-ets-1; motif: ACCGGAWRYN
TF	Factor: E2F-1; motif: NNNSSCGCSAANN
TF	Factor: E2F-3; motif: GGCGGGN
TF	Factor: E2F-7; motif: GRGGCGGGAANNN

TF Factor: E2F-1; motif: TTTSGCGS
TF Factor: Elk-1; motif: NNNNCCGGAARTNN; match class: 1
TF Factor: E2F1; motif: NNNNNGCGSSAAAN
TF Factor: GABPalpha; motif: ACCGGAARYN
TF Factor: BEN; motif: CWGCGAYA
TF Factor: ERF; motif: ACCGGAARTN
TF Factor: E2F-4; motif: GCGGGAAANA
TF Factor: RNF96; motif: BCCCGCRGCC
TF Factor: ERG; motif: ACCGGAART
TF Factor: SP100; motif: NNCGTCGNNTAAWNN
TF Factor: Elk-1; motif: NCCGGAAGTGN
TF Factor: Hes1; motif: NNCACGYGNN
TF Factor: E2F-4; motif: NTTTCSCGCC; match class: 1
TF Factor: SP100; motif: NNCGTCGNNTAAWNN; match class: 1
TF Factor: E2F-4; motif: GNNGGCGGGAAN
TF Factor: c-Ets-1; motif: NNNRCCGGAWRYNNNN
TF Factor: GABP-alpha; motif: GGNRCCGGAAGTGN
TF Factor: IRF4; motif: NNNAYTCTCGGWNNN
TF Factor: Pax-5; motif: RRMSWGANWYCTNRAGCGKRACSRYNSM; match class: 1
TF Factor: Sp1; motif: NGGGGCGGGGN
TF Factor: PEA3; motif: RCCGGAAGYN; match class: 1
TF Factor: CTCF; motif: NAGGGGGCGCNNKNNNN
TF Factor: E2F-4; motif: NGGGGGCGGGRMNN
TF Factor: CPBP; motif: SNCCCNN; match class: 1
TF Factor: PEA3; motif: RCCGGAAGYN
TF Factor: c-Myc:Max; motif: GCCAYGYGSN
TF Factor: Sp1; motif: NNGGGGCGGGGNN
TF Factor: Atf-1; motif: RTGACGTA
TF Factor: Pax-3; motif: NNNNNNCGTCACGSTYNNNNN
TF Factor: Sp1; motif: GGGGCGGGGT
TF Factor: ER81; motif: RCCGGAWRYN
TF Factor: CPBP; motif: NGGGCGG
TF Factor: SP6; motif: WGGGCGG
TF Factor: LKLF; motif: NGGGCGG
TF Factor: Sp3; motif: AGGGCGG
TF Factor: Sp1; motif: GGGGCGGGGC
TF Factor: ZBED6; motif: NRRGCTCGCCNN
TF Factor: Sp1; motif: NGGGGGCGGGGYN
TF Factor: Hes1; motif: NNCACGYGNN; match class: 1
TF Factor: SP2; motif: GGGCGGGAC
TF Factor: E2F-3; motif: GGCGGGN; match class: 1
TF Factor: IRF4; motif: NNNAYTCTCGGWNNN; match class: 1
TF Factor: Ets2; motif: ACCGGAWRYN
TF Factor: KLF3; motif: NNNNNNGGGCGGGGCNNGN; match class: 1
TF Factor: Egr-2; motif: GCGTGGGCGG
TF Factor: CREB; motif: NSTGACGTAANN
TF Factor: KLF3; motif: NNNNNNGGGCGGGGCNNGN
TF Factor: Sp1; motif: CCCCGCCCCN
TF Factor: ELF4; motif: CCCGGAARTN
TF Factor: Kaiso; motif: GCMGGGRGCRGS

TF	Factor: Tcfl5; motif: NNCNCGNGNN
TF	Factor: Tcfl5; motif: NNCNCGNGNN; match class: 1
TF	Factor: CREM; motif: TGACGTCASYN
TF	Factor: E2F-2; motif: NNNANGGCGCGCNNN
TF	Factor: IRF6; motif: NNNACTCYCGGKNNN; match class: 1
TF	Factor: XBP-1; motif: WNNGMCACGTC; match class: 1
TF	Factor: E2F-2; motif: NNNWYGGCGCCAANNNN
TF	Factor: E2F-1; motif: NNNSSCGCSAANN; match class: 1
TF	Factor: E2F-3; motif: NNNWYGGCGCCAMNNNN; match class: 1
TF	Factor: E2F-1; motif: TTGGCGCGRAANNGNM
TF	Factor: Sp2; motif: NYSGCCCCGCCCCCY
TF	Factor: Ehf; motif: NNNANCCGGAAGTNN
TF	Factor: E2F; motif: NKCGCGCSAAAN; match class: 1
TF	Factor: BCL6B; motif: NNNNCCGCCCCWNNNN
TF	Factor: Elk-1; motif: NCCGGAAGTGN; match class: 1
TF	Factor: ZAC; motif: KGGGCCR
TF	Factor: Fli-1; motif: GNNRCCGGAAGYGS
TF	Factor: MAZ; motif: NKGGGAGGGGRGGR
TF	Factor: E2F-1; motif: TTTSGCGCGMNR
TF	Factor: CTCF; motif: NAGGGGGCGCNNKNNNN; match class: 1
TF	Factor: GABP-alpha; motif: CTTCCK
TF	Factor: ATF2; motif: VGTGACGTMACN
TF	Factor: ERG; motif: ACCGGAARYN
TF	Factor: Kid3; motif: CCACN; match class: 1
TF	Factor: SP2; motif: GNNGGGGGCGGGGSN
TF	Factor: AhR,; motif: NRCGTGNGN
TF	Factor: nerf; motif: NCCGGAARTN
TF	Factor: ZF5; motif: GYCGCGCARNGCNN; match class: 1
TF	Factor: BTEB2; motif: RGGGNGKGGN
TF	Factor: GKLF; motif: NNNRGGNGNGGSN
TF	Factor: NRF-1; motif: YGCGCMTGCGC
TF	Factor: Sp1; motif: GGNGGGGGNGGGGGMGGGGCNGGG; match class: 1
TF	Factor: ETV3; motif: ANCGGAARYN
TF	Factor: CREB,; motif: NTGACGTNA
TF	Factor: BCL6B; motif: NNNNCCGCCCCWNNNN; match class: 1
TF	Factor: CPBP; motif: SNCCCNN
TF	Factor: E2F-1; motif: NKTSSCGC; match class: 1
TF	Factor: Elf-1; motif: AWCCCGGAAGTN
TF	Factor: CREB; motif: TGACGTMA
TF	Factor: Klf7; motif: NNNRCCMCGCCCNNNN
TF	Factor: AP-2; motif: MKCCCSCNGGCG
TF	Factor: Pet-1; motif: ACCGGAARYN
TF	Factor: E2F-3; motif: GGGCGGGARN
TF	Factor: AhR:Arnt; motif: GRGKATYGCGTGMCWNSCC
TF	Factor: Ets2; motif: ACCGGAWRYN; match class: 1
TF	Factor: GABPalpha_GABPbeta; motif: CTTCCKGY
TF	Factor: Sp1; motif: NGGGGCGGGGN; match class: 1
TF	Factor: BTEB2; motif: RGGGNGKGGN; match class: 1
TF	Factor: Atf-1; motif: RTGACGTA; match class: 1
TF	Factor: E2F-1; motif: TWSGCGCGAAAAYKR; match class: 1

TF	Factor: AhR,; motif: NRCGTGNGN; match class: 1
TF	Factor: Klf17; motif: NGGGCGG
TF	Factor: Pet-1; motif: GCNGGAAGYG
TF	Factor: ELF1; motif: NCCGGAARTN
TF	Factor: E2F-1; motif: TWSGCGCGAAAAYKR
TF	Factor: GKLF; motif: NNRRGRRNGNSNNN
TF	Factor: Elf-1; motif: NNANCCGGAAGTGN
TF	Factor: AP-2alpha; motif: NGCCYSNNGSN
TF	Factor: Irf-4; motif: NNNANCGAAACYNNA
HP	Cataract
HP	Abnormality of the lens
HP	Microcornea

term_id	adjusted_p_value
GO:0003676	8.05E-12
GO:0005488	3.44E-10
GO:0003677	2.13E-07
GO:1901363	8.25E-07
GO:0097159	2.28E-06
GO:0140110	4.89E-06
GO:0005515	5.76E-06
GO:0000977	1.62E-05
GO:0000976	2.52E-05
GO:0001067	2.73E-05
GO:0000981	2.87E-05
GO:0043167	5.64E-05
GO:1990837	6.94E-05
GO:0003700	0.000117714
GO:0003690	0.000120328
GO:0000987	0.00016812
GO:0000978	0.00017246
GO:0043565	0.000292873
GO:0043169	0.000336015
GO:0046872	0.000375312
GO:0003723	0.020940535
GO:0044238	7.70E-17
GO:0044237	1.08E-15
GO:0006807	1.93E-15
GO:0080090	2.37E-15
GO:0090304	3.35E-15
GO:0019222	6.57E-15
GO:0016070	8.96E-15
GO:0034641	9.54E-15
GO:0060255	1.29E-14
GO:0046483	1.58E-14
GO:0043170	1.59E-14
GO:0031323	1.86E-14
GO:0006725	2.02E-14
GO:0006139	2.54E-14
GO:0051171	1.37E-13
GO:1901360	1.82E-13
GO:0044260	2.21E-13
GO:0071704	4.22E-13
GO:0009059	5.31E-13
GO:0010556	8.17E-13
GO:0010467	1.21E-12
GO:0019219	1.52E-12
GO:0009889	1.57E-12
GO:0034645	1.74E-12
GO:0009058	3.80E-12
GO:1901576	4.12E-12
GO:2000112	5.88E-12

GO:0008152	8.94E-12
GO:0051252	9.40E-12
GO:0031326	1.09E-11
GO:0044249	3.15E-11
GO:0010468	8.36E-10
GO:0044271	3.49E-09
GO:0032774	7.41E-09
GO:0034654	9.57E-09
GO:0097659	1.12E-08
GO:1903506	1.52E-08
GO:2001141	1.66E-08
GO:0018130	3.52E-08
GO:0006351	3.52E-08
GO:0019438	4.58E-08
GO:0006355	8.89E-08
GO:1901362	1.02E-07
GO:0006366	1.11E-07
GO:0006357	2.10E-07
GO:0022613	9.75E-07
GO:0031325	1.20E-05
GO:0051276	2.19E-05
GO:0044085	4.44E-05
GO:0051173	5.18E-05
GO:0002088	5.26E-05
GO:0071840	8.42E-05
GO:0009893	0.000190711
GO:0010604	0.00031656
GO:0010558	0.00034956
GO:0001654	0.000558518
GO:0150063	0.000654257
GO:0042254	0.000845413
GO:0048880	0.000848343
GO:0044267	0.000863094
GO:0006396	0.000880444
GO:0009890	0.000924255
GO:0006325	0.001289342
GO:0006333	0.001330895
GO:0031327	0.001348418
GO:0009987	0.001394933
GO:2000113	0.002129014
GO:0010605	0.00238901
GO:0009892	0.002471586
GO:0045934	0.003726161
GO:0019538	0.003737276
GO:0070306	0.004493631
GO:0043010	0.005173456
GO:0048513	0.005935899
GO:0010557	0.007555322
GO:0048523	0.007823983
GO:0045935	0.009788308

GO:0006364	0.010901317
GO:0051253	0.011824018
GO:0031324	0.018967438
GO:0016072	0.021056607
GO:1903507	0.021616307
GO:0051172	0.021980592
GO:1902679	0.022089121
GO:0031497	0.022129207
GO:0048522	0.02262212
GO:0048519	0.023082064
GO:0009891	0.023914195
GO:0010629	0.02456312
GO:0051254	0.026175467
GO:0009057	0.028294049
GO:0006464	0.029756281
GO:0036211	0.029756281
GO:0043412	0.033217716
GO:0031328	0.033755025
GO:0016043	0.036046692
GO:0006996	0.040194576
GO:0048592	0.040313036
GO:0006323	0.042617634
GO:0045444	0.043865671
GO:1901564	0.049337835
GO:0005634	4.96E-27
GO:0005622	2.27E-24
GO:0043229	1.04E-17
GO:0043231	1.90E-17
GO:0043226	5.34E-15
GO:0043227	9.79E-15
GO:0031981	4.81E-11
GO:0070013	2.20E-10
GO:0043233	2.93E-10
GO:0031974	2.93E-10
GO:0005654	2.16E-08
GO:0005730	2.01E-07
GO:0043232	6.52E-06
GO:0043228	7.79E-06
GO:0005737	6.07E-05
GO:0016604	0.000263655
GO:0044815	0.003522471
GO:0110165	0.003821549
GO:0032991	0.004579369
GO:0000785	0.007182553
GO:0000786	0.009498194
GO:0032993	0.013567061
GO:0005829	0.017340308
GO:0016607	0.018534432
GO:0031012	0.029521422
GO:0062023	0.032038892

GO:0030684	0.032244857
KEGG:03008	5.07E-06
KEGG:05168	0.000149381
REAC:R-MMU-73857	0.000244328
REAC:R-MMU-212436	0.000390542
REAC:R-MMU-74160	0.001000724
REAC:R-MMU-5625886	0.001073357
REAC:R-MMU-2559582	0.001382564
REAC:R-MMU-8936459	0.001387621
REAC:R-MMU-2559586	0.002000601
REAC:R-MMU-2299718	0.002451385
REAC:R-MMU-73728	0.004866427
REAC:R-MMU-2559583	0.006189829
REAC:R-MMU-5625740	0.011360342
REAC:R-MMU-4839726	0.01179583
REAC:R-MMU-3247509	0.01179583
REAC:R-MMU-3000157	0.01687446
REAC:R-MMU-212300	0.018876443
REAC:R-MMU-68875	0.021645848
TF:M04662	3.38E-22
TF:M00716	7.74E-21
TF:M04662_1	1.58E-19
TF:M00716_1	3.60E-18
TF:M07380	3.53E-17
TF:M00333	5.27E-16
TF:M10276	3.22E-15
TF:M00803	1.09E-14
TF:M10209	4.35E-14
TF:M00333_1	5.19E-12
TF:M02874	4.27E-10
TF:M02933	8.80E-10
TF:M00144	1.35E-09
TF:M01240	4.74E-09
TF:M01165	1.04E-08
TF:M10209_1	1.07E-08
TF:M01981	1.28E-08
TF:M01993	1.43E-08
TF:M01240_1	3.02E-08
TF:M10276_1	9.02E-08
TF:M01981_1	1.94E-07
TF:M00803_1	2.49E-07
TF:M01163	2.96E-07
TF:M00428	4.17E-07
TF:M00327_1	1.23E-06
TF:M08875	2.50E-06
TF:M00025	2.58E-06
TF:M01986	2.82E-06
TF:M07250	6.65E-06
TF:M02089	7.15E-06
TF:M10212	7.20E-06

TF:M00431	7.74E-06
TF:M00025_1	7.90E-06
TF:M08874	8.46E-06
TF:M02039	1.19E-05
TF:M01241	1.65E-05
TF:M01984	1.87E-05
TF:M02090	2.16E-05
TF:M01199	2.33E-05
TF:M01752	2.85E-05
TF:M02913	3.03E-05
TF:M10219	4.12E-05
TF:M07991	4.66E-05
TF:M07380_1	5.80E-05
TF:M02913_1	6.26E-05
TF:M09682	6.45E-05
TF:M01078	8.27E-05
TF:M10247	8.31E-05
TF:M02872	0.000159019
TF:M00144_1	0.000181986
TF:M07395	0.00018978
TF:M01991_1	0.000203078
TF:M08995	0.000209782
TF:M10211	0.000237048
TF:M01822_1	0.000238382
TF:M01991	0.000250036
TF:M00322	0.000296089
TF:M00932	0.000333696
TF:M07980	0.000356157
TF:M00327	0.000398228
TF:M00008	0.000409167
TF:M01987	0.000432852
TF:M05301	0.000434329
TF:M05455	0.000434329
TF:M05391	0.000434329
TF:M05665	0.000434329
TF:M00931	0.000440036
TF:M01598	0.00044774
TF:M00196	0.000813015
TF:M07991_1	0.000824494
TF:M01783	0.000930162
TF:M02089_1	0.00095089
TF:M02872_1	0.001144192
TF:M01989	0.001445646
TF:M10278_1	0.00146211
TF:M05494	0.00149253
TF:M00177	0.001529674
TF:M10278	0.001563477
TF:M00933	0.001667265
TF:M01979	0.001683612
TF:M03876	0.001692208

TF:M08005	0.001699939
TF:M08005_1	0.001699939
TF:M01820	0.001716857
TF:M02742	0.001877173
TF:M02874_1	0.002138515
TF:M01770_1	0.002179304
TF:M02846	0.002266692
TF:M07250_1	0.002489297
TF:M02847_1	0.002537864
TF:M00938	0.002624034
TF:M03567	0.002861487
TF:M02745	0.002983043
TF:M00920_1	0.003147472
TF:M02844	0.00350297
TF:M10219_1	0.003647684
TF:M05547	0.003879072
TF:M10234	0.003988012
TF:M02023	0.005125887
TF:M00516	0.005148034
TF:M08995_1	0.005149649
TF:M01660	0.00557826
TF:M00179	0.006439138
TF:M01985	0.006964353
TF:M01160_1	0.007087166
TF:M03807	0.007848727
TF:M00976	0.008150833
TF:M01976	0.008805034
TF:M02933_1	0.008885301
TF:M07277	0.009353792
TF:M07289	0.010462414
TF:M02102	0.011455578
TF:M10375_1	0.012450949
TF:M01990	0.012534005
TF:M00981	0.01272765
TF:M02844_1	0.013378417
TF:M01822	0.014727765
TF:M00428_1	0.014832239
TF:M07415	0.016262451
TF:M00039	0.018522823
TF:M02773	0.01875345
TF:M00189	0.023289979
TF:M02037	0.02380326
TF:M10210	0.024040443
TF:M00237	0.028382557
TF:M01989_1	0.029305086
TF:M03791	0.030385618
TF:M07395_1	0.031692958
TF:M07277_1	0.035549168
TF:M07980_1	0.036055944
TF:M00024_1	0.036359724

TF:M00976_1	0.037443424
TF:M05407	0.037810603
TF:M10233	0.038428321
TF:M01975	0.039003904
TF:M00024	0.039552393
TF:M07040	0.03958936
TF:M10216	0.040884079
TF:M01857	0.04182586
TF:M02768	0.049318817
HP:0000518	0.001779557
HP:0000517	0.003810826
HP:0000482	0.031479703

Full List of Statistically Significant DEGs in WT vs OE

GENES	logFC	AveExpr	t	P.Value	adj.P.Val	B
ENSMUSG00000005233//Spc25	-6.405	3.65231	-44	6E-12	1E-07	17.4368
ENSMUSG00000044783//A730008H23Rik	-2.386	4.92796	-28	3.4E-10	9.9E-07	14.2158
ENSMUSG00000078249//Hmga1b	5.4679	1.96459	26.5	5.6E-10	1.2E-06	13.04
ENSMUSG00000020014//Cfap54	2.419	4.16556	25.5	8E-10	1.6E-06	13.3538
ENSMUSG00000005043//Sgsh	1.9589	4.18289	24.5	1.2E-09	1.8E-06	13.0019
ENSMUSG00000073411//H2-D1	1.843	5.84656	24.2	1.3E-09	1.9E-06	12.8606
ENSMUSG00000023942//Slc29a1	2.7389	4.53443	24	1.4E-09	1.9E-06	12.8124
ENSMUSG00000053835//H2-T24	-3.068	3.22595	-23	2.3E-09	2.8E-06	12.2802
ENSMUSG00000071047//Ces1a	-6.71	-0.07608	-21	3.8E-09	4.5E-06	10.1285
ENSMUSG00000038241//Cep250	-1.458	6.87952	-20	6.4E-09	6.6E-06	11.15
ENSMUSG00000048087//Gm4737	5.2154	0.55496	19.9	7.5E-09	7.3E-06	10.2226
ENSMUSG00000052085//Dock8	2.163	4.05793	19.7	8.4E-09	7.7E-06	11.015
ENSMUSG00000043424//Eif3j2	3.1405	2.04718	19.4	9.3E-09	8.1E-06	10.7554
ENSMUSG00000024120//Lrpprc	1.3087	7.66445	19.2	1.1E-08	8.8E-06	10.6017
ENSMUSG00000034681//Rnps1	-1.218	6.17439	-19	1.4E-08	1.1E-05	10.3299
ENSMUSG00000044949//Ubtd2	1.7397	3.48511	18.2	1.7E-08	1.2E-05	10.3244
ENSMUSG00000027070//Lrp2	-1.338	4.591	-18	2.3E-08	1.5E-05	9.93734
ENSMUSG00000042229//Rabif	1.9506	6.09756	16.9	3.3E-08	1.8E-05	9.45119
ENSMUSG00000041921//Metap1d	-1.15	5.48623	-17	3.3E-08	1.8E-05	9.48743
ENSMUSG00000096696//Zfp960	-2.073	2.87899	-16	4.6E-08	2.5E-05	9.33084
ENSMUSG00000049928//Glp2r	-1.028	7.18642	-16	6E-08	3.2E-05	8.75699
ENSMUSG00000019997//Ctgf	1.822	5.97469	15.5	7.2E-08	3.6E-05	8.61262
ENSMUSG00000024055//Cyp4f13	-2.521	3.37258	-15	1.2E-07	5.3E-05	8.32372
ENSMUSG00000068396//Rpl34-ps1	3.8614	1.46888	14.5	1.3E-07	5.4E-05	8.26721
ENSMUSG00000024121//Atp6v0c	-0.816	6.44706	-14	1.4E-07	5.7E-05	7.88587
ENSMUSG00000001767//Crnkl1	0.9774	6.19905	13.8	1.9E-07	7.5E-05	7.54936
ENSMUSG00000022899//Slc15a2	1.4522	7.2033	13.8	2E-07	7.5E-05	7.45494
ENSMUSG00000096768//Erdr1	1.226	4.12068	13.7	2E-07	7.5E-05	7.69299
ENSMUSG00000046711//Hmga1	-0.908	5.59506	-14	2.2E-07	8E-05	7.43528
ENSMUSG00000079491//H2-T10	5.0798	-0.18337	12.8	3.7E-07	0.00013	6.87449
ENSMUSG00000033450//Tagap	2.3701	1.20028	12.8	3.8E-07	0.00013	7.20765
ENSMUSG00000027002//Nckap1	-0.654	7.39396	-12	5E-07	0.00016	6.45522
ENSMUSG00000036480//Prss56	-1.522	3.0567	-12	5.8E-07	0.00018	6.73802
ENSMUSG00000032549//Rab6b	-1.034	7.87856	-12	6E-07	0.00019	6.25288
ENSMUSG00000026999//Nup35	-0.88	4.84747	-12	7E-07	0.00021	6.27585
ENSMUSG00000027401//Tgm3	3.4253	1.38973	11.7	8.4E-07	0.00025	6.45507
ENSMUSG00000049291//Prss38	-2.329	0.70032	-11	1.1E-06	0.00033	6.1069
ENSMUSG00000032425//Zfp949	1.5065	6.42997	11.1	1.3E-06	0.00036	5.44307
ENSMUSG00000028788//Ptp4a2	-0.685	7.44597	-11	1.4E-06	0.00039	5.31837
ENSMUSG00000023043//Krt18	0.7525	5.29421	10.8	1.7E-06	0.00044	5.28852
ENSMUSG00000040093//Bmf	-1.78	2.99277	-11	1.8E-06	0.00046	5.56478
ENSMUSG00000003541//Ier3	0.6965	5.27964	10.5	2E-06	0.0005	5.08338
ENSMUSG00000040084//Bub1b	-1.197	4.75782	-10	2.1E-06	0.00051	5.08518
ENSMUSG00000048292//Olfr1417	-2.891	-0.28223	-10	2.2E-06	0.00051	5.36864
ENSMUSG00000033207//Mamdc2	1.2797	2.47089	10.4	2.2E-06	0.00051	5.43905
ENSMUSG00000024766//Lipo3	-1.213	3.25863	-10	2.2E-06	0.00051	5.30467
ENSMUSG00000077450//Rab11b	-0.644	6.36245	-10	2.2E-06	0.00051	4.87764
ENSMUSG00000022912//Pros1	0.8597	4.16319	10.1	2.9E-06	0.00064	4.86206

ENSMUSG00000027015//Cybrd1	-1.259	2.76512	-10	2.9E-06	0.00064	5.0948
ENSMUSG00000096255//Dynlt1b	-6.371	-0.60304	-10	3.1E-06	0.00068	4.97395
ENSMUSG00000074896//Ifit3	-1.757	2.61476	-10	3.1E-06	0.00068	5.03432
ENSMUSG00000079429//Mroh2a	-1.643	1.70631	-9.9	3.4E-06	0.00072	5.0518
ENSMUSG00000033066//Gas7	0.5607	7.82873	9.8	3.7E-06	0.00077	4.26179
ENSMUSG00000086962//Gm12248	3.717	-0.0984	9.77	3.8E-06	0.00077	4.89562
ENSMUSG00000004668//Abca13	1.1995	4.68933	9.76	3.8E-06	0.00077	4.45775
ENSMUSG00000026669//Mcm10	-1.319	2.18886	-9.8	3.8E-06	0.00077	4.87559
ENSMUSG00000029135//Fosl2	2.027	4.1157	9.73	3.9E-06	0.00078	4.52661
ENSMUSG00000020781//Tsen54	0.9886	3.22834	9.66	4.2E-06	0.00082	4.61894
ENSMUSG00000036196//Slc26a8	-1.84	2.01445	-9.6	4.4E-06	0.00085	4.75444
ENSMUSG00000033705//Stard9	0.6542	5.21668	9.58	4.5E-06	0.00086	4.20911
ENSMUSG00000097891//Gm3650	3.4135	-0.14102	9.57	4.5E-06	0.00086	4.72677
ENSMUSG00000027217//Tspan18	1.5889	2.34722	9.47	5E-06	0.00092	4.59105
ENSMUSG00000032698//Lmo2	1.7829	2.79878	9.44	5.1E-06	0.00093	4.49426
ENSMUSG00000034674//Tdg	-0.875	5.15218	-9.4	5.2E-06	0.00095	4.05869
ENSMUSG00000090693//Trim43a	-1.181	3.09156	-9.4	5.4E-06	0.00098	4.3714
ENSMUSG00000053420//Gm4792	-1.109	5.01819	-9.3	5.6E-06	0.00099	4.00166
ENSMUSG00000024084//Qpct	0.5648	5.64728	9.28	5.9E-06	0.00103	3.87129
ENSMUSG00000041528//Rnf123	-0.647	7.42219	-9.1	6.9E-06	0.00119	3.60207
ENSMUSG00000045636//Mtus1	0.5734	6.67211	9.08	7.1E-06	0.0012	3.60557
ENSMUSG00000027597//Ahcy	-0.682	4.50249	-9.1	7.1E-06	0.0012	3.8186
ENSMUSG00000000628//Hk2	-0.518	8.58221	-9.1	7.2E-06	0.0012	3.5257
ENSMUSG00000018574//Acadvl	0.7695	5.73222	9.05	7.3E-06	0.0012	3.63328
ENSMUSG00000056288//Gm11961	1.2732	5.58075	9.04	7.3E-06	0.0012	3.64608
ENSMUSG00000050211//Pla2g4e	1.2742	3.12524	8.89	8.4E-06	0.00137	3.90022
ENSMUSG00000027404//Snrpb	0.5112	5.9392	8.79	9.2E-06	0.00146	3.35953
ENSMUSG00000027048//Abcb11	-3.019	-0.30726	-8.7	9.6E-06	0.00149	4.02133
ENSMUSG00000074384//AI429214	1.4758	3.32013	8.73	9.7E-06	0.00149	3.70696
ENSMUSG00000087303//Lipo2	4.1456	-0.44494	8.73	9.8E-06	0.00149	3.98594
ENSMUSG00000073407//Gm6034	4.7082	-2.48455	8.72	9.8E-06	0.00149	3.44733
ENSMUSG00000032221//Mns1	-0.89	3.29321	-8.7	9.9E-06	0.00149	3.69849
ENSMUSG00000054134//Umodl1	-3.938	-1.68734	-8.7	1E-05	0.00153	3.69327
ENSMUSG00000041488//Stx3	-0.651	9.47642	-8.7	1E-05	0.00154	3.12448
ENSMUSG00000035299//Mid1	1.0121	5.10276	8.66	1E-05	0.00154	3.31678
ENSMUSG00000071984//Fndc1	-0.681	6.99402	-8.7	1.1E-05	0.00154	3.15741
ENSMUSG00000078552//Dcdc2b	-1.405	1.39703	-8.5	1.2E-05	0.00173	3.79554
ENSMUSG00000046101//Mcmdc2	-2.896	0.01013	-8.5	1.2E-05	0.00176	3.81189
ENSMUSG00000019935//Slc17a8	-0.785	4.11272	-8.5	1.3E-05	0.00176	3.28388
ENSMUSG00000007682//Dio2	1.8639	7.52746	8.47	1.3E-05	0.00176	2.95112
ENSMUSG00000023921//Mut	0.6767	5.12528	8.36	1.4E-05	0.00191	2.99696
ENSMUSG00000020515//Cnot8	0.5411	5.32192	8.35	1.4E-05	0.00191	2.96576
ENSMUSG00000046523//Kctd4	1.2874	2.0288	8.29	1.5E-05	0.00202	3.48316
ENSMUSG00000050822//Slc29a4	-0.77	4.12932	-8.3	1.5E-05	0.00202	3.07986
ENSMUSG00000031613//Hpgd	-0.956	4.56956	-8.3	1.5E-05	0.00203	2.9836
ENSMUSG00000044533//Rps2	-1.035	6.56534	-8.2	1.6E-05	0.00207	2.74021
ENSMUSG00000041073//Nacad	0.4762	5.95223	8.23	1.6E-05	0.00207	2.76985
ENSMUSG00000039530//Tusc3	-0.533	6.12282	-8.2	1.6E-05	0.00214	2.71478
ENSMUSG00000094707//A830019P07Rik	-4.813	-0.15653	-8.2	1.7E-05	0.0022	3.49587
ENSMUSG00000031889//D230025D16Rik	0.6062	4.53089	7.99	2E-05	0.00258	2.69102

ENSMUSG00000014813//Stc1	0.7382	4.61363	7.82	2.4E-05	0.00304	2.49298
ENSMUSG00000024319//Vps52	0.4767	6.03972	7.8	2.5E-05	0.00309	2.28807
ENSMUSG00000021464//Ror2	-2.07	0.14017	-7.8	2.5E-05	0.00309	3.14813
ENSMUSG00000036964//Trim17	1.0515	4.08806	7.77	2.5E-05	0.00313	2.52812
ENSMUSG00000027082//Tfpi	1.6133	1.74944	7.72	2.7E-05	0.00326	2.92777
ENSMUSG00000027286//Lrrc57	0.63	5.21041	7.71	2.7E-05	0.00329	2.26976
ENSMUSG00000052949//Rnf157	-0.617	7.84136	-7.6	3E-05	0.00363	1.99158
ENSMUSG00000027332//Ivd	-0.423	6.4492	-7.6	3.2E-05	0.0038	1.98135
ENSMUSG00000072774//Zfp951	-1.54	1.05816	-7.5	3.3E-05	0.00387	2.80896
ENSMUSG00000027012//Dync1i2	0.449	7.27759	7.51	3.3E-05	0.0039	1.90262
ENSMUSG00000025017//Pik3ap1	-1.795	0.40766	-7.5	3.4E-05	0.00393	2.83545
ENSMUSG00000024640//Psat1	0.6601	5.34537	7.48	3.4E-05	0.00396	1.99847
ENSMUSG00000044573//Acp1	-0.613	4.71581	-7.5	3.5E-05	0.00403	2.06523
ENSMUSG00000045319//Proser2	-0.617	4.0872	-7.3	4E-05	0.00451	2.03389
ENSMUSG00000025981//Coq10b	0.6368	4.43798	7.32	4.1E-05	0.00457	1.94497
ENSMUSG00000042622//Maff	1.33	1.81983	7.31	4.1E-05	0.00459	2.45474
ENSMUSG00000009076//Zmat5	-0.963	2.24105	-7.3	4.2E-05	0.00459	2.37305
ENSMUSG00000095990//Zfp97	-0.96	3.4073	-7.3	4.3E-05	0.00467	2.10865
ENSMUSG00000028341//Nr4a3	1.5836	3.08888	7.27	4.3E-05	0.00468	2.16622
ENSMUSG00000027284//Cdan1	0.4188	6.02375	7.18	4.7E-05	0.00509	1.57631
ENSMUSG00000032366//Tpm1	0.3906	6.75291	7.13	5E-05	0.00532	1.47391
ENSMUSG00000027875//Hmgcs2	1.1538	3.795	7.13	5E-05	0.00532	1.85409
ENSMUSG00000024112//Cacna1h	-0.532	6.67158	-7.1	5.2E-05	0.00549	1.4356
ENSMUSG00000039633//Lonrf1	-0.578	4.62406	-7.1	5.3E-05	0.00554	1.62887
ENSMUSG00000046152//Fut10	0.5577	3.92164	7.08	5.3E-05	0.00554	1.7649
ENSMUSG00000067212//H2-T23	-1.302	1.25307	-7.1	5.4E-05	0.00562	2.26323
ENSMUSG00000097574//C920006O11Rik	-0.894	2.85612	-7	5.5E-05	0.00567	1.95381
ENSMUSG00000029869//Ephb6	-0.697	3.8076	-7	5.8E-05	0.00593	1.69414
ENSMUSG00000032418//Me1	-0.391	7.19186	-7	5.8E-05	0.00593	1.29314
ENSMUSG00000038843//Gcnt1	1.702	0.71832	6.98	5.9E-05	0.00599	2.23851
ENSMUSG00000030409//Dmpk	0.6707	4.28933	6.97	6E-05	0.00599	1.56205
ENSMUSG00000027602//Map1lc3a	-0.584	4.76388	-7	6E-05	0.00599	1.47474
ENSMUSG00000050705//2310061I04Rik	-0.534	5.01708	-6.9	6.7E-05	0.0067	1.3085
ENSMUSG00000063320//1190007I07Rik	-1.08	2.15906	-6.9	6.8E-05	0.00673	1.86967
ENSMUSG00000006494//Pdk1	-0.455	5.95945	-6.8	7.1E-05	0.00695	1.14605
ENSMUSG00000067942//Zfp160	0.7001	5.19804	6.82	7.1E-05	0.00695	1.22441
ENSMUSG00000074652//Myh7b	0.7414	4.04771	6.81	7.2E-05	0.007	1.41301
ENSMUSG00000055560//Zfp459	1.6636	1.66527	6.78	7.4E-05	0.00719	1.86752
ENSMUSG00000021750//Fam107a	-0.579	4.68146	-6.8	7.6E-05	0.00726	1.2386
ENSMUSG00000055945//Prr18	1.76	0.00207	6.74	7.8E-05	0.00743	2.01962
ENSMUSG00000009734//Pou6f2	-0.651	4.12387	-6.7	8E-05	0.00746	1.28894
ENSMUSG00000068614//Actc1	1.1611	2.468	6.72	8E-05	0.00746	1.64107
ENSMUSG00000007594//Hapln4	-0.735	2.66684	-6.7	8.6E-05	0.00795	1.51453
ENSMUSG00000026864//Hspa5	0.3965	7.99012	6.64	8.7E-05	0.00799	0.83248
ENSMUSG00000053441//Adamts19	-1.433	1.17501	-6.6	8.8E-05	0.00803	1.76899
ENSMUSG00000026896//Ifih1	-0.843	2.52173	-6.6	8.9E-05	0.00803	1.51789
ENSMUSG00000001103//Sebox	0.5376	5.45626	6.62	9E-05	0.00808	0.94027
ENSMUSG00000027173//Depdc7	1.5963	-0.17182	6.55	9.7E-05	0.0087	1.81742
ENSMUSG00000000276//Dgke	0.3821	8.82534	6.54	9.8E-05	0.00874	0.69288
ENSMUSG00000019232//Etnppl	-1.03	3.79676	-6.5	9.8E-05	0.00874	1.12846

ENSMUSG00000022054//Nefm	-0.518	6.97383	-6.5	0.0001	0.0091	0.67691
ENSMUSG00000027004//Frzb	0.4963	4.61503	6.44	0.00011	0.00968	0.83803
ENSMUSG00000089789//Rdh1	1.5575	0.82431	6.41	0.00012	0.00997	1.55129
ENSMUSG00000024114//Prss41	2.7175	-0.62275	6.39	0.00012	0.01008	1.64503
ENSMUSG00000027298//Tyro3	-0.997	3.56877	-6.4	0.00012	0.01008	0.98658
ENSMUSG00000091387//Gcnt4	0.5248	4.03891	6.38	0.00012	0.01015	0.87309
ENSMUSG00000024077//Strn	-0.475	6.30789	-6.4	0.00012	0.01016	0.55167
ENSMUSG00000017737//Mmp9	-1.01	3.46003	-6.4	0.00012	0.01039	0.9632
ENSMUSG00000091405//Hist2h4	0.7204	2.78267	6.32	0.00013	0.01071	1.07282
ENSMUSG00000005649//Cabp5	0.6652	6.96839	6.32	0.00013	0.01071	0.44767
ENSMUSG00000061186//Sfmbt2	0.9096	2.44201	6.31	0.00013	0.01077	1.13271
ENSMUSG00000046275//Tusc5	-0.485	5.08804	-6.3	0.00013	0.01083	0.5779
ENSMUSG00000102869//2900097C17Rik	-0.681	5.7177	-6.3	0.00013	0.01083	0.4989
ENSMUSG00000027430//Dtd1	-0.508	4.07828	-6.3	0.00014	0.01104	0.72838
ENSMUSG00000019718//L3hypdh	0.863	3.94577	6.26	0.00014	0.01111	0.74472
ENSMUSG00000097211//BC065403	-1.713	-0.00327	-6.2	0.00014	0.01123	1.44824
ENSMUSG00000060572//Mfap2	0.658	3.39657	6.21	0.00015	0.01162	0.79746
ENSMUSG00000027288//Zfp106	0.3223	8.23381	6.21	0.00015	0.01162	0.27343
ENSMUSG00000028527//Ak4	-0.423	5.23267	-6.2	0.00015	0.01197	0.40742
ENSMUSG00000033845//Mrpl15	0.4794	4.84806	6.16	0.00015	0.01223	0.43778
ENSMUSG00000026360//Rgs2	0.5564	5.24881	6.15	0.00016	0.0123	0.36653
ENSMUSG00000039989//Cbx4	-0.47	4.56021	-6.1	0.00016	0.01253	0.45263
ENSMUSG00000054519//Zfp867	-0.668	4.33643	-6.1	0.00016	0.01276	0.46755
ENSMUSG00000034175//Rhbdd3	0.5079	3.83433	6.11	0.00016	0.01276	0.57058
ENSMUSG00000090307//1700071M16Rik	-0.983	3.2639	-6.1	0.00017	0.01304	0.66833
ENSMUSG00000024781//Lipa	0.6595	4.61083	6.07	0.00017	0.01326	0.36287
ENSMUSG00000031521//Aga	0.8411	2.34248	6.07	0.00017	0.01333	0.84016
ENSMUSG00000030235//Slco1c1	0.5511	4.09105	6.05	0.00018	0.01345	0.43774
ENSMUSG00000027610//Gss	0.511	5.53769	6.05	0.00018	0.01345	0.19605
ENSMUSG00000027236//Eif3j1	-0.508	5.35673	-6	0.00018	0.01352	0.20527
ENSMUSG00000052031//Tagap1	-0.521	4.5745	-6	0.00018	0.01371	0.30549
ENSMUSG00000027433//Xrn2	0.4179	7.03158	6.02	0.00018	0.01376	0.0516
ENSMUSG00000097073//9430037G07Rik	-0.956	3.3089	-6	0.00019	0.01403	0.53828
ENSMUSG00000026429//Ube2t	-1.903	0.19741	-6	0.00019	0.01409	1.11885
ENSMUSG00000023341//Mx2	-1.615	-0.46385	-6	0.0002	0.01461	1.12693
ENSMUSG00000069874//Irgm2	-1.342	1.49173	-6	0.0002	0.01461	0.87086
ENSMUSG00000023034//Nr4a1	0.765	5.39834	5.92	0.00021	0.01526	0.03477
ENSMUSG00000025403//Shmt2	0.6667	5.25181	5.89	0.00022	0.01581	0.00924
ENSMUSG00000048602//Morc2b	-2.155	-1.09894	-5.9	0.00022	0.01581	1.05126
ENSMUSG00000067577//A430093F15Rik	2.9282	-1.33661	5.87	0.00022	0.01596	1.03525
ENSMUSG00000035459//Stab2	-1.183	1.47474	-5.8	0.00023	0.01638	0.72534
ENSMUSG00000024248//Cox7a2l	-0.449	6.12614	-5.8	0.00023	0.01638	-0.142
ENSMUSG00000002266//Zim1	-1.163	2.11773	-5.8	0.00023	0.01638	0.58977
ENSMUSG00000038453//Srcin1	-0.4	5.71631	-5.8	0.00023	0.0165	-0.12119
ENSMUSG00000031376//Atp2b3	-0.492	4.87884	-5.8	0.00024	0.01667	-0.02697
ENSMUSG00000024190//Dusp1	0.6203	4.72759	5.78	0.00025	0.01751	-0.06192
ENSMUSG00000034427//Myo15b	-1.899	-0.23294	-5.8	0.00025	0.01753	0.87858
ENSMUSG00000052512//Nav2	-0.459	5.67341	-5.8	0.00025	0.01754	-0.20396
ENSMUSG00000033192//Lpcat2	0.4599	5.29302	5.76	0.00026	0.01762	-0.1719
ENSMUSG00000092397//C130080G10Rik	1.3756	1.53288	5.76	0.00026	0.01762	0.59626

ENSMUSG00000023075//Akirin1	0.3793	5.75488	5.75	0.00026	0.01762	-0.23241
ENSMUSG00000039385//Cdh6	-0.585	3.0384	-5.8	0.00026	0.01762	0.26424
ENSMUSG00000094724//Rnaset2b	-0.876	1.88264	-5.7	0.00026	0.01783	0.50297
ENSMUSG00000042724//Map3k9	-0.429	5.25663	-5.7	0.00027	0.01796	-0.20732
ENSMUSG00000039063//Echdc3	1.1503	1.42507	5.72	0.00027	0.01796	0.57347
ENSMUSG00000001604//Tcea3	-1.103	2.03889	-5.7	0.00027	0.01796	0.44833
ENSMUSG00000051855//Mest	0.3207	5.95029	5.72	0.00027	0.01796	-0.29348
ENSMUSG00000058952//Cfi	-1.403	0.01501	-5.7	0.00027	0.01817	0.77681
ENSMUSG00000037275//Gemin5	0.413	4.9973	5.69	0.00028	0.01843	-0.21833
ENSMUSG00000030203//Dusp16	0.4527	5.29875	5.69	0.00028	0.01843	-0.26366
ENSMUSG00000023918//Adgrf4	-1.011	1.36009	-5.7	0.00028	0.01849	0.53796
ENSMUSG00000027009//Itga4	-0.484	6.45968	-5.7	0.00029	0.01927	-0.42808
ENSMUSG00000031578//Mak16	0.5332	4.19003	5.65	0.00029	0.01927	-0.13228
ENSMUSG00000027259//Adal	0.4529	6.66285	5.64	0.0003	0.01936	-0.44871
ENSMUSG00000025867//Cplx2	-0.353	8.03071	-5.6	0.0003	0.01949	-0.50392
ENSMUSG00000024726//Carnmt1	-0.613	3.68605	-5.6	0.0003	0.01954	-0.05402
ENSMUSG00000028977//Casz1	-0.317	8.30528	-5.6	0.00031	0.01967	-0.53176
ENSMUSG00000079108//Srp54c	-0.419	5.94737	-5.6	0.00031	0.01987	-0.45471
ENSMUSG00000034311//Kif4	-0.628	3.68137	-5.6	0.00031	0.01987	-0.08309
ENSMUSG00000057899//Adgrf2	-1.119	1.08942	-5.6	0.00032	0.02023	0.4542
ENSMUSG00000098659//1110015O18Rik	-1.307	0.24335	-5.6	0.00032	0.02032	0.58141
ENSMUSG00000025876//Unc5a	-0.411	5.19694	-5.6	0.00032	0.0204	-0.4146
ENSMUSG00000099583//Hist1h3d	1.2407	0.21316	5.56	0.00033	0.02067	0.56082
ENSMUSG00000087259//2610035D17Rik	-1.582	2.58011	-5.5	0.00034	0.02139	0.0674
ENSMUSG00000034156//Tspoap1	-0.311	7.00676	-5.5	0.00035	0.02155	-0.62932
ENSMUSG00000008668//Rps18	-0.706	6.02149	-5.5	0.00036	0.02217	-0.61395
ENSMUSG00000090173//Fbxw10	-2.697	-1.80135	-5.5	0.00036	0.02231	0.56615
ENSMUSG00000040327//Cul9	-0.436	6.38237	-5.5	0.00036	0.02234	-0.65434
ENSMUSG00000069910//Spdl1	1.0154	0.78176	5.47	0.00037	0.02268	0.355
ENSMUSG00000015243//Abca1	-0.64	6.00189	-5.5	0.00037	0.02273	-0.65398
ENSMUSG00000032356//Rasgrf1	-0.356	6.92616	-5.4	0.00039	0.02326	-0.74377
ENSMUSG00000039661//Dusp26	0.3803	5.30164	5.44	0.00039	0.02326	-0.6198
ENSMUSG00000032842//Abcc10	0.4621	4.42252	5.44	0.00039	0.02326	-0.47537
ENSMUSG00000044712//Slc38a6	-0.48	4.84114	-5.4	0.00039	0.02326	-0.55215
ENSMUSG00000096910//Zfp955b	-0.524	4.33706	-5.4	0.00039	0.02326	-0.46044
ENSMUSG00000020121//Srgap1	-0.926	4.98893	-5.4	0.00039	0.02355	-0.59485
ENSMUSG00000009376//Met	0.5755	4.53506	5.41	0.0004	0.02399	-0.54185
ENSMUSG00000050668//Gpatch11	0.3123	6.13678	5.41	0.00041	0.02399	-0.75248
ENSMUSG00000038085//Cnbd2	0.5665	3.29039	5.4	0.00041	0.02405	-0.28076
ENSMUSG00000026615//Eprs	0.3484	7.62788	5.39	0.00041	0.02424	-0.83567
ENSMUSG00000037921//Ddx60	-0.764	2.24531	-5.4	0.00042	0.02447	-0.06582
ENSMUSG00000024170//Telo2	-0.419	4.12438	-5.4	0.00043	0.02509	-0.53036
ENSMUSG00000039908//Slc26a11	0.4696	3.61049	5.35	0.00044	0.02524	-0.42703
ENSMUSG00000031210//Gpr165	-0.712	2.44072	-5.3	0.00044	0.02551	-0.17502
ENSMUSG00000034656//Cacna1a	-0.493	6.06394	-5.3	0.00045	0.02554	-0.85004
ENSMUSG00000018378//2210416O15Rik	-0.311	7.16264	-5.3	0.00045	0.02561	-0.9128
ENSMUSG00000008658//Rbfox1	-0.385	6.17744	-5.3	0.00045	0.02561	-0.86822
ENSMUSG00000061232//H2-K1	-0.863	3.92277	-5.3	0.00046	0.02576	-0.54335
ENSMUSG00000074863//Gm45951	1.7425	1.26958	5.29	0.00047	0.02648	0.01521
ENSMUSG00000027305//Ndufaf1	0.4585	4.08636	5.29	0.00047	0.02648	-0.61512

ENSMUSG00000097727//F630040K05Rik	-0.39	5.69097	-5.3	0.00048	0.02669	-0.88842
ENSMUSG00000058153//Sez6l	-0.829	5.56876	-5.3	0.00048	0.02669	-0.87841
ENSMUSG00000048905//4930539E08Rik	1.3215	0.21076	5.28	0.00048	0.02677	0.17875
ENSMUSG00000025161//Slc16a3	-0.565	5.01818	-5.3	0.00048	0.02683	-0.81806
ENSMUSG00000022122//Ednrb	0.7009	3.6458	5.27	0.00049	0.02683	-0.55069
ENSMUSG00000024079//Eif2ak2	-0.513	3.4379	-5.3	0.00049	0.02683	-0.50484
ENSMUSG00000023236//Scg5	0.4561	7.47101	5.25	0.0005	0.02742	-1.04094
ENSMUSG00000073437//D330041H03Rik	0.6823	3.08623	5.24	0.00051	0.02762	-0.46403
ENSMUSG00000033615//Cplx1	-0.33	6.67206	-5.2	0.00052	0.02799	-1.04363
ENSMUSG00000031358//Msl3	0.7307	4.91098	5.22	0.00052	0.02822	-0.88142
ENSMUSG00000010086//Rnf112	-0.688	3.16639	-5.2	0.00053	0.02855	-0.53304
ENSMUSG00000067786//Nnat	0.6387	4.89195	5.2	0.00053	0.02855	-0.8973
ENSMUSG00000027074//Slc43a3	1.1723	1.56682	5.2	0.00054	0.02876	-0.18357
ENSMUSG00000024793//Tnfrsf25	1.279	0.69815	5.18	0.00055	0.02919	-0.0286
ENSMUSG00000032252//Glce	0.434	5.30927	5.18	0.00055	0.02923	-0.99789
ENSMUSG00000042851//Zc3h6	-0.331	5.30737	-5.2	0.00055	0.02923	-0.99851
ENSMUSG00000024042//Sik1	0.9659	4.8286	5.17	0.00055	0.02923	-0.92853
ENSMUSG00000097886//Gsg1l2	-0.667	3.47418	-5.2	0.00056	0.02935	-0.65515
ENSMUSG00000028931//Kcnab2	-0.36	7.94351	-5.2	0.00056	0.02935	-1.16976
ENSMUSG00000034570//Inpp5j	-0.426	4.11904	-5.2	0.00057	0.02969	-0.81751
ENSMUSG00000073436//Eme2	-0.664	2.52116	-5.1	0.00057	0.02989	-0.46478
ENSMUSG00000090035//Galnt4	-0.287	6.35113	-5.1	0.00058	0.02995	-1.14719
ENSMUSG00000030096//Slc6a6	-0.327	10.8488	-5.1	0.00058	0.02997	-1.23503
ENSMUSG00000096140//Ankrd66	-2.931	-1.44103	-5.1	0.00059	0.0304	0.1123
ENSMUSG00000032359//Ctsh	-0.513	3.70761	-5.1	0.0006	0.0309	-0.79448
ENSMUSG00000031075//Ano1	-0.783	1.53123	-5.1	0.0006	0.0309	-0.29842
ENSMUSG00000017167//Cntnap1	-0.452	6.28165	-5.1	0.00061	0.03099	-1.19657
ENSMUSG00000020814//Mxra7	-0.332	6.95849	-5.1	0.00061	0.03115	-1.23825
ENSMUSG00000034165//Ccnd3	0.3575	5.44535	5.09	0.00062	0.03131	-1.13675
ENSMUSG00000020806//Rhbdf2	-0.998	3.03209	-5.1	0.00062	0.03155	-0.6733
ENSMUSG00000027487//Cdk5rap1	-0.814	3.35215	-5.1	0.00063	0.03175	-0.75858
ENSMUSG00000001774//Chordc1	0.4003	6.06357	5.08	0.00063	0.03175	-1.22261
ENSMUSG00000070644//Etnk2	-1.479	-0.07145	-5.1	0.00064	0.03196	-0.06689
ENSMUSG00000063889//Crem	0.4197	4.02875	5.07	0.00064	0.032	-0.92842
ENSMUSG00000031765//Mt1	0.4032	4.19828	5.04	0.00066	0.03302	-1.00467
ENSMUSG00000005804//Bloc1s6	0.3993	4.5531	5.04	0.00067	0.03302	-1.07875
ENSMUSG00000026655//Fam107b	0.315	6.57901	5.02	0.00069	0.03395	-1.34504
ENSMUSG00000068154//Insm1	0.377	5.18594	5.01	0.00069	0.03395	-1.21983
ENSMUSG00000021919//Chat	-0.547	3.42892	-5	0.00069	0.03399	-0.87893
ENSMUSG00000001039//B9d1	0.4285	3.66184	5	0.0007	0.03421	-0.94676
ENSMUSG00000078851//Hist3h2a	0.6506	4.97784	5	0.0007	0.03421	-1.21161
ENSMUSG00000034075//Zdhhc5	-0.273	7.95324	-5	0.00071	0.03421	-1.42127
ENSMUSG00000079470//Utp14b	0.456	6.3845	5	0.00071	0.03421	-1.36421
ENSMUSG00000095253//Zfp799	0.332	5.3923	4.99	0.00071	0.03421	-1.27695
ENSMUSG00000024887//Asah2	-0.528	3.28526	-5	0.00072	0.03449	-0.87857
ENSMUSG00000086938//4930481A15Rik	-0.922	2.49377	-5	0.00072	0.03449	-0.69497
ENSMUSG00000090877//Hspa1b	1.2522	5.6034	4.97	0.00073	0.0352	-1.34178
ENSMUSG00000019944//Rhobtb1	-0.499	3.66033	-5	0.00074	0.0352	-0.99623
ENSMUSG00000050556//Kcnb1	-0.29	9.52501	-5	0.00075	0.03566	-1.50342
ENSMUSG00000020808//Pimreg	-0.875	0.91787	-5	0.00075	0.0357	-0.39701

ENSMUSG00000028790//Khdrbs1	-0.311	6.99298	-4.9	0.00076	0.03595	-1.46887
ENSMUSG00000061462//Obscn	0.7682	2.66269	4.94	0.00076	0.03595	-0.79612
ENSMUSG00000061654//Spry3	-0.485	4.90655	-4.9	0.00076	0.03599	-1.28702
ENSMUSG00000070476//Fam217b	-0.355	5.03246	-4.9	0.00077	0.03601	-1.31038
ENSMUSG00000021510//Zfp729a	0.3317	5.7718	4.93	0.00078	0.03642	-1.41845
ENSMUSG00000024210//Ip6k3	-0.81	2.27036	-4.9	0.00079	0.03665	-0.73903
ENSMUSG00000028804//Csmd2	-0.577	4.52998	-4.9	0.00079	0.03665	-1.2525
ENSMUSG00000018339//Gpx3	-1.058	6.62244	-4.9	0.00079	0.03665	-1.49953
ENSMUSG00000078532//Nkain1	-0.913	1.18851	-4.9	0.00079	0.03665	-0.50868
ENSMUSG00000060639//Hist1h4i	0.758	3.10771	4.91	0.00079	0.03665	-0.94932
ENSMUSG00000037447//Arid5a	0.6256	3.31539	4.91	0.0008	0.03665	-0.99908
ENSMUSG00000026179//Pnkd	-0.304	6.11259	-4.9	0.00081	0.03707	-1.48963
ENSMUSG00000032392//Parp16	0.6338	3.04054	4.89	0.00081	0.03726	-0.95771
ENSMUSG00000070304//Scn2b	-0.358	5.94521	-4.9	0.00082	0.03767	-1.4992
ENSMUSG00000073421//H2-Ab1	-1.59	-0.31094	-4.9	0.00083	0.03791	-0.30515
ENSMUSG00000084960//B430010I23Rik	1.5973	0.40263	4.87	0.00084	0.03797	-0.41202
ENSMUSG00000036398//Ppp1r11	0.2945	5.3625	4.87	0.00084	0.038	-1.45451
ENSMUSG00000028410//Dnaja1	0.2972	7.61713	4.87	0.00084	0.03821	-1.60604
ENSMUSG00000024039//Cbs	-1.052	1.00377	-4.9	0.00085	0.03834	-0.54336
ENSMUSG00000079339//Ifit1bl1	-1.459	0.6889	-4.9	0.00086	0.03871	-0.49359
ENSMUSG00000030283//St8sia1	-0.256	7.78376	-4.8	0.00087	0.03877	-1.63714
ENSMUSG00000020303//Stc2	-0.971	1.46543	-4.8	0.00087	0.03877	-0.66208
ENSMUSG00000049555//Tmie	-0.97	1.6014	-4.8	0.00088	0.03904	-0.70441
ENSMUSG00000054909//Wbscr25	2.1713	-1.90856	4.83	0.00089	0.03954	-0.26802
ENSMUSG00000024048//Myl12a	0.5637	4.5624	4.83	0.00089	0.03954	-1.39345
ENSMUSG00000041014//Nrg3	-0.443	3.51694	-4.8	0.0009	0.03972	-1.17825
ENSMUSG00000020744//Slc25a19	-0.379	5.90174	-4.8	0.0009	0.03972	-1.59095
ENSMUSG00000074657//Kif5a	-0.358	7.98558	-4.8	0.0009	0.03972	-1.68871
ENSMUSG00000017288//Vps53	-0.381	5.78677	-4.8	0.00091	0.03972	-1.58538
ENSMUSG00000046230//Vps13a	0.4443	6.1883	4.81	0.00092	0.04027	-1.63596
ENSMUSG00000011486//Slc25a41	1.5735	0.19282	4.8	0.00093	0.04064	-0.48939
ENSMUSG00000035161//Ints6	0.3072	5.59877	4.79	0.00093	0.04064	-1.59832
ENSMUSG00000027400//Pdyn	-0.447	3.80619	-4.8	0.00094	0.0407	-1.287
ENSMUSG00000020412//Ascc2	0.2838	5.7599	4.79	0.00094	0.04074	-1.62385
ENSMUSG00000027287//Snap23	0.3237	5.24015	4.79	0.00094	0.04074	-1.56443
ENSMUSG00000020848//Doc2b	-0.362	6.73475	-4.8	0.00095	0.04074	-1.69491
ENSMUSG00000045215//Asxl3	-0.569	3.10368	-4.8	0.00096	0.04118	-1.14626
ENSMUSG00000060212//Pcnx2	-0.609	4.26546	-4.8	0.00096	0.04126	-1.41446
ENSMUSG00000046329//Slc25a23	-0.3	7.38934	-4.8	0.00097	0.04138	-1.74515
ENSMUSG00000033715//Akr1c14	-1.984	-0.70805	-4.8	0.00097	0.04138	-0.41714
ENSMUSG00000040738//Ints8	0.2898	5.86397	4.77	0.00097	0.04138	-1.6693
ENSMUSG00000043857//Mgat5b	-0.668	3.78323	-4.8	0.00097	0.04138	-1.32291
ENSMUSG00000024654//Asrgl1	-0.277	6.78819	-4.8	0.00099	0.04173	-1.74144
ENSMUSG00000047638//Nr1h4	-0.761	1.59785	-4.8	0.00099	0.04196	-0.8337
ENSMUSG00000072663//Spef2	0.9436	2.02979	4.75	0.001	0.04206	-0.93653
ENSMUSG00000001467//Cyp51	0.2816	6.26101	4.75	0.001	0.04206	-1.72966
ENSMUSG00000021091//Serpina3n	-1.118	2.48534	-4.7	0.001	0.0421	-1.04792
ENSMUSG00000032327//Stra6	-0.841	2.20918	-4.7	0.00101	0.04231	-0.99071
ENSMUSG00000073409//H2-Q6	-2.326	-0.44181	-4.7	0.00101	0.04231	-0.4867
ENSMUSG00000056895//Hist3h2ba	0.4806	3.76211	4.71	0.00105	0.04386	-1.40061

ENSMUSG00000024176//Sox8	-0.482	4.06935	-4.7	0.00106	0.04406	-1.47622
ENSMUSG00000040606//Kazn	-0.514	4.41009	-4.7	0.00107	0.04417	-1.5527
ENSMUSG00000030703//Gdpd3	0.6231	4.22241	4.7	0.00107	0.04421	-1.51748
ENSMUSG00000086003//B230206L02Rik	-1.007	1.4035	-4.7	0.00108	0.04436	-0.87746
ENSMUSG00000066441//Rdh11	0.3038	5.86902	4.69	0.00108	0.04436	-1.78162
ENSMUSG00000032177//Pde4a	-0.375	5.47011	-4.7	0.00108	0.04436	-1.74132
ENSMUSG00000039850//Endov	-0.384	4.83245	-4.7	0.00108	0.04436	-1.64848
ENSMUSG00000032911//Cspg4	-1.026	0.55021	-4.7	0.00109	0.04458	-0.71138
ENSMUSG00000052387//Trpm3	-0.278	7.73821	-4.7	0.00111	0.04535	-1.90315
ENSMUSG00000051111//Sv2c	-0.603	4.45993	-4.7	0.00112	0.04542	-1.61233
ENSMUSG00000023979//Guca1b	-0.285	9.62552	-4.7	0.00112	0.04556	-1.93885
ENSMUSG00000020893//Per1	0.3018	7.07894	4.66	0.00113	0.04561	-1.89576
ENSMUSG00000031822//Gse1	-0.262	6.79593	-4.7	0.00114	0.04601	-1.89813
ENSMUSG00000001098//Kctd10	0.3467	4.4836	4.65	0.00115	0.04601	-1.64839
ENSMUSG00000031492//Chrnb3	-0.314	6.39933	-4.6	0.00115	0.04601	-1.88975
ENSMUSG00000015656//Hspa8	0.4243	10.0081	4.65	0.00116	0.04601	-1.9712
ENSMUSG00000024524//Gnal	-0.362	5.28181	-4.6	0.00116	0.04601	-1.78838
ENSMUSG00000043635//Adamts3	-0.362	6.43617	-4.6	0.00116	0.04601	-1.89646
ENSMUSG00000041263//Rusc1	-0.427	4.75831	-4.6	0.00117	0.04627	-1.72005
ENSMUSG00000029552//Tes	0.473	3.13629	4.63	0.00118	0.04659	-1.37743
ENSMUSG00000060992//Copz1	0.2728	5.74827	4.63	0.00119	0.04673	-1.872
ENSMUSG00000028795//Ccdc28b	-0.517	3.8843	-4.6	0.0012	0.04689	-1.56343
ENSMUSG00000024299//Adamts10	-0.601	4.04434	-4.6	0.00121	0.04729	-1.61001
ENSMUSG00000030519//Apba2	-0.337	5.49703	-4.6	0.00124	0.04827	-1.88632
ENSMUSG00000002835//Chaf1a	-0.628	2.75248	-4.6	0.00124	0.04835	-1.33639
ENSMUSG00000026587//Astn1	-0.349	6.05211	-4.6	0.00125	0.04852	-1.95235
ENSMUSG00000032878//Ccdc85a	-0.305	4.75519	-4.6	0.00125	0.04852	-1.78904
ENSMUSG00000036473//Ntn3	-0.257	7.92734	-4.6	0.00127	0.04925	-2.05185
ENSMUSG00000026567//Adcy10	-1.136	0.35271	-4.6	0.00129	0.04991	-0.84961

Gene Ontology Terms- Downregulated Terms/Processes in the OE

source	term_name	term_id	adjusted_p_value
GO:MF	ion binding	GO:0043167	0.003290166
GO:MF	ion transmembrane transporter activity	GO:0015075	0.00358121
GO:MF	transmembrane transporter activity	GO:0022857	0.004129219
GO:MF	transporter activity	GO:0005215	0.005624958
GO:MF	peptide antigen binding	GO:0042605	0.014859242
GO:MF	inorganic molecular entity transmembrane transporter activity	GO:0015318	0.015823505
GO:BP	neurotransmitter transport	GO:0006836	0.003211089
GO:BP	chemical synaptic transmission	GO:0007268	0.014648675
GO:BP	anterograde trans-synaptic signaling	GO:0098916	0.014648675
GO:BP	trans-synaptic signaling	GO:0099537	0.017294907
GO:BP	synaptic signaling	GO:0099536	0.029720788
GO:BP	modulation of chemical synaptic transmission	GO:0050804	0.032034039
GO:BP	regulation of trans-synaptic signaling	GO:0099177	0.032824833
GO:CC	synapse	GO:0045202	0.000162416
GO:CC	neuron projection	GO:0043005	0.000724889
GO:CC	cell junction	GO:0030054	0.003690365
GO:CC	distal axon	GO:0150034	0.004311481
GO:CC	presynapse	GO:0098793	0.004541246
GO:CC	MHC protein complex	GO:0042611	0.016778622
GO:CC	somatodendritic compartment	GO:0036477	0.017430032
GO:CC	axon	GO:0030424	0.030446824
GO:CC	neuronal cell body	GO:0043025	0.03352833
GO:CC	MHC class I protein complex	GO:0042612	0.037587264
GO:CC	plasma membrane protein complex	GO:0098797	0.048846183
GO:CC	cell body	GO:0044297	0.049782537
KEGG	Synaptic vesicle cycle	KEGG:04721	0.011804415
KEGG	Graft-versus-host disease	KEGG:05332	0.025690405
KEGG	Allograft rejection	KEGG:05330	0.025690405
KEGG	Type I diabetes mellitus	KEGG:04940	0.045051318
TF	Factor: ZF5; motif: GSGCGCGR	TF:M00716	0.013130211
TF	Factor: CACD; motif: CCACRCCC; match class: 1	TF:M01113_1	0.027734229
TF	Factor: KLF; motif: GGGNGGGG	TF:M07461	0.044629481

Gene Ontology Terms- Upregulated Terms/Processes in the OE

source	term_name	term_id	adjusted_p_value
GO:MF	unfolded protein binding	GO:0051082	0.014141123
GO:CC	intracellular anatomical structure	GO:0005622	0.000725978
GO:CC	intracellular organelle	GO:0043229	0.011268107
GO:CC	intracellular membrane-bounded organelle	GO:0043231	0.045222016
TF	Factor: ZF5; motif: GSGCGCGR	TF:M00716	0.000124395
TF	Factor: FOXN4; motif: NNWANNCGWMCGCGTCNNNNMT	TF:M04662	0.005239864
TF	Factor: BEN; motif: CAGCGRNV	TF:M01240	0.01753581
